# Synthetic studies toward longeracemine: a SmI_2_-mediated spirocyclization and rearrangement cascade to construct the 2-azabicyclo[2.2.1]heptane framework†

**DOI:** 10.1039/d0sc03422c

**Published:** 2020-07-30

**Authors:** Keita Komine, Kyle M. Lambert, Quentin R. Savage, Joshua B. Cox, John L. Wood

**Affiliations:** Department of Chemistry and Biochemistry, Baylor University One Bear Place 97348 Waco TX 76798 USA john_l_wood@baylor.edu

## Abstract

Longeracemine, a member of the *Daphniphyllum* family of alkaloids contains a novel carbon framework featuring a highly functionalized 2-azabicyclo[2.2.1]heptane core as part of an overall 5/6/5/5/6/5 skeleton. A synthetic intermediate containing the core of longeracemine has been efficiently prepared by employing a stereoselective SmI_2_-mediated cascade reaction to advance a 7-azabicyclo[2.2.1]heptadiene to a 2-azabicyclo[2.2.1]heptene that is functionally poised for conversion to the natural product.

## Abstract 2

A synthetic intermediate containing the core of longeracemine, that is functionally poised for conversion to the natural product, has been efficiently prepared by employing a stereoselective SmI_2_-mediated cascade reaction.

## Graphical abstract

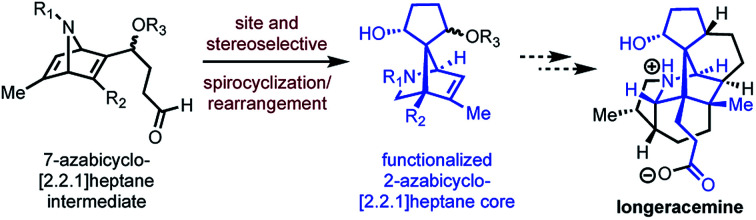

## Introduction

**Fig. 1:**
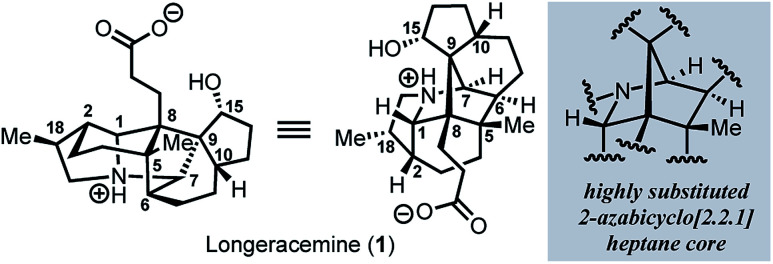
Longeracemine (**1**) and the highly substituted 2-azabicyclo-[2.2.1]heptane core.

Since the isolation of daphnimacrine in 1909 by Yagi,^1*a*^ over 320 members of the *Daphniphyllum* family have been isolated from evergreen shrubs and trees from southeast Asia.^1^*Daphniphyllum* alkaloids have been shown to exhibit a wide variety of biological activities including cytotoxicity against a number of cancer cell lines, antioxidant properties, inhibition of platelet aggregation, insecticidal, and anti-HIV activity.^1*c*,2*g*^ In addition to their promising pharmacological profiles, these alkaloids are structurally diverse and possess a plethora of polycyclic fused structures, and as such, have gained a wide-interest in the synthetic community as highly sought-after and attractive targets for total synthesis.^2^ The *Daphniphyllum* alkaloids isolated to date, have been found to contain 35 unique carbon frameworks,^2*g*^ and longeracemine, which was isolated in 2013 by Di and co-workers from the fruits of *Daphniphyllum longeracemosum*,^3^ possesses a novel structure consisting of a 2-azabicyclo[2.2.1]heptane core. Longeracemine's bicyclic system features a tertiary amine, eight alkyl substituents, and three contiguous quaternary carbons within an overall 5/6/5/5/6/5 skeleton (Fig. 1).

**Scheme 1:**
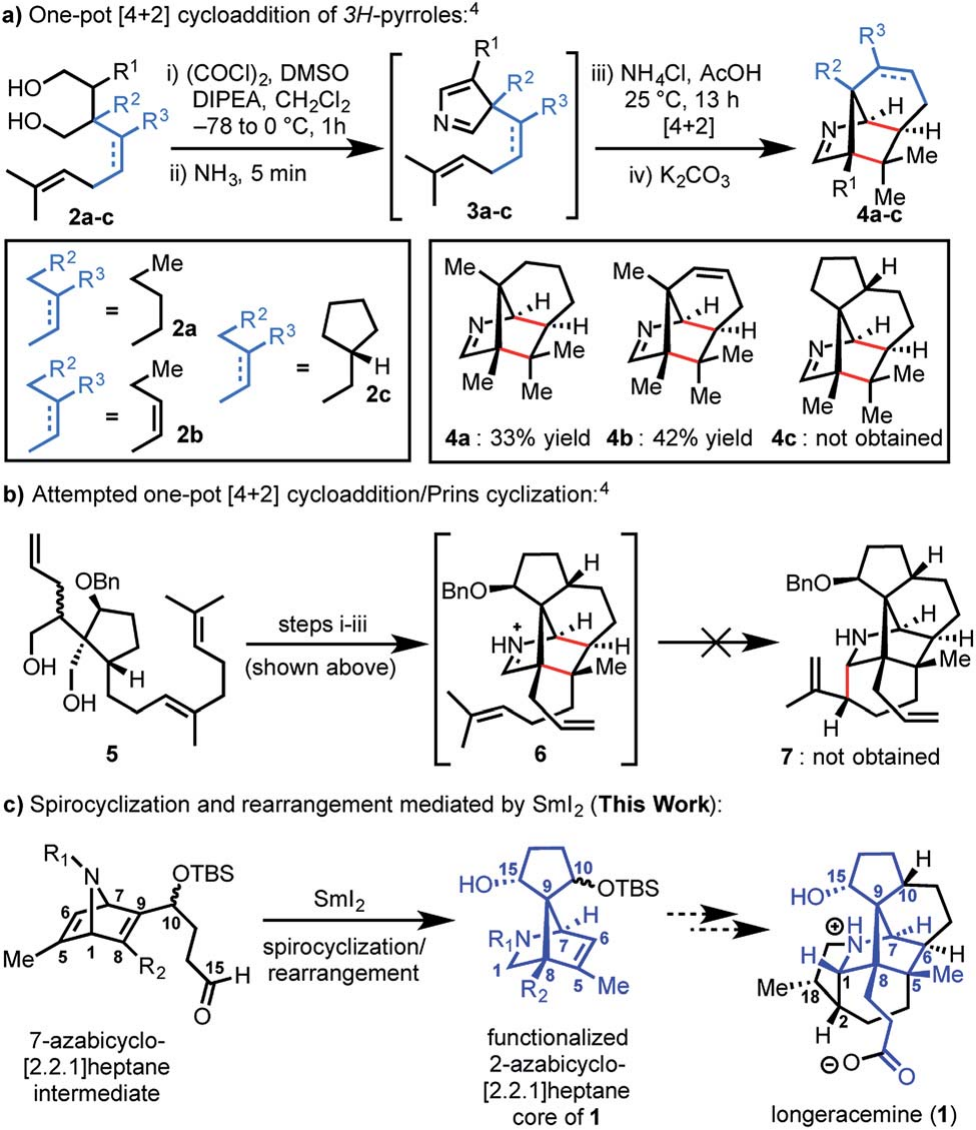
(a) Our previous work involving an intramolecular [4+2] cycloaddition; (b) our attempted tandem [4+2] cycloaddition/Prins cyclization; (c) SmI_2_ mediated spirocyclization/rearrangement to afford the 2-azabicyclo[2.2.1]heptane core of **1**; DMSO = dimethylsulfoxide, DIPEA = diisopropylethylamine, Bn = benzyl, TBS = *tert*-butyldimethylsilyl.

Recently, we reported an efficient assembly of the 2-azabicyclo[2.2.1]heptane core of **1** through an intramolecular [4+2] cycloaddition of 3*H*-pyrroles (Scheme 1).^4^ Exposure of diols **2a-b** to Swern oxidation, amine condensation, and an acid-mediated [4+2] cycloaddition afforded the desired bicycles **4a** and **4b** in 33% and 42% yields, respectively, in single pot transformations; **2c** failed to afford **4c** (Scheme 1a). Employing optimized conditions, the reaction of a more functionalized diol (**5**), to access **7**, which is better poised for elaboration to longeracemine (**1**) compared to **4a-c** was explored (Scheme 1b). However, the [4+2] cycloaddition was found to be highly substrate dependent and the reaction of **5** failed to provide desired compound **7**.^4^ Based on these results, an alternative approach was required to construct the 2-azabicyclo[2.2.1]heptane framework. Herein, we report that a novel SmI_2_-mediated cascade reaction, involving a spirocyclization and rearrangement, affords a 2-azabicyclo[2.2.1]heptene framework that is suitably functionalized for advancement to **1** (Scheme 1c). The reaction proceeds in good yield, with excellent regio- and stereoselectivity.

## Results and discussion

**Scheme 2:**
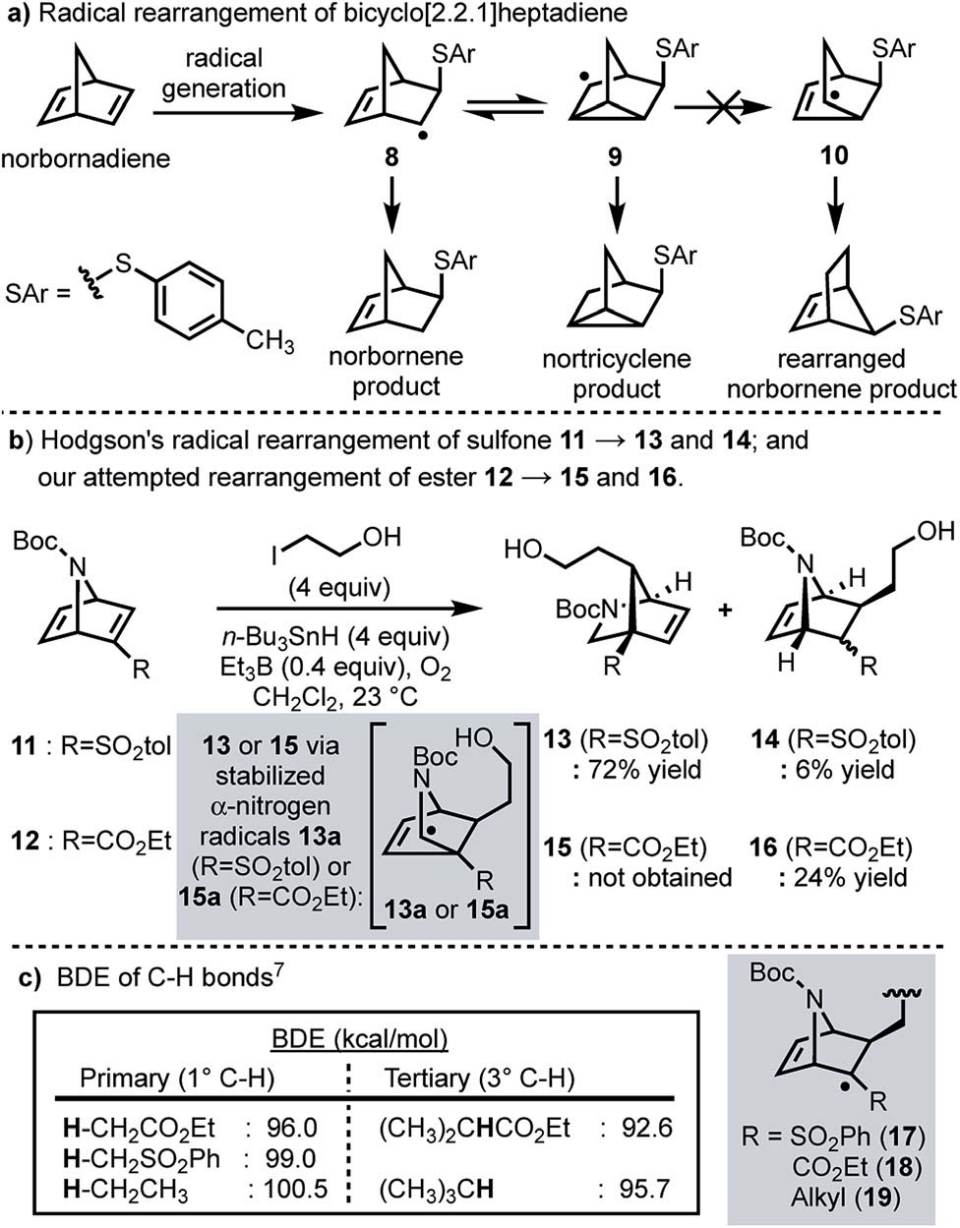
(a) Radical rearrangement of bicyclo[2.2.1]heptadiene; (b) radical rearrangements of 7-azabicyclo[2.2.1]heptadienes; (c) BDEs of relevant C–**H** bonds.

In 1958, Cristol and co-workers reported that the addition of aryl mercaptans to norbornadiene under radical conditions generates homoallyl radical **8**, which undergoes a reversible ring closure to form nortricyclene radical **9**, resulting in a (1 : 1) mixture of nortricyclane and norbornene products; products resulting from rearranged norbornene radical **10** were never observed (Scheme 2a).^5^ More recently, Hodgson and co-workers have reported a selective radical rearrangement of 7-azabicyclo[2.2.1]heptadienes that is guided by the increased stability of a resultant α-nitrogen radical *versus* a secondary alkyl radical (*cf.***13a** to **10**; Scheme 2b).^6,7^ In one instance, Hodgson demonstrated that treatment of vinyl sulfone **11** with 2-iodoethanol and an excess of tributyltin hydride in presence of a catalytic amount of triethylborane and oxygen afforded the rearranged adduct **13** in 72% yield.^7*g*^ Given the range of substrates evaluated and seemingly robust nature of this transformation,^7^ we envisioned that this radical rearrangement could be performed in more complex systems and perhaps prove useful for construction of the bicyclic framework of longeracemine (**1**).

In considering the application of Hodgson's rearrangement to **1**, we first focused our attention on the sulfone moiety. Although advancing the derived neopentyl sulfones might be possible in a synthesis, substrates wherein sulfur is replaced by carbon were envisioned as much more viable. Thus, in an initial study we evaluated whether this rearrangement could be performed on the respective ester. To this end we prepared ethyl ester **12** which, upon exposure to Hodgson's conditions, was found to exclusively afford the non-rearranged product **16** in 24% yield (Scheme 2b). The failure of the intermediate radical derived from **12** to undergo rearrangement and furnish **15**, compared to the efficient conversion of **11** to **13**, clearly indicated that the electron-withdrawing nature of the substituent alpha to the intermediate radical (sulfone *vs.* ester) was important as well, not just the increased stability of the terminal α-nitrogen radical (*i.e.***13a***versus***10**). Thus, we considered the differences in the known bond dissociation energies (BDE) of C–**H** bonds adjacent to ester, sulfone, and alkyl moieties (Scheme 2c).^8^ The BDE of a primary C_α_–**H** bond for an ester is lower than that of a sulfone or an alkyl group; 96.0 *vs.* 99.0 and 100.5 kcal mol^−1^, respectively (Scheme 2c). The BDE of the C_α_–**H** bond of a tertiary sulfone has, to our knowledge, not been reported, however, by analogy one might anticipate it as being similar to the C–**H** bond of a tertiary alkyl group (95.7 kcal mol^−1^; Scheme 2c). Thus, we anticipated that replacing the sulfone with an alkyl group (*cf.*, **17** and **19**) would result in a slightly more reactive intermediate, thereby facilitating the desired rearrangement.

**Scheme 3:**
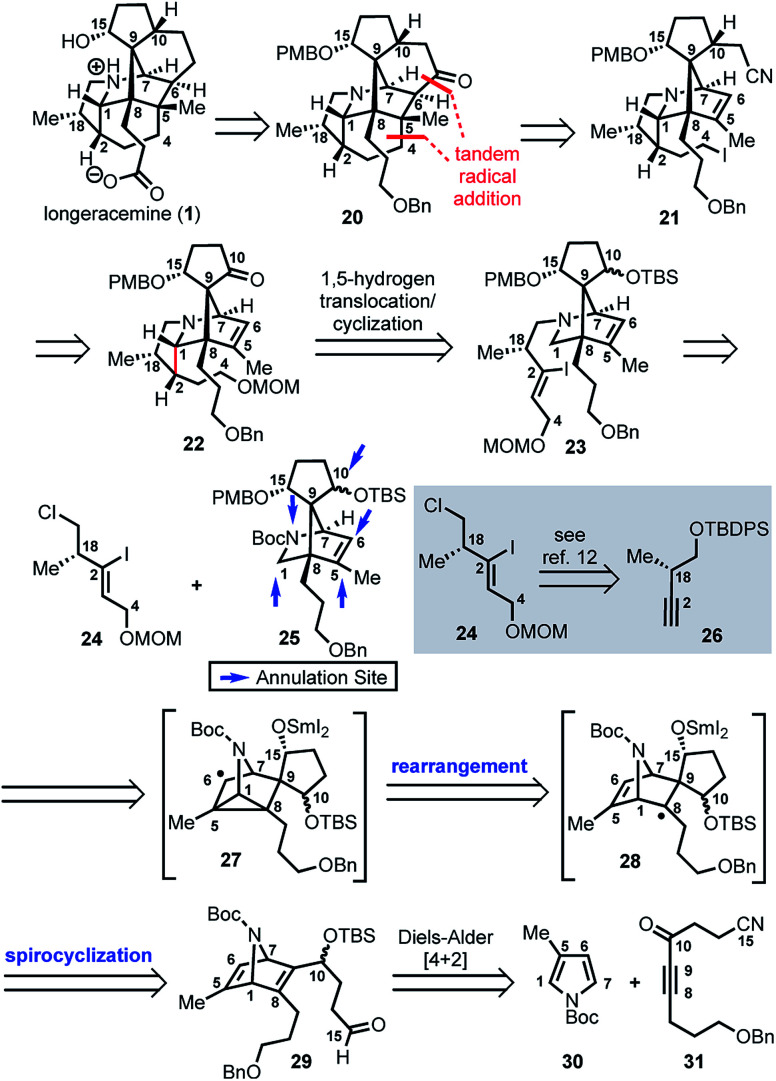
Retrosynthesis of longeracemine (**1**); PMB = *p*-methoxybenzyl, Boc = *t*-butoxycarbonyl.

Based on these preliminary results, as illustrated retrosynthetically in Scheme 3, we envisioned that longeracemine (**1**) would be derived from ketone **20**, which would, in turn, arise from iodide **21***via* tandem radical cyclization (Scheme 3).^9^ To access iodide **21**, we considered an approach from **22** that called for homologation at the C10 ketone with acetonitrile^10^ and iodination at C4. For the stereoselective synthesis of **22**, we seek to employ vinyl iodide **23** in a SnBu_3_H or SmI_2_-mediated 1,5-hydrogen translocation to afford an α-aminoalkyl radical which would be followed by a 5-*exo*-trig cyclization.^11^ Vinyl iodide **23** would arise from deprotection and *N*-alkylation of **25** with **24**, available from **26**.^12^ Key intermediate **25** would, in turn, arise from **29***via* a tandem radical spirocyclization/rearrangement sequence triggered by initial formation of a Sm-derived ketyl radical.^13,14^ Spirocyclization of the ketyl in a 5-*exo*-trig-fashion would afford tertiary radical **28** which, in contrast to the analogous radical derived from **12**, was anticipated to undergo rearrangement to intermediate cyclopropane **27**. Subsequent homolysis of the C1–C5 bond in **27** and hydrogen atom abstraction,^7*b*,15^ or further reduction of the stabilized α-nitrogen radical by excess SmI_2_ to the secondary Sm-anion^16^ followed by protonation, would then deliver **25**. We anticipated that **29** could be prepared from *N*-Boc pyrrole **30** and dienophile **31** by a Diels–Alder reaction.

**Scheme 4:**
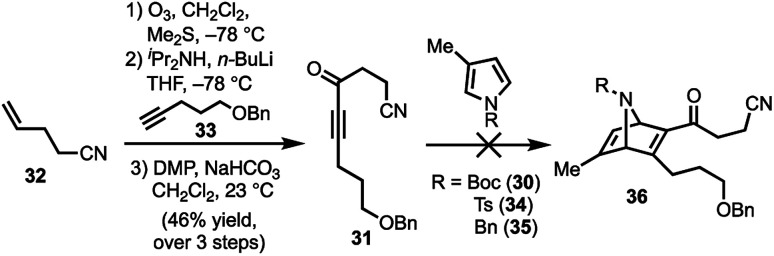
Attempt to perform the Diels–Alder reaction of **31** with pyrroles **30**, **34**, and **35**; DMP = Dess–Martin periodinane.

Our synthetic efforts began with the preparation of aldehyde **29***via* a Diels–Alder reaction (Scheme 4). Ozonolysis of commercially available nitrile **32**, followed by nucleophilic alkynylation with **33**,^17^ and oxidation of the resulting alcohol furnished **31**. Next, we examined various thermal and Lewis acid conditions to promote the [4+2] cycloaddition of pyrroles **30**, **34**, and **35**; however, desired bicycle **36** was not obtained.^18,19^ Since pyrroles are generally poor dienes for the Diels–Alder reaction and can react with dienophiles to afford Michael addition products, we sought to alter the electron-withdrawing nature of the dienophile (Table 1).^20,21^

**Table 1:** Diels–Alder reaction of pyrroles (**30**, **34**, or **35**) with **37**

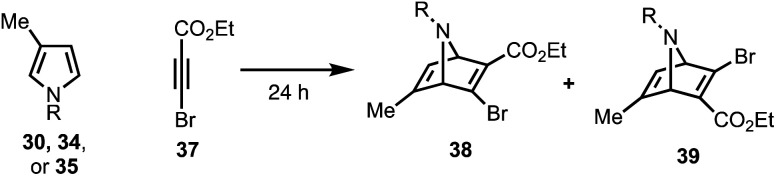					
Entry	R	Temperature	Pyrrole (**30**, **34**, **35**)	Alkyne (**37**)	Yield^a^ (%)
1	Boc (**30**)	90 °C	2 equiv.	1 equiv.	39% (4 : 1)
2	Ts (**34**)	90 °C	2 equiv.	1 equiv.	14% (4 : 1)
3	Boc (**30**)	90 °C	1 equiv.	2 equiv.	55% (5 : 1)
4	Ts (**34**)	90 °C	1 equiv.	2 equiv.	21% (3 : 1)
5	Ts (**34**)	110 °C	1 equiv.	2 equiv.	0%
6	Bn (**35**)	90 °C	1 equiv.	2 equiv.	0%

a Yield (%) refers to the isolated yields of the mixture; ratios of the products (**38** : **39**) were determined by ^1^H NMR analysis of the mixture; Ts = *p*-toluenesulfonyl.

To this end, we examined the use of ethyl 3-bromopropiolate **37**, under thermal conditions (Table 1). Treatment of **30**^22^ or **34**^23^ with **37**^24^ at 90 °C under neat conditions, afforded mixtures (4 : 1) of *N*-Boc and *N*-Ts protected regiomers **38** and **39** in 39% and 14% yield, respectively (entries 1–2), as well as Michael adducts. Reversing the ratio of pyrrole to alkyne from 2 : 1 to 1 : 2, respectively, improved the overall yields (entries 3–4) while increasing the temperature proved ineffective (entry 5). Notably, the use of a relatively electron-rich *N*-benzyl pyrrole (**35**) failed to furnish any cycloaddition products (entry 6).

**Scheme 5:**
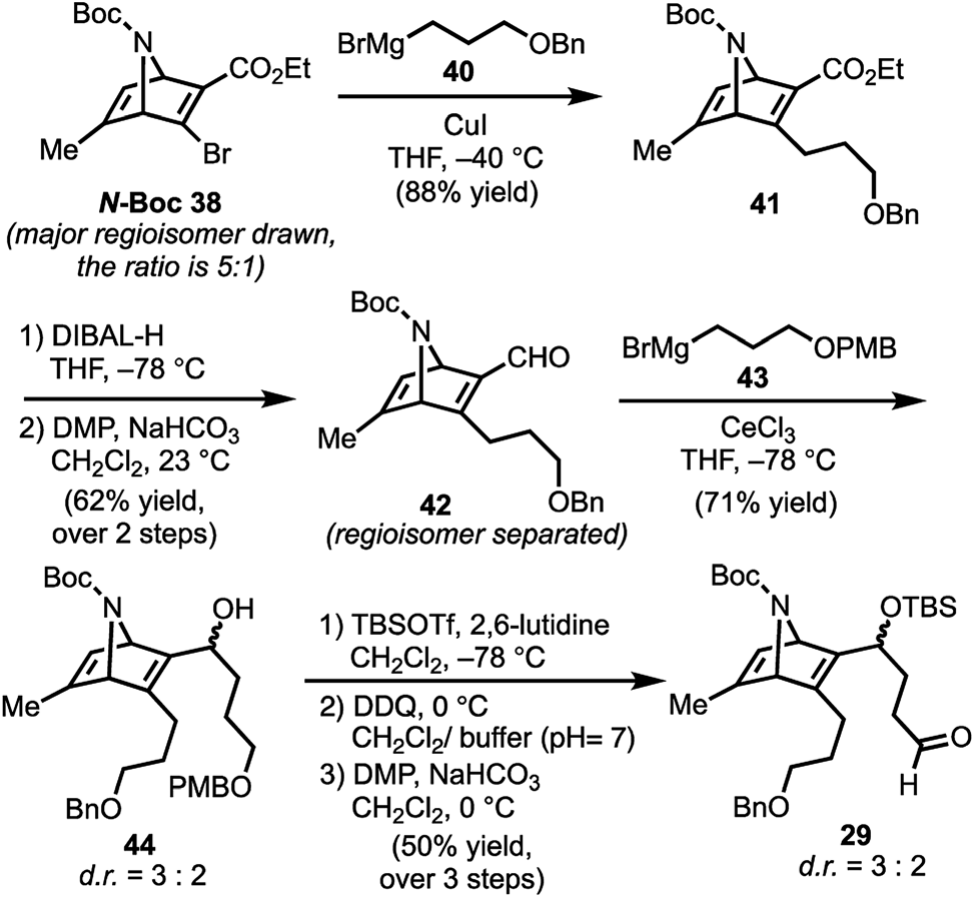
Preparation of key intermediate **29**; DIBAL-H = diisobutylaluminum hydride, TBSOTf = *tert*-butyldimethylsilyl trifluoromethanesulfonate; DDQ = 2,3-dichloro-5,6-dicyano-1,4-benzoquinone.

With **N-Boc-38** in hand, we moved to prepare key intermediate **29** (Scheme 5). The regiomeric mixture containing **N-Boc-38** was subjected to an organocopper coupling^25^ with **40** to afford **41** in 88% yield. Ester **41** was then converted to aldehyde **42** by DIBAL-H reduction and subsequent Dess–Martin oxidation of the resulting alcohol. At this point, chromatography allowed removal of the unwanted regiomer and isolation of **42**. Next, aldehyde **42** was subjected to Grignard reagent **43** in the presence of CeCl_3_, which suppresses 1,4-addition and facilitates production of the desired alcohol **44** (71% yield).^26^ Protection of the secondary alcohol in **44** with TBSOTf, followed by deprotection of the PMB-ether (DDQ) and Dess–Martin oxidation of the resulting alcohol delivers aldehyde **29**.

**Scheme 6:**
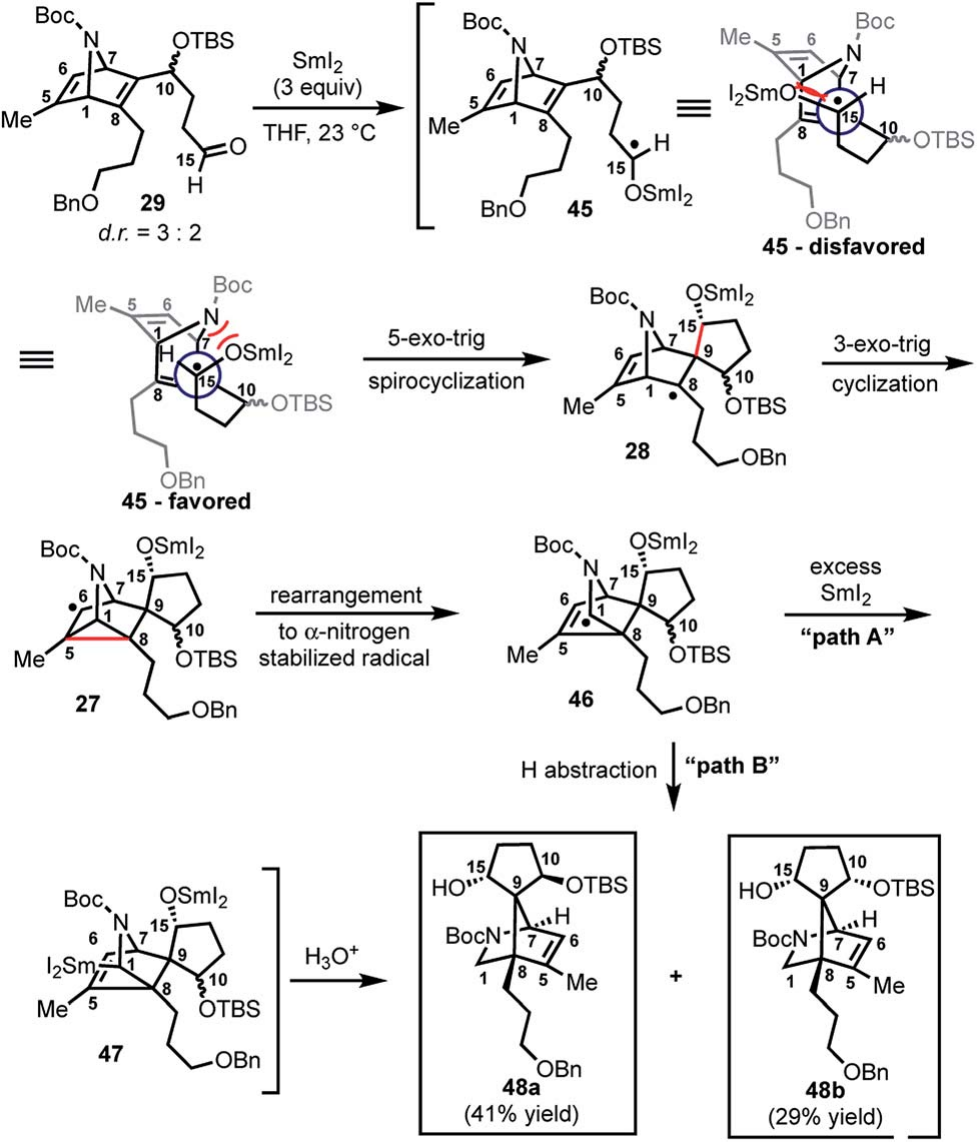
SmI_2_-mediated spirocyclization/rearrangement cascade to construct key bicyclic framework **25**.

**Scheme 7:**
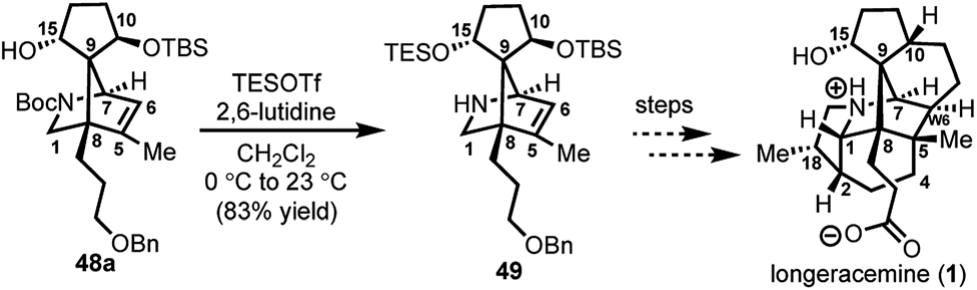
C15 hydroxyl protection of **48a** and concomitant Boc removal to afford **49**; TESOTf = triethylsilyl trifluoromethanesulfonate.

With **29** in hand, we were able to investigate the key SmI_2_-mediated spirocyclization/rearrangement cascade (Scheme 6). In the event, treatment of **29** with three molar equivalents of SmI_2_ in THF at 23 °C for 1 h furnished desired 2-azabicyclo[2.2.1]heptenes **48a** and **48b** in good yield with excellent site selectivity, and in a stereoselective fashion. We were delighted to find that reaction proceeds under these very mild conditions, and exclusively affords the C-15 hydroxyl stereochemistry necessary to access **1** as evident by ^1^H NOESY analysis; a strong NOE correlation is observed between the C15 hydrogen and the equatorial C1 hydrogen in both **48a** and **48b**.^27^ Mechanistically, we believe that ketyl radical **45** is generated *via* reaction of SmI_2_ with aldehyde **29** (Scheme 6). Subsequent 5-*exo*-trig spirocyclization of **45** furnishes tertiary radical **28** wherein the newly formed stereogenic center derives from the ketyl radical (**45**) engaging the olefin from the least hindered face with the C(15) hydrogen over the ring system thereby avoiding steric repulsion between the bicycle and the OSmI_2_ moiety (*cf.*, **45-disfavored** and **45-favored**). Finally, the intermediate tertiary radical (**28**) undergoes a 3-*exo*-trig cyclization to radical **27**, which then rapidly fragments^7*b*^ to afford the stabilized α-nitrogen radical **46**. Termination of the sequence can occur through two possible pathways, which efficiently delivers **48a** and **48b** as a pair of inconsequential epimers (3 : 2) at C10 in a combined isolated yield of 70%. One pathway involves the further reduction of **46** in the presence of excess SmI_2_,^16^ to dipole-stabilized Sm-anion **47**^28^ followed by protonation (“path A”; Scheme 6). A second, more direct pathway involves hydrogen atom abstraction by **46** (“path B”; Scheme 6). Experimentally, quenching of the terminal reactive species was attempted with D_2_O and CH_3_OD at −78 °C to effect deuterium exchange, however, no deuterium incorporation was ever observed and **48a** and **48b** were cleanly afforded; thus, H-atom abstraction from the THF solvent (path B; Scheme 6) cannot be ruled out at this time. Additionally, when the sequence is conducted with 2 molar equivalents of SmI_2_*versus* 3 molar equivalents, **48a** and **48b** are produced a significantly reduced yield of 42%, lending support for “path A” over “path B”, which would not require an excess of SmI_2_. Regardless, of the details of the termination sequence, the SmI_2_-mediated cascade stereoselectively forges two adjacent all-carbon quaternary centers and sets the correct stereochemistry at the C-15 hydroxyl necessary to access **1**. Protection of the C15 secondary alcohol as a triethylsilyl (TES) ether can be accomplished using triethylsilyl trifluoromethanesulfonate and 2,6-lutidine; concomitant removal of the Boc group, which conveniently reveals the secondary amine, as demonstrated with **48a** → **49**, and sets the stage for further advancement of **49** to **1** (Scheme 7).^27^

## Conclusions

In conclusion, we have accessed the 2-azabicyclo[2.2.1]heptane framework of longeracemine (**1**) *via* a novel SmI_2_ spirocyclization/rearrangement cascade. The reaction proceeds under mild conditions, in good yield, with excellent site selectivity, and in a stereoselective fashion. Our efforts to implement this rearrangement in a complex setting revealed that success is dependent on the stability of the radical intermediate (*e.g.*, **17–19**, and **28**), which can be estimated by considering the BDE of corresponding C–**H** bond. Investigation of the biological activity of the intermediates produced in this synthetic sequence along with efforts to complete their advancement to longeracemine (**1**) are currently underway and will be reported in due course.

## Conflicts of interest

There are no conflicts to declare.

## Supplementary Material

SC-011-D0SC03422C-s001
