# Supplementary material for: Synthetic studies toward longeracemine: a SmI2-mediated spirocyclization and rearrangement cascade to construct the 2-azabicyclo[2.2.1]heptane framework
Source: Chem Sci. 2020 Jul 30;11(35):9488–93. doi: 10.1039/d0sc03422c (PMC8162136; doi:10.1039/d0sc03422c)
Supplement: SC-011-D0SC03422C-s001 [file SC-011-D0SC03422C-s001.pdf]

Electronic Supplementary Material (ESI) for Chemical Science.
This journal is © The Royal Society of Chemistry 2020

Supplementary Information

Synthetic Studies Toward Longeracemine: A SmI_2-mediated Spirocyclization and Rearrangement Cascade to Construct the 2-Azabicyclo[2.2.1]heptane Framework

Keita Komine, Kyle M. Lambert, Quentin R. Savage, Joshua B. Cox, and John L. Wood*

Department of Chemistry and Biochemistry, Baylor University
One Bear Place 97348, Waco, TX 76798 (USA)

E-mail: john_l_wood@baylor.edu

Table of Contents

I. GENERAL INFORMATION 5

II. EXPERIMENTAL PROCEDURES 6

Preparation of Ester 12 6
Preparation of Primary Alcohol 16 7
Monitoring of Retro Diels-Alder Reaction of 16 8
Preparation of Propargyl Alcohol SI-5 9
Preparation of Ketone 31 10
Preparation of N-Benzyl Pyrrole 35 11
Preparation of N-Tosyl Bicyclic Esters N-Ts-38 and N-Ts-39 12
Preparation of N-Boc Bicyclic Esters N-Boc-38 and N-Boc-39 13
Preparation of Grignard Reagent 40 14
Preparation of Benzyl Ethers 41 and SI-8 14
Preparation of Alcohols SI-9 and SI-10 15
Preparation of Aldehyde 42 16
Preparation of Alkyl Bromide SI-12 17
Preparation of Grignard Reagent 43 18
Preparation of Alcohol 44 18
Preparation of TBS Ether SI-13 19
Preparation of Primary Alcohol SI-14 20
Preparation of Aldehyde 29 21
Preparation of Spirocycles 48a and 48b 22
Preparation of Secondary Amine 49 23

III. NMR SPECTRA 24

Figure S1. ^{1}H NMR (400 MHz, DMSO-d_6, 85 °C) Ester 12 25
Figure S2. ^{1}H NMR (400 MHz, DMSO-d_6, 85 °C) Ester 12 (-1 – 5 ppm inset) 26
Figure S3. ^{1}H NMR (400 MHz, DMSO-d_6, 85 °C) Ester 12 (5 – 10 ppm inset) 27
Figure S4. ^{13}C NMR (101 MHz, DMSO-d_6, 85 °C) Ester 12 28
Figure S5. ^{1}H NMR (600 MHz, DMSO-d_6, 27 °C) Primary Alcohol 16 29
Figure S6. ^{1}H NMR (600 MHz, DMSO-d_6, 27 °C) Primary Alcohol 16 (-1 – 5 ppm inset) 30
Figure S7. ^{1}H NMR (600 MHz, DMSO-d_6, 27 °C) Primary Alcohol 16 (5 – 10 ppm inset) 31
Figure S8. ^{13}C NMR (151 MHz, DMSO-d_6, 27 °C) Primary Alcohol 16 32
Figure S9. COSY (DMSO-d_6, 27 °C) Primary Alcohol 16 33
Figure S10. HSQC (DMSO-d_6, 27 °C) Primary Alcohol 16 34
Figure S11. HMBC (DMSO-d_6, 27 °C) Primary Alcohol 16 35
Figure S12. NOESY (DMSO-d_6, 27 °C) Primary Alcohol 16 36
Figure S13. ^{1}H NMR (400 MHz) Monitoring of Retro Diels-Alder Reaction (DMSO-d_6, 16 $\rightarrow$ SI-2 and SI-3) 37
Figure S14. ^{1}H NMR (400 MHz, DMSO-d_6, 27 °C) Primary Alcohol SI-3 38
Figure S15. ^{1}H NMR (400 MHz, DMSO-d_6, 27 °C) Primary Alcohol SI-3 (-1 – 5 ppm inset) 39
Figure S16. ^{1}H NMR (400 MHz, DMSO-d_6, 27 °C) Primary Alcohol SI-3 (5 – 10 ppm inset) 40
Figure S17. ^{13}C NMR (101 MHz, DMSO-d_6, 27 °C) Primary Alcohol SI-3 41
Figure S18. ^{1}H NMR (600 MHz, chloroform-d) Propargyl Alcohol SI-5 42
Figure S19. ^{1}H NMR (600 MHz, chloroform-d) Propargyl Alcohol SI-5 (-1 – 5 ppm inset) 43
Figure S20. ^{1}H NMR (600 MHz, chloroform-d) Propargyl Alcohol SI-5 (5 – 10 ppm inset) 44
Figure S21. ^{13}C NMR (151 MHz, chloroform-d) Propargyl Alcohol SI-5 45

Figure S22. ^{1}H NMR (400 MHz, chloroform-d) Ketone 31 46
Figure S23. ^{1}H NMR (400 MHz, chloroform-d) Ketone 31 (-1 – 5 ppm inset) 47
Figure S24. ^{1}H NMR (400 MHz, chloroform-d) Ketone 31 (5 – 10 ppm inset) 48
Figure S25. ^{13}C NMR (101 MHz, chloroform-d) Ketone 31 49
Figure S26. ^{1}H NMR (400 MHz, chloroform-d) N-Benzyl Pyrrole 35 50
Figure S27. ^{1}H NMR (400 MHz, chloroform-d) N-Benzyl Pyrrole 35 (-1 – 5.5 ppm inset) 51
Figure S28. ^{1}H NMR (400 MHz, chloroform-d) N-Benzyl Pyrrole 35 (5.5 – 10 ppm inset) 52
Figure S29. ^{13}C NMR (101 MHz, chloroform-d) N-Benzyl Pyrrole 35 53
Figure S30. ^{1}H NMR (400 MHz, chloroform-d) N-Tosyl Bicyclic Esters N-Ts-38 and N-Ts-39 54
Figure S31. ^{1}H NMR (400 MHz, chloroform-d) N-Tosyl Bicyclic Esters N-Ts-38 and N-Ts-39 (-1 – 4.5 ppm inset) 55
Figure S32. ^{1}H NMR (400 MHz, chloroform-d) N-Tosyl Bicyclic Esters N-Ts-38 and N-Ts-39 (4.5 – 10 ppm inset) 56
Figure S33. ^{13}C NMR (101 MHz, chloroform-d) N-Tosyl Bicyclic Esters N-Ts-38 and N-Ts-39 57
Figure S34. ^{1}H NMR (400 MHz, DMSO-d_6, 85 °C) N-Boc Bicyclic Esters N-Boc-38 and N-Boc-39 58
Figure S35. ^{1}H NMR (400 MHz, DMSO-d_6, 85 °C) N-Boc Bicyclic Esters N-Boc-38 and N-Boc-39 (-1 – 4.5 ppm inset) 59
Figure S36. ^{1}H NMR (400 MHz, DMSO-d_6, 85 °C) N-Boc Bicyclic Esters N-Boc-38 and N-Boc-39 (4.5 – 10 ppm inset) 60
Figure S37. ^{13}C NMR (101 MHz, DMSO-d_6, 85 °C) N-Boc Bicyclic Esters N-Boc-38 and N-Boc-39 61
Figure S38. ^{1}H NMR (400 MHz, DMSO-d_6, 85 °C) Benzyl Ethers 41 and SI-8 62
Figure S39. ^{1}H NMR (400 MHz, DMSO-d_6, 85 °C) Benzyl Ethers 41 and SI-8 (-1 – 4.7 ppm inset) 63
Figure S40. ^{1}H NMR (400 MHz, DMSO-d_6, 85 °C) Benzyl Ethers 41 and SI-8 (4.7 – 10 ppm inset) 64
Figure S41. ^{13}C NMR (101 MHz, DMSO-d_6, 85 °C) Benzyl Ethers 41 and SI-8 65
Figure S42. NOESY (DMSO-d_6, 27 °C) Benzyl Ethers 41 and SI-8 66
Figure S43. ^{1}H NMR (400 MHz, DMSO-d_6, 85 °C) Alcohol SI-9 and SI-10 67
Figure S44. ^{1}H NMR (400 MHz, DMSO-d_6, 85 °C) Alcohol SI-9 and SI-10 (-1 – 5 ppm inset) 68
Figure S45. ^{1}H NMR (400 MHz, DMSO-d_6, 85 °C) Alcohol SI-9 and SI-10 (5 – 10 ppm inset) 69
Figure S46. ^{13}C NMR (101 MHz, DMSO-d_6, 85 °C) Alcohol SI-9 and SI-10 70
Figure S47. ^{1}H NMR (400 MHz, DMSO-d_6, 85 °C) Aldehyde 42 71
Figure S48. ^{1}H NMR (400 MHz, DMSO-d_6, 85 °C) Aldehyde 42 (-1 – 4.7 ppm inset) 72
Figure S49. ^{1}H NMR (400 MHz, DMSO-d_6, 85 °C) Aldehyde 42 (4.7 – 11 ppm inset) 73
Figure S50. ^{13}C NMR (101 MHz, DMSO-d_6, 85 °C) Aldehyde 42 74
Figure S51. ^{1}H NMR (400 MHz, chloroform-d) alkyl bromide SI-12 75
Figure S52. ^{1}H NMR (400 MHz, chloroform-d) alkyl bromide SI-12 (-1 – 5 ppm inset) 76
Figure S53. ^{1}H NMR (400 MHz, chloroform-d) alkyl bromide SI-12 (5 – 10 ppm inset) 77
Figure S54. ^{13}C NMR (101 MHz, chloroform-d) alkyl bromide SI-12 78
Figure S55. ^{1}H NMR (400 MHz, DMSO-d_6, 85 °C) PMB Ether 44 79
Figure S56. ^{1}H NMR (400 MHz, DMSO-d_6, 85 °C) PMB Ether 44 (-1 – 5 ppm inset) 80
Figure S57. ^{1}H NMR (400 MHz, DMSO-d_6, 85 °C) PMB Ether 44 (5 – 10 ppm inset) 81
Figure S58. ^{13}C NMR (101 MHz, DMSO-d_6, 85 °C) PMB Ether 44 82
Figure S59. ^{1}H NMR (400 MHz, DMSO-d_6, 85 °C) TBS Ether SI-13 83
Figure S60. ^{1}H NMR (400 MHz, DMSO-d_6, 85 °C) TBS Ether SI-13 (-1 – 4 ppm inset) 84
Figure S61. ^{1}H NMR (400 MHz, DMSO-d_6, 85 °C) TBS Ether SI-13 (4 – 10 ppm inset) 85
Figure S62. ^{13}C NMR (101 MHz, DMSO-d_6, 85 °C) TBS Ether SI-13 86
Figure S63. ^{1}H NMR (400 MHz, DMSO-d_6, 85 °C) Primary Alcohol SI-14 87
Figure S64. ^{1}H NMR (400 MHz, DMSO-d_6, 85 °C) Primary Alcohol SI-14 (-1 – 4 ppm inset) 88
Figure S65. ^{1}H NMR (400 MHz, DMSO-d_6, 85 °C) Primary Alcohol SI-14 (4 – 10 ppm inset) 89

Figure S66. ^{13}C NMR (101 MHz, DMSO-d_6, 85 °C) Primary Alcohol SI-14..90
Figure S67. ^{1}H NMR (400 MHz, DMSO-d_6, 85 °C) Aldehyde 29 91
Figure S68. ^{1}H NMR (400 MHz, DMSO-d_6, 85 °C) Aldehyde 29 (-1 – 4 ppm inset) 92
Figure S69. ^{1}H NMR (400 MHz, DMSO-d_6, 85 °C) Aldehyde 29 (4 – 11 ppm inset)..93
Figure S70. ^{13}C NMR (101 MHz, DMSO-d_6, 85 °C) Aldehyde 29..94
Figure S71. ^{1}H NMR (400 MHz, DMSO-d_6, 85 °C) Spirocycle 48a..95
Figure S72. ^{1}H NMR (400 MHz, DMSO-d_6, 85 °C) Spirocycle 48a (-1 – 5 ppm inset) 96
Figure S73. ^{1}H NMR (400 MHz, DMSO-d_6, 85 °C) Spirocycle 48a (5 – 10 ppm inset)..97
Figure S74. ^{13}C NMR (101 MHz, DMSO-d_6, 85 °C) Spirocycle 48a..98
Figure S75. COSY (DMSO-d_6, 27 °C) Spirocycle 48a..99
Figure S76. HSQC (DMSO-d_6, 27 °C) Spirocycle 48a 100
Figure S77. HMBC (DMSO-d_6, 27 °C) Spirocycle 48a..101
Figure S78. NOESY (DMSO-d_6, 27 °C) Spirocycle 48a..102
Figure S79. ^{1}H NMR (400 MHz, DMSO-d_6, 85 °C) Spirocycle 48b 103
Figure S80. ^{1}H NMR (400 MHz, DMSO-d_6, 85 °C) Spirocycle 48b (-1 – 4 ppm inset) 104
Figure S81. ^{1}H NMR (400 MHz, DMSO-d_6, 85 °C) Spirocycle 48b (4 – 10 ppm inset)..105
Figure S82. ^{13}C NMR (101 MHz, DMSO-d_6, 85 °C) Spirocycle 48b..106
Figure S83. COSY (DMSO-d_6, 27 °C) Spirocycle 48b..107
Figure S84. HSQC (DMSO-d_6, 27 °C) Spirocycle 48b 108
Figure S85. HMBC (DMSO-d_6, 27 °C) Spirocycle 48b 109
Figure S86. NOESY (DMSO-d_6, 27 °C) Spirocycle 48b 110
Figure S87. ^{1}H NMR (400 MHz, acetonitrile-d_3) Secondary Amine 49..111
Figure S88. ^{1}H NMR (400 MHz, acetonitrile-d_3) Secondary Amine 49 (-1 – 5 ppm inset)..112
Figure S89. ^{1}H NMR (400 MHz, acetonitrile-d_3) Secondary Amine 49 (5 – 10 ppm inset) 113
Figure S90. ^{13}C NMR (101 MHz, acetonitrile-d_3) Secondary Amine 49 114
Figure S91. COSY (acetonitrile-d_3) Secondary Amine 49 115
Figure S92. HSQC (acetonitrile-d_3) Secondary Amine 49..116
Figure S93. HMBC (acetonitrile-d_3) Secondary Amine 49..117
Figure S94. NOESY (acetonitrile-d_3) Secondary Amine 49 118

I. GENERAL INFORMATION

Unless otherwise stated, all reactions were performed in flame-dried glassware under a nitrogen (N2) atmosphere, and reagents were used as received from the manufacturers. The reactions were monitored and analytical samples purified by normal phase thin-layer chromatography (TLC) using MilliporeSigma glass-backed 60 Å plates (indicator F-254, 250 µM) or by using Sigma Aldrich glass-backed 60 Å reverse phase C-18 fully end-capped plates (fluorescent indicator, 250 µM). Tetrahydrofuran, diethyl ether, benzene, dichloromethane, acetonitrile, dimethylformamide, and toluene were dried using a solvent purification system manufactured by SG Water, USA LLC. Diisopropylamine and *N,N*-diisopropylamine were dried over CaH_2 and freshly distilled prior to use. Dry air was obtained by slow passage through a 12-inch column of $CaCl_2$. Reactions involving organometallic reagents were conducted in flame-dried glassware under an argon atmosphere using standard techniques for handling air sensitive reagents,[1] solvents were deoxygenated by bubbling dry argon gas through the neat liquid for 10 min before use, and *n*-butyllithium was titrated immediately prior to use utilizing the method of Watson and Eastham.[2] Manual flash chromatography was performed using the indicated solvent systems with Silicycle SiliaFlash® P60 (230–400 mesh) silica gel as the stationary phase. Automated flash chromatography was performed on a Teledyne RF+UV-Vis MS Comp MPLC using the indicated solvent systems, and Teledyne RediSep® Rf normal phase disposable silica gel columns of the indicated size at the indicated flow rate.

^{1}H and ^{13}C NMR spectra were recorded on a Bruker AvanceTM III 300 MHz, Bruker AscendTM 400 MHz or Bruker AscendTM 600 MHz spectrometer fitted with autosamplers. Chemical shifts (δ) are reported in parts per million (ppm) relative to the residual solvent resonance and coupling constants (*J*) are reported in hertz (Hz). NMR peak pattern abbreviations are as follows: s = singlet, bs = broad singlet, d = doublet, dd = doublet of doublets, ddd = doublet of doublet of doublets, dddd = doublet of doublet of doublet of doublets, dt = doublet of triplets, ddt = doublet of doublet of triplets, dtd = doublet of triplet of doublets, dq = doublet of quartets, t = triplet, td = triplet of doublets, tdd = triplet of doublet of doublets, q = quartet, qd = quartet of doublets, qdd = quartet of doublet of doublets, qt = quartet of triplets, m = multiplet. Proton spectra recorded in deuterated chloroform are referenced to the residual ^{1}H signal of $CHCl_3$ at δ = 7.26 and are reported relative to TMS at δ = 0.00; carbon spectra are referenced to the central ^{13}C signal for $CDCl_3$ at δ = 77.16 and are reported relative to TMS at δ = 0.00. Proton spectra recorded in deuterated dimethylsulfoxide are referenced to the residual ^{1}H signal of DMSO-d_5 at δ = 2.50 and are reported relative to TMS at δ = 0.00; carbon spectra are referenced to the central ^{13}C signal for DMSO-d_6 at δ = 39.52 and are reported relative to TMS at δ = 0.00. Proton spectra recorded in deuterated acetonitrile are referenced to the residual ^{1}H signal for acetonitrile-d_2 at δ = 1.94 and are reported relative to TMS at δ = 0.00; carbon spectra are referenced to the central ^{13}C signals for acetonitrile-d_3 at δ = 1.32 or 118.26, and are reported relative to TMS at δ = 0.00. Variable Temperature NMR was performed at the given temperature to allow for the coalescence of rotameric peaks. ^{13}C NMR peaks reported for inseparable mixtures denote peaks that are differentiable in the observed spectrum. Two-dimensional NMR spectra (HMBC, HSQC, ^{1}H-^{1}H COSY, and ^{1}H-^{1}H NOSEY) when necessary, and all recorded spectra are provided below. Infrared (IR) spectra were recorded on Bruker Platinum-ATR IR spectrometer using a diamond window. High Resolution mass spectra (HRMS) were obtained in the Baylor University Mass Spectrometry Center on a Thermo Scientific LTQ Orbitrap Discovery spectrometer using +ESI or –ESI and reported for the molecular ion ($[M+H]^+$ and $[M+Na]^+$ or $[M-H]^-$ respectively).

1. (a) T. L. Rathman, J. A. Schwindeman, *Org. Process Res. Dev.* **2014**, *18,* 1192. (b) D. F. Shriver, M. A. Drezdon, in *The Manipulation of Air-Sensitive Compounds*, 2nd ed., Wiley, New York, **1986**.
2. S. C. Watson, J. F. Eastham, *J. Organomet. Chem.* **1967**, *9*, 165.

II. EXPERIMENTAL PROCEDURES

Preparation of Ester ***12***

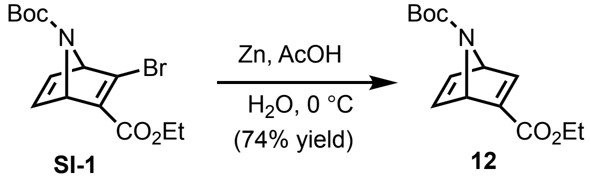

Ester **12** was prepared according to the procedure reported by Hodgson and co-workers.[3] To a round-bottomed flask containing a magnetic stir bar and powdered zinc dust (409 mg, 6.25 mmol, 7.3 equiv) was added a 10% aqueous solution of hydrochloric acid (2.5 mL, 6.8 mmol, 8.0 equiv) dropwise to and the reaction mixture stirred for five minutes. At which point, the liquid was decanted and the zinc was washed with acetone (2 x 3 mL) and Et_2O (3 mL). A suspension of AgOAc (14 mg, 0.0856 mmol, 0.1 equiv) in AcOH (1 mL) was then added with stirring to the digested zinc. After one minute, the supernatant was decanted and the black Zn/Ag couple was washed successively with AcOH (1.5 mL), Et_2O (4 x 3 mL) and MeOH (3 mL). The moist Zn/Ag was then added to a stirred solution of known bromodiene **SI-1**[4] (283 mg, 0.856 mmol, 1 equiv) in MeOH (3 mL), and the reaction mixture was stirred for 3 h. The reaction mixture was then filtered through a pad of Celite, extracted with EtOAc (3 x 10 mL), and evaporated *in vacuo*. The residue was purified by silica gel chromatography (40 g SiO_2, hexanes/EtOAc = 10:1) to afford ester **12** (168 mg, 74% yield) as a yellow oil. **^{1}H NMR** (400 MHz, DMSO-d_6, 85 °C) δ 7.78 (d, J = 2.5 Hz, 1H), 7.18 (dd, J = 5.4, 2.5 Hz, 1H), 7.06 (dd, J = 5.4, 2.5 Hz, 1H), 5.26 (q, J = 2.5 Hz, 1H), 5.21 (bs, 1H), 4.18 (q, J = 7.1 Hz, 2H), 1.36 (s, 9H), 1.24 (t, J = 7.1 Hz, 3H); **^{13}C NMR** (101 MHz, DMSO-d_6, 85 °C) δ 162.2, 154.0, 153.2, 149.4, 143.3, 142.6, 79.6, 67.1, 65.8, 59.8, 27.5, 13.6. **FTIR** (thin film): 2981, 1710, 1368, 1338, 1318, 1269, 1167, 1081 cm^{-1}; **HRMS** (ESI) m/z $[M+Na]^+$ calcd for $C_{14}H_{19}NNaO_4$: 288.1206, found: 288.1206.

NOTE**:** NMR spectra were taken at 85 °C due to the rotameric nature of the Boc amide in **12**.

3. D. M. Hodgson, M. L. Jones, C. R. Maxwell, A. R. Cowley, A. L. Thompson, O. Ichihara, I. R. Matthews, *Tetrahedron* **2009**, *65*, 7825–7836.
4. C. Zhang, M. L. Trudell, *J. Org. Chem.* **1996**, *61*, 7189–7191.

*Preparation of Primary Alcohol **16***

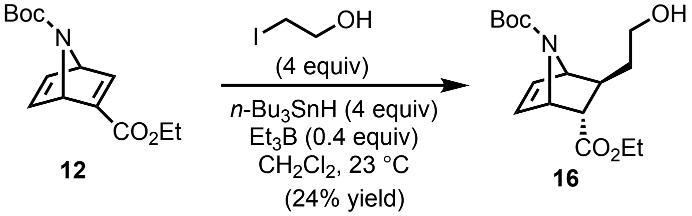

Primary alcohol **16** was prepared through slight modification of the procedure reported by Hodgson and co-workers for the related sulfone **13**.[5] Ester **12** (154 mg, 0.581 mmol, 1.0 equiv) was dissolved in CH_2Cl_2 (29 mL) and argon gas was bubbled through the solution for 2 h. At which point, 2-iodoethanol (0.18 mL, 2.32 mmol, 4.0 equiv) and Et_3B (1.0 M in hexane, 0.23 mL, 0.232 mmol, 0.4 equiv) were added via syringe to the degassed solution. To the reaction mixture was added Bu_3SnH (0.63 mL, 2.32 mmol, 4 equiv) via syringe pump over a period of 1 h, and dry air was injected into the headspace of the reaction mixture, in parallel, at 3 min intervals. After the additions were complete, the reaction mixture was allowed to stir for 3 h, concentrated *in vacuo*, and purified by silica gel chromatography (40 g SiO_2, hexanes/EtOAc = 3:1 to 1:1) to afford primary alcohol **16** (44.4 mg, 24% yield) as a colorless oil: **^{1}H NMR** (600 MHz, DMSO-d_6, 27 °C, reported as 1:1 mixture of rotamers) δ 6.56 – 6.45 (m, 1H), 6.28 – 6.20 (m, 1H), 4.68 (m, 1H), 4.43 (bs, 1H), 4.38 (bs, 1H), 4.09 – 3.89 (m, 2H), 3.49 (apparent q, J = 6.2 Hz, 2H), 2.80 – 2.64 (m, 1H), 1.85–1.75 (m, 1H), 1.68 – 1.50 (m, 2H), 1.35 (s, 9H), 1.16 (t, J = 7.1 Hz, 3H); **^{13}C NMR** (151 MHz, DMSO-d_6, 27 °C, reported as 1:1 mixture of rotamers) δ 171.4 (2C), 154.7, 154.2, 138.6, 137.3, 133.7, 132.8, 79.4, 79.3, 64.3, 64.0, 61.6, 60.7, 60.1 (2C), 59.5 (2C), 50.2, 49.4, 41.1, 40.0, 37.1, 36.9, 27.8 (2C), 14.0 (2C); **FTIR** (thin film): 3463, 2977, 1702, 1366, 1277, 1248, 1162, 1095, 1034, 872, 703 cm^{-1}; **HRMS** (ESI) m/z $[M+Na]^+$ calcd for $C_{16}H_{25}NNaO_5$: 334.1625, found: 334.1628.

NOTE**:** Variable temperature NMR analysis was attempted to achieve coalescence of the rotameric peaks, however, a retro-Diels-Alder reaction of **16** occurred under the VT-NMR conditions to afford **SI-2** and **SI-3** (DMSO-d_6, 85 °C, 1.5 h). See Figure S13 for the monitoring of the retro-Diels-Alder reaction over time.

5. D. M. Hodgson, S. Hachisu, and M. D. Andrews, *Org. Lett.* **2005**, *7*, 815–817.

*Monitoring of Retro Diels-Alder Reaction of **16***

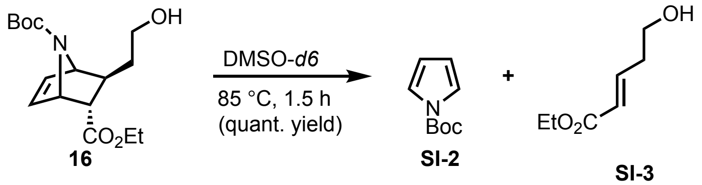

SI-2

^{1}H NMR (400 MHz, DMSO-d_6, 27 °C) δ 7.23 (t, J = 2.2 Hz, 2H), 6.25 (t, J = 2.2 Hz, 2H), 1.55 (s, 9H). The spectral data is fully in accord with the literature.[6]

SI-3

^{1}H NMR (400 MHz, DMSO-d_6, 27 °C) δ 6.90 (dt, J = 15.8, 7.1 Hz, 1H), 5.88 (dt, J = 15.7, 1.6 Hz, 1H), 4.66 (t, J = 5.2 Hz, 1H), 4.10 (q, J = 7.1 Hz, 2H), 3.51 (td, J = 6.3, 5.2 Hz, 2H), 2.33 (qd, J = 6.4, 1.6 Hz, 2H), 1.20 (t, J = 7.1 Hz, 3H); **^{13}C NMR** (101 MHz, DMSO-d_6, 27 °C) δ 165.6, 147.2, 122.1, 59.7, 59.4, 35.2, 14.2; **FTIR** (thin film): cm^{-1} 3430, 2930, 1699, 1654, 1266, 1161, 1038, 977; **HRMS** (ESI) *m/z* $[M+Na]^+$ calcd for $C_7H_{12}NaO_3$: 167.0684, found: 167.0710. The spectral data is fully in accord with the literature.[7]

6. T. Fukuda, Y. Nanjo, M. Fujimoto, K. Yoshida, Y. Natsui, F. Ishibashi, F. Okazaki, H. To, M. Iwao, *Bioorg. Med. Chem.* **2019**, *27*, 265–277.
7. K. Heckenbichler, A. Schweiger, L. A. Brandner, A. Binter, M. Toplak, P. Macheroux, K. Gruber, R. Breinbauer *Angew. Chem. Int. Ed.* **2018**, *57*, 7240–7244.

*Preparation of Propargyl Alcohol **SI-5***

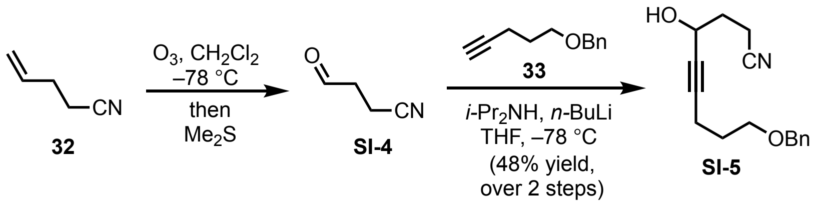

Aldehyde **SI-4** was prepared following the general procedure of Njardarson and co-workers.[8] A flask was charged with 4-pentenenitrile **32** (500 mg, 6.16 mmol, 1.0 equiv), CH_2Cl_2 (31 mL) and cooled to –78 °C in a dry ice/acetone bath. Ozone was bubbled through the –78 °C solution for about 30 minutes, until the reaction turned blue. At which point, nitrogen gas was bubbled through the solution to remove the ozone, then Me_2S (1.0 mL, 13.6 mmol, 2.2 equiv) was added. The reaction mixture was then gradually allowed to warm to room temperature (23 °C) and stirred for 8 h. The solution was concentrated *in vacuo* to afford **SI-4**,[8] which was used directly for the next step without further purification.

A solution of freshly distilled *N,N*-diisopropylamine (1.12 mL, 8.01 mmol, 1.3 equiv) in dry THF (40 mL) placed under argon, was cooled to 0 °C and *n*-BuLi (2.40 M in hexane, 3.2 mL, 7.70 mmol, 1.25 equiv) was added via syringe. The mixture was allowed to stir at 0 °C for 15 minutes, then cooled to –78 °C in a dry ice/acetone bath. At which point, freshly prepared acetylene **33**[9] (1.61 g, 9.25 mmol, 1.5 equiv) was added dropwise to the reaction mixture. After stirring at –78 °C for 1 h, a solution of crude **SI-4** (512 mg, 6.16 mmol, 1.0 equiv) in THF (5 mL) was cooled to –78 °C then added dropwise to the reaction mixture. The reaction mixture was then allowed to stir at –78 °C for 1.5 h, at which point, saturated aqueous NH_4Cl (25 mL) was added and the mixture was allowed to warm to 23 °C. The mixture was then extracted twice with EtOAc (2 x 25 mL), and the combined organic layers were washed with brine (25 mL), dried over Na_2SO_4, filtered, and concentrated *in vacuo*. The resulting residue was purified by silica gel chromatography (100 g SiO_2, hexanes/EtOAc= 5:1 to 3:1) to afford **SI-5** (769 mg, 48% yield, over 2 steps from **SI-5**) as colorless oil: **^{1}H NMR** (600 MHz, chloroform-*d*) δ 7.39 – 7.24 (m, 5H), 4.51 (s, 2H), 4.42 (bs, 1H), 3.54 (t, *J* = 6.1 Hz, 2H), 2.63 – 2.40 (m, 3H), 2.33 (td, *J* = 7.1, 1.9 Hz, 2H), 1.99 – 1.90 (m, 2H), 1.87 – 1.80 (m, 2H); **^{13}C NMR** (151 MHz, chloroform-*d*) δ 138.3, 128.4, 127.8, 127.7, 119.5, 86.2, 79.7, 73.0, 68.6, 60.5, 33.2, 28.6, 15.6, 13.0; **FTIR** (thin film): 3414, 2935, 2861, 2248, 1101, 1068, 737, 698 cm^{-1}; **HRMS** (ESI) *m/z* $[M+Na]^+$ calcd for $C_{16}H_{19}NNaO_2$: 280.1308, found: 280.1310.

8. Q. Yang, C. Draghici, J. T. Njardarson, F. Li, B. R. Smith, P. Das, *Org. Biomol. Chem.* **2014**, *12*, 330–344.
9. E. D. Slack, C. M. Gabriel, B. H. Lipshutz, *Angew. Chem. Int. Ed.* **2014**, *53*, 14051–14054.

Preparation of Ketone ***31***

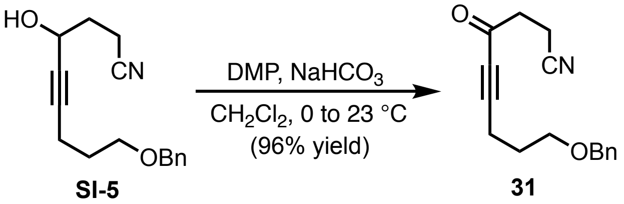

To a solution of **SI-5** (769 mg, 2.99 mmol, 1.0 equiv) in CH_2Cl_2 (15 mL) at 0 °C, was added $NaHCO_3$ (753 mg, 8.96 mmol, 3.0 equiv), and Dess-Martin periodinane (1.90 g, 4.48 mmol, 1.5 equiv). The reaction mixture was then allowed to warm to 23 °C and stirred for an additional 1 h. At which point, a solution of saturated, aqueous $NaHCO_3$ (10 mL) was added followed by a solution of 10% aqueous $Na_2S_2O_3$ (10 mL) and the mixture was extracted twice with EtOAc (2 x 25 mL). The combined organic layers were washed with brine (25 mL), dried over Na_2SO_4, filtered, and concentrated *in vacuo*. The resulting residue was purified by silica gel chromatography (7 g SiO_2, hexanes/EtOAc= 2:1) to afford **31** (732 mg, 96% yield) as a colorless oil: **^{1}H NMR** (400 MHz, chloroform-*d*) δ 7.40 – 7.25 (m, 5H), 4.52 (s, 2H), 3.56 (t, J = 5.9 Hz, 2H), 2.87 (t, J = 7.2 Hz, 2H), 2.60 (t, J = 7.2 Hz, 2H), 2.54 (t, J = 7.1 Hz, 2H), 1.94 – 1.83 (m, 2H);**^{13}C NMR** (101 MHz, chloroform-*d*) δ 182.6, 138.1, 128.3, 127.6, 118.4, 96.2, 79.9, 72.9, 68.1, 40.2, 27.7, 15.9, 11.4; **FTIR** (thin film): 2861, 2210, 1673, 1409, 1363, 1167, 1103, 738, 698 cm^{-1}; **HRMS** (ESI) *m/z* $[M+Na]^+$ calcd for $C_{16}H_{17}NNaO_2$ 278.1151, found: 278.1153.

*Preparation of N-Benzyl Pyrrole **35***

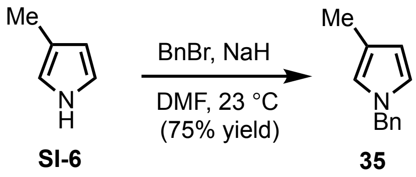

To a 0 °C solution of sodium hydride (60% in mineral oil, 37 mg, 0.925 mmol, 1.5 equiv) in DMF (1.8 mL) was added **SI-6** (50 mg, 0.616 mmol, 1.0 equiv). The reaction mixture was allowed to warm to 23 °C and stirred for an additional 30 minutes. At which point, the reaction mixture was cooled to 0°C and benzyl bromide (73 µL, 0.616 mmol, 1.0 equiv) was added. The reaction mixture was then allowed to warm to 23 °C and stirred for an additional 4 h. At which point, a solution of saturated, aqueous NH_4Cl (10 mL) was added and the mixture was extracted twice with hexanes (2 x 25 mL). The combined organic layers were washed with brine (25 mL), dried over Na_2SO_4, filtered, and concentrated *in vacuo*. The resulting residue was purified by silica gel chromatography (10 g SiO_2, hexanes/EtOAc= 100:1 to 50:1) to afford **35** (80 mg, 75% yield) as a colorless oil; **^{1}H NMR** (400 MHz, chloroform-*d*) δ 7.40 – 7.20 (m, 3H), 7.14 – 7.09 (m, 2H), 6.58 (d, *J* = 2.5 Hz, 1H), 6.44 (bs, 1H), 6.00 (d, *J* = 2.3 Hz, 1H), 4.99 (s, 2H), 2.10 (s, 3H); **^{13}C NMR** (101 MHz, chloroform-*d*) δ 138.5, 128.8, 127.7, 127.1, 121.1, 119.3, 119.2, 109.8, 53.3, 12.1; **FTIR** (thin film): 2921, 1497, 1453, 1329, 1158, 1069, 758, 717, 694 cm^{-1}; **HRMS** (ESI) *m/z* $[M+H]^+$ calcd for $C_{12}H_{14}N$: 172.1121, found: 172.1120.

*Preparation of N-Tosyl Bicyclic Esters **N-Ts-38** and **N-Ts-39***

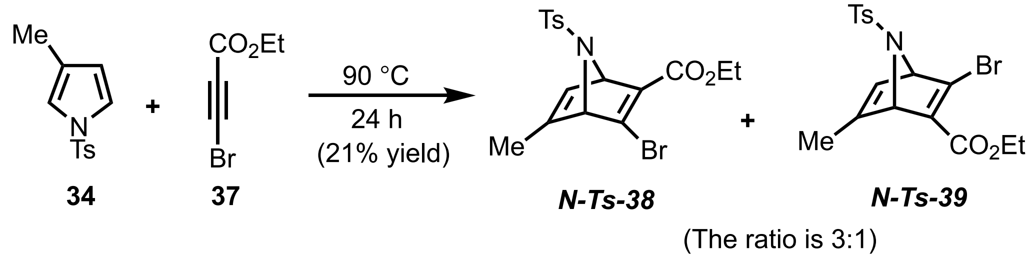

To a flame-dried, 1.5 dram vial containing pyrrole **34**[10] (30 mg, 0.128 mmol, 1.0 equiv) was added ethyl-3-bromopropiolate **37**[11] (42 mg, 0.255 mmol, 2.0 equiv). The reaction mixture was then heated to 90 °C and allowed to stir for 24 h. At which point, the reaction mixture was cooled to 23 °C, concentrated *in vacuo,* and purified by silica gel chromatography (3 g SiO_2, hexanes/EtOAc= 8:1 to 5:1) to afford an inseparable mixture of bicyclic esters ***N-Ts-38*** and ***N-Ts-39*** (11.1 mg, 21% yield, ***N-Ts-38*** : ***N-Ts-39*** = 3:1) as colorless oils: **^{1}H NMR** (400 MHz, chloroform-*d*, as a 3:1 mixture of ***N-Ts*-38** and ***N-Ts-39***) ***N-Ts-38*** δ 7.58 (d, *J* = 8.1 Hz, 2H), 7.25 (d, *J* = 7.2 Hz, 2H), 6.51 (s, 1 H), 5.34 (t, *J* = 2.6 Hz, 1H), 4.80 (d, *J* = 2.4 Hz, 1H), 4.19 – 3.98 (m, 2H), 2.40 (s, 3H), 1.93 (d, *J* = 1.9 Hz, 3H), 1.25 (td, *J* = 7.1, 1.8 Hz, 3H), ***N-Ts-39*** δ 7.58 (d, *J* = 8.1 Hz, 2H), 7.25 (d, *J* = 7.2 Hz, 2H), 6.47 (s, 0.3H), 5.11 (d, *J* = 2.4 Hz, 0.3H), 4.99 (t, *J* = 2.4 Hz, 0.3H), 4.19 – 3.98 (m, 2H), 2.40 (s, 3H), 1.89 (d, *J* = 1.9 Hz, 3H), 1.25 (td, *J* = 7.1, 1.8 Hz, 3H); **^{13}C NMR** (101 MHz, chloroform-*d*, as a 3:1 mixture of ***N-Ts-38*** and ***N-Ts-39***) δ 161.9, 161.7, 156.8, 154.0, 147.5, 145.0, 144.1, 143.6, 141.6, 135.8, 135.2, 132.5, 130.1, 128.5, 79.0, 77.4, 76.2, 72.8, 70.0, 61.0, 60.9, 21.7, 15.8, 15.7, 14.3; **FTIR** (thin film): 2981, 1697, 1597, 1346, 1309, 1159, 1091, 814, 702, 583 cm^{-1}; **HRMS** (ESI) *m/z* $[M+Na]^+$ calcd for $C_{17}H_{18}{}^{79}BrNNaO_4S$: 434.0032, found: 434.0035.

10. P. Folly, Ph.D. Dissertation, University of Fribourg, Fribourg, Switzerland, **2000**.
11. F. Beltran, I. Fabre, I. Ciofini, L. Miesch, *Org. Lett.* **2017**, *19*, 5042–5045.

Preparation of N-Boc Bicyclic Esters ***N-Boc-38*** *and* ***N-Boc-39***

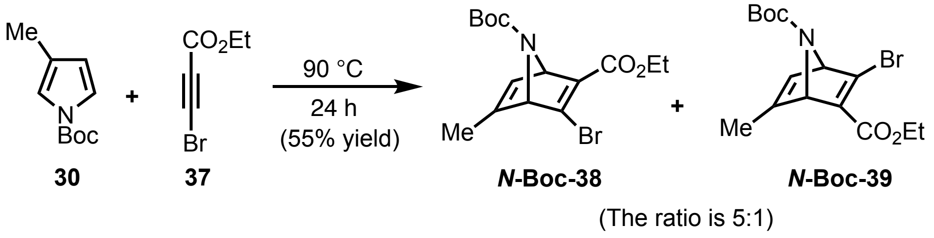

To a flame-dried, 1.5 dram vial containing pyrrole **30**[12] (100 mg, 0.552 mmol, 1.0 equiv) was added ethyl 3-bromopropiorate **37**[11] (180 mg, 1.10 mmol, 2.0 equiv). The reaction mixture was then heated to 90 °C and allowed to stir for 24 h. At which point, the reaction mixture was cooled to 23 °C, concentrated *in vacuo,* and purified by silica gel chromatography (10 g SiO_2, hexanes/EtOAc= 40:1 to 30:1) to afford an inseparable mixture of bicyclic esters ***N*-Boc-38** and ***N*-Boc-39** (108 mg, 55% yield, ***N*-Boc-38** : ***N*-Boc-39** = 5:1) as yellow oils: **^{1}H NMR** (400 MHz, DMSO-d_6, 85 °C, as a 5:1 mixture of ***N*-Boc-38** and ***N*-Boc-39**) ***N*-Boc-38**: δ 6.66 – 6.58 (m, 1H), 5.21 (t, *J* = 2.4 Hz, 1H), 4.89 (t, *J* = 2.4 Hz, 1H), 4.28 – 4.14 (m, 2H), 1.98 (d, *J* = 2.0 Hz, 3H), 1.37 (s, 9H), 1.26 (t, *J* = 7.2 Hz, 3H), ***N*-Boc-39**: δ 6.66 – 6.58 (m, 1H), 5.03 (d, *J* = 2.4 Hz, 1H), 5.01 (t, *J* = 2.4 Hz, 1H), 4.28 – 4.14 (m, 2H), 1.95 (d, *J* = 2.0 Hz, 3H), 1.37 (s, 9H), 1.27 (t, *J* = 7.2 Hz, 3H); **^{13}C NMR** (101 MHz, DMSO-d_6, 85 °C, as a 5:1 mixture of ***N*-Boc-38** and ***N*-Boc-39**) δ 161.4, 161.3, 154.5, 153.4, 152.1, 143.6, 143.5, 133.9, 133.9, 131.1, 80.3, 77.9, 77.8, 74.8, 71.8, 68.7, 60.0, 27.3, 27.3, 23.3, 14.7, 14.5, 13.5, 13.5; **FTIR** (thin film): 2977, 1711, 1596, 1303, 1254, 1204, 1158, 1080, 858, 789 cm^{-1}; **HRMS** (ESI) *m/z* $[M+Na]^+$ calcd for $C_{15}H_{20}{}^{79}BrNNaO_4$ 380.0468, found: 380.0469.

***NOTE*:** NMR spectra were taken at 85 °C due to the rotameric nature of the Boc amide in ***N*-Boc-38** and ***N*-Boc-39**.

12. T. Ikawa, A. Takagi, Y. Kurita, K. Saito, K. Azechi, M. Egi, K. Kakiguchi, Y. Kita, S. Akai, *Angew. Chem. Int. Ed.* **2010**, *49*, 5563–5566.

*Preparation of Grignard Reagent **40***

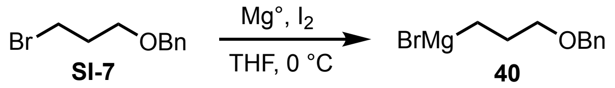

Fresh Grignard reagent **37** was prepared using the following procedure: To a flame-dried, 100 mL round-bottomed flask containing magnesium turnings (616 mg, 25.3 mmol, 1.2 equiv) cooled to 0 °C in an ice/water bath, was added a 0 °C solution of **SI-7**[13] (4.84 g, 21.1 mmol, 1.0 equiv) and iodine (107.1 mg, 0.422 mmol, 0.02 equiv) in THF (21 mL). The ice/water bath as then removed and the reaction mixture was allowed to warm to 23 °C. The suspension containing **40** was allowed to settle and used for the next step.

*Preparation of Benzyl Ethers **41** and **SI-8***

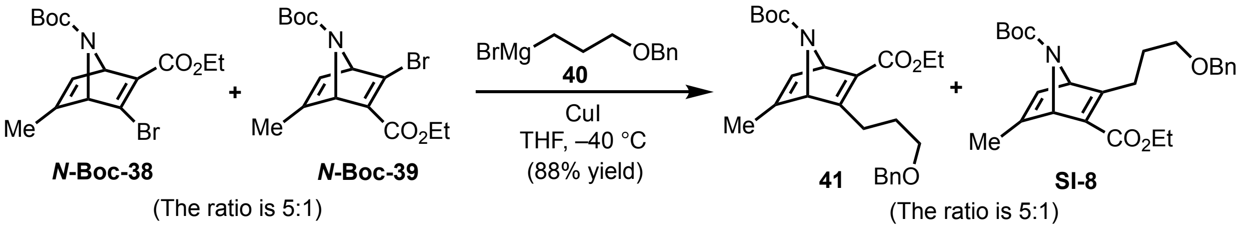

To a flame-dried flask under argon containing a solution of copper(I) iodide (2.0 g, 10.6 mmol, 1.2 equiv) in dry THF (130 mL) at –78 °C was added a cooled solution (–78 °C) of the freshly prepared Grignard reagent **40** (1.0 M in THF solution, 21 mL, 21.1 mmol, 2.4 equiv). The reaction mixture was allowed to stir at –78 °C for 20 minutes, then warmed to –35 °C and stirred for an additional 15 minutes. At which point a cooled solution (–35 °C) of a mixture of ***N*-Boc-38** and ***N*-Boc-39** (3.15g, 8.79 mmol, 1.0 equiv, ***N*-Boc-38**: ***N*-Boc-39** = 5:1) in THF (25 mL) was added. The reaction mixture was then allowed to stir at –35 °C for 1 h. At which point, a solution of saturated, aqueous NH_4Cl (50 mL) was added and the mixture was allowed to warm to 23 °C and extracted twice with Et_2O (2 x 100 mL). The combined organic layers were washed with brine (50 mL), dried over Na_2SO_4, filtered, and concentrated *in vacuo*. The resulting residue was purified by silica gel chromatography (80 g SiO_2, hexanes/EtOAc= 20:1 to 10:1) to afford an inseparable mixture of **41**and **SI-8** (3.30 g, 88% yield, **38** : **SI-8** = 5:1) as yellow oils: **^{1}H NMR** (400 MHz, DMSO-d_6, 85 °C, as a mixture of **38** : **SI-8** = 5:1) **41**: δ 7.40 – 7.22 (m, 5H), 6.55 (bs, 1H), 5.11 (s, 1H), 4.84 (d, *J* = 2.1 Hz, 1H), 4.47 (s, 2H), 4.22 – 4.05 (m, 2H), 3.52 – 3.42 (m, 2H), 2.84 – 2.76 (m, 1H), 2.74 – 2.66 (m, 1H), 1.92 (d, *J* = 2.0 Hz, 3H), 1.83 – 1.70 (m, 2H), 1.35 (s, 9H), 1.21 (t, *J* = 7.1 Hz, 3H), **SI-8**: δ 7.40 – 7.22 (m, 5H), 6.40 (bs, 1H), 4.98 (s, 1H), 4.92 (s, 1H), 4.47 (s, 2H), 4.22 – 4.05 (m, 2H), 3.52 – 3.42 (m, 2H), 2.84 – 2.76 (m, 1H), 2.64 – 2.57 (m, 1H), 1.91 (d, *J* = 2.0 Hz, 3H), 1.83 – 1.70 (m, 2H), 1.35 (s, 9H), 1.22 (t, *J* = 7.1 Hz, 3H); **^{13}C NMR** (101 MHz, DMSO-d_6, 85 °C, as a mixture of **41** : **SI-8** = 5:1) δ 163.3, 163.1, 153.5, 152.0, 139.5, 139.5, 138.3, 138.3, 134.7, 134.7, 131.6, 127.6, 126.9, 126.8, 126.8, 126.7, 79.3, 73.9, 73.9, 71.6, 71.5, 70.5, 68.8, 68.8, 67.1, 67.0, 59.2, 59.2, 27.4, 27.3, 26.0, 25.2, 14.6, 14.6, 13.6; **FTIR** (thin film): 2977, 1706, 1368, 1333, 1256, 1200, 1167, 1100, 1071 cm^{-1}; **HRMS** (ESI) *m/z* $[M+Na]^+$ calcd for $C_{25}H_{33}NNaO_5$: 450.2251, found: 450.2252.

***NOTE*:** NMR spectra were taken at 85 °C due to the rotameric nature of the Boc amide in **38** and **SI-8**.

13. K. J. Frankowski, J. E. Golden, Y. Zeng, Y. Lei, J. Aubé, *J. Am. Chem. Soc.* **2008**, *130*, 6018–6024.

*Preparation of Alcohols **SI-9** and **SI-10***

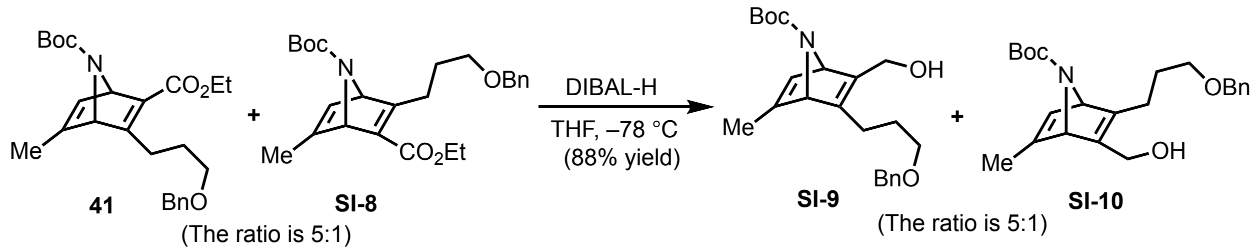

To a cooled solution (–78 °C) of a mixture of **41** and **SI-8** (1.40 g, 3.27 mmol, 1.0 equiv, **41**: **SI-8** = 5:1) in THF (17 mL), was added DIBAL-H (1.0 M hexane solution, 10 mL, 10 mmol, 3.0 equiv). The reaction mixture was allowed to stir at –78 °C for 1.5 h, at which point, an aqueous solution of saturated Rochelle salt (25 mL) was added and the mixture was allowed to warm to 23 °C and stirred for 14 h. The mixture was then extracted twice with EtOAc (2 x 50 mL). The combined organic layers were washed with brine (25 mL), dried over Na_2SO_4, filtered, and concentrated *in vacuo*. The resulting residue was purified by silica gel chromatography (60 g SiO_2, hexanes/EtOAc= 2:1 to 1:1) to afford an inseparable mixture of **SI-9** and **SI-10** (1.11 g, 88% yield, **SI-9** : **SI-10** = 5:1) as colorless oils: **^{1}H NMR** (400 MHz, DMSO-d_6, 85 °C, as a mixture of **SI-9** : **SI-10** = 5:1) **SI-9**: δ 7.36 – 7.25 (m, 5H), 6.42 (bs, 1H), 4.86 (s, 1H), 4.58 (d, J = 2.0 Hz, 1H), 4.45 (s, 2H), 4.11 (d, J = 13.6 Hz, 1H), 3.98 (d, J = 13.6 Hz, 1H), 3.44 – 3.37 (m, 2H), 2.36 – 2.20 (m, 3H), 1.89 (d, J = 2.0 Hz, 3H), 1.73 – 1.59 (m, 2H), 1.35 (s, 9H), **SI-10**: δ 7.36 – 7.25 (m, 5H), 6.34 (s, 1H), 4.74 (s, 1H), 4.67 (s, 1H), 4.45 (s, 2H), 4.11 (d, J = 13.6 Hz, 1H), 3.98 (d, J = 13.6 Hz, 1H), 3.44 – 3.37 (m, 2H), 2.16 – 2.09 (m, 3H), 1.89 (d, J = 2.0 Hz, 3H), 1.73 – 1.59 (m, 2H), 1.35 (s, 9H); **^{13}C NMR** (101 MHz, DMSO-d_6, 85 °C, as a mixture of **SI-9** : **SI-10** = 5:1) δ 153.6, 152.5, 148.2, 138.4, 134.4, 127.7, 126.9, 126.9, 126.8, 126.8, 78.5, 78.5, 72.4, 71.6, 71.5, 68.7, 67.8, 55.9, 55.5, 28.0, 27.6, 26.6, 23.1, 23.0, 14.8, 14.7; **FTIR** (thin film): 3429, 2974, 2930, 2855, 1704, 1685, 1365, 1332, 1254, 1164, 1080, 1012, 736, 697 cm^{-1}; **HRMS** (ESI) *m/z* $[M+Na]^+$ calcd for $C_{23}H_{31}NNaO_4$: 408.2145, found: 408.2147.

***NOTE*:** NMR spectra were taken at 85 °C due to the rotameric nature of the Boc amide in **SI-9** and **SI-10**.

*Preparation of Aldehyde **42***

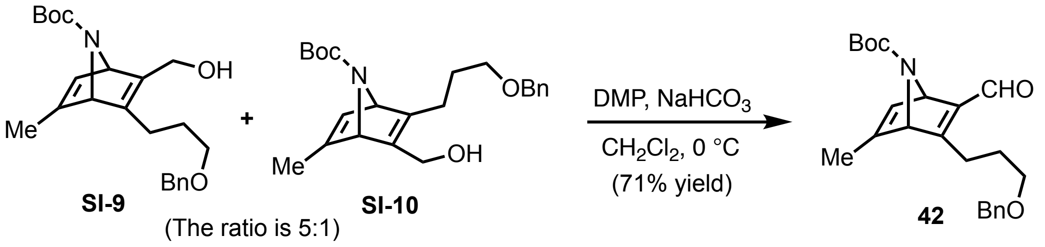

To a cooled solution (0 °C) containing a mixture of **SI-9** and **SI-10** (1.11 g, 2.88 mmol, 1.0 equiv, **SI-9** : **SI-10** = 5:1) in CH_2Cl_2 (15 mL), was added $NaHCO_3$ (1.06 g, 12.7 mmol, 4.4 equiv) followed by Dess-Martin periodinane (2.44 g, 5.76 mmol, 2.0 equiv) and the reaction mixture was allowed to stir for 2 h at 0°C. At which point, a solution of saturated aqueous $NaHCO_3$ (10 mL) followed by a 10% aqueous solution of $Na_2S_2O_3$ (10 mL) were sequentially added. The mixture was extracted twice with EtOAc (2 x 50 mL), and the combined organic layers were washed with brine (25 mL), dried over Na_2SO_4, filtered, and concentrated *in vacuo*. The resulting residue was purified by silica gel chromatography (60 g SiO_2, hexanes/EtOAc= 5:1 to 3:1) to afford **42** (788 mg, 71% yield) as a yellow oil: **^{1}H NMR** (400 MHz, DMSO-d_6, 85 °C) δ 9.80 (s, 1H), 7.36 – 7.24 (m, 5H), 6.50 (bs, 1H), 5.18 (bs, 1H), 4.91 (d, *J* = 2.0 Hz, 1H), 4.47 (s, 2H), 3.48 (t, *J* = 6.4 Hz, 2H), 2.90 – 2.70 (m, 2H), 1.92 (d, *J* = 1.9 Hz, 3H), 1.90 – 1.77 (m, 2H), 1.35 (s, 9H); **^{13}C NMR** (101 MHz, DMSO-d_6, 85 °C) δ 183.7, 153.5, 152.0, 138.2, 134.6, 127.7, 127.7, 126.9, 126.9, 126.8, 79.5, 73.5, 71.6, 68.3, 64.4, 27.5, 27.3, 24.2, 14.6; **FTIR** (thin film): 2856, 1707, 1651, 1366, 1327, 1254, 1161, 1099, 1079, 736, 698 cm^{-1}; **HRMS** (ESI) *m/z* $[M+Na]^+$ calcd for $C_{23}H_{29}NNaO_4$: 406.1989, found: 406.1989.

***NOTE*:** NMR spectra were taken at 85 °C due to the rotameric nature of the Boc amide in **42**.

***NOTE*:** Compound **42** decomposes slowly under the variable temperature NMR conditions (DMSO-d_6, 85 °C).

*Preparation of Alkyl Bromide **SI-12***

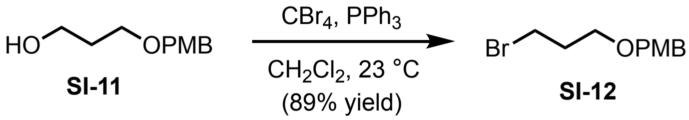

To a cooled solution (0 °C) of **SI-11**[14] (4.2 g, 21.4 mmol, 1.0 equiv) in CH_2Cl_2 (22 mL), was added carbon tetrabromide (7.8 g, 23.5 mmol, 1.1 equiv) and triphenylphosphine (6.2 g, 23.5 mmol, 1.1 equiv), and the reaction mixture was allowed to subsequently warm to 23 °C. After stirring at 23 °C for 12 h, the reaction mixture was concentrated *in vacuo*, filtered through short pad of Celite, washing with five portions of Et_2O (50 mL each), and the resulting filtrate was concentrated *in vacuo*. The resulting residue was purified by silica gel chromatography (60 g SiO_2, hexanes/EtOAc= 40:1 to 20:1) to afford **SI-12** (4.9 g, 89% yield) as a colorless oil: **^{1}H NMR** (400 MHz, chloroform-*d*) δ 7.26 (d, *J* = 8.6 Hz, 2H), 6.88 (d, *J* = 8.6 Hz, 2H), 4.45 (s, 2H), 3.81 (s, 3H), 3.58 (t, *J* = 5.9 Hz, 2H), 3.52 (t, *J* = 6.6 Hz, 2H), 2.12 (p, *J* = 6.2 Hz, 2H);**^{13}C NMR** (101 MHz, chloroform-*d*) δ 159.4, 130.5, 129.4, 114.0, 73.0, 67.6, 55.4, 33.1, 30.9; **FTIR** (thin film): 2858, 1612, 1511, 1362, 1301, 1243, 1172, 1095, 1033, 816 cm^{-1}; **HRMS** (ESI) *m/z* $[M+Na]^+$ calcd for $C_{11}H_{15}{}^{79}BrNaO_2$: 281.0148, found: 281.0151.

14. Y. Hayashi, H. Yamaguchi, M. Toyoshima, K. Okado, T. Toyo, M. Shoji, *Org. Lett.* **2008**, *10*, 1405–1408.

*Preparation of Grignard Reagent **43***

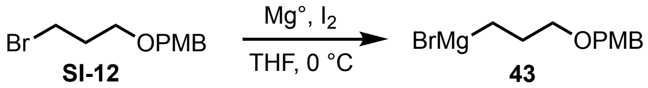

Fresh Grignard reagent **43** was prepared using the following procedure: To a flame-dried, 100 mL round-bottomed flask, under argon, containing magnesium turnings (377 mg, 15.5 mmol, 1.2 equiv) cooled to 0 °C in an ice/water bath was added a cooled solution (0 °C) of **SI-12** (3.35 g, 12.9 mmol 1.0 equiv) and iodine (65.6 mg, 0.258 mmol, 0.02 equiv) in THF (13 mL). Upon completion of the addition of **SI-12**, the reaction mixture was allowed to warm to 23 °C. The resulting Grignard reagent **43** was decanted from residual magnesium and used directly for the next step.

*Preparation of Alcohol **44***

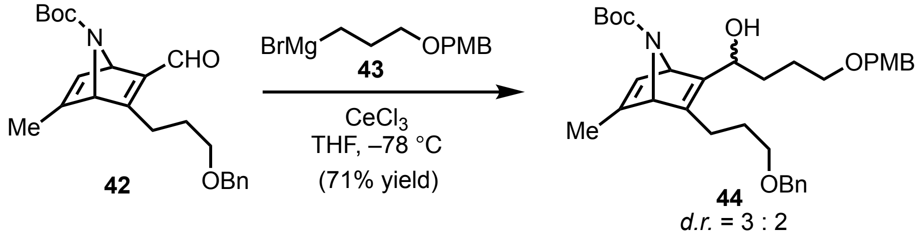

A flame-dried, 500-mL round-bottomed flask containing a large magnetic stir bar, was charged with cerium(III) chloride heptahydrate (4.81 g, 12.9 mmol, 3.5 equiv) and heated to 140 °C under vacuum (*ca.* 0.1 Torr) and allowed to stir at 140 °C for 2 h. At which point, the flask was cooled to 23 °C, THF (100 mL) was added, and the suspension was stirred at 23 °C for 16 h. Then the reaction mixture was cooled to –78 °C and freshly prepared Grignard reagent **43** (1.0 M in THF solution, 13 mL, 12.9 mmol, 3.5 equiv) was added at –78 °C. After stirring for 1.5 h, a solution of **42** (1.40 g, 3.65 mmol, 1.0 equiv) in THF (20 mL) was cooled to –78 °C and added to the reaction mixture, which was allowed to stir for an additional 1 h at –78 °C. At which point, a solution of saturated aqueous NH_4Cl (25 mL) was added to the reaction mixture, and it was then allowed to warm to 23 °C, filtered through short pad of Celite, and the filtrate was extracted five times with EtOAc (5 x 50 mL). The combined organic layers were washed with brine (50 mL), dried over Na_2SO_4, filtered, and concentrated *in vacuo*. The resulting residue was purified by silica gel chromatography (80 g SiO_2, hexanes/EtOAc= 2:1 to 1:1) to afford **44** (1.46 g, 71% yield, as a 3:2 mixture of diastereomers) as a colorless oils: **^{1}H NMR** (400 MHz, DMSO-d_6, 85 °C, as a 3:2 mixture of diastereomers) 7.37 – 7.24 (m, 5H), 7.21 (dd, J = 8.6, 2.4 Hz, 2H), 6.88 (dd, J = 8.6, 1.7 Hz, 2H), 6.38 (bs, 1H), [4.92 (t, J = 2.3 Hz, 0.6H), 4.87 (t, J = 2.3 Hz, 0.4H), 1H total], 4.57 (s, 1H), 4.45 (s, 2H), [4.36 (s, 0.8H), 4.34 (s, 1.2H), 2H total], 4.28 – 4.24 (m, 1H), 3.75 (s, 3H), 3.52 – 3.30 (m, 4H), 2.40 – 2.13 (m, 2H), 1.88 (s, 3H), 1.77 – 1.57 (m, 3H), 1.56 – 1.43 (m, 3H), [1.35 (s, 5.4H), 1.34 (s, 3.6H), 9H total]; **^{13}C NMR** (101 MHz, DMSO-d_6, 85 °C) δ 158.4, 158.4 153.5, 151.3, 151.3, 150.4, 138.4, 138.4, 134.8, 134.8, 130.5, 130.5, 128.4, 128.4, 127.7, 126.9, 126.8, 126.7, 126.7, 113.3, 113.3, 78.5, 78.5, 71.6, 71.5, 71.5, 71.2, 71.2, 71.1, 69.2, 69.0, 68.9, 68.9, 65.2, 65.1, 54.7, 30.8, 28.1, 28.1, 27.6, 27.5, 24.9, 23.3, 23.2, 14.8, 14.7; **FTIR** (thin film): 3453, 2932, 2854, 1703, 1512, 1365, 1339, 1246, 1168, 1092, 1031, 821, 737, 698 cm^{-1}; **HRMS** (ESI): m/z $[M+Na]^+$ calcd for $C_{34}H_{45}NNaO_6$: 586.3139, found: 586.3136.

***NOTE*:** NMR spectra were taken at 85 °C due to the rotameric nature of the Boc amide in **44**.

*Preparation of TBS Ether **SI-13***

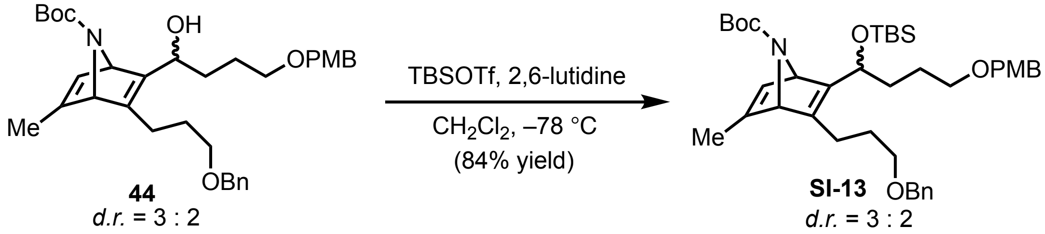

To a –78 °C solution of **44** (430 mg, 0.830 mmol, 1.0 equiv, 3:2 mixture of diastereomers) in CH_2Cl_2 (17 mL) was added 2,6-lutidine (0.58 mL, 4.98 mmol, 6.0 equiv), *tert*-butyldimethylsilyl trifluoromethanesulfonate (0.57 mL, 2.49 mmol, 3.0 equiv), and the reaction mixture was allowed to stir at –78 °C for 50 minutes. At which point, a solution of saturated aqueous $NaHCO_3$ (25 mL) was added, the mixture was allowed to warm to 23 °C, and then was extracted twice with EtOAc (2 x 50 mL). The combined organic layers were washed with brine (25 mL), dried over Na_2SO_4, filtered, and concentrated *in vacuo*. The resulting residue was purified by silica gel chromatography (30 g SiO_2, hexanes/EtOAc= 8:1 to 5:1) to afford **SI-13** (466 mg, 84% yield, as 3:2 mixture of diastereomers) as colorless oils: **^{1}H NMR** (400 MHz, DMSO-d_6, 85 °C, as 3:2 mixture of diastereomers) δ 7.38 – 7.23 (m, 5H), 7.20 (d, *J* = 8.8 Hz, 2H), 6.87 (d, *J* = 8.8 Hz, 2H), [6.42 (s, 0.6H), 6.34 (s, 0.4H), 1H total], [4.89 (bs, 0.6H), 4.84 (bs, 0.4H), 1H total], [4.63 (bs, 0.4H), 4.62 (bs, 0.6H), 1H total], 4.45 (s, 2H), 4.45 – 4.41 (m, 1H), [4.35 (s, 0.8H), 4.33 (s, 1.2H), 2H total], 3.74 (s, 3H), 3.49 – 3.32 (m, 4H), 2.39 – 2.27 (m, 1H), 2.25 – 2.14 (m, 1H), 1.87 (d, *J* = 1.9 Hz, 3H), 1.79 – 1.24 (m, 6H), 1.34 (d, *J* = 1.6 Hz, 9H), [0.88 (s, 5.4H), 0.85 (s, 3.6H), 9H total], [0.01 (s, 1.8H), -0.02 (s, 1.8H), -0.03 (s, 1.2H), -0.13 (s, 1.2H), 6H total]; **^{13}C NMR** (101 MHz, DMSO-d_6, 85 °C, as 3:2 mixture of diastereomers) δ 158.4, 158.4, 151.0, 138.4, 138.3, 134.6, 130.4, 130.4, 128.4, 128.4, 127.7, 126.8, 126.8, 126.7, 126.7, 113.3, 113.3, 78.6, 78.4, 71.4, 71.4, 71.2, 71.2, 71.1, 69.0, 68.9, 67.1, 66.9, 66.1, 65.8, 65.8, 54.7, 31.4, 28.0, 27.6, 27.5, 25.3, 25.3, 24.7, 23.3, 17.4, 14.6, -5.3, -5.4, -5.6, -5.6; **FTIR** (thin film): 2929, 2854, 1704, 1513, 1364, 1340, 1247, 1170, 1079, 1035, 831, 775, 697 cm^{-1}; **HRMS** (ESI) *m/z* $[M+Na]^+$ calcd for $C_{40}H_{59}NNaO_6Si$: 700.4004, found: 700.4001.

***NOTE*:** NMR spectra were taken at 85 °C due to the rotameric nature of the Boc amide in **SI-13**.

*Preparation of Primary Alcohol **SI-14***

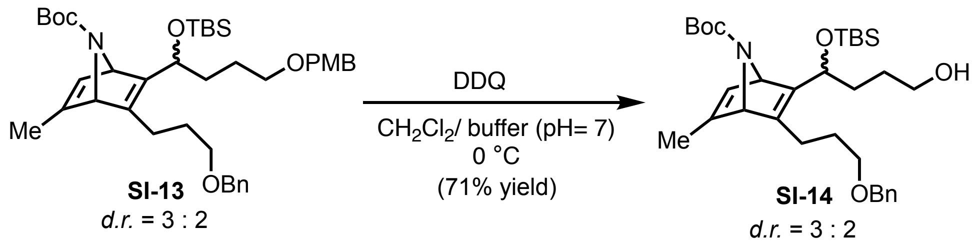

To a cooled (0 °C) biphasic solution of **SI-13** (412.7 mg, 0.618 mmol, 1.0 equiv, as a 3:2 mixture of diastereomers) in CH_2Cl_2 (26 mL) and pH 7 phosphate buffer (5 mL), was added 2,3-dichloro-5,6-dicyano-1,4-benzoquinone (561 mg, 2.47 mmol, 4.0 equiv). The reaction mixture was then allowed to stir at 0 ˚C for 1.5 h. At which point, a saturated aqueous solution of $NaHCO_3$ (10 mL) was added followed by a 10% aqueous solution of $Na_2S_2O_3$ (10 mL), and the mixture was extracted twice with EtOAc (2 x 50 mL). The combined organic layers were washed with brine (25 mL), dried over Na_2SO_4, filtered, and concentrated *in vacuo*. The resulting residue was purified by silica gel chromatography (20 g SiO_2, hexanes/EtOAc= 5:1 to 2:1) to afford **SI-14** (245 mg, 71% yield, as a 3:2 mixture of diastereomers) as yellow oils: **^{1}H NMR (**400 MHz, DMSO-*d*$_6$, 85 °C, as a 3:2 mixture of diastereomers) δ 7.39 – 7.20 (m, 5H), [6.44 (bs, 0.6H), 6.35 (brs, 0.4H), 1H total], [4.91 (s, 0.6H), 4.84 (s, 0.4H), 1H total], 4.62 (s, 1H), 4.53 – 4.36 (m, 3H), 3.55 – 3.31 (m, 4H), 2.40 – 2.14 (m, 2H), 1.88 (bs, 3H), 1.80 – 1.55 (m, 2H), 1.55 – 1.31 (m, 2H), 1.35 (s, 9H), 1.31 – 1.12 (m, 2H), 0.99 – 0.77 (m, 9H), [0.02 (s, 1.8H), -0.01 (s, 1.2H), -0.02 (s, 1.8H), -0.12 (s, 1.2H) 6H total]; **^{13}C NMR** (101 MHz, DMSO-*d*$_6$, 85 °C, as a 3:2 mixture of diastereomers) δ 151.1, 138.4, 138.4, 127.7, 127.7, 126.9, 126.8, 126.8, 126.7, 78.6, 78.6, 78.4, 78.4, 71.5, 68.9, 68.8, 67.2, 67.1, 60.5, 60.4, 31.3, 28.0, 28.0, 27.6, 27.5, 25.4, 25.3, 25.3, 23.3, 23.3, 17.4, 17.3, 14.7, 14.7, -3.7, -5.2, -5.4, -5.5, -5.6; **FTIR** (thin film): 3458, 2929, 2855, 1705, 1365, 1252, 1167, 1057, 833, 774, 697 cm^{-1}; **HRMS** (ESI) *m/z* $[M+Na]^+$ calcd for $C_{32}H_{51}NNaO_5Si$: 580.3429, found: 580.3428.

***NOTE*:** NMR spectra were taken at 85 °C due to the rotameric nature of the Boc amide in **SI-14**.

***NOTE*:** Compound **SI-14** decomposes slowly under the variable temperature NMR conditions (DMSO-*d*$_6$, 85 °C).

*Preparation of Aldehyde **29***

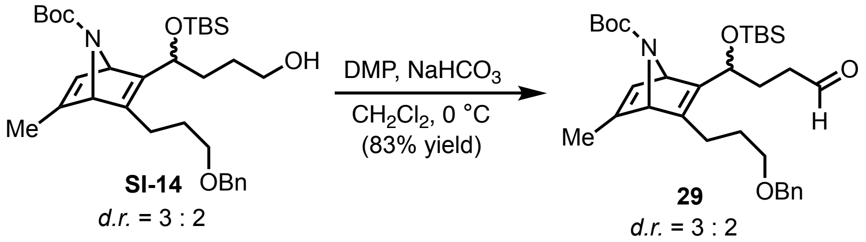

To a cooled solution (0 °C) of **SI-14** (220.3 mg, 0.395 mmol, 1.0 equiv, as a 3:2 mixture of diastereomers) in CH_2Cl_2 (4 mL) was added $NaHCO_3$ (146 mg, 1.74 mmol, 4.4 equiv), Dess-Martin periodinane (335 mg, 0.790 mmol, 2.0 equiv), and allowed to stir at 0 °C for 3 h. At which point, a saturated aqueous solution of $NaHCO_3$ (10 mL) was added followed by a 10% aqueous solution of $Na_2S_2O_3$ (10 mL), and the mixture was extracted twice with EtOAc (2 x 50 mL). The combined organic layers were washed with brine (25 mL), dried over Na_2SO_4, filtered, and concentrated *in vacuo*. The resulting residue was purified by silica gel chromatography (3 g SiO_2, hexanes/EtOAc= 8:1) to afford **29** (183 mg, 83% yield, as a 3:2 mixture of diastereomers) as colorless oils: **^{1}H NMR** (400 MHz, DMSO-d_6, 85 °C, as a 3:2 mixture of diastereomers) δ [9.67 (s, 0.4H), 9.63 (s, 0.6H), 1H total], 7.39 – 7.22 (m, 5H), [6.47 (t, J = 2.3 Hz, 0.6H), 6.35 (bs, 0.4H), 1H total], [4.91 (s, 0.6H), 4.85 (s, 0.4H), 1H total], 4.64 (bs, 1H), 4.54 – 4.41 (m, 3H), 3.55 – 3.35 (m, 2H), 2.41 – 2.13 (m, 4H), [1.89 (d, J = 2.0 Hz, 1.8H), 1.88 (d, J = 2.0 Hz, 1.2H), 3H total], 1.81 – 1.57 (m, 3H), 1.57 – 1.38 (m, 1H), [1.36 (s, 3.6H), 1.35 (s, 5.4H), 9H total], [0.88 (s, 5.4H), 0.86 (s, 3.6H), 9H total], [0.02 (s, 1.8H), -0.01 (s, 1.2H), -0.01 (s, 1.8H), -0.12 (s, 1.2H), 6H total]; **^{13}C NMR** (101 MHz, DMSO-d_6, 85 °C, as a 3:2 mixture of diastereomers) δ 201.9, 201.8, 151.1, 138.4, 138.3, 134.5, 127.7, 126.8, 126.8, 78.8, 78.5, 71.5, 68.9, 66.7, 66.3, 66.1, 65.8, 27.9, 27.6, 27.5, 27.2, 25.4, 25.3, 25.3, 23.3, 17.3, 14.7, 14.6, -3.7, -5.3, -5.5, -5.6, -5.7; **FTIR** (thin film): 2929, 2856, 1706, 1366, 1253, 1166, 1075, 834, 776, 697 cm^{-1}; **HRMS** (ESI) *m/z* $[M+Na]^+$ calcd for $C_{32}H_{49}NNaO_5Si$: 578.3272, found: 578.3270.

***NOTE*:** NMR spectra were taken at 85 °C due to the rotameric nature of the Boc amide in **29**.

*Preparation of Spirocycles **48a** and **48b***

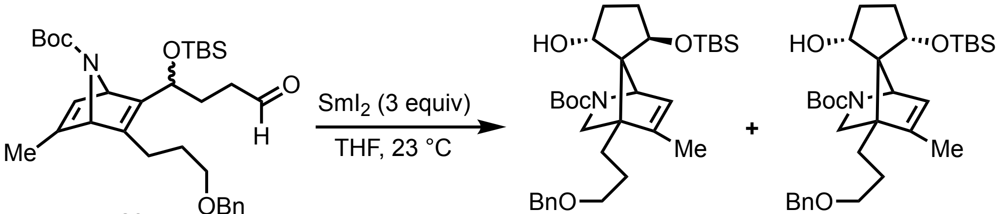

29 **48a** (42% yield) **48b** (29% yield)

Fresh SmI_2 reagent (0.05 M in THF) was prepared using the following procedure: To a Schlenk tube equipped with a stir-bar and a reflux condenser was added freshly shaved samarium (328 mg, 2.17 mmol, 1.8 equiv), 1,2-diiodoethane (340 mg, 1.21 mmol, 1.0 equiv), and the flask was immediately covered with aluminum foil due to the light sensitivity of the reagent. Then dry THF (24 mL) was added with stirring, and the reaction mixture was heated to 55 ˚C, in the dark, for 4 h. At which point the reaction mixture was allowed to cool to 23 ˚C and settle for at least 10 min prior to use. To a solution of **29** (128 mg, 0.230 mmol, 1.0 equiv, as a 3:2 mixture of diastereomers) in THF (12 mL) was dropwise added the fresh SmI_2 solution (13.8 mL, 0.690 mmol, 3.0 equiv) at 23 ˚C. After stirring at 23 ˚C for 1h, air was bubbled into the reaction mixture the reaction color changes from blue to yellow upon quenching of any active radical species. Then a solution of saturated, aqueous NH_4Cl (10 mL) was added followed by a saturated aqueous solution of Rochelle's salt (10 mL). The suspension was extracted twice with EtOAc (2 x 25 mL) and the combined organic layers were washed with brine (25 mL), dried over Na_2SO_4, filtered, and concentrated *in vacuo*. The resulting residue was purified by silica gel chromatography (10 g SiO_2, hexanes/EtOAc= 4:1) to afford **48a** (54 mg, 42% yield) as a colorless oil and **48b** (38 mg, 29% yield) as a colorless oil: **48a:** **^{1}H NMR** (400 MHz, DMSO-d_6, 85 °C) δ 7.36 – 7.24 (m, 5H), 5.81 (bs, 1H), 4.47 (s, 2H), 4.20 (d, J = 2.6 Hz, 1H), 4.10 – 4.06 (m, 1H), 3.83 (bs, 1H), 3.76 (bs, 1H), 3.44 (td, J = 6.2, 2.2 Hz, 2H), 3.23 (d, J = 9.7 Hz, 1H), 2.52 – 2.49 (m, 1H), 2.16 – 2.08 (m, 1H), 1.99 – 1.92 (m, 1H), 1.89 – 1.72 (m, 2H), 1.81 (d, J = 1.2 Hz, 3H), 1.63 – 1.47 (m, 3H), 1.44 – 1.33 (m, 1H), 1.38 (s, 9H), 0.85 (s, 9H), 0.00 (s, 3H), -0.02 (s, 3H); **^{13}C NMR** (101 MHz, DMSO-d_6, 85 °C) δ 153.9, 149.9, 138.4, 127.6, 126.8, 126.7, 124.6, 77.3, 76.1, 74.7, 71.6, 70.3, 64.2, 59.2, 57.4, 48.7, 32.7, 32.5, 27.9, 25.5, 25.5, 23.8, 17.2, 14.0, -5.1, -5.2; **FTIR** (thin film): 3462, 2929, 2855, 1692, 1364, 1250, 1175, 1101, 1013, 833, 773, 697 cm^{-1}; **HRMS** (ESI) *m/z* [M+Na]$^+$ calcd for $C_{32}H_{51}NNaO_5Si$: 580.3429, found: 580.3425; **48b:** **^{1}H NMR** (400 MHz, DMSO-d_6, 85 °C) δ 7.39 – 7.24 (m, 5H), 5.97 (bs, 1H), 4.48 (s, 2H), 4.35 (d, J = 2.8 Hz, 1H), 3.77 (dd, J = 5.1, 2.8 Hz, 1H), 3.69 (bs, 1H), 3.51 (bs, 1H), 3.47 (t, J = 6.0 Hz, 2H), 3.17 (d, J = 9.8 Hz, 1H), 2.61 (d, J = 9.8 Hz, 1H), 1.93 – 1.66 (m, 3H), 1.80 (d, J = 2.0 Hz, 3H), 1.66 – 1.46 (m, 4H), 1.41 – 1.31 (m, 1H), 1.37 (s, 9H), 0.88 (s, 9H), 0.00 (s, 3H), -0.01 (s, 3H); **^{13}C NMR** (101 MHz, DMSO-d_6, 85 °C) δ 154.2, 146.4, 138.4, 128.4, 127.7, 126.9, 126.8, 77.3, 76.5, 76.2, 71.5, 71.2, 69.9, 62.5, 57.6, 48.9, 33.4, 31.9, 27.8, 25.3, 25.2, 22.9, 17.1, 13.7, -4.8, -5.5; **FTIR** (thin film): 3485, 2929, 2855, 1694, 1364, 1251, 1174, 1083, 833, 773, 697 cm^{-1}; **HRMS** (ESI) *m/z* [M+Na]$^+$ calcd for $C_{32}H_{51}NNaO_5Si$: 580.3429, found: 580.3426.

***NOTE*:** NMR spectra were taken at 85 °C due to the rotameric nature of the Boc amides in **48a** and **48b**.

*Preparation of Secondary Amine **49***

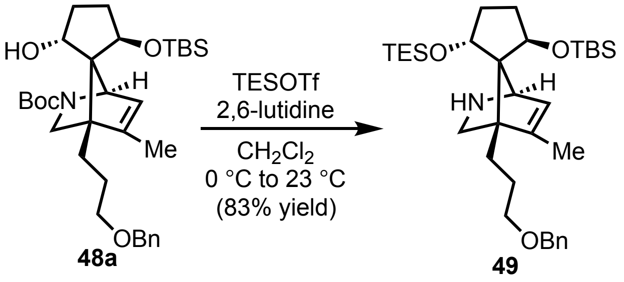

To a cooled solution (0 °C) of **48a** (9.7 mg, 0.0174 mmol, 1.0 equiv) in CH_2Cl_2 (0.9 mL) was added 2,6-lutidine (20 μL, 0.174 mmol, 10.0 equiv) followed by triethylsilyl trifluoromethanesulfonate (20 μL, 0.0869 mmol, 5.0 equiv), and the reaction mixture was allowed to stir at 0 °C for 1.5 h, then allowed to warm to 23 °C and stirred for an additional 1 h. At which point, a second portion of 2,6-lutidine (20 μL, 0.174 mmol, 10.0 equiv) followed by triethylsilyl trifluoromethanesulfonate (20 μL, 0.0869 mmol, 5.0 equiv) was added at 23 °C, and the reaction mixture was allowed to stir at 23 °C for an additional 3.5 h. Then a solution of saturated aqueous $NaHCO_3$ (5 mL) was added and the mixture was extracted three times with EtOAc (3 x 10 mL). The combined organic layers were washed with brine (15 mL), dried over Na_2SO_4, filtered, and concentrated *in vacuo*. The residue was purified by silica gel chromatography (1 g SiO_2, hexanes/EtOAc= 1:1, and then, CH_2Cl_2/MeOH= 10:1) to afford **49** (8.2 mg, 83% yield) as a light brown oil; **^{1}H NMR** (400 MHz, acetonitrile-d_3) δ 7.41 – 7.24 (m, 5H), 7.17 (bs, 1H), 6.73 (bs, 1H), 5.86 (bs, 1H), 4.49 (s, 2H), 4.41 (t, J = 2.8 Hz, 1H), 4.37 (t, J = 8.0 Hz, 1H), 4.03 (t, J = 5.6 Hz, 1H), 3.49 (t, J = 5.9 Hz, 2H), 3.28 (ddd, J = 10.5, 7.3, 2.9 Hz, 1H), 2.65 (ddd, J = 10.5, 6.9, 3.6 Hz, 1H), 2.26 – 2.10 (m, 2H), 1.98 – 1.79 (m, 3H), 1.94 (s, 3H), 1.64 – 1.50 (m, 2H), 1.39 – 1.24 (m, 1H), 0.98 (t, J = 7.9 Hz, 9H), 0.86 (s, 9H), 0.72 (q, J = 7.6 Hz, 6H), 0.01 (s, 3H), 0.00 (s, 3H); **^{13}C NMR** (101 MHz, acetonitrile-d_3) δ 159.0, 140.0, 129.2, 128.5, 128.4, 122.9, 75.9, 74.5, 73.6, 73.4, 71.4, 68.1, 58.9, 47.3, 32.0, 31.6, 27.2, 26.4, 24.6, 18.5, 15.2, 7.2, 5.4, -4.3, -4.4; **FTIR** (thin film): 2954, 2879, 2857, 1456, 1361, 1287, 1251, 1161, 1100, 1054, 1029, 837, 777, 733, 637 cm^{-1}; **HRMS** (ESI) *m/z* $[M+H]^+$ calcd for $C_{33}H_{58}NO_3Si_2$: 572.3950, found: 572.3945.

III. NMR SPECTRA

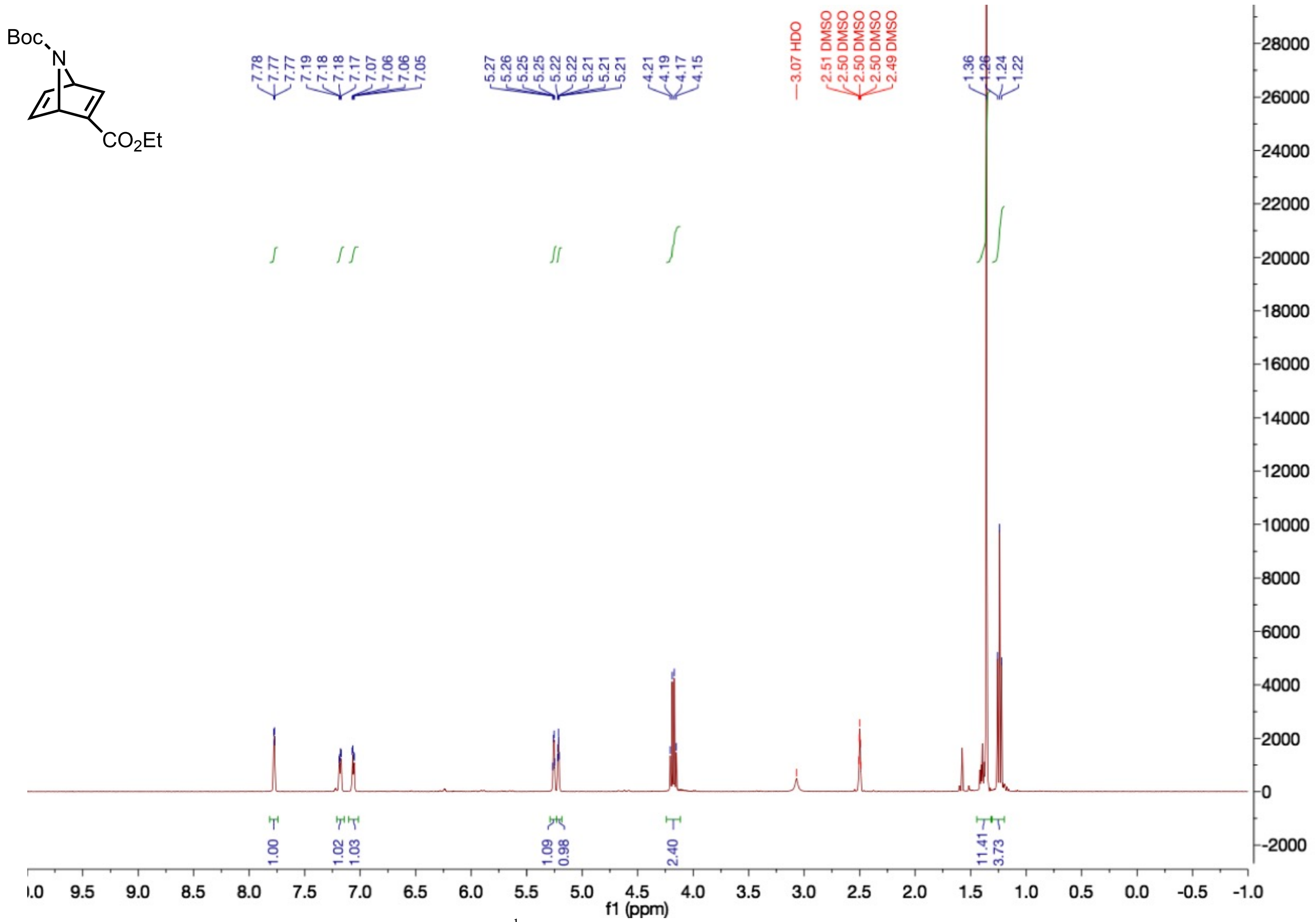

Figure S1. ^{1}H NMR (400 MHz, DMSO-d_6, 85 °C) Ester **12**

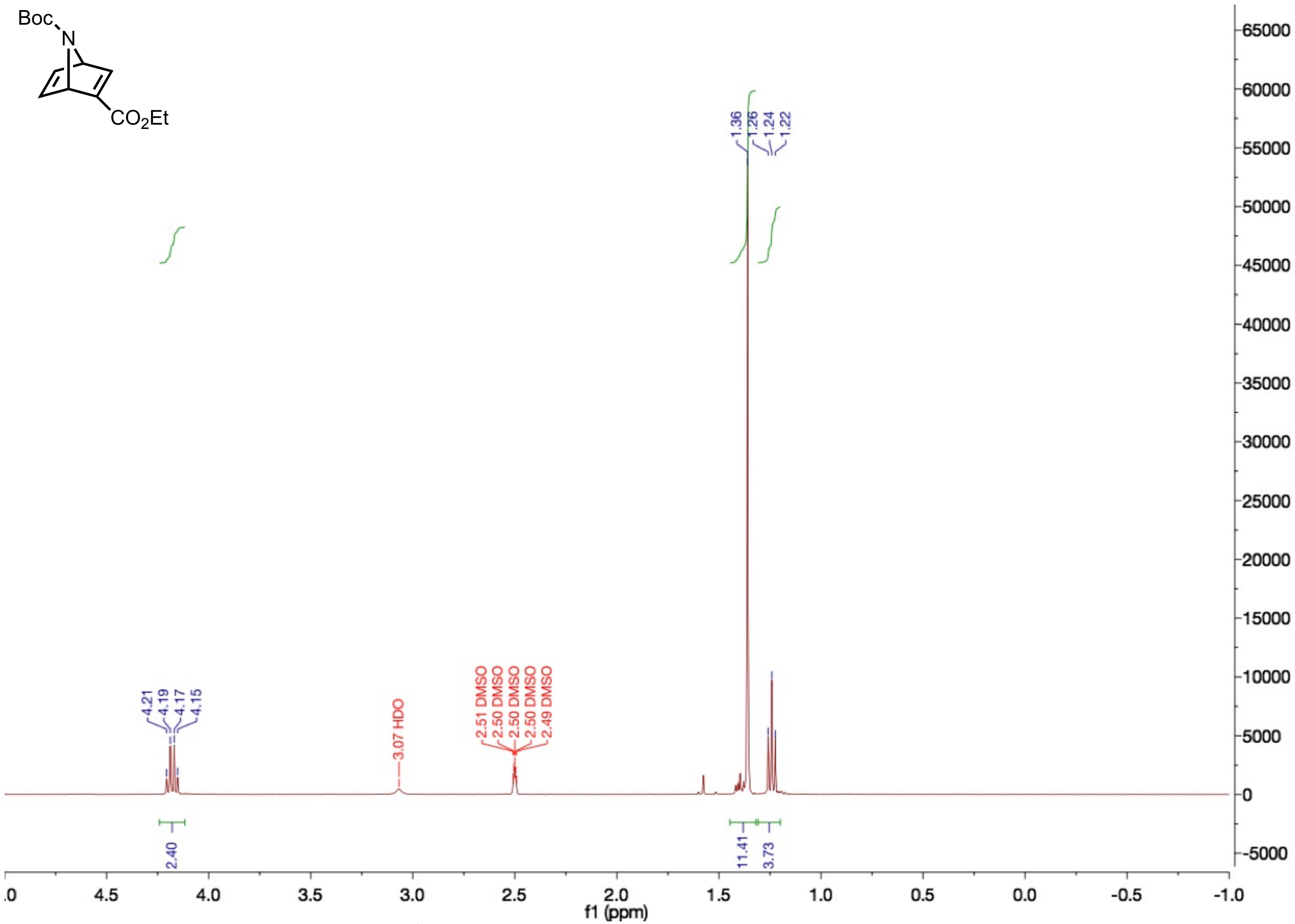

Figure S2. ^{1}H NMR (400 MHz, DMSO-d_6, 85 °C) Ester **12** (-1 – 5 ppm inset)

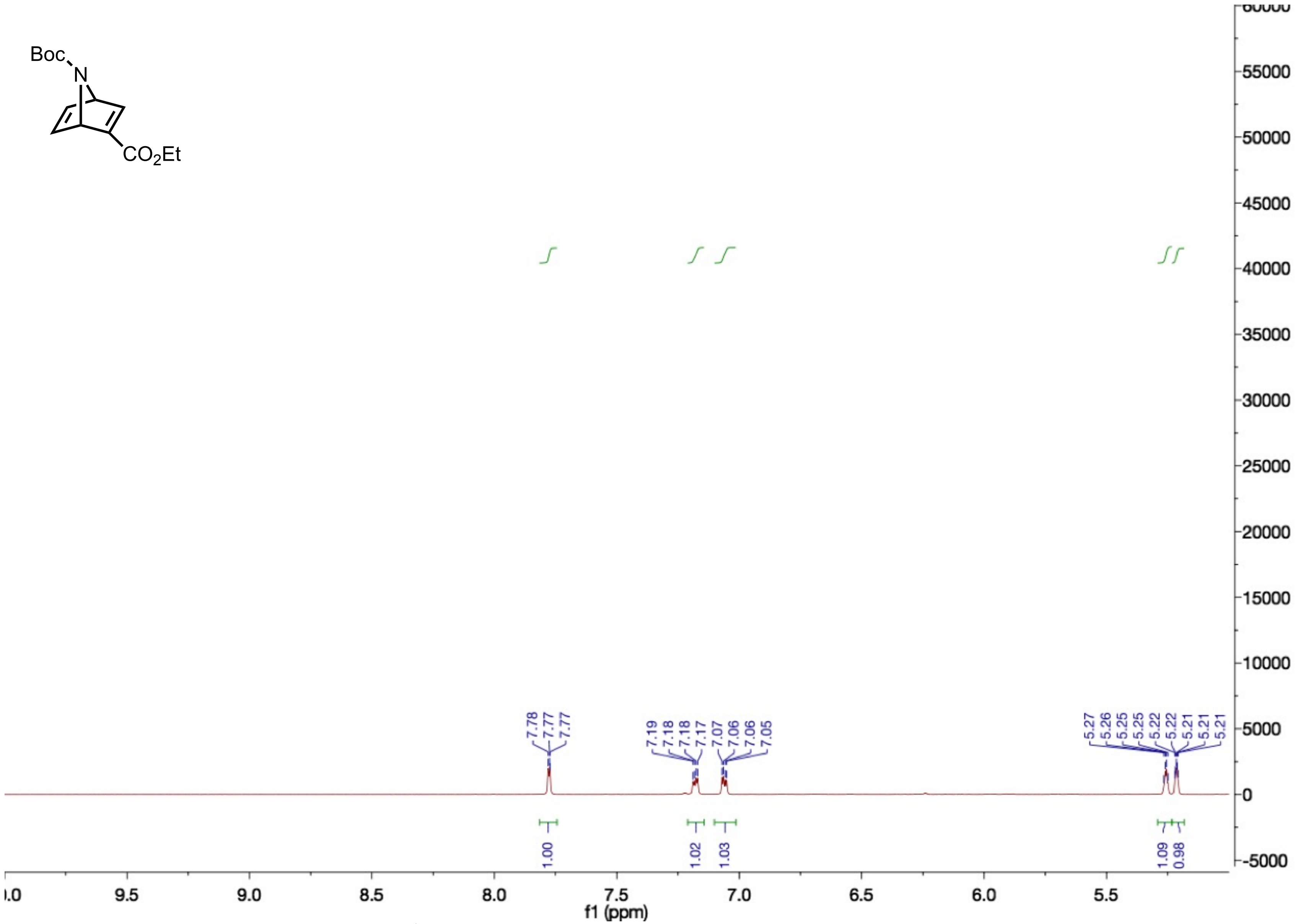

Figure S3. ^{1}H NMR (400 MHz, DMSO-d_6, 85 °C) Ester **12** (5 – 10 ppm inset)

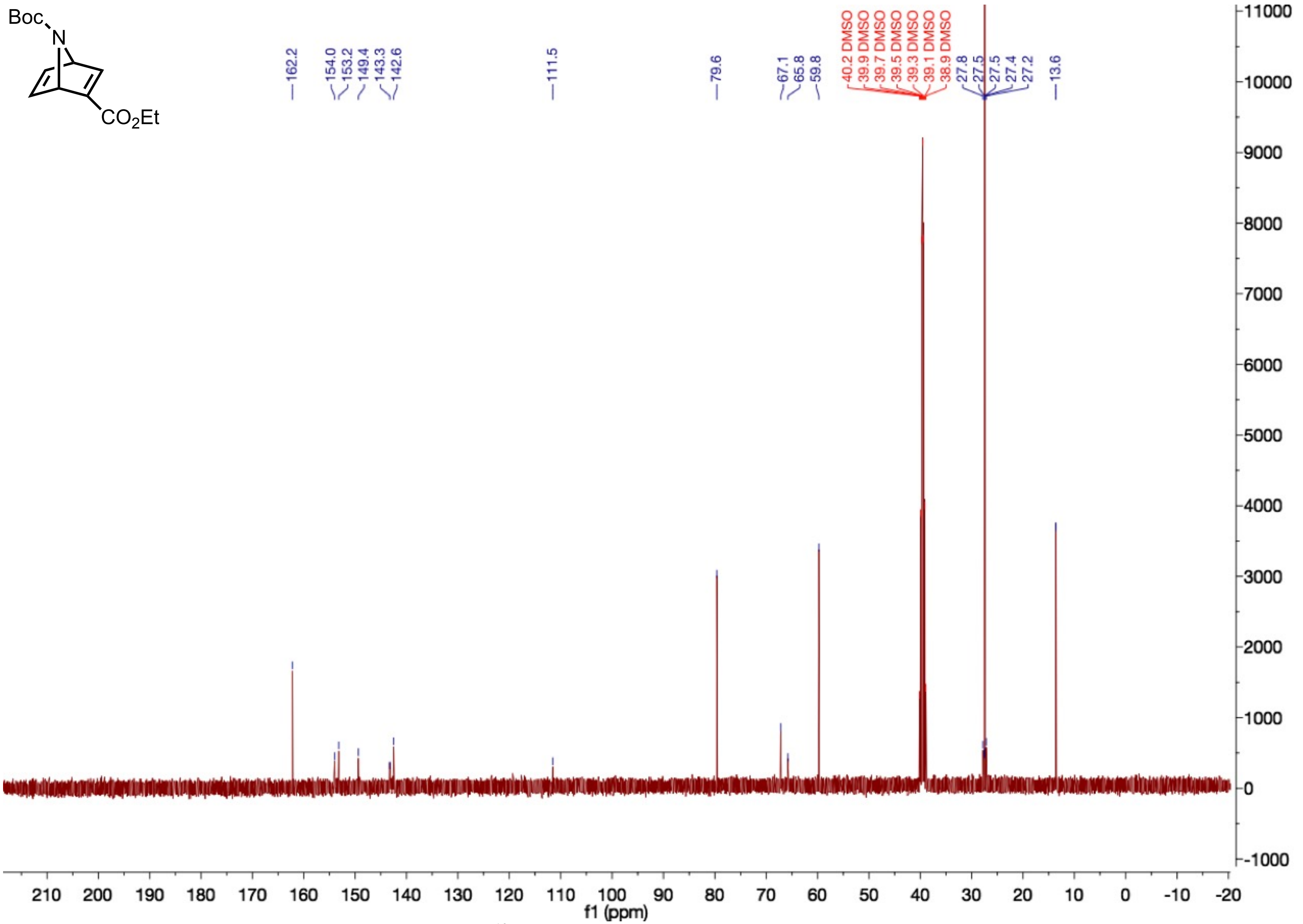

Figure S4. ^{13}C NMR (101 MHz, DMSO-d_6, 85 °C) Ester **12**

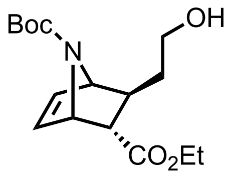

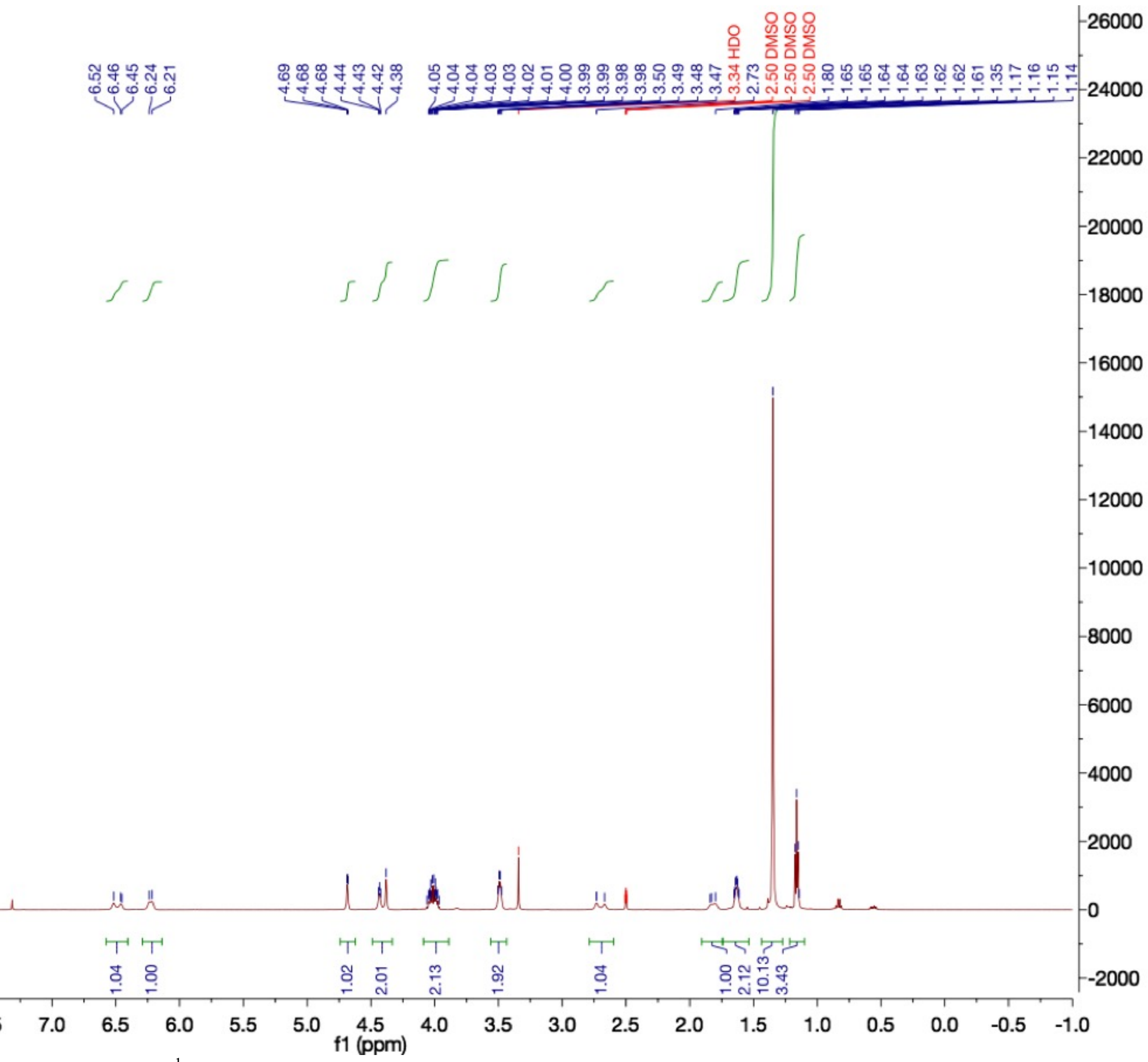

Figure S5. ^1H NMR (600 MHz, DMSO-d_6, 27 °C) Primary Alcohol **16**

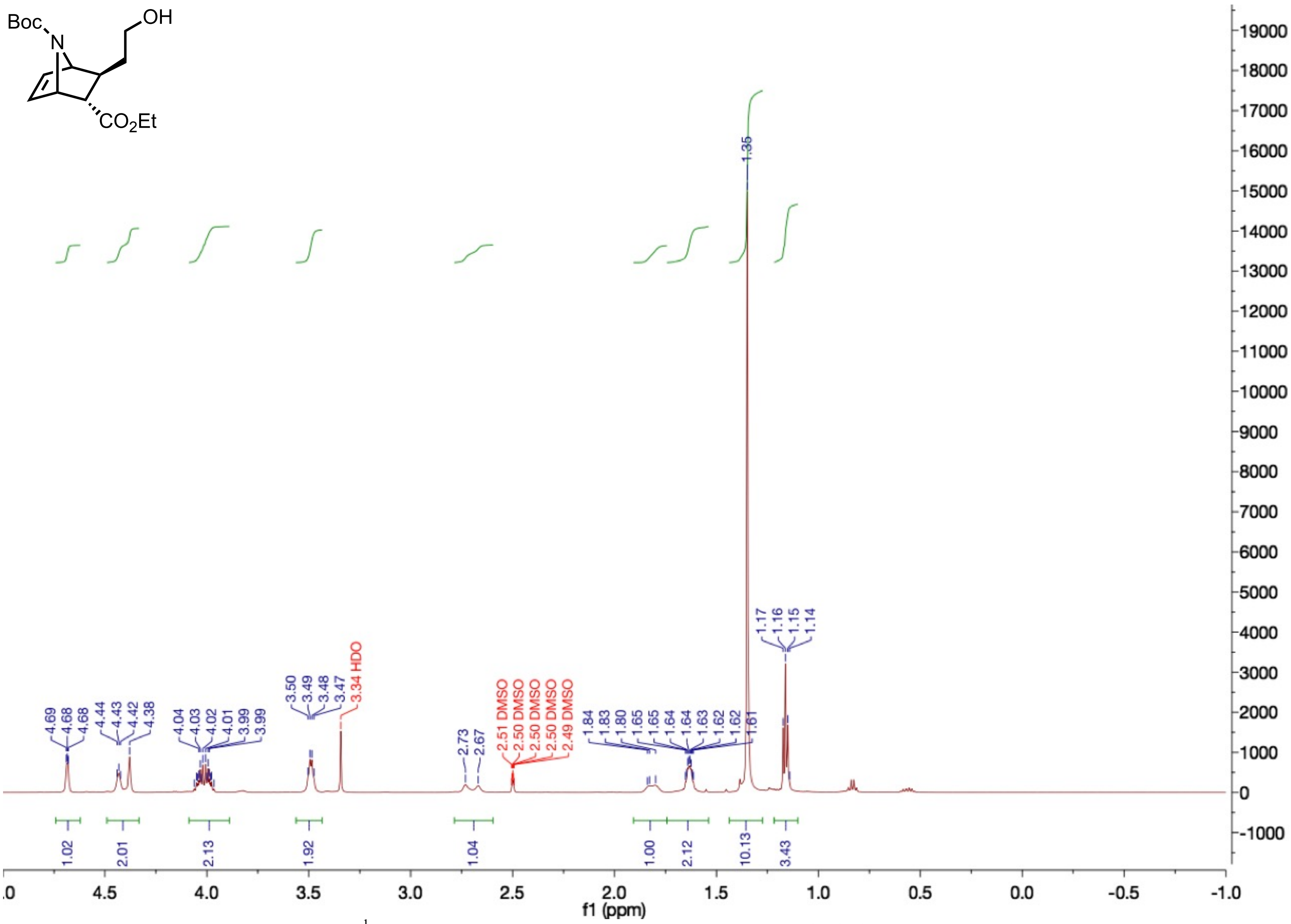

Figure S6. ¹H NMR (600 MHz, DMSO-d_6, 27 °C) Primary Alcohol **16** (-1 – 5 ppm inset)

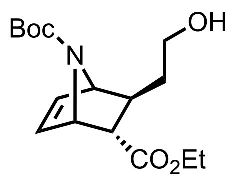

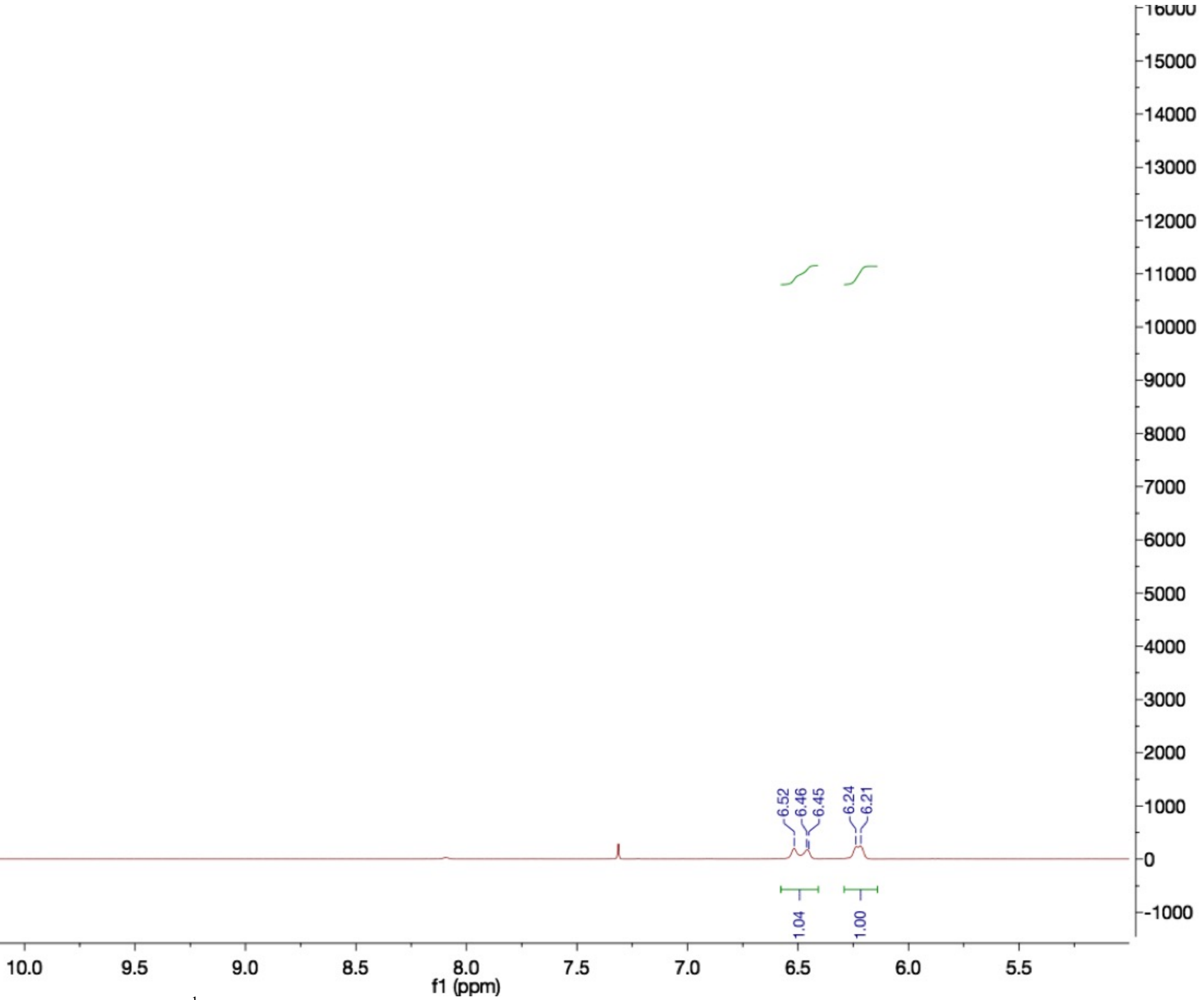

Figure S7. ^{1}H NMR (600 MHz, DMSO-d_6, 27 °C) Primary Alcohol **16** (5 – 10 ppm inset)

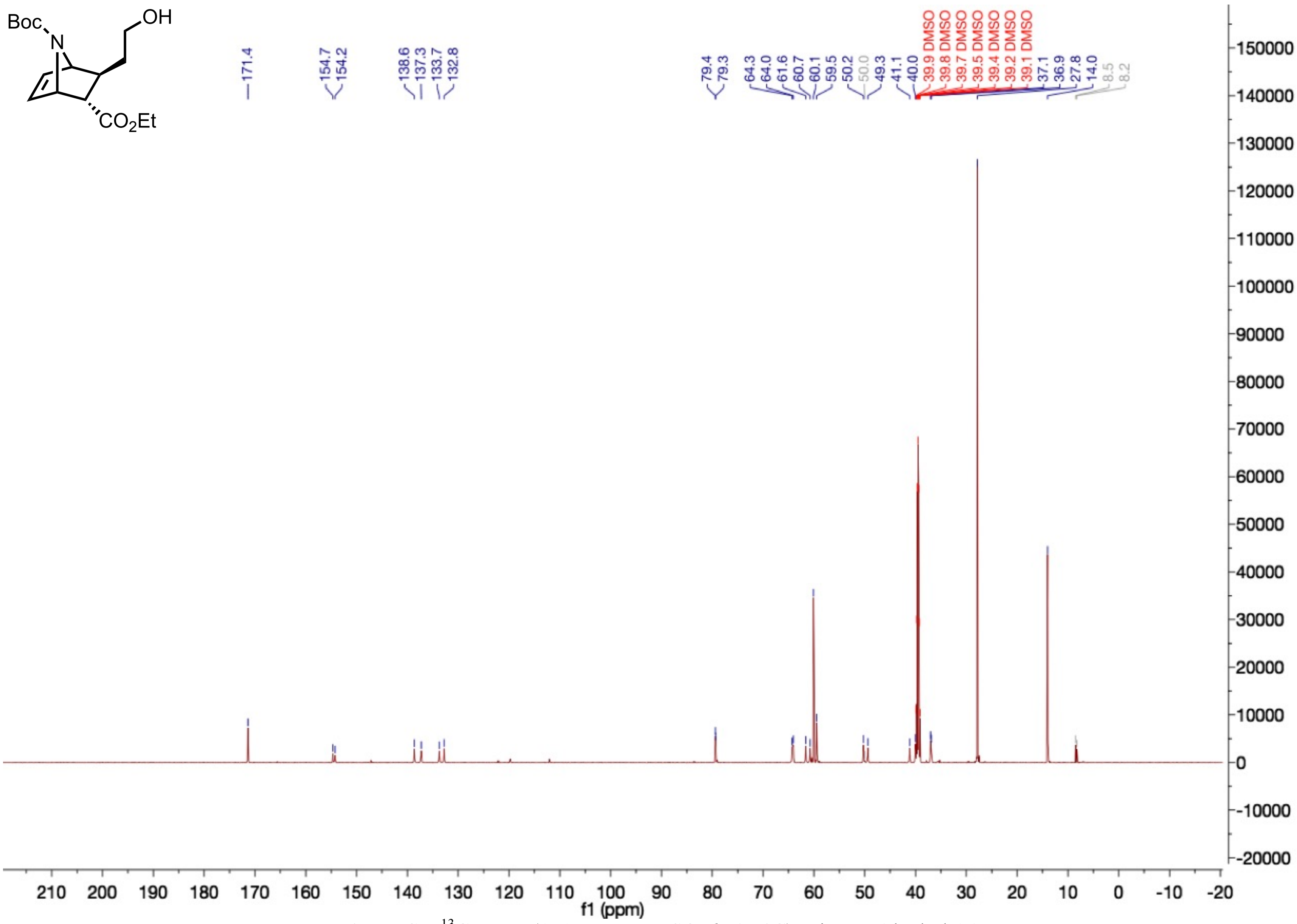

Figure S8. ^{13}C NMR (151 MHz, DMSO-d_6, 27 °C) Primary Alcohol **16**

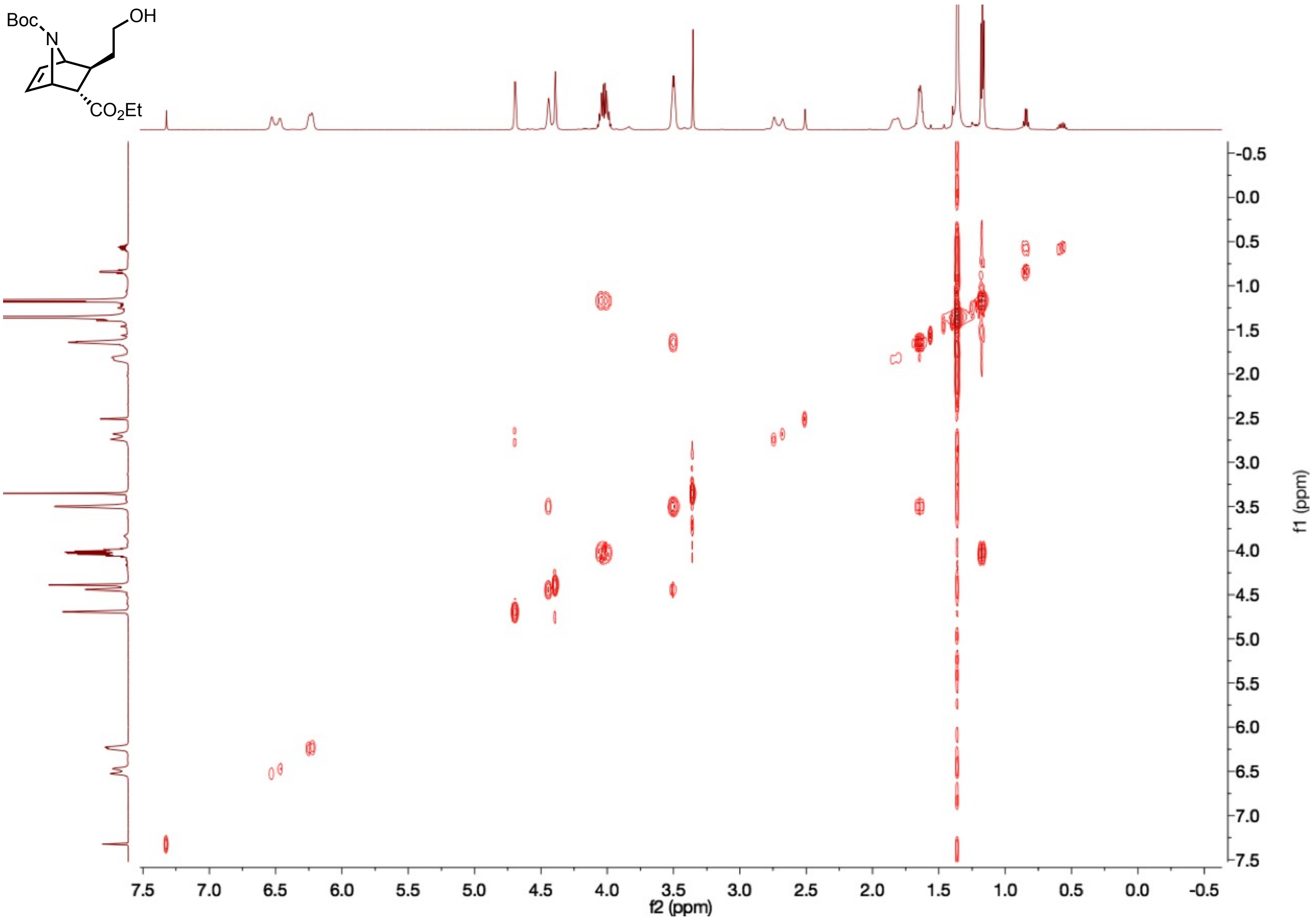

Figure S9. COSY (DMSO-d_6, 27 °C) Primary Alcohol **16**

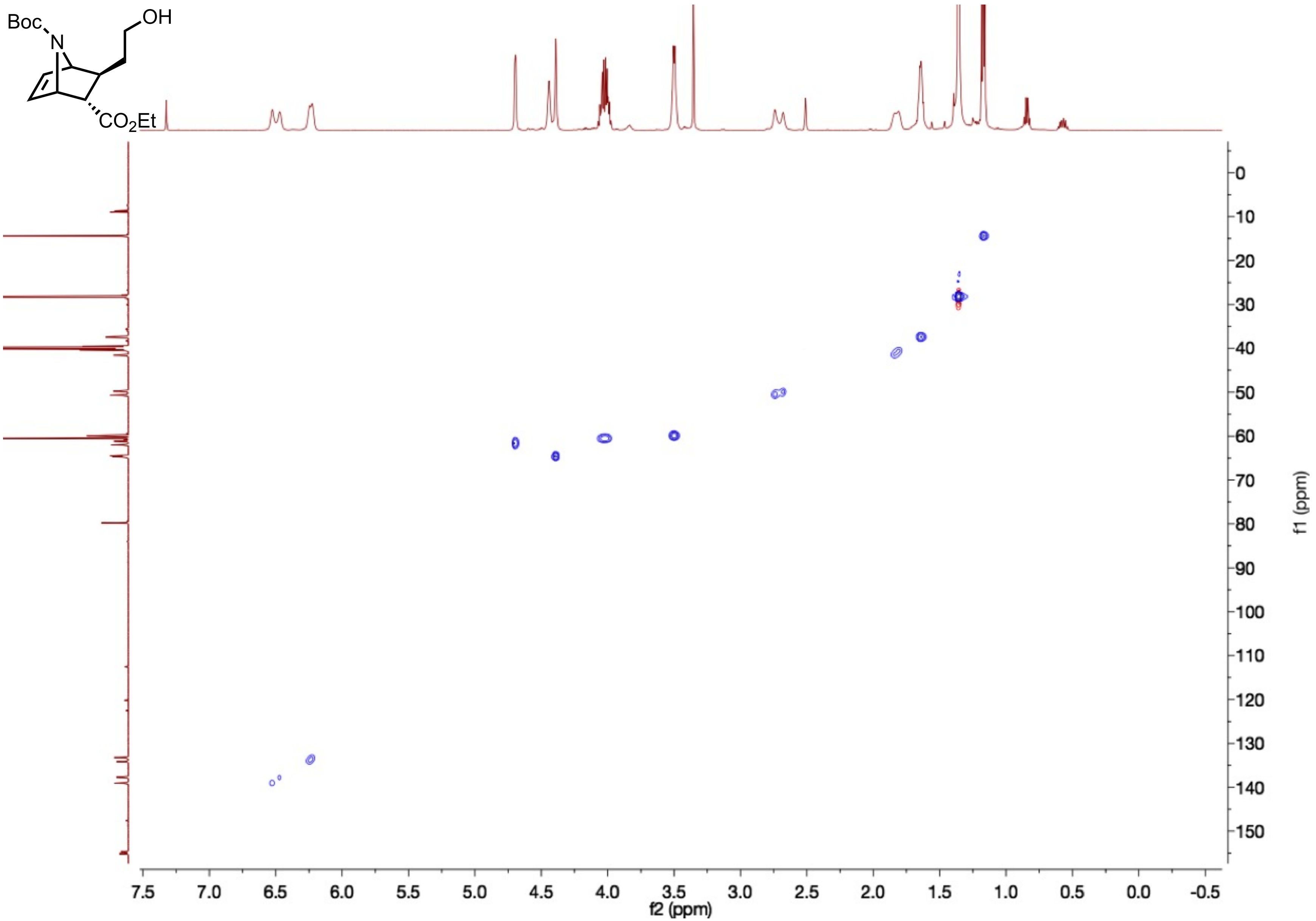

Figure S10. HSQC (DMSO-d_6, 27 °C) Primary Alcohol **16**

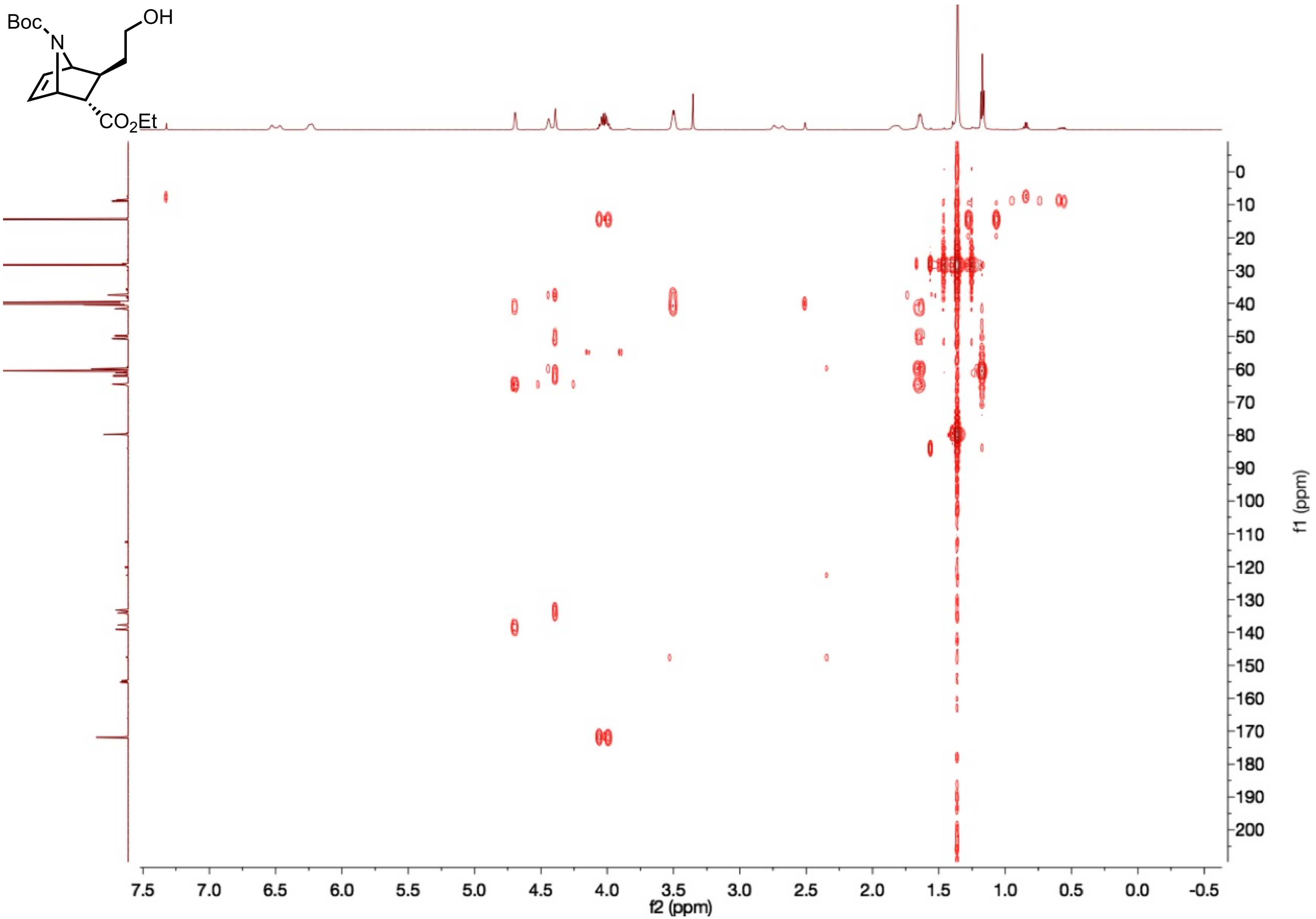

Figure S11. HMBC (DMSO-d_6, 27 °C) Primary Alcohol **16**

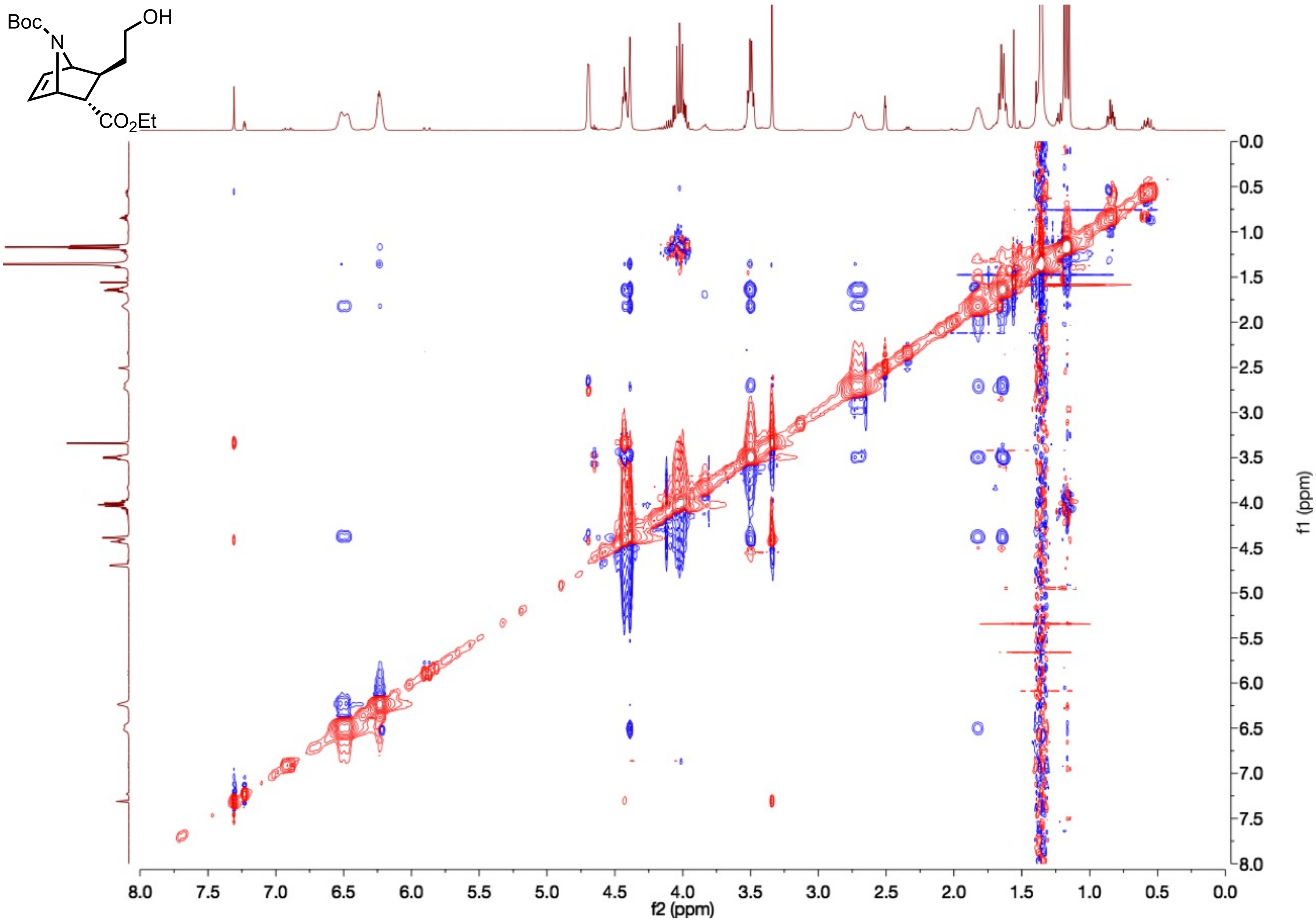

Figure S12. NOESY (DMSO-d_6, 27 °C) Primary Alcohol **16**

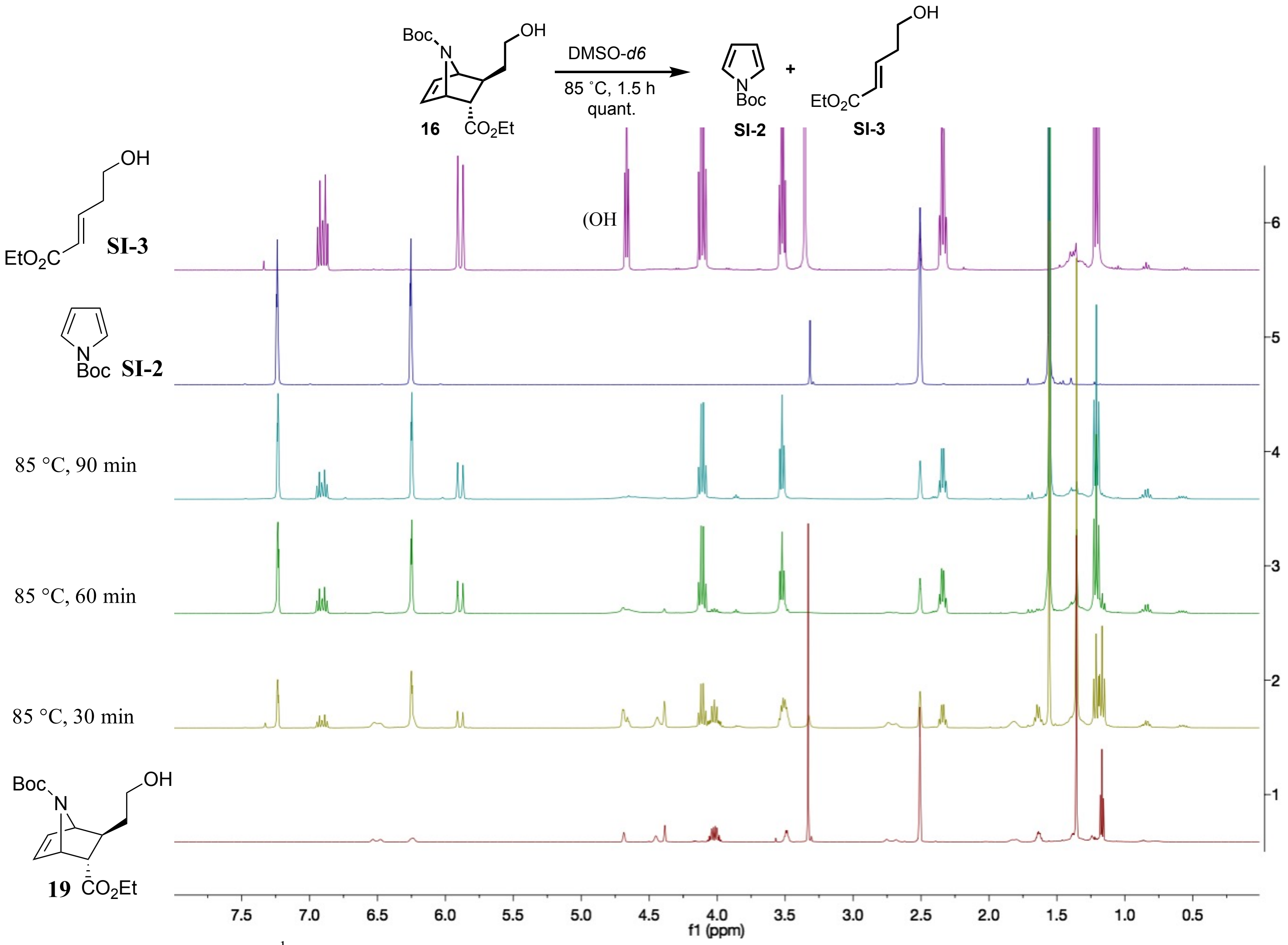

Figure S13. ^{1}H NMR (400 MHz) Monitoring of Retro Diels-Alder Reaction (DMSO-d_6, **16** → **SI-2** and **SI-3**)

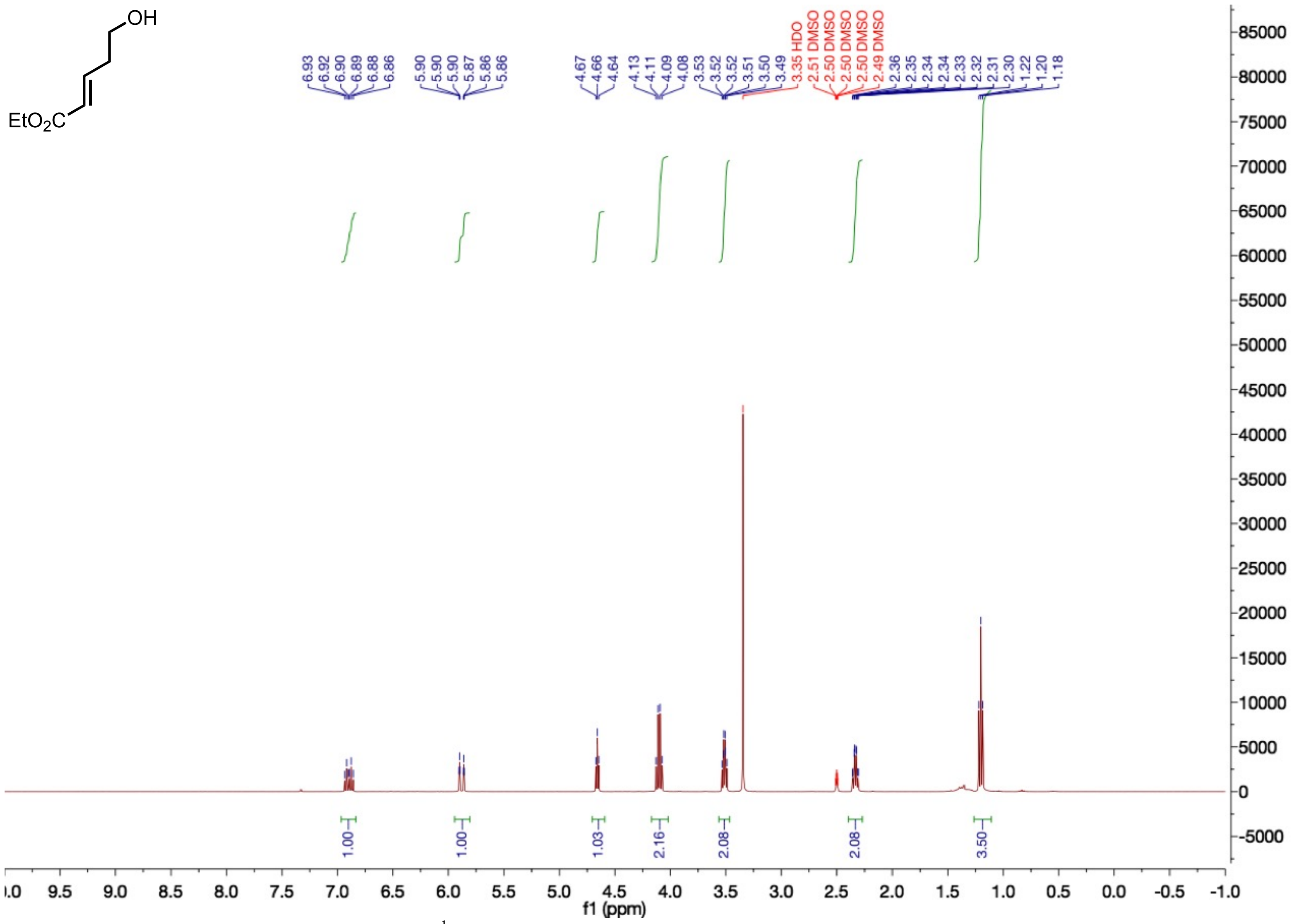

Figure S14. ^{1}H NMR (400 MHz, DMSO-d_6, 27 °C) Primary Alcohol **SI-3**

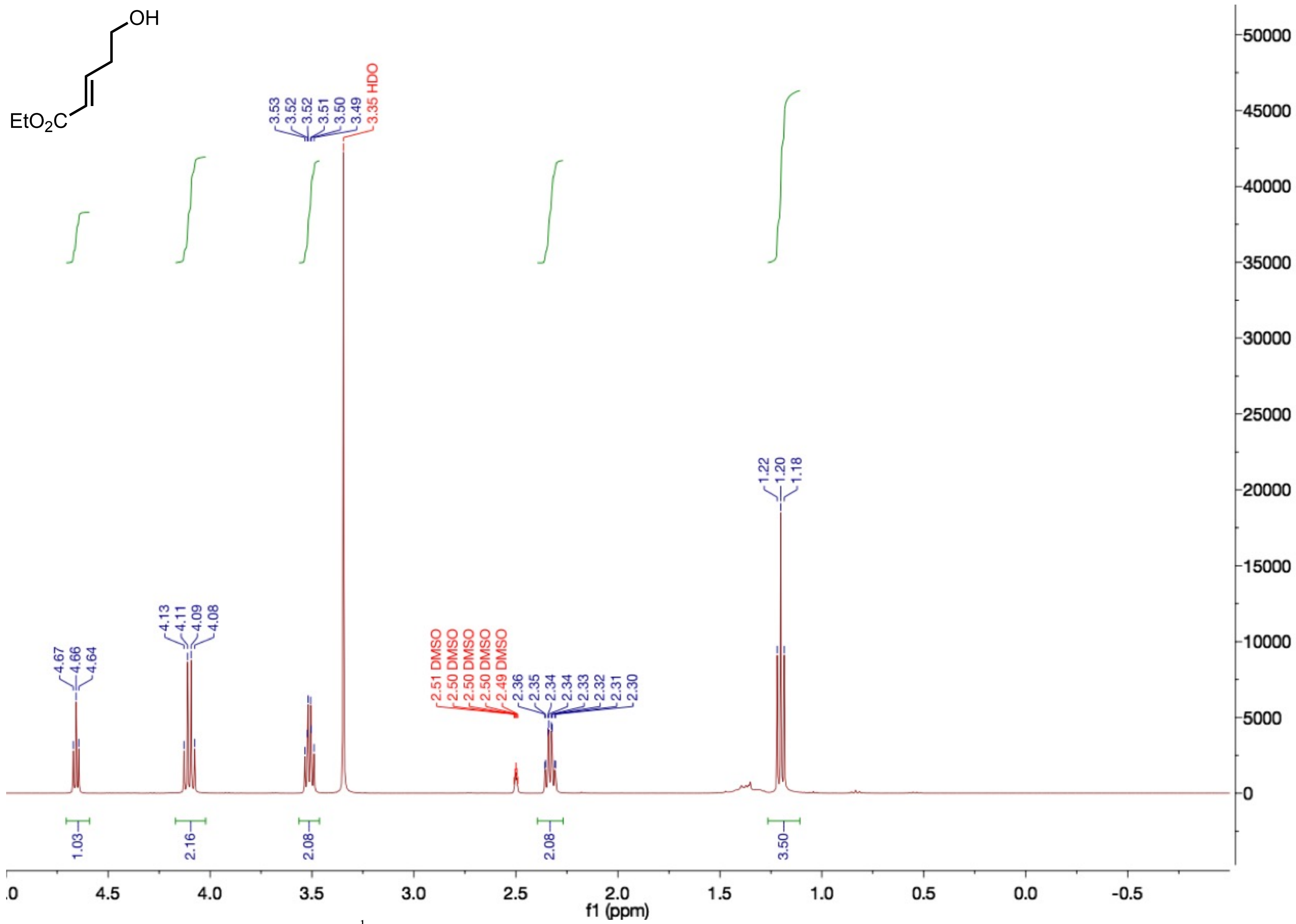

Figure S15. ^{1}H NMR (400 MHz, DMSO-d_6, 27 °C) Primary Alcohol **SI-3** (-1 – 5 ppm inset)

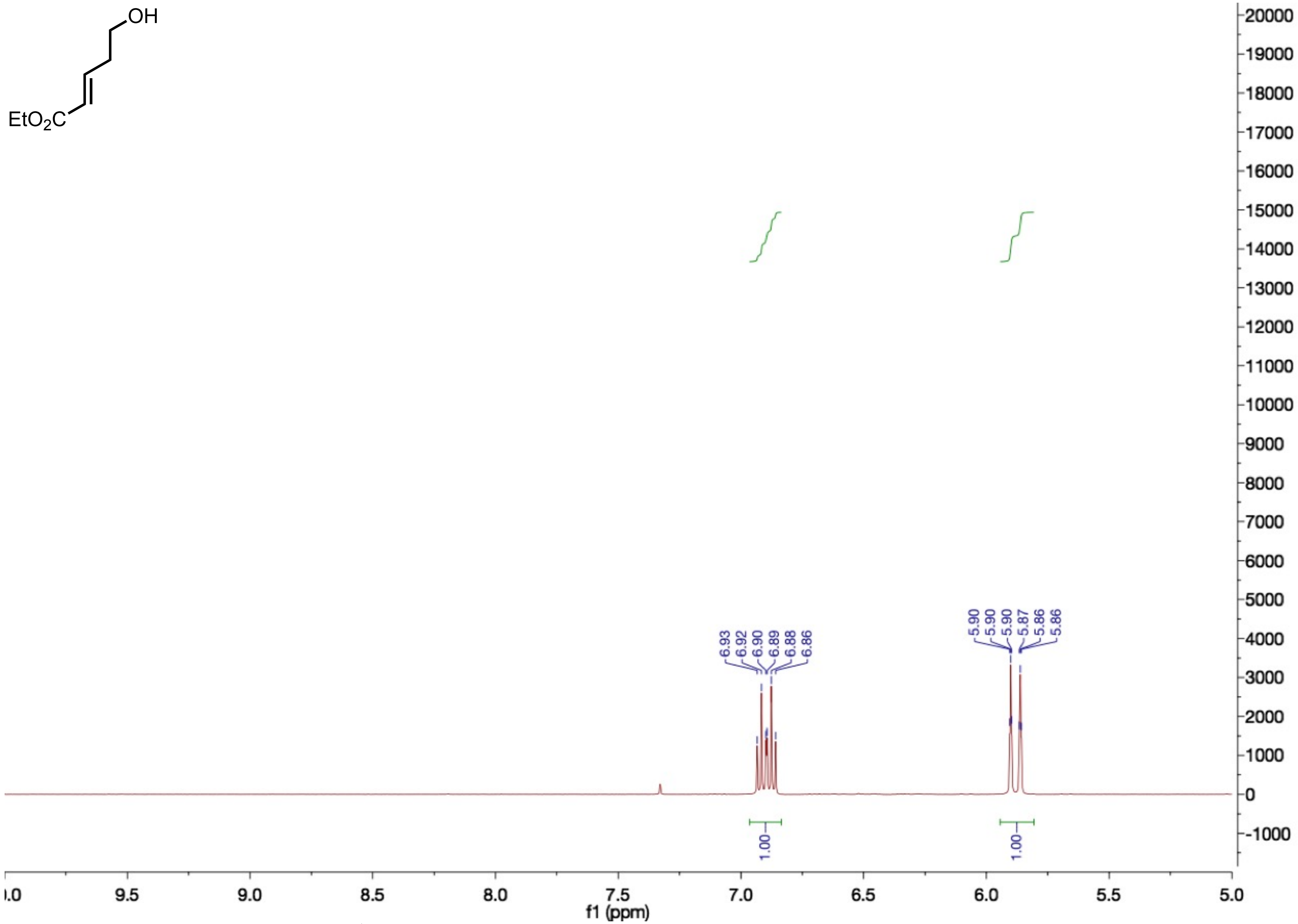

Figure S16. ^{1}H NMR (400 MHz, DMSO-d_6, 27 °C) Primary Alcohol **SI-3** (5 – 10 ppm inset)

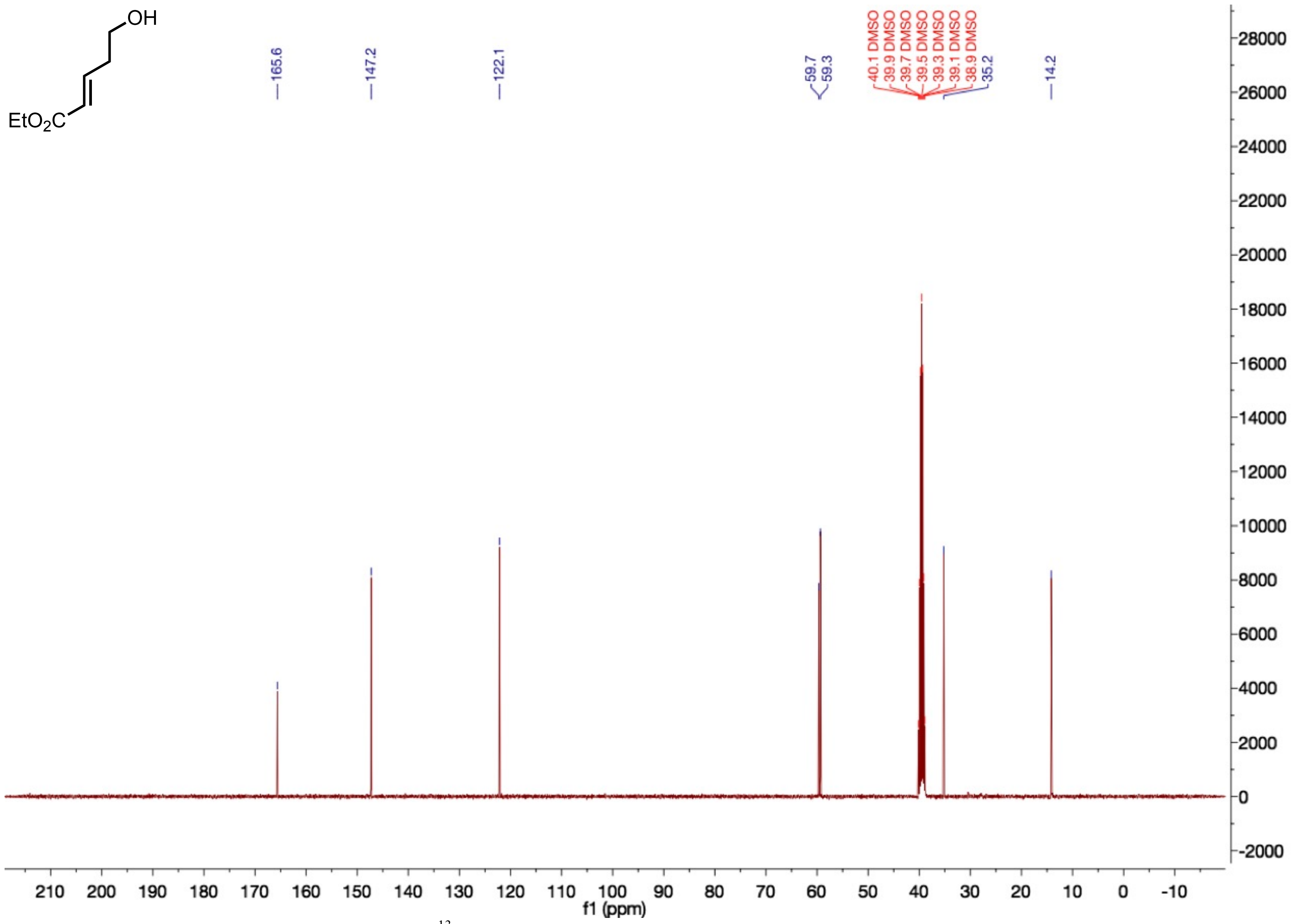

Figure S17. ^{13}C NMR (101 MHz, DMSO-d_6, 27 °C) Primary Alcohol **SI-3**

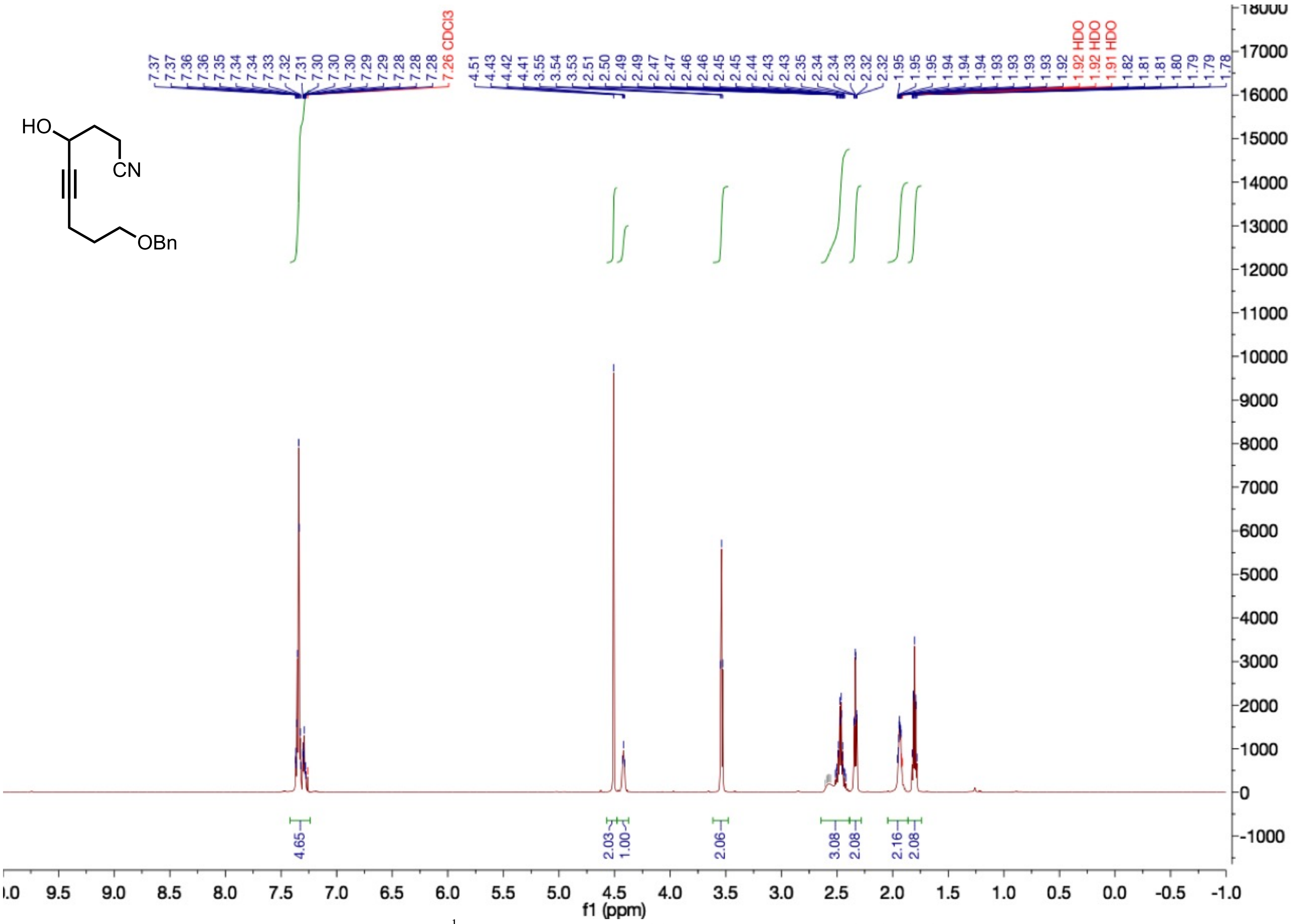

Figure S18. ^{1}H NMR (600 MHz, chloroform-*d*) Propargyl Alcohol **SI-5**

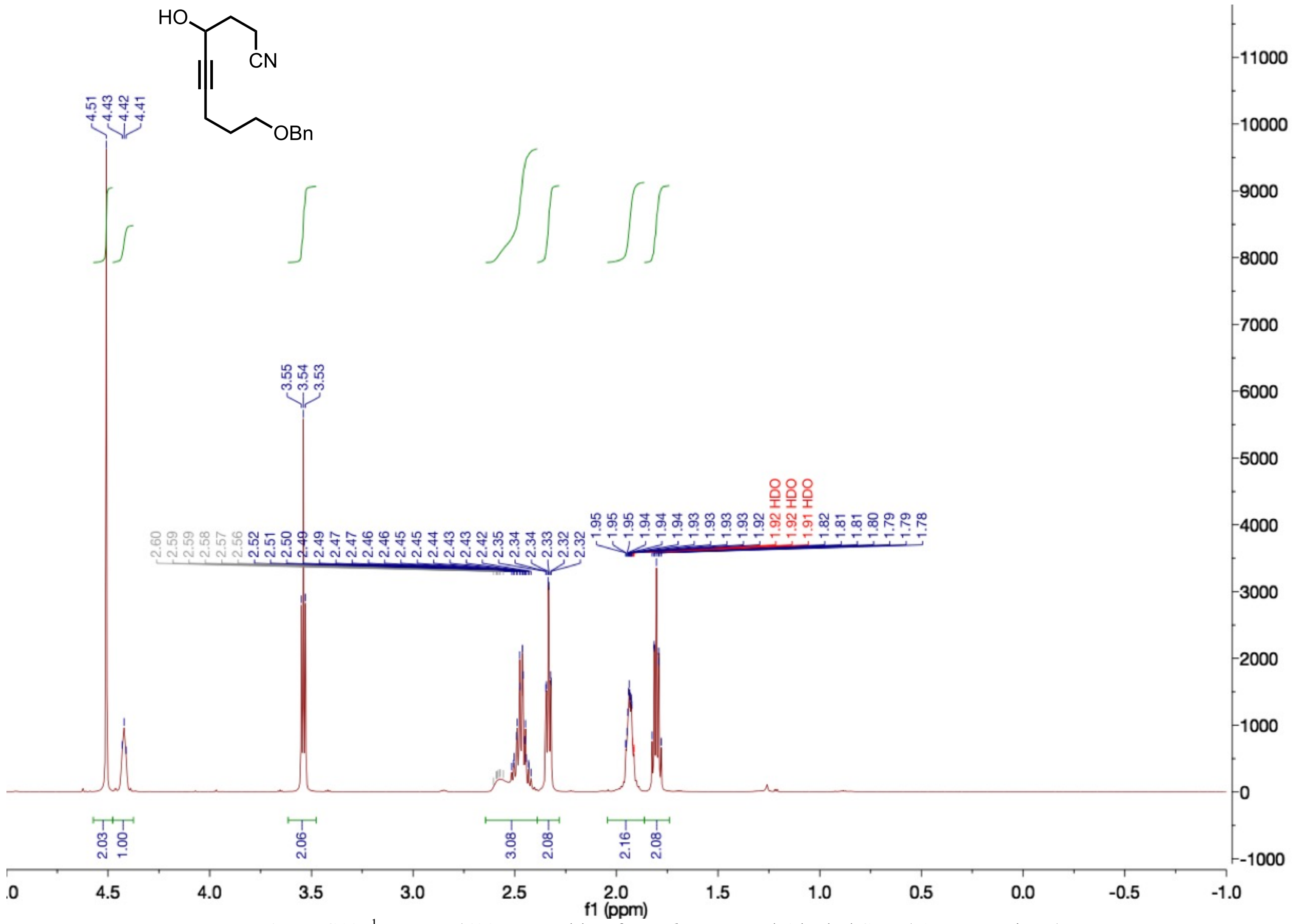

Figure S19. ^{1}H NMR (600 MHz, chloroform-*d*) Propargyl Alcohol **SI-5** (-1 – 5 ppm inset)

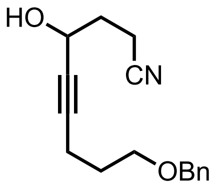

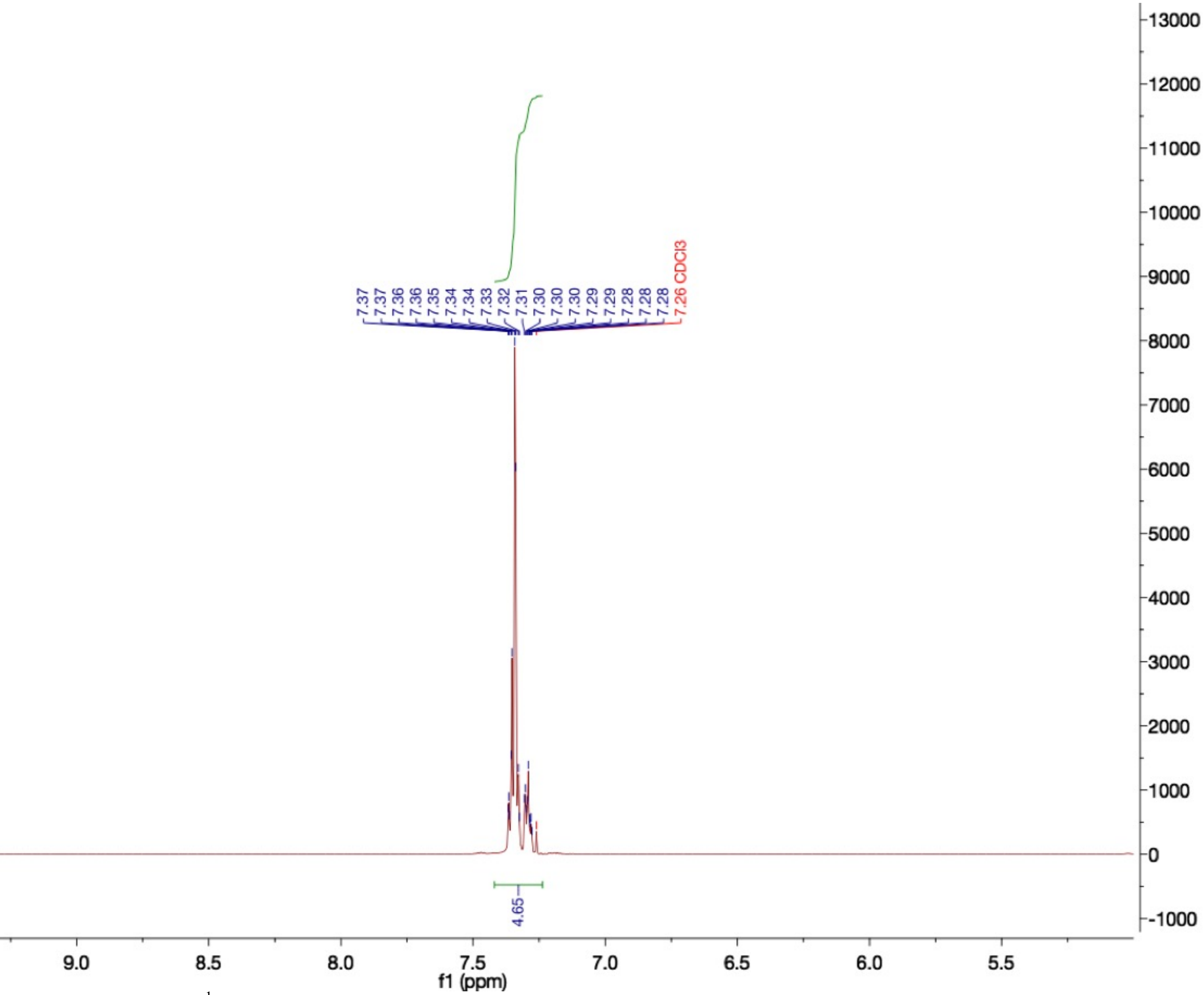

Figure S20. ^{1}H NMR (600 MHz, chloroform-*d*) Propargyl Alcohol **SI-5** (5 – 10 ppm inset)

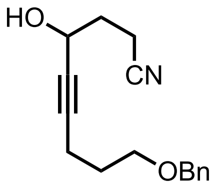

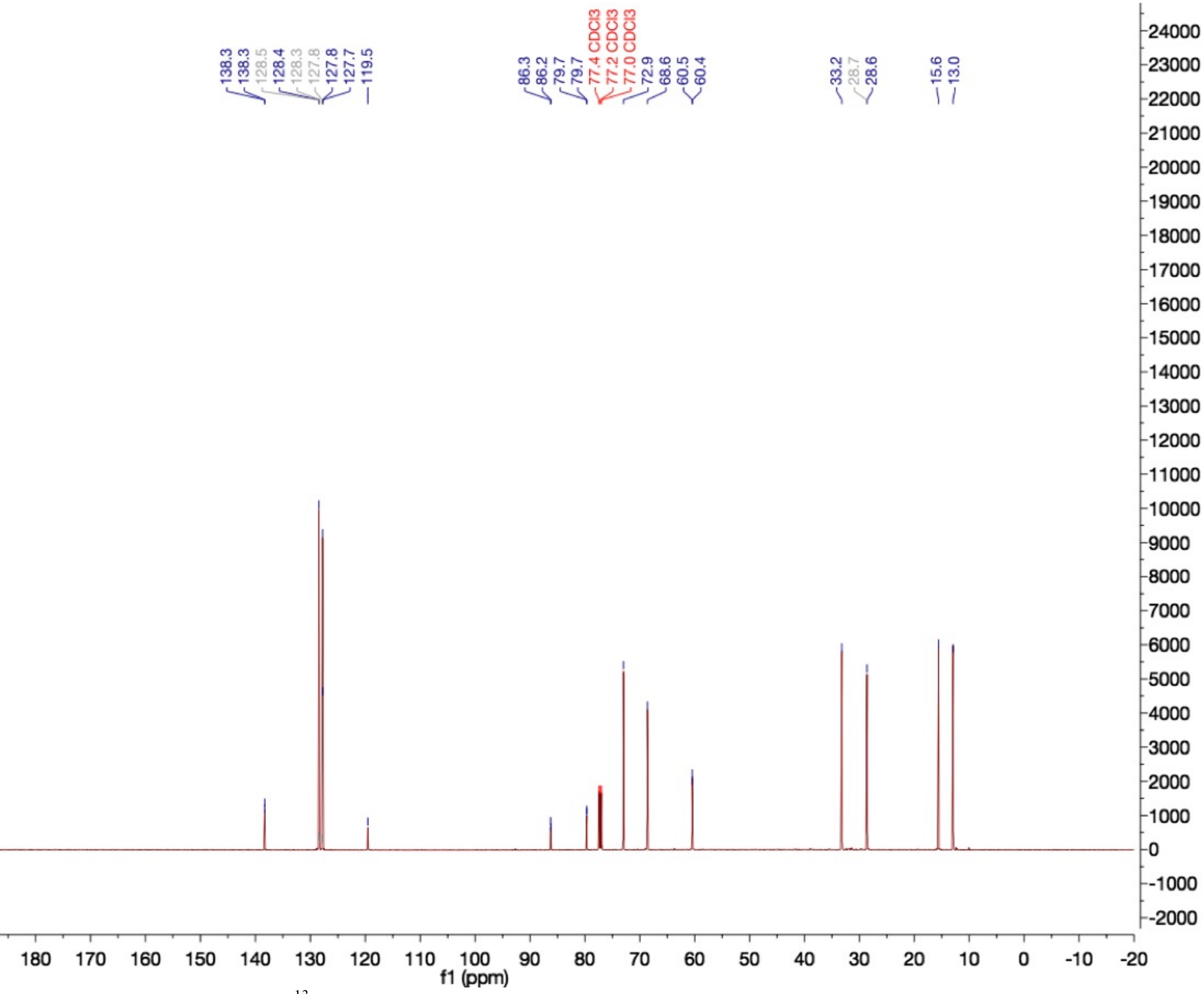

Figure S21. ^{13}C NMR (151 MHz, chloroform-*d*) Propargyl Alcohol **SI-5**

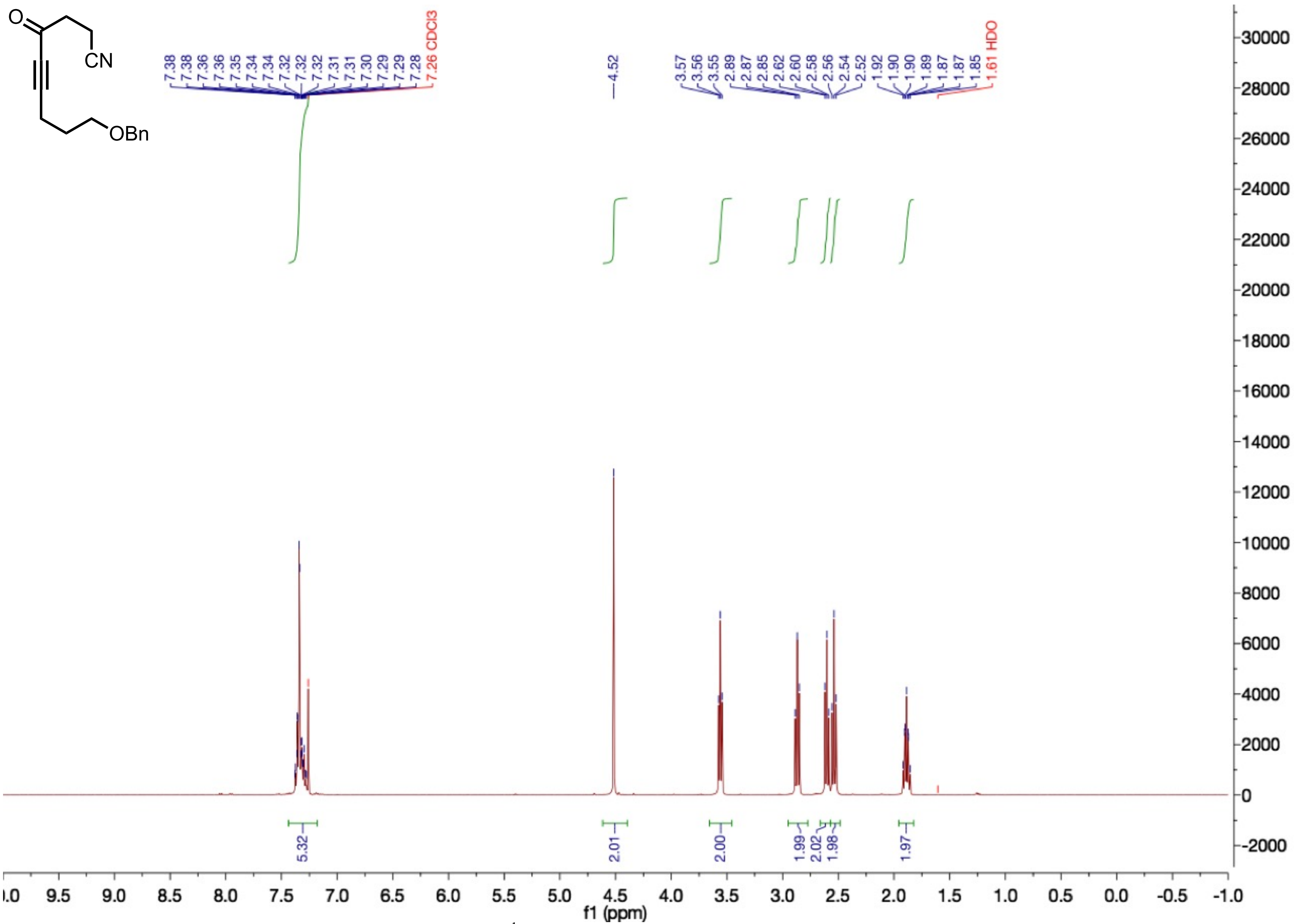

Figure S22. ^{1}H NMR (400 MHz, chloroform-*d*) Ketone **31**

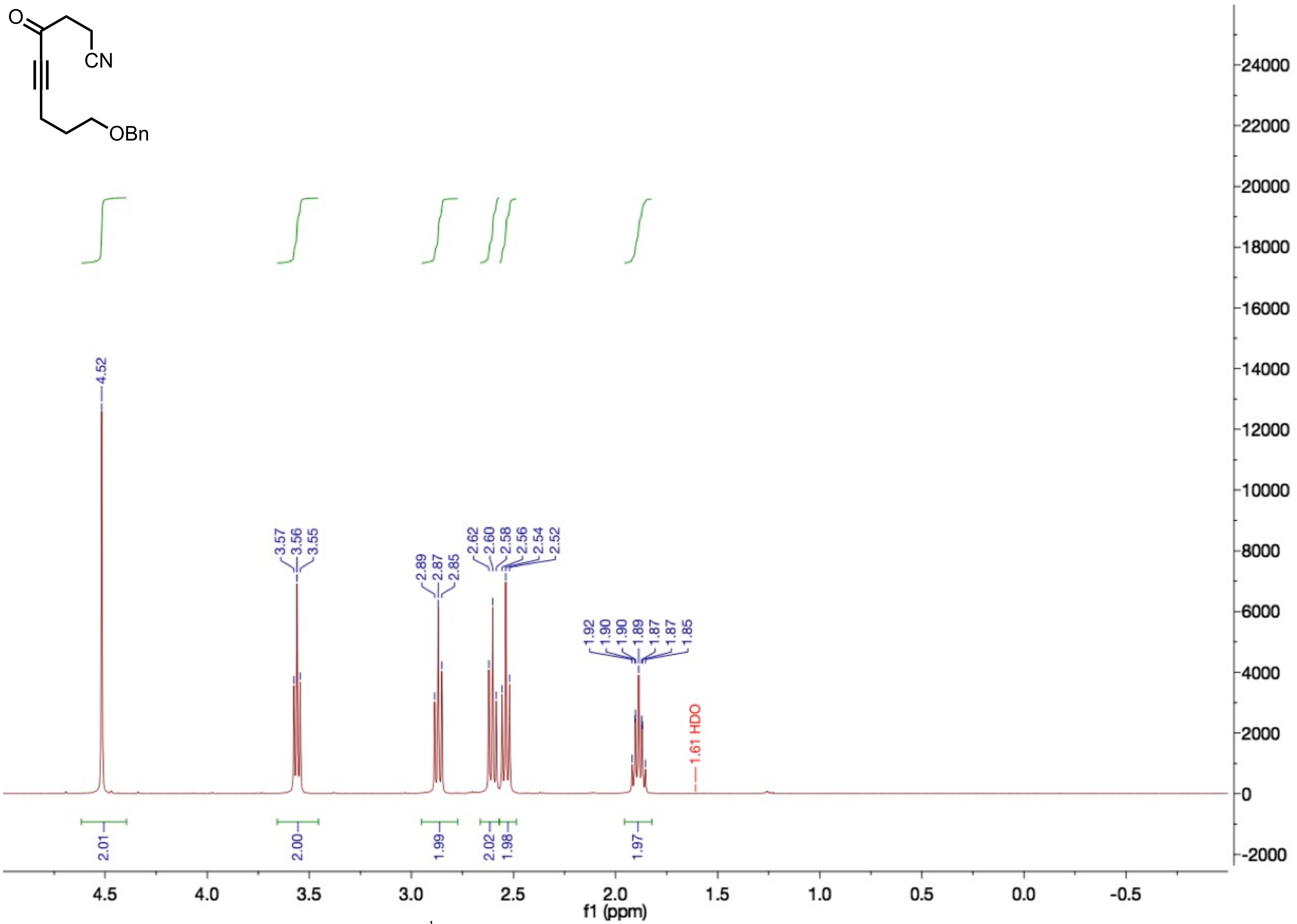

Figure S23. ^{1}H NMR (400 MHz, chloroform-*d*) Ketone **31** (-1 – 5 ppm inset)

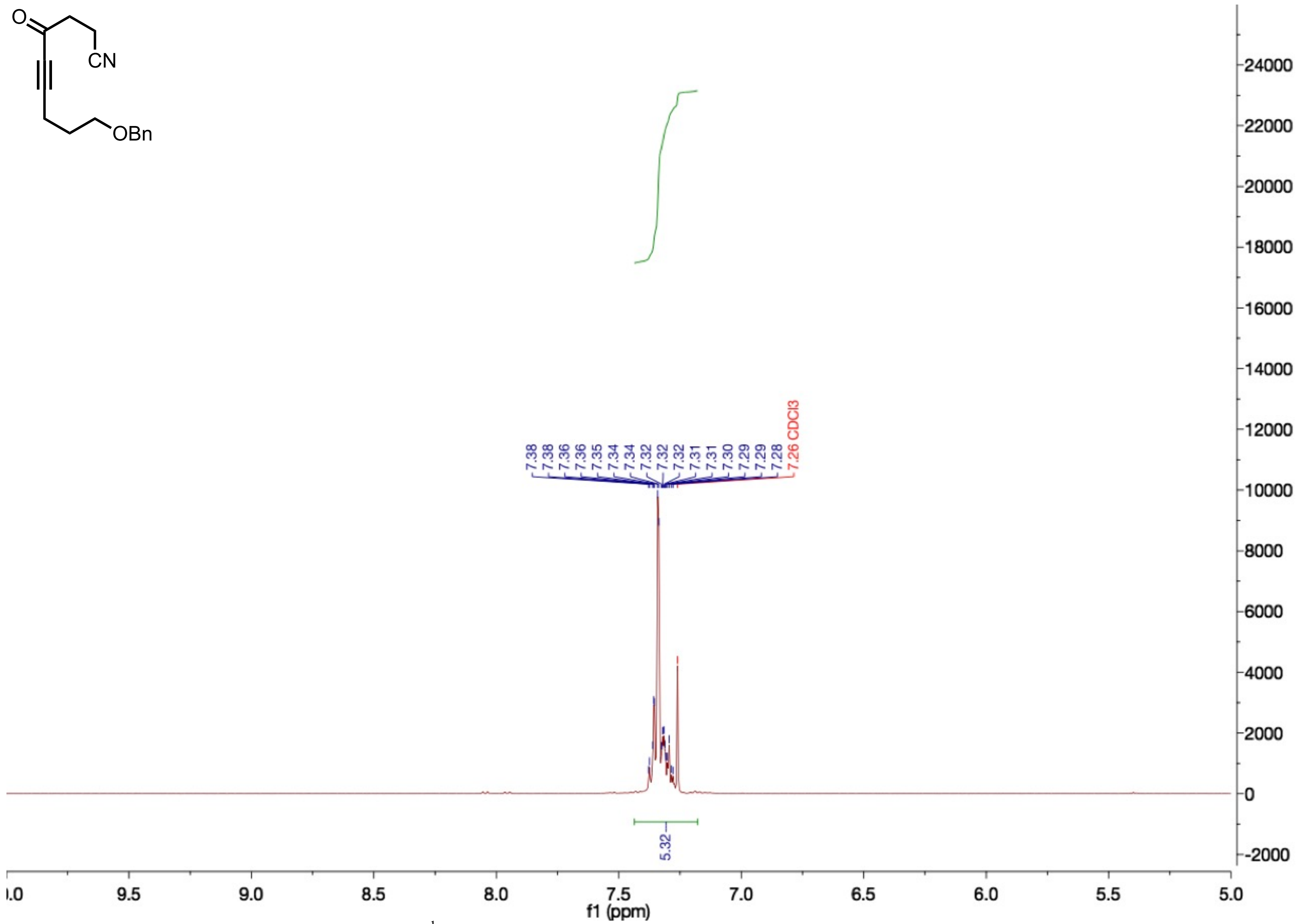

Figure S24. ^{1}H NMR (400 MHz, chloroform-*d*) Ketone **31** (5 – 10 ppm inset)

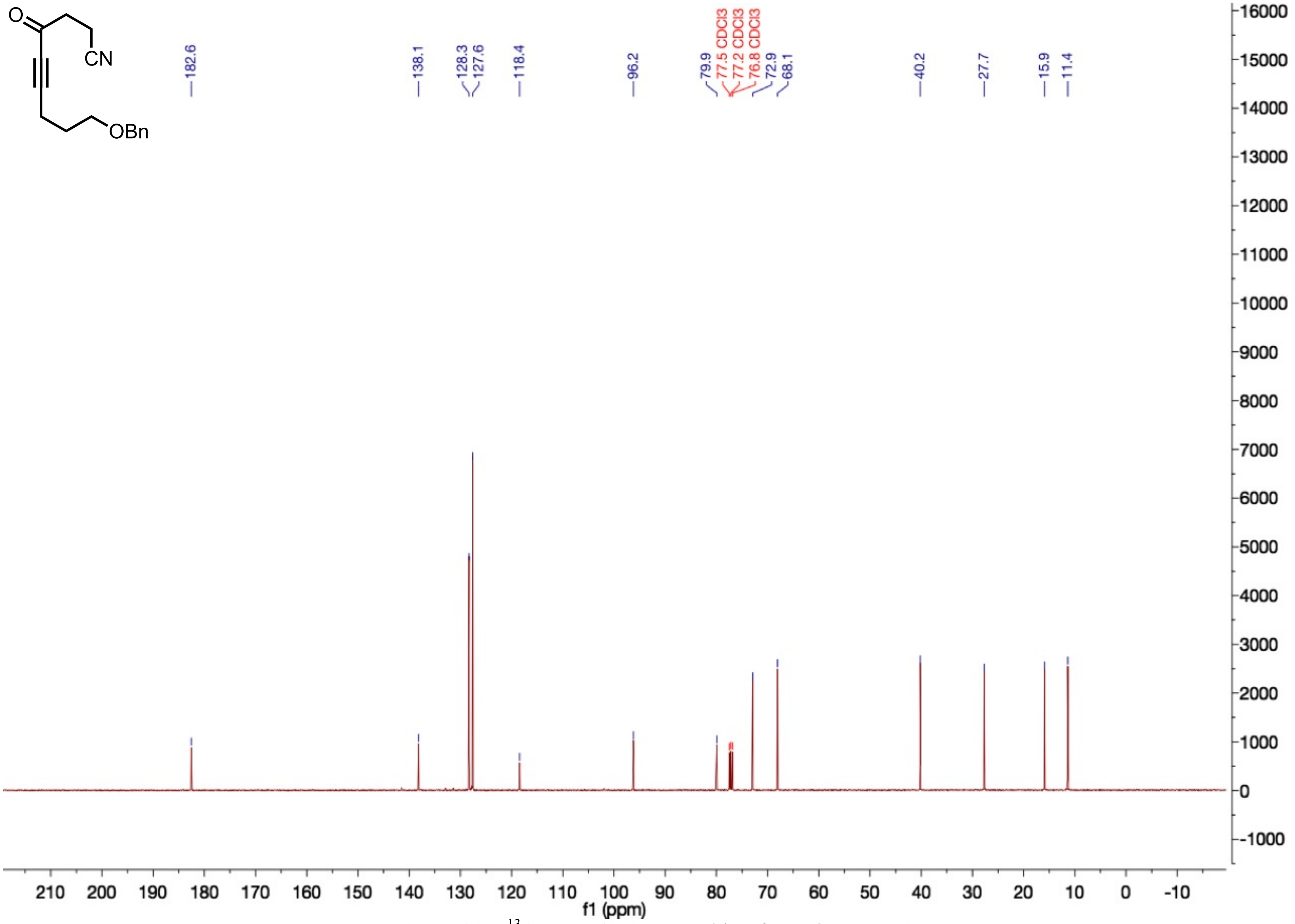

Figure S25. ^{13}C NMR (101 MHz, chloroform-*d*) Ketone **31**

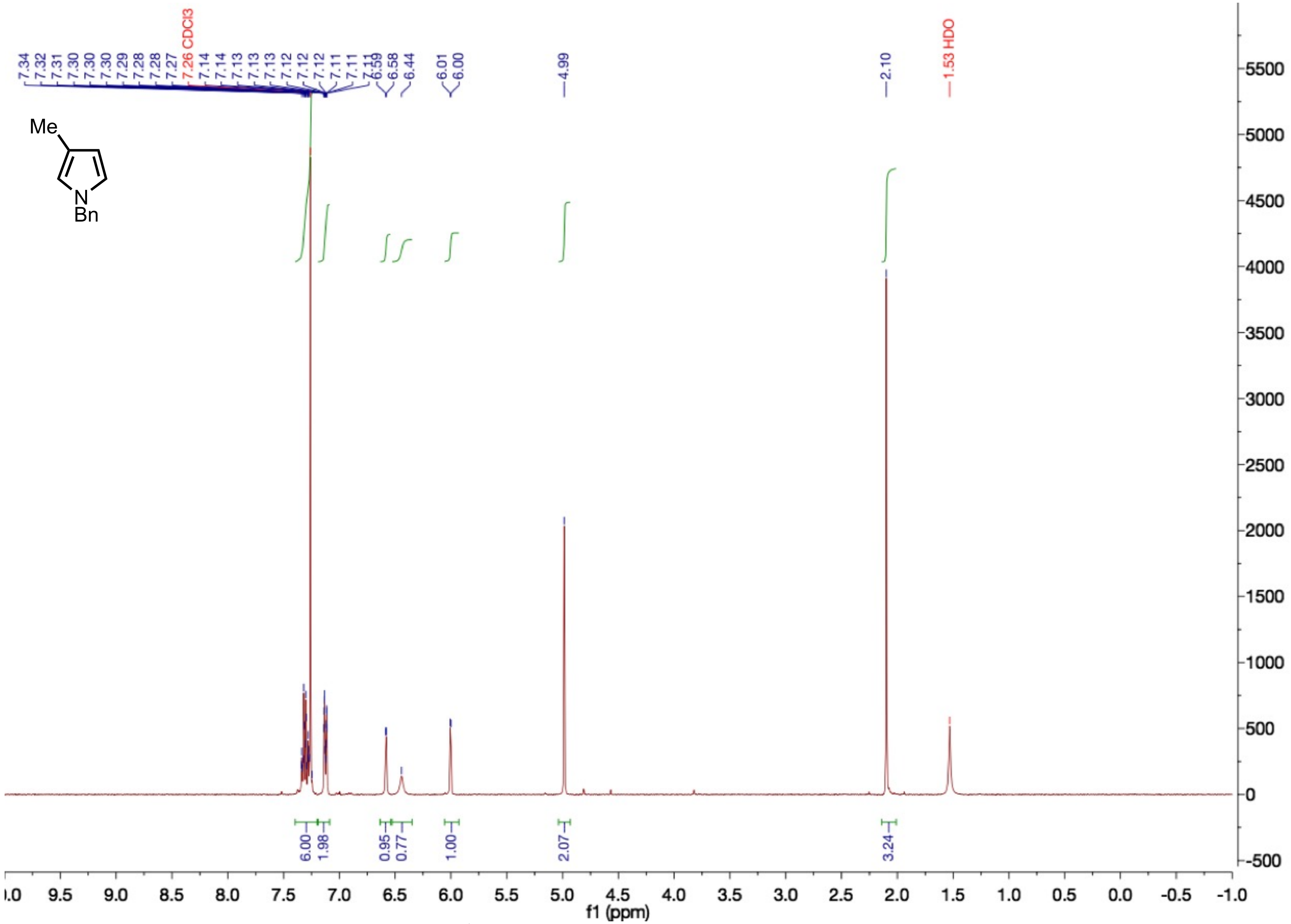

Figure S26. ^{1}H NMR (400 MHz, chloroform-*d*) *N*-Benzyl Pyrrole **35**

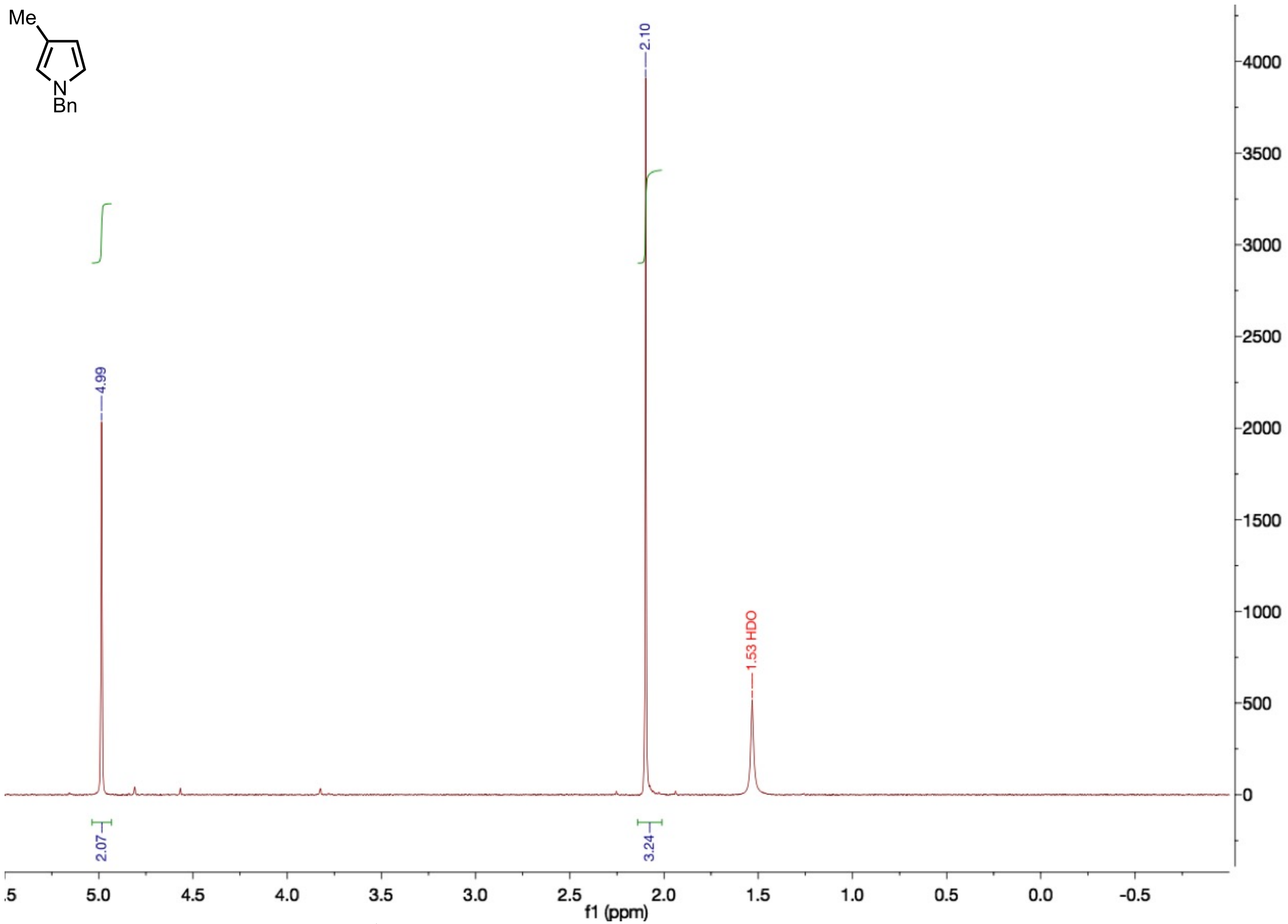

Figure S27. ^{1}H NMR (400 MHz, chloroform-*d*) *N*-Benzyl Pyrrole **35** (-1 – 5.5 ppm inset)

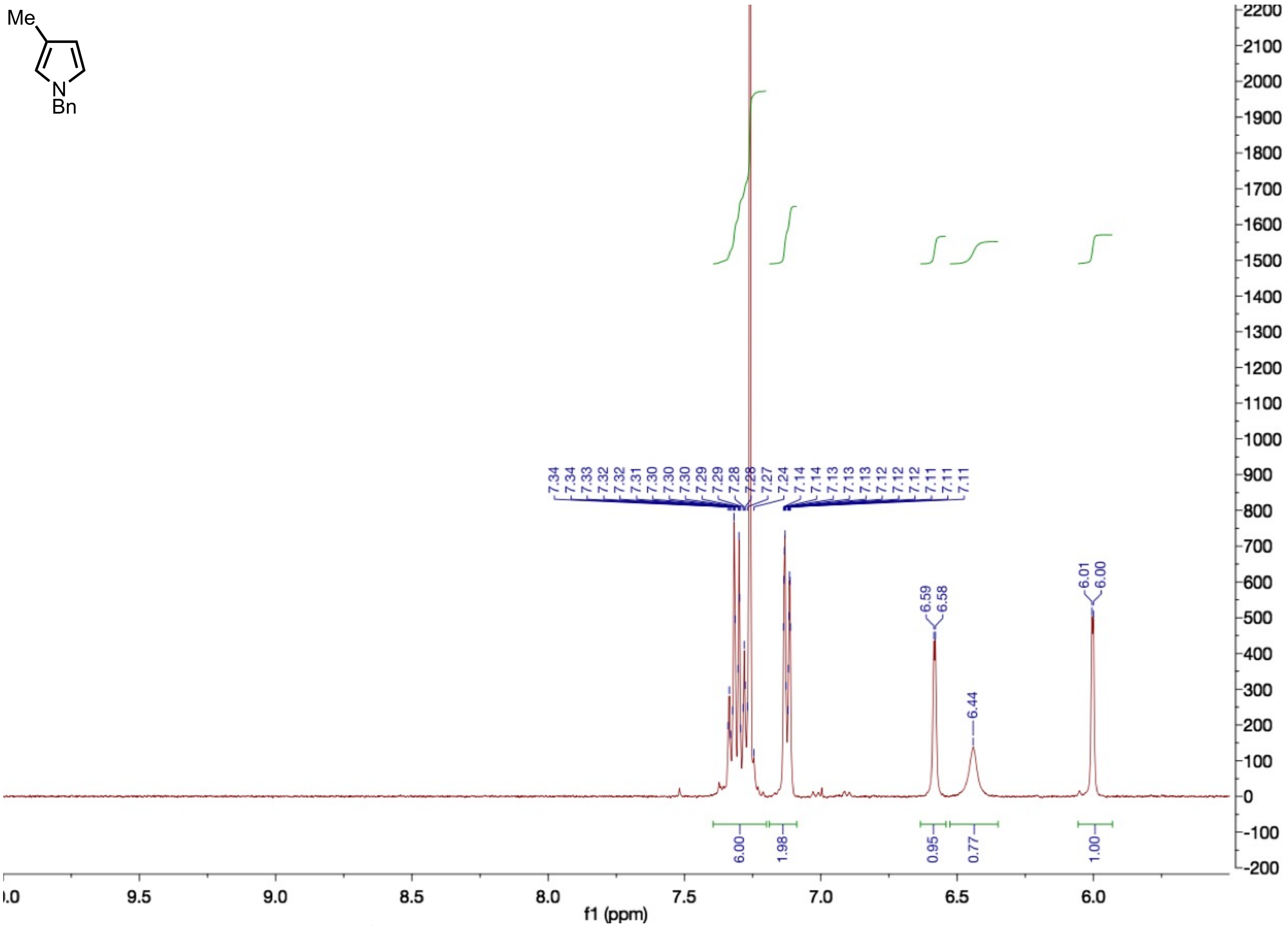

Figure S28. ^{1}H NMR (400 MHz, chloroform-*d*) *N*-Benzyl Pyrrole **35** (5.5 – 10 ppm inset)

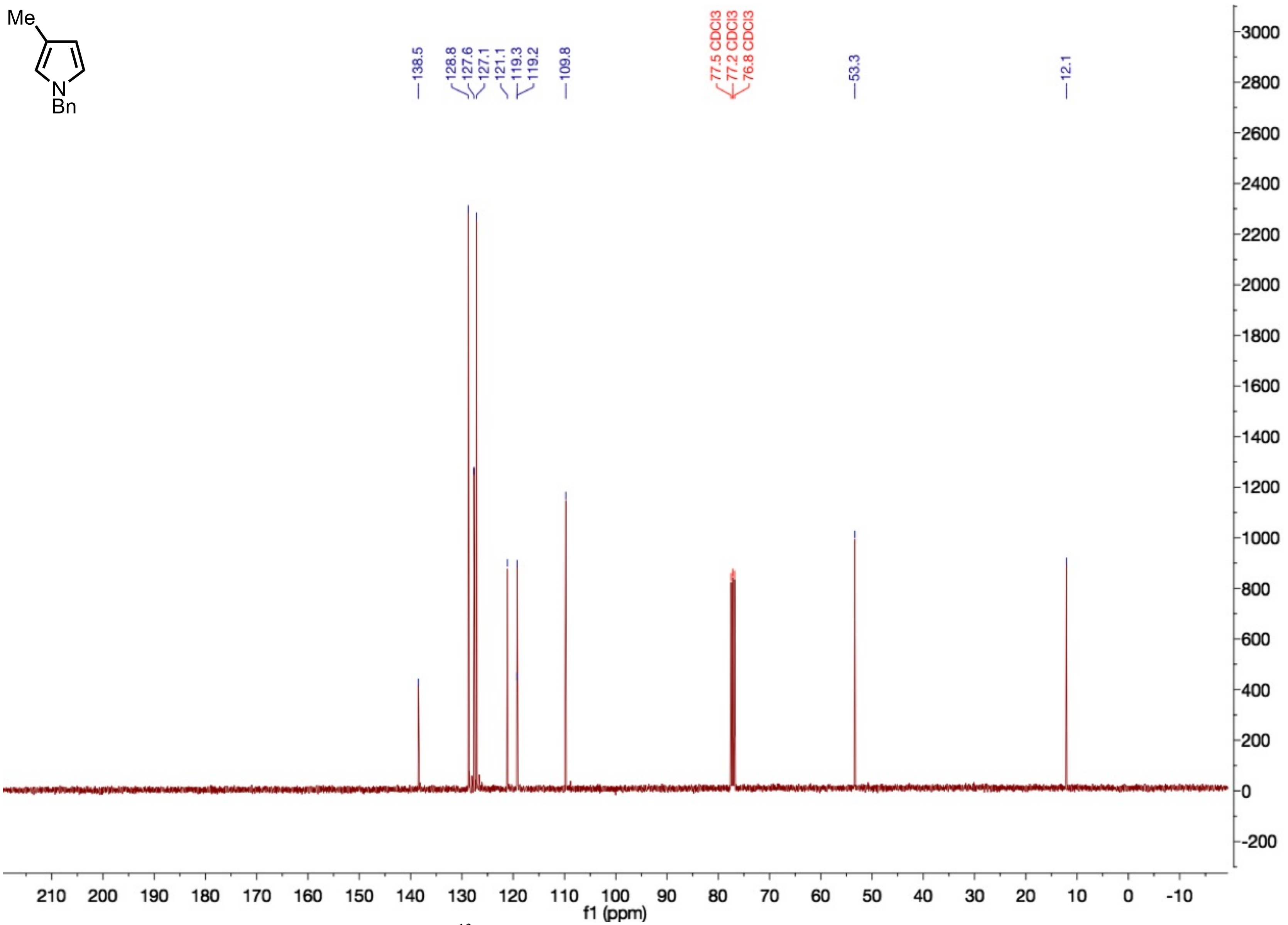

Figure S29. ^{13}C NMR (101 MHz, chloroform-*d*) *N*-Benzyl Pyrrole **35**

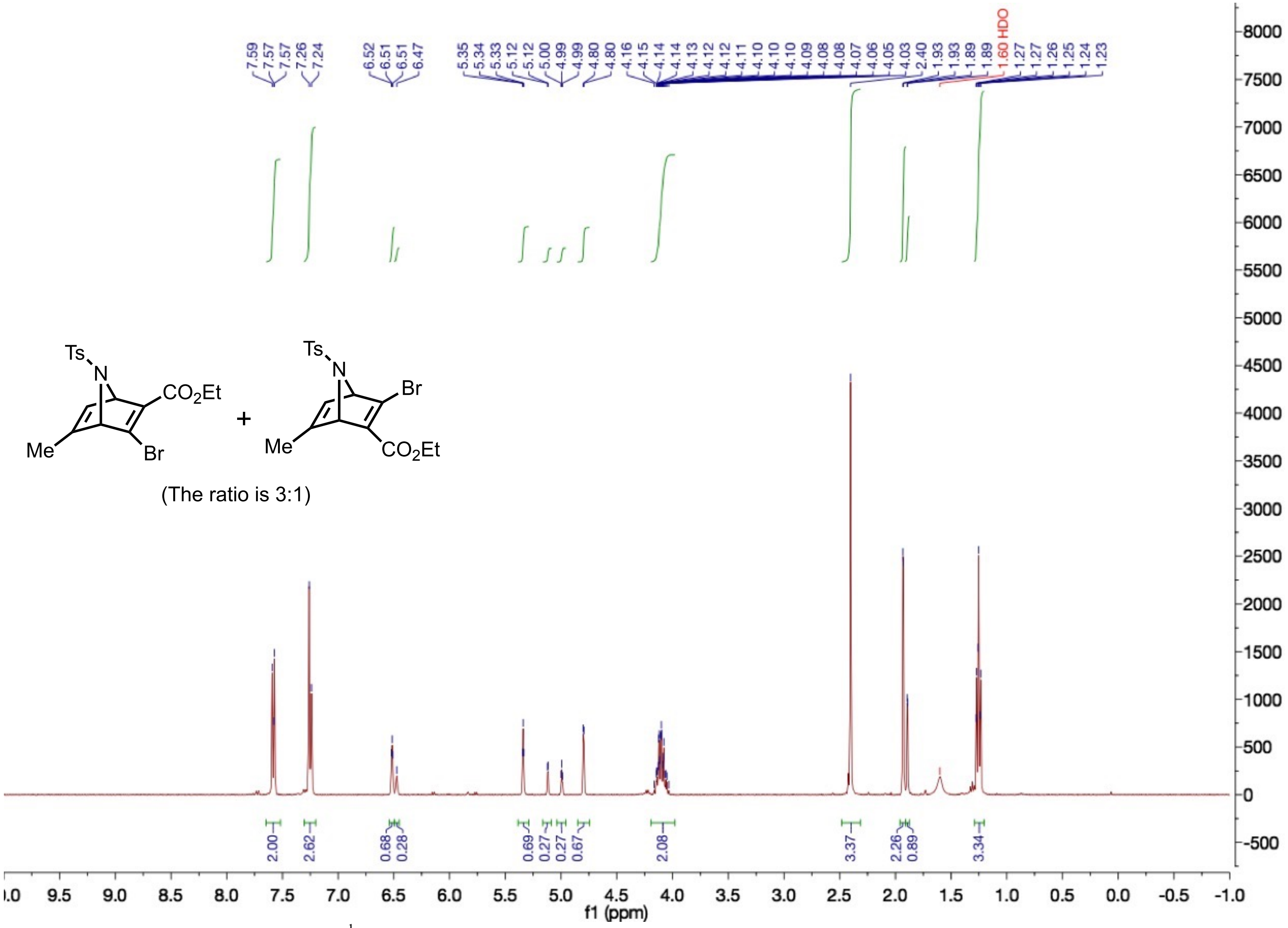

Figure S30. ^{1}H NMR (400 MHz, chloroform-*d*) *N*-Tosyl Bicyclic Esters ***N-Ts*-38** and ***N-Ts*-39**

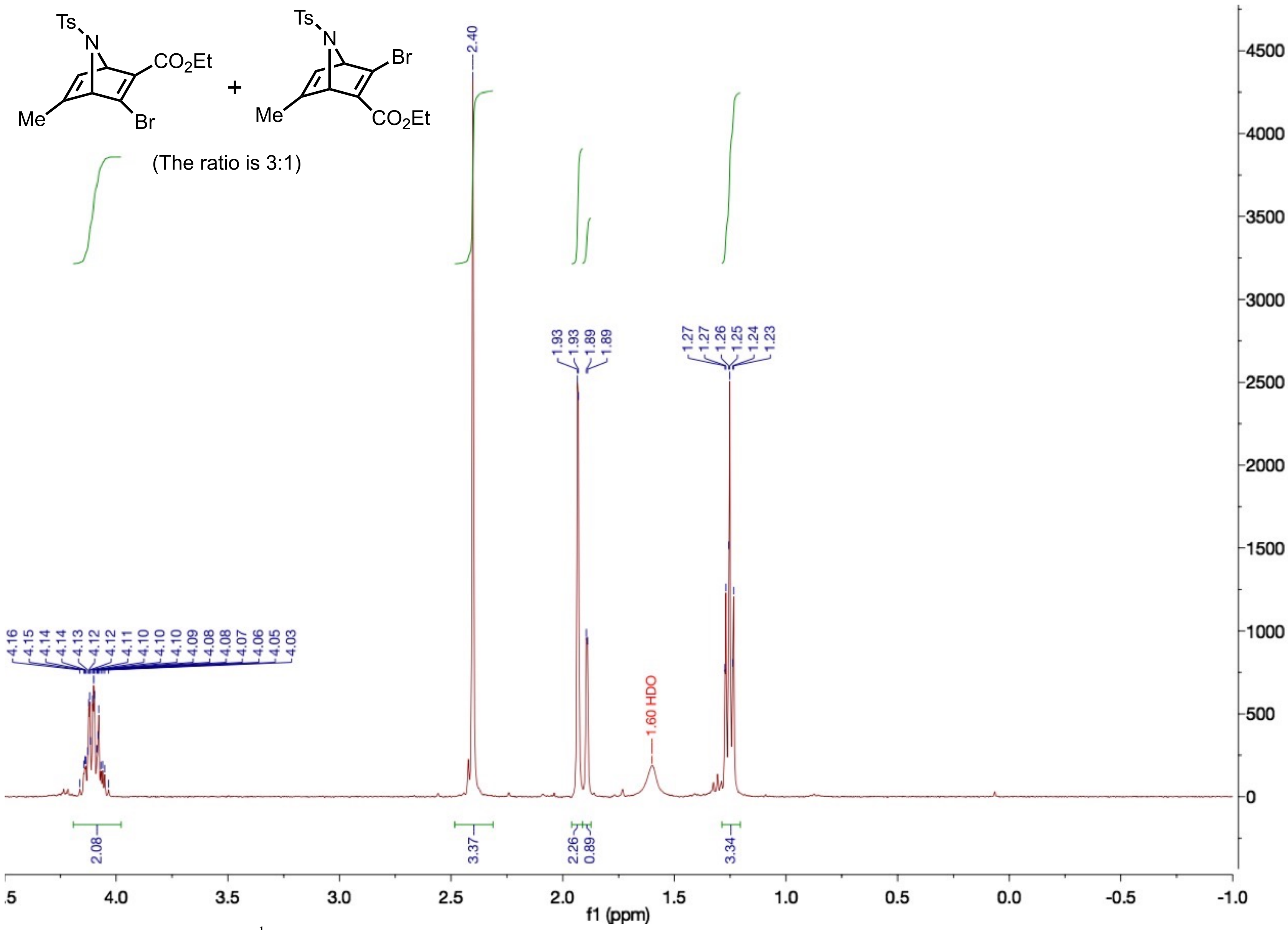

Figure S31. ^{1}H NMR (400 MHz, chloroform-*d*) *N*-Tosyl Bicyclic Esters ***N-Ts*-38** and ***N-Ts*-39** (-1 – 4.5 ppm inset)

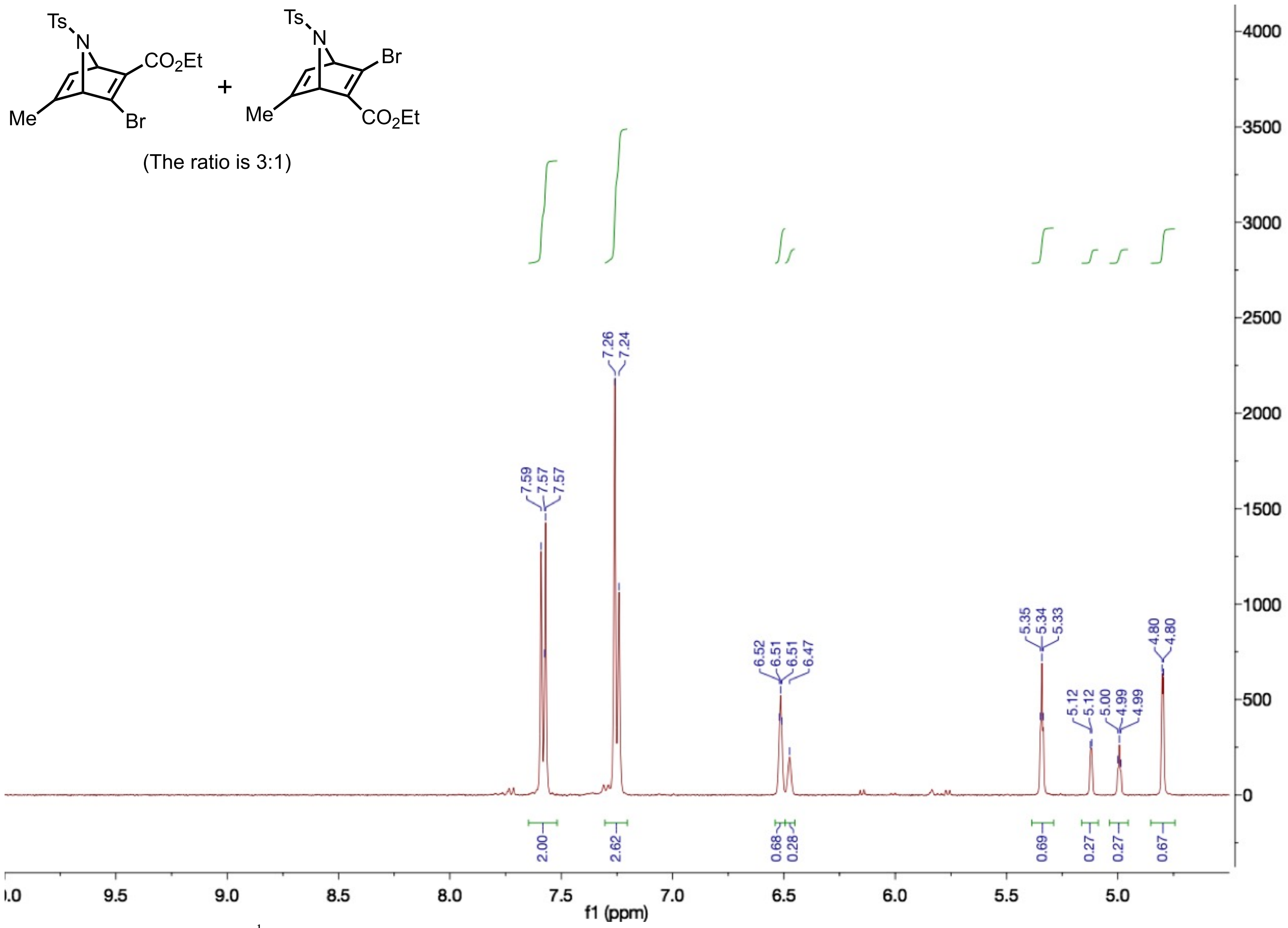

Figure S32. ^{1}H NMR (400 MHz, chloroform-*d*) *N*-Tosyl Bicyclic Esters ***N*-*Ts*-38** and ***N*-*Ts*-39** (4.5 – 10 ppm inset)

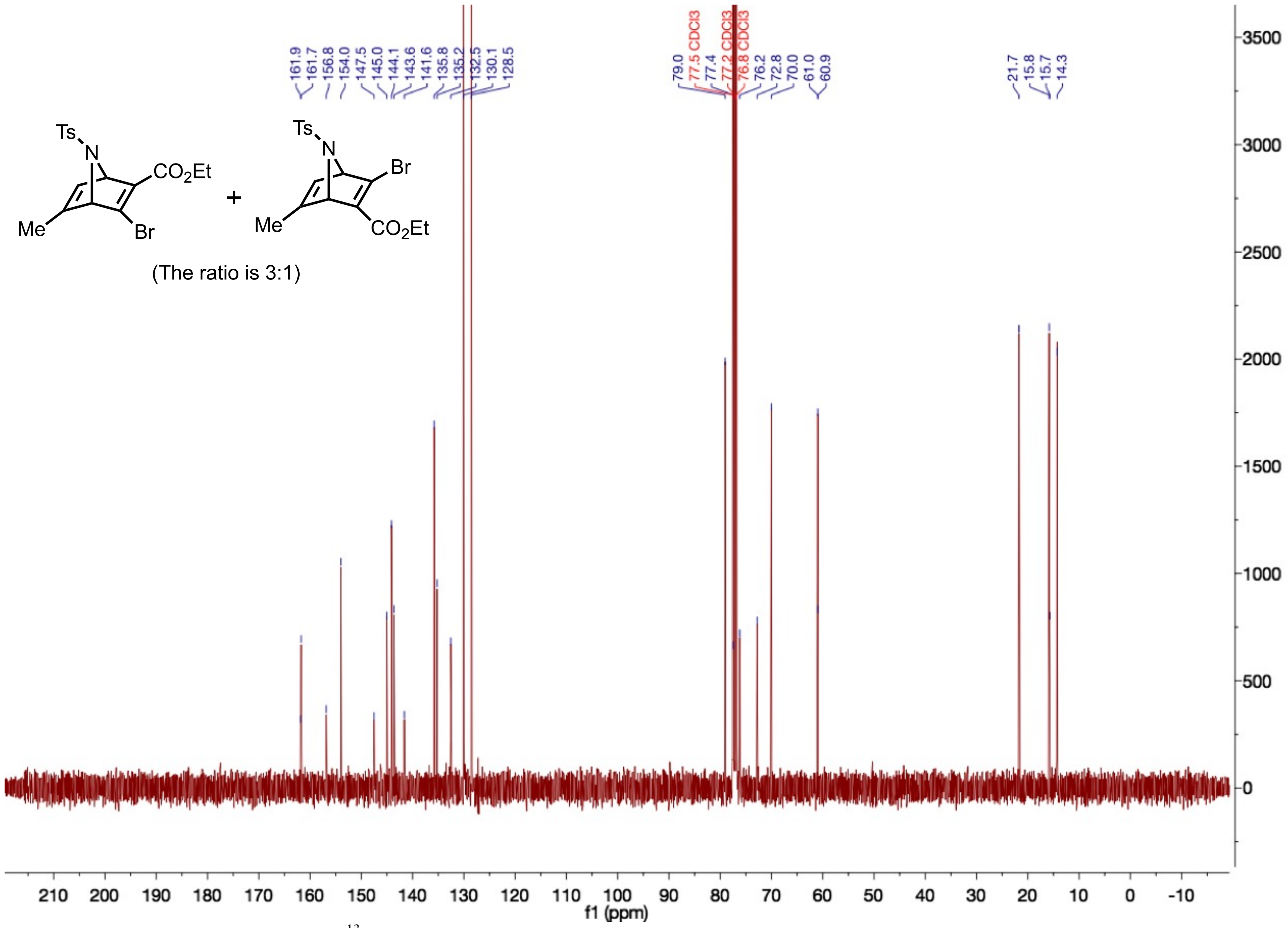

Figure S33. ^{13}C NMR (101 MHz, chloroform-*d*) *N*-Tosyl Bicyclic Esters ***N-Ts*-38** and ***N-Ts*-39**

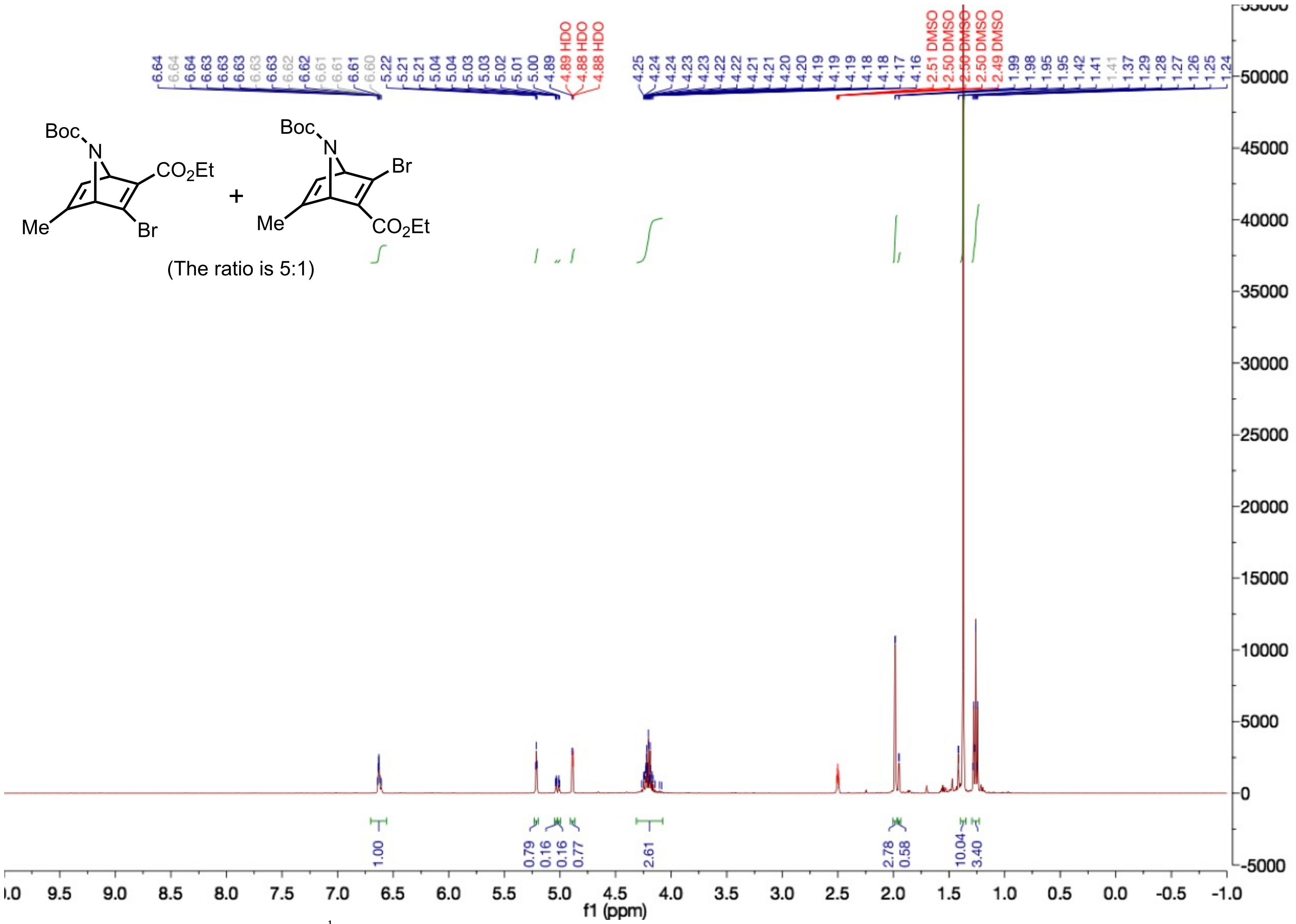

Figure S34. ^{1}H NMR (400 MHz, DMSO-d_6, 85 °C) *N*-Boc Bicyclic Esters ***N*-Boc-38** and ***N*-Boc-39**

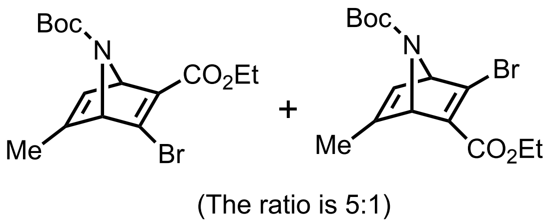

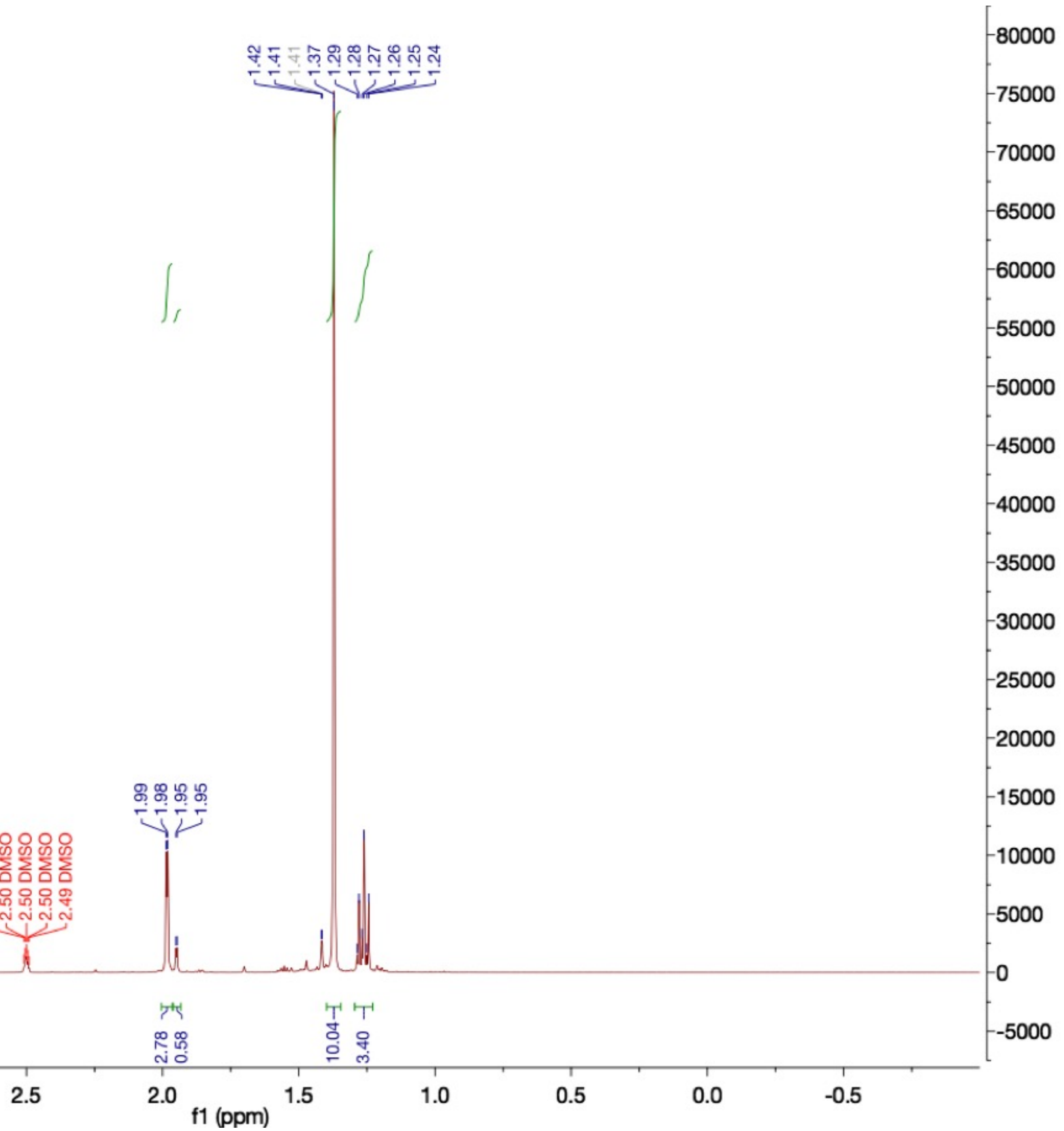

Figure S35. ^{1}H NMR (400 MHz, DMSO-d_6, 85 °C) *N*-Boc Bicyclic Esters ***N*-Boc-38** and ***N*-Boc-39** (-1 – 4.5 ppm inset)

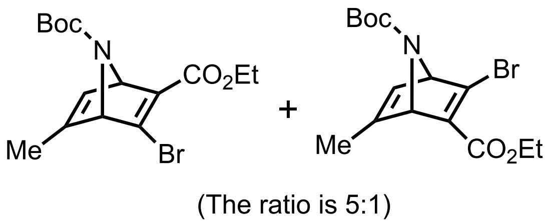

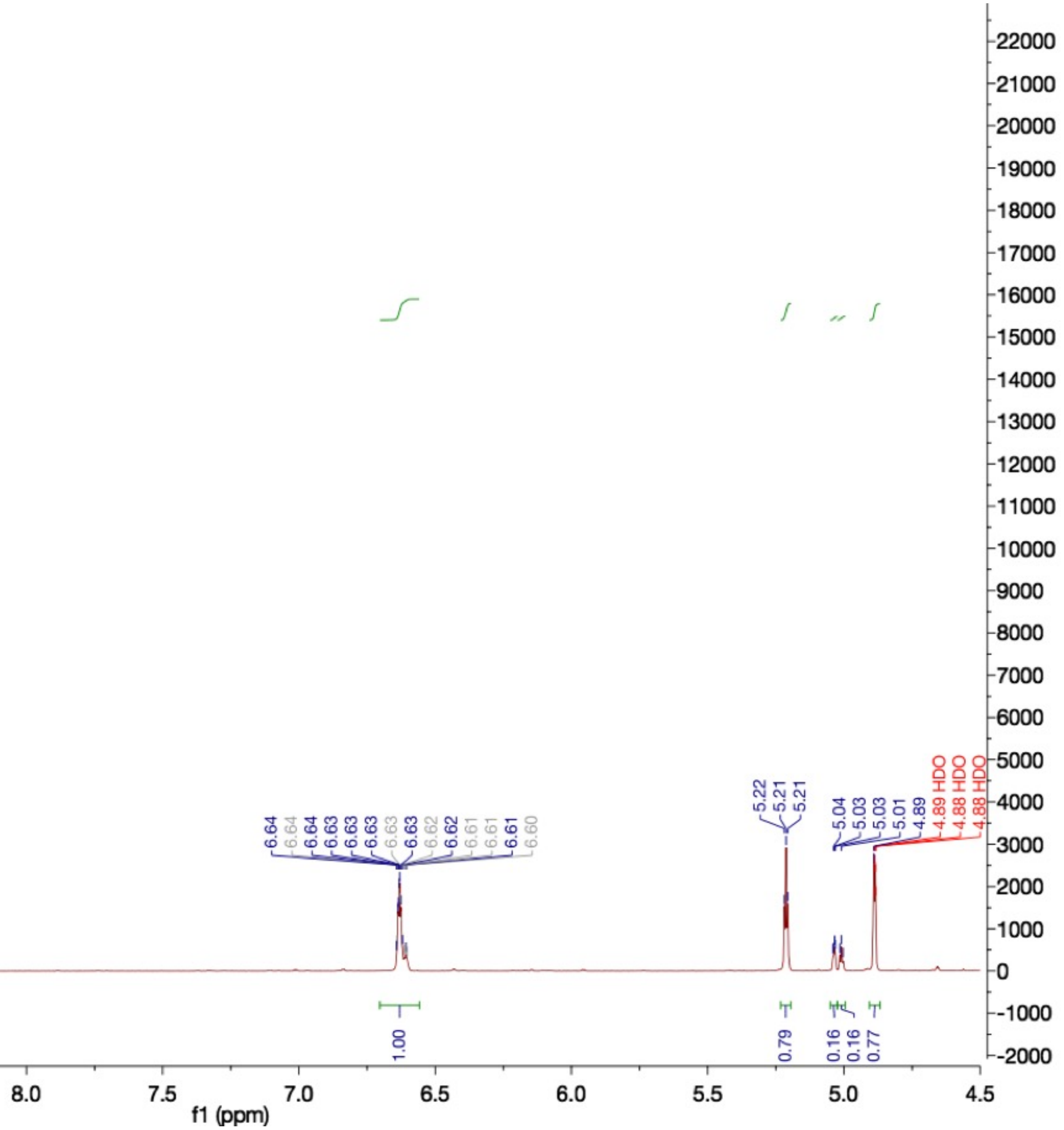

Figure S36. ^{1}H NMR (400 MHz, DMSO-d_6, 85 °C) *N*-Boc Bicyclic Esters ***N*-Boc-38** and ***N*-Boc-39** (4.5 – 10 ppm inset)

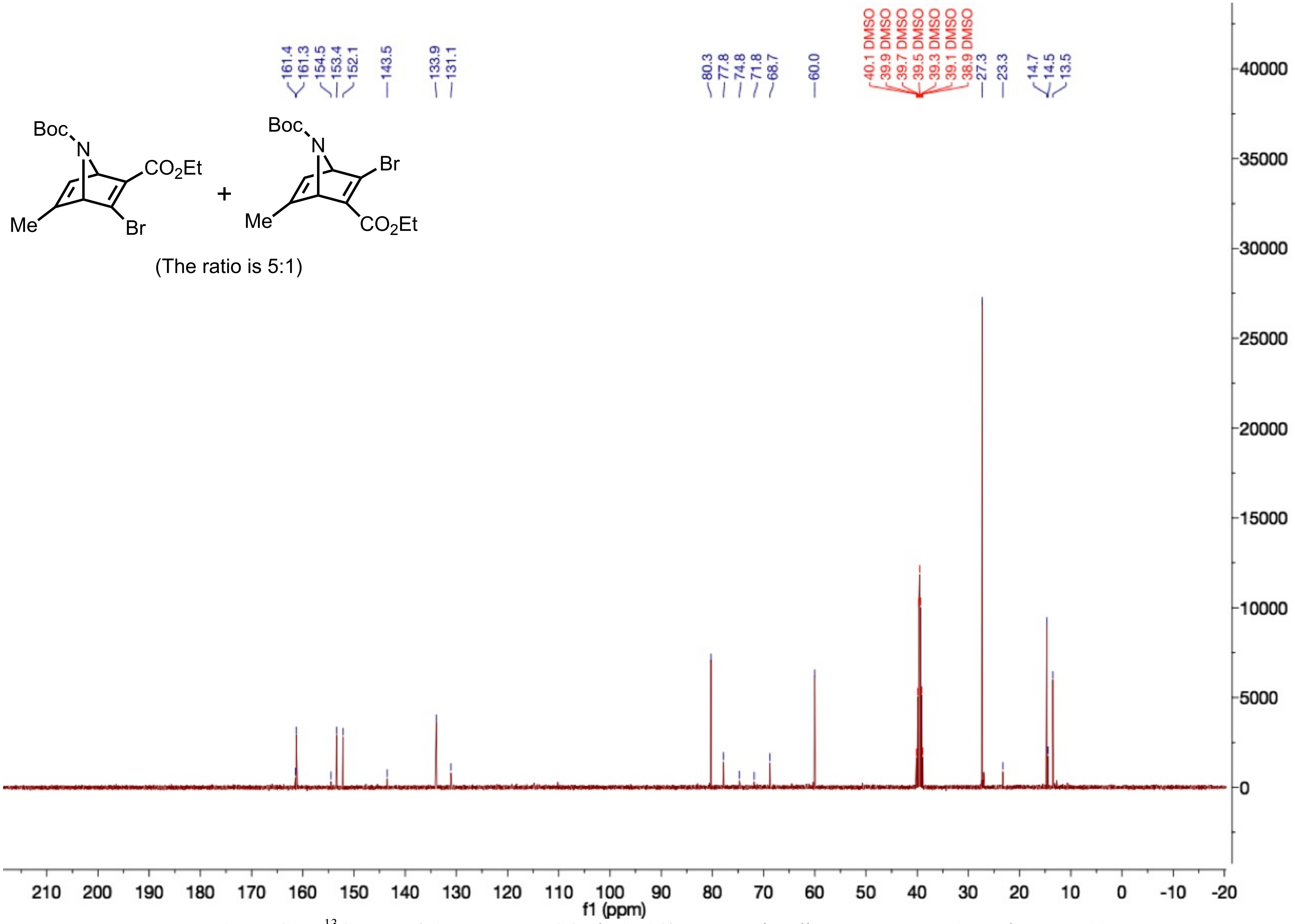

Figure S37. ^{13}C NMR (101 MHz, DMSO-d_6, 85 °C) *N*-Boc Bicyclic Esters ***N*-Boc-38** and ***N*-Boc-39**

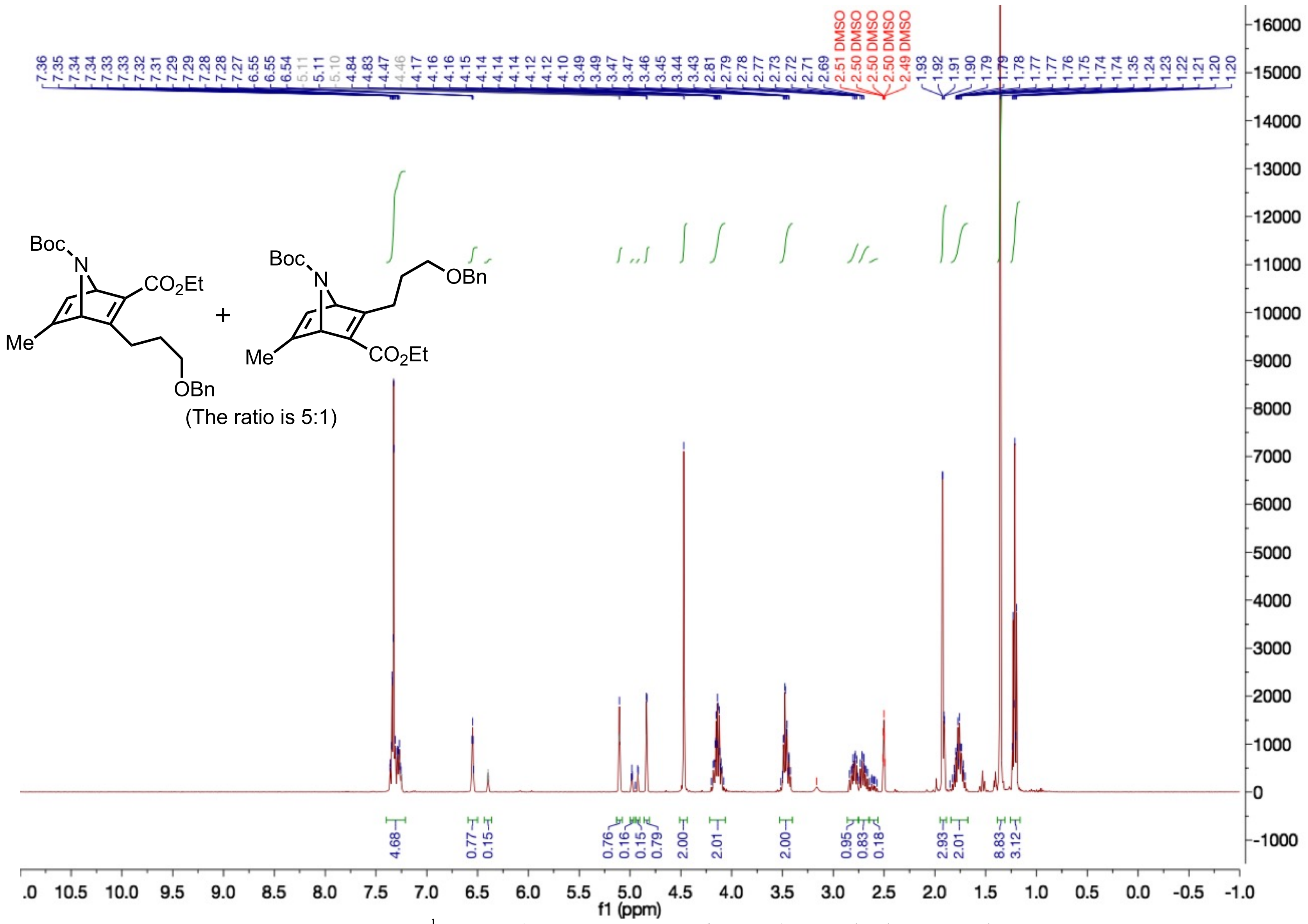

Figure S38. ^{1}H NMR (400 MHz, DMSO-d_6, 85 °C) Benzyl Ethers **41** and **SI-8**

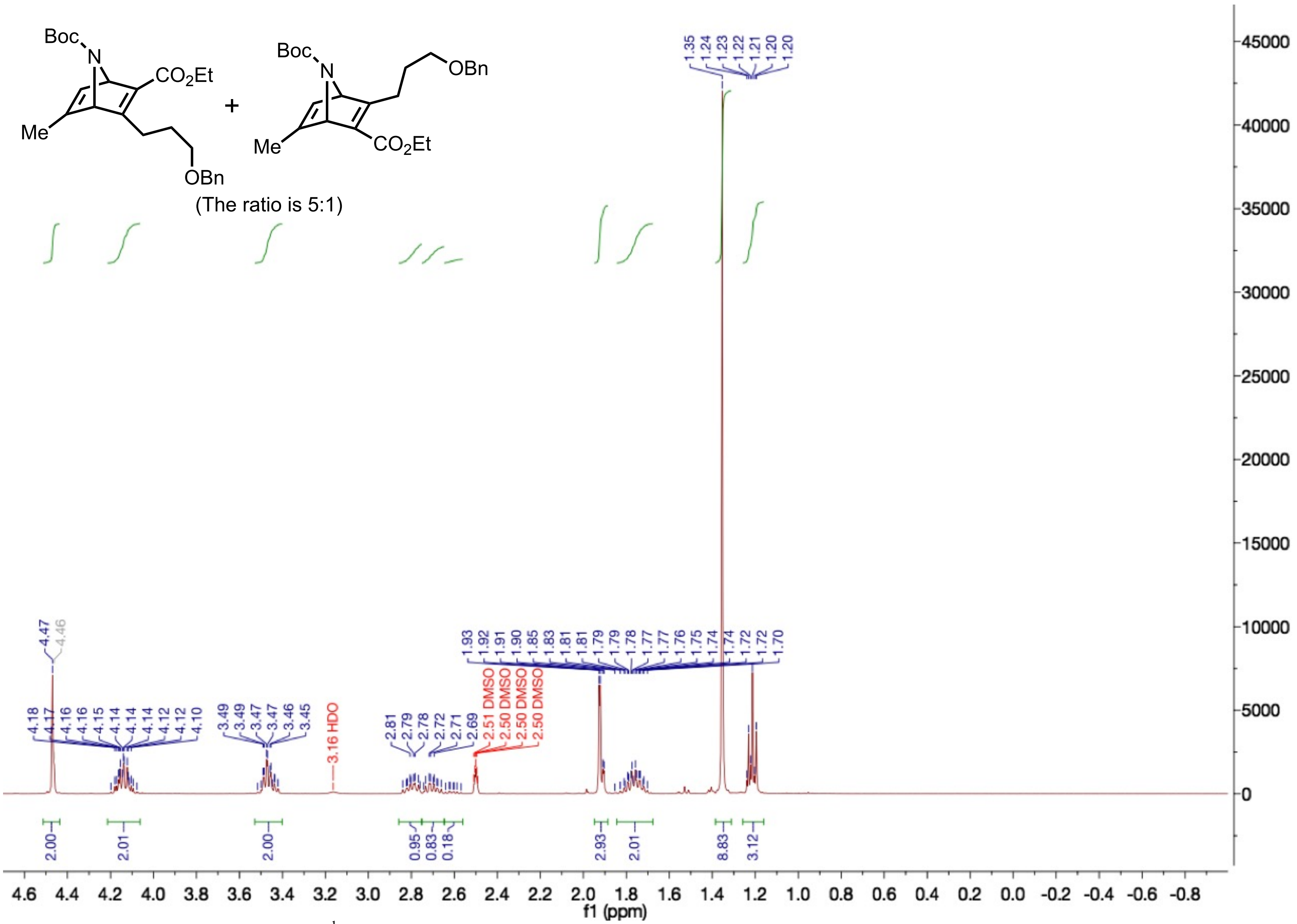

Figure S39. ^{1}H NMR (400 MHz, DMSO-d_6, 85 °C) Benzyl Ethers **41** and **SI-8** (-1 – 4.7 ppm inset)

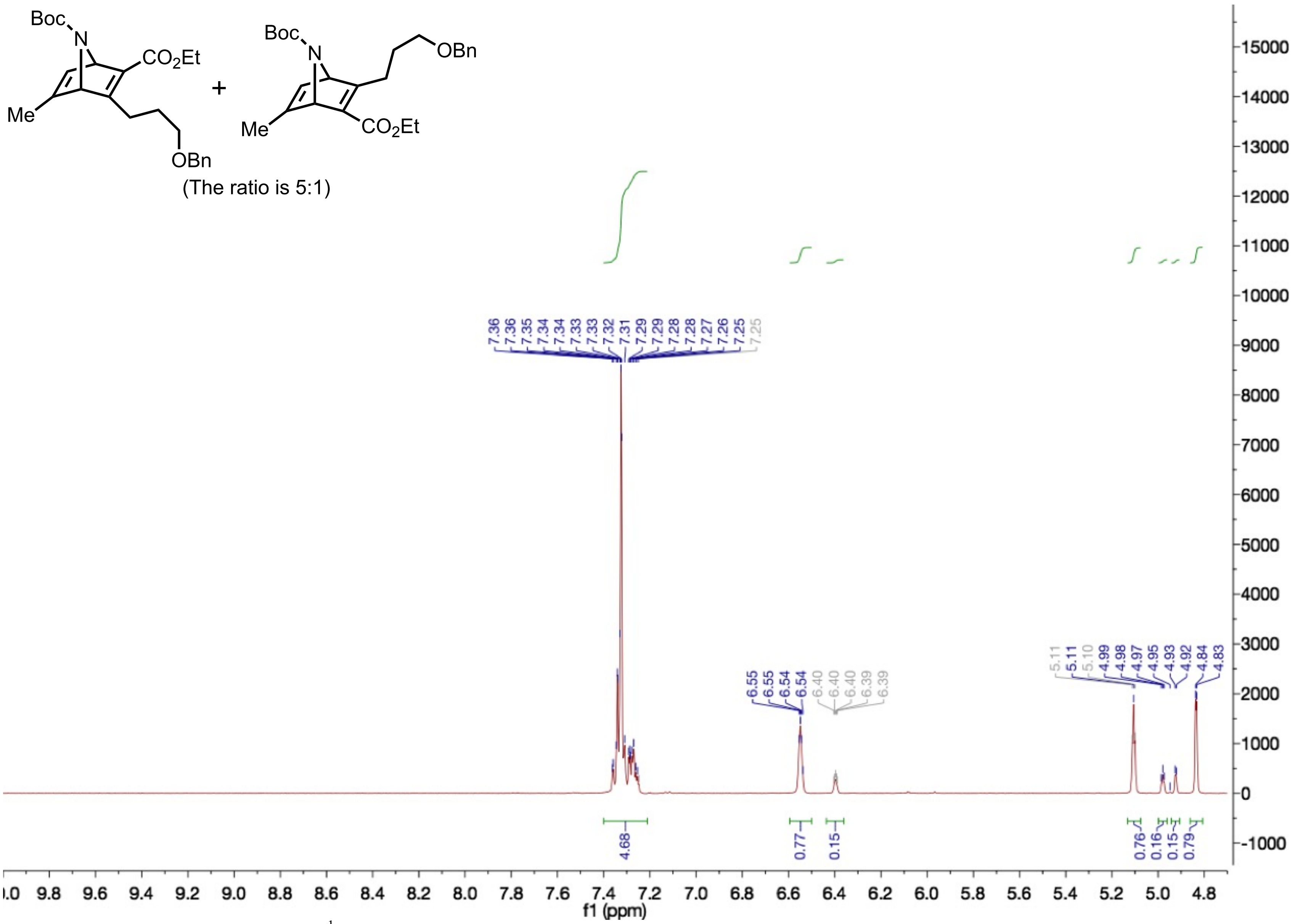

Figure S40. ^{1}H NMR (400 MHz, DMSO-d_6, 85 °C) Benzyl Ethers **41** and **SI-8** (4.7 – 10 ppm inset)

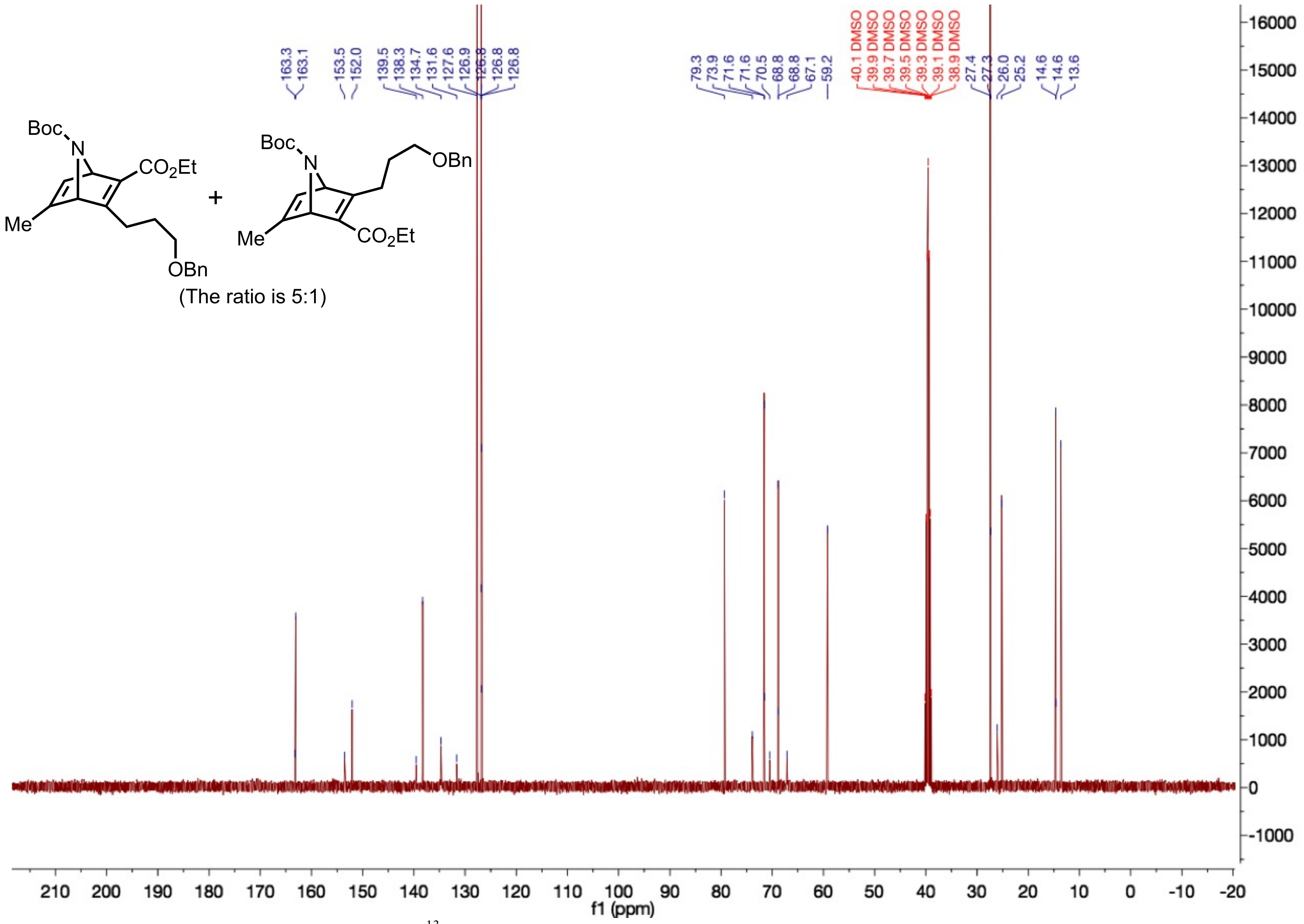

Figure S41. ^{13}C NMR (101 MHz, DMSO-d_6, 85 °C) Benzyl Ethers **41** and **SI-8**

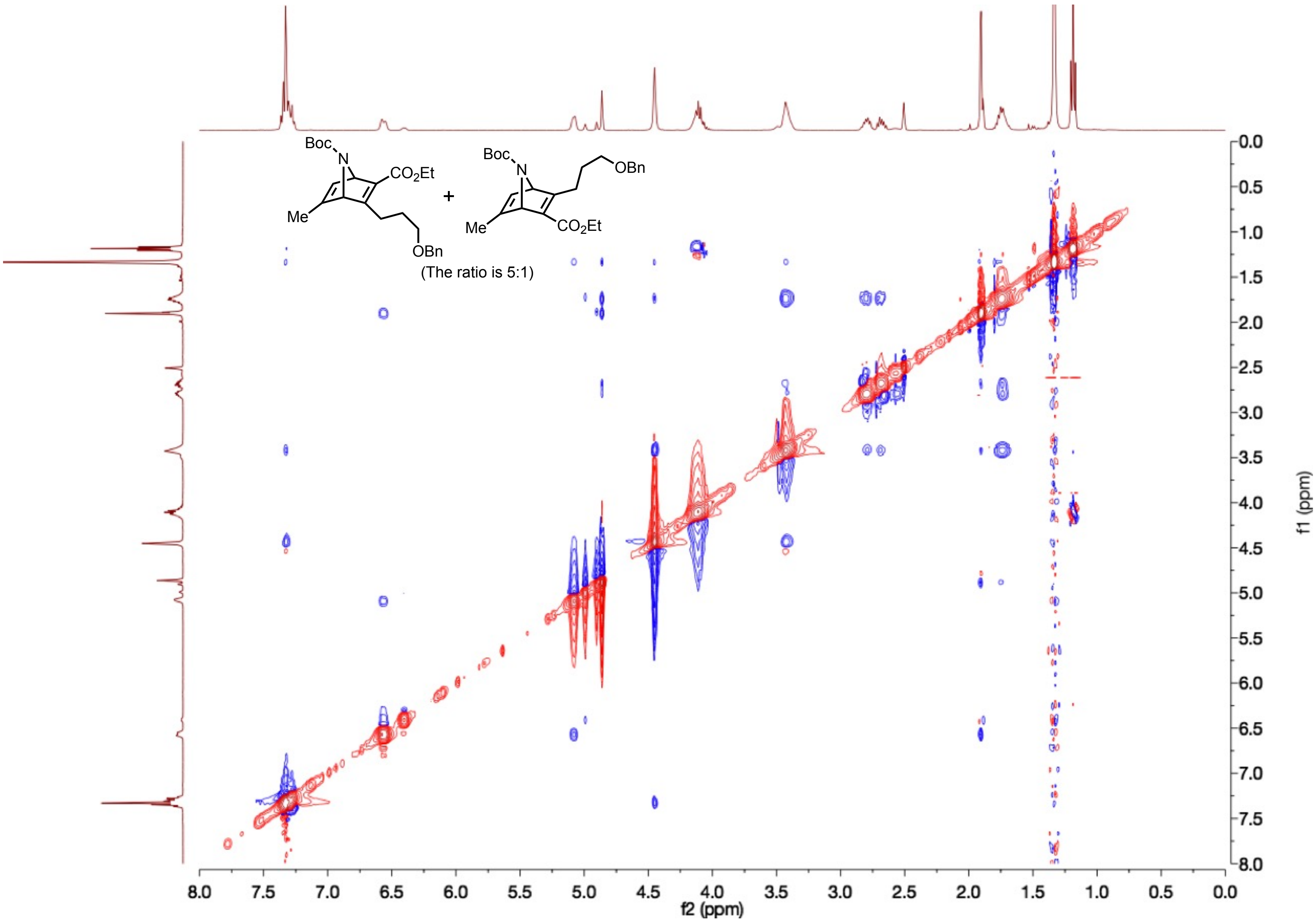

Figure S42. NOESY (DMSO-d_6, 27 °C) Benzyl Ethers **41** and **SI-8**

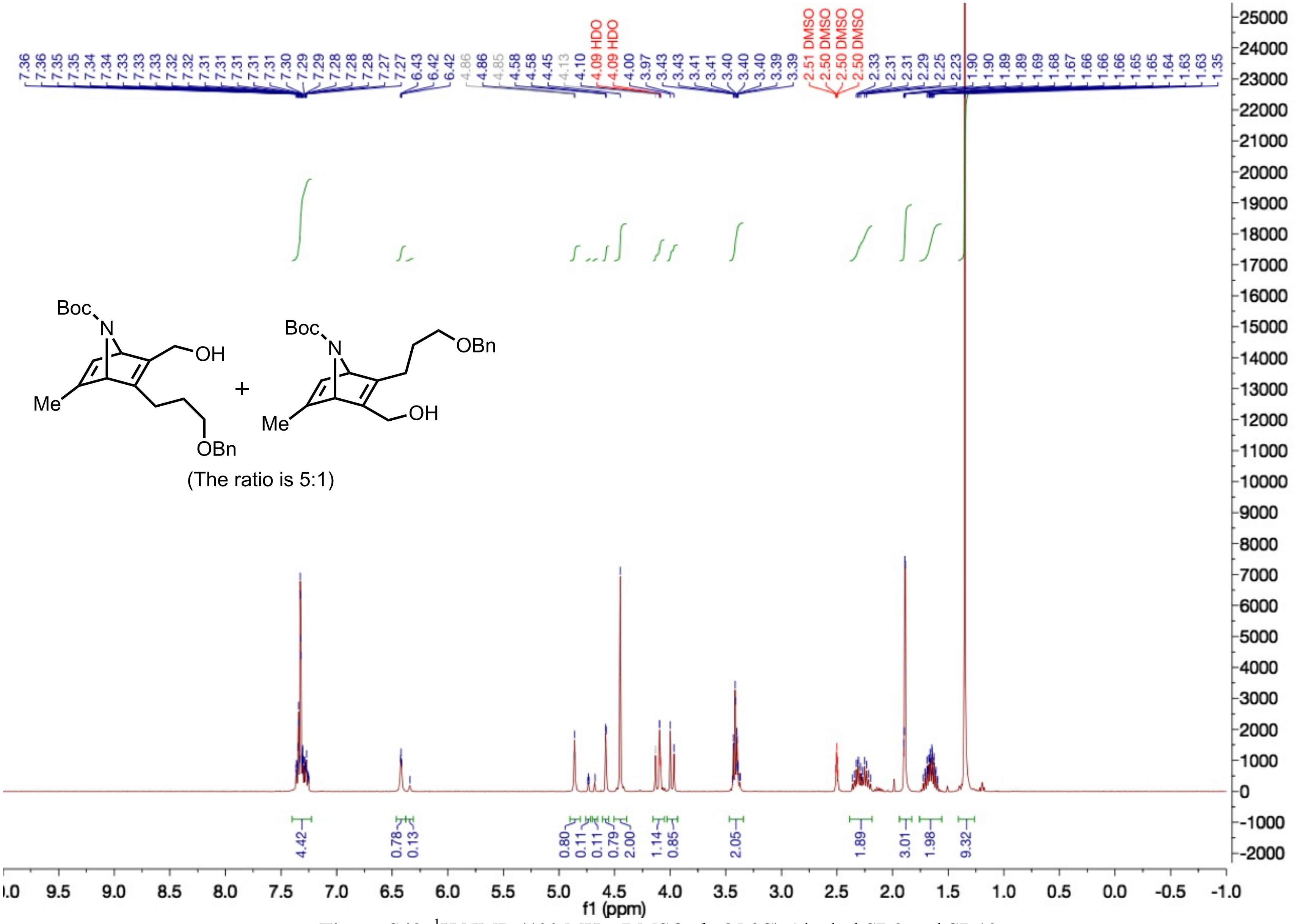

Figure S43. ^{1}H NMR (400 MHz, DMSO-d_6, 85 °C) Alcohol **SI-9** and **SI-10**

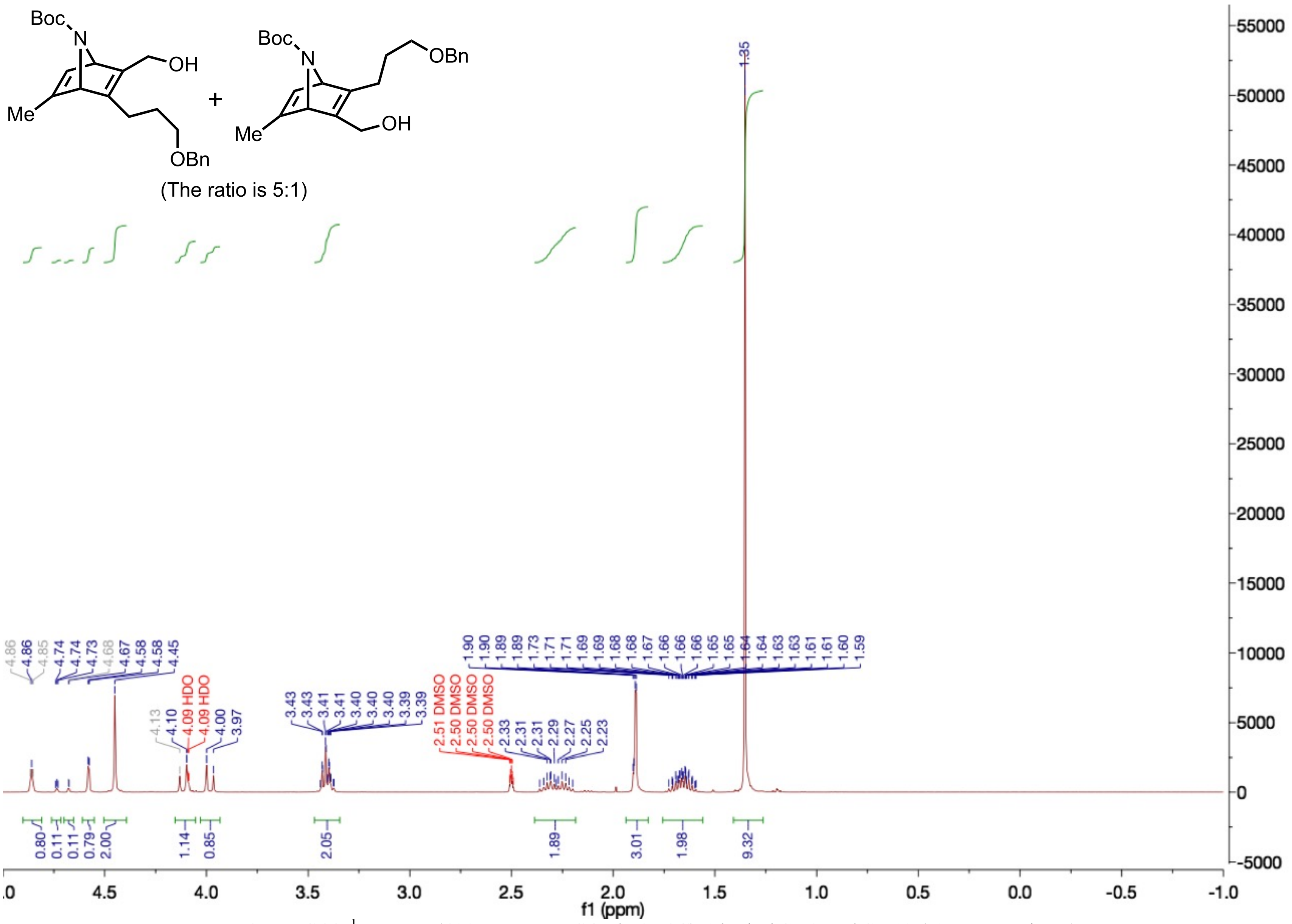

Figure S44. ^{1}H NMR (400 MHz, DMSO-d_6, 85 °C) Alcohol **SI-9** and **SI-10** (-1 – 5 ppm inset)

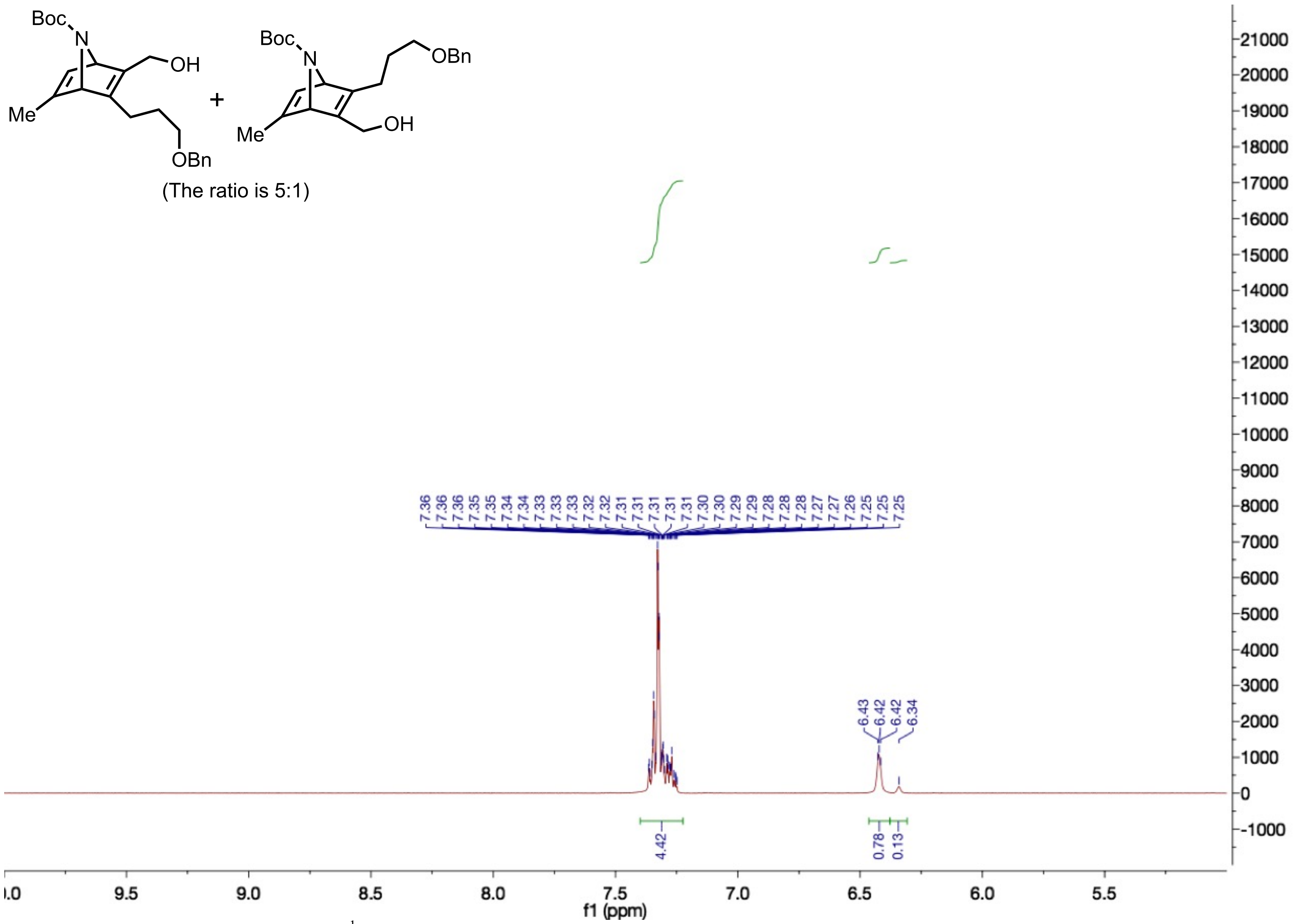

Figure S45. ^{1}H NMR (400 MHz, DMSO-d_6, 85 °C) Alcohol **SI-9** and **SI-10** (5 – 10 ppm inset)

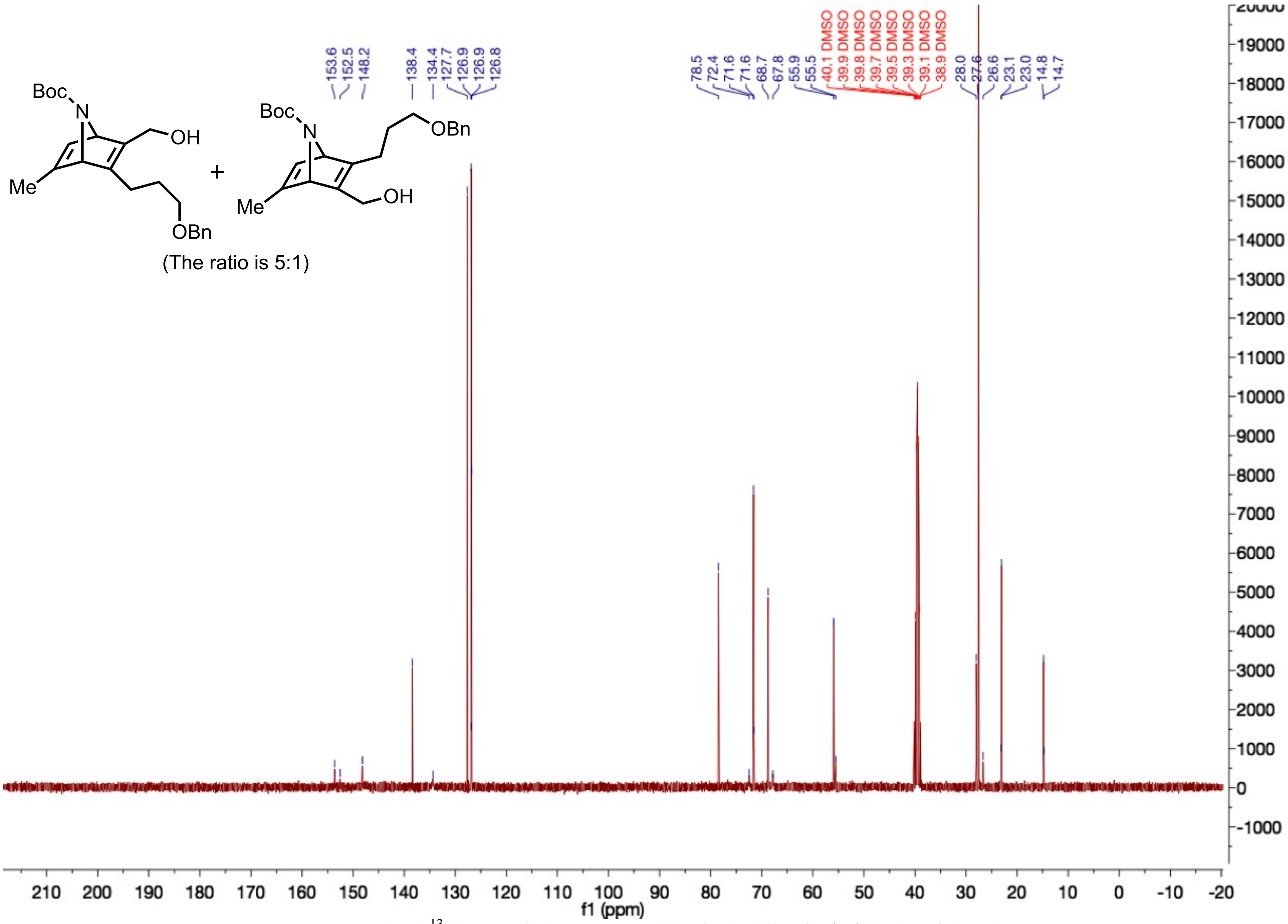

Figure S46. ^{13}C NMR (101 MHz, DMSO-d_6, 85 °C) Alcohol **SI-9** and **SI-10**

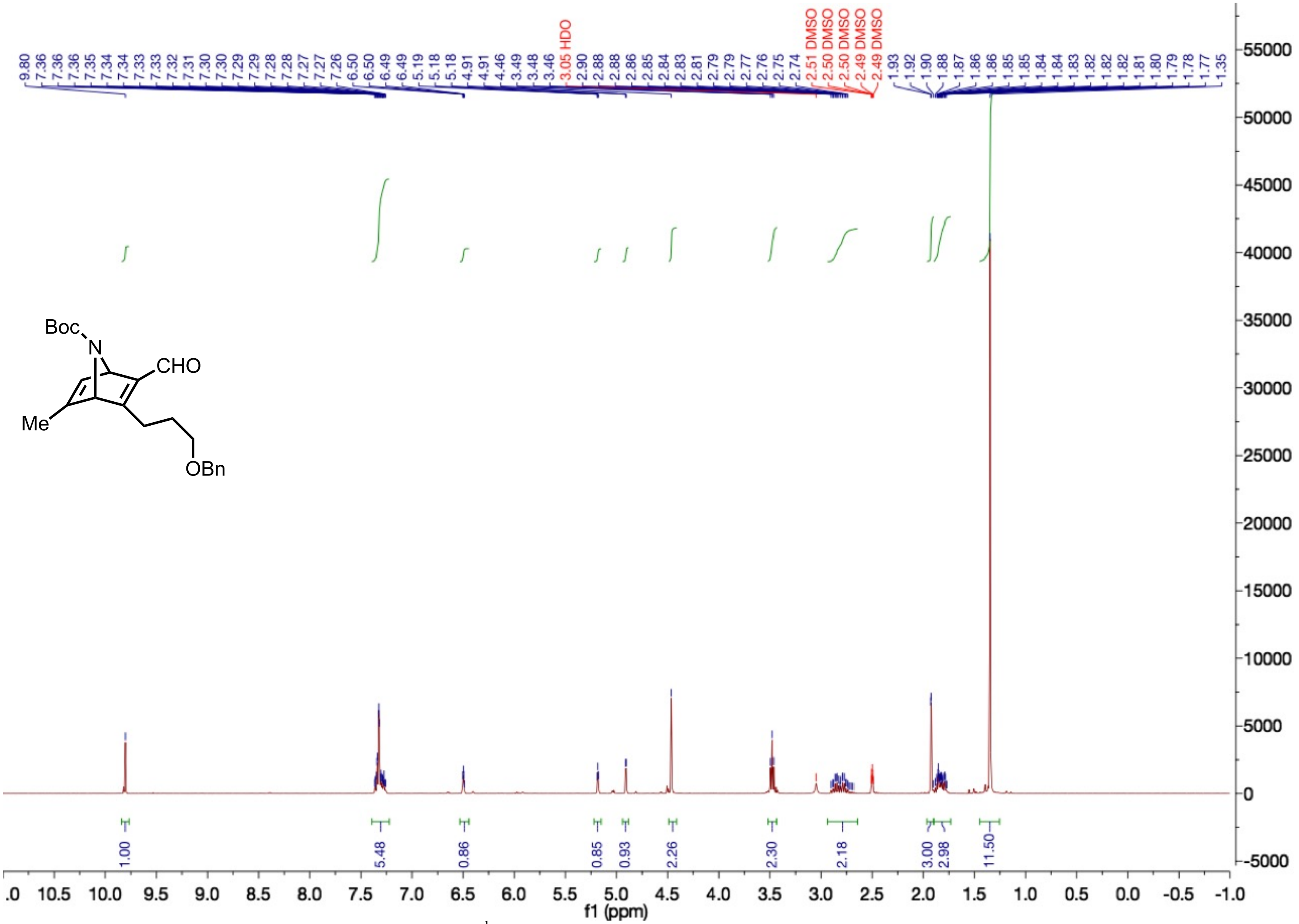

Figure S47. ^{1}H NMR (400 MHz, DMSO-d_6, 85 °C) Aldehyde **42**

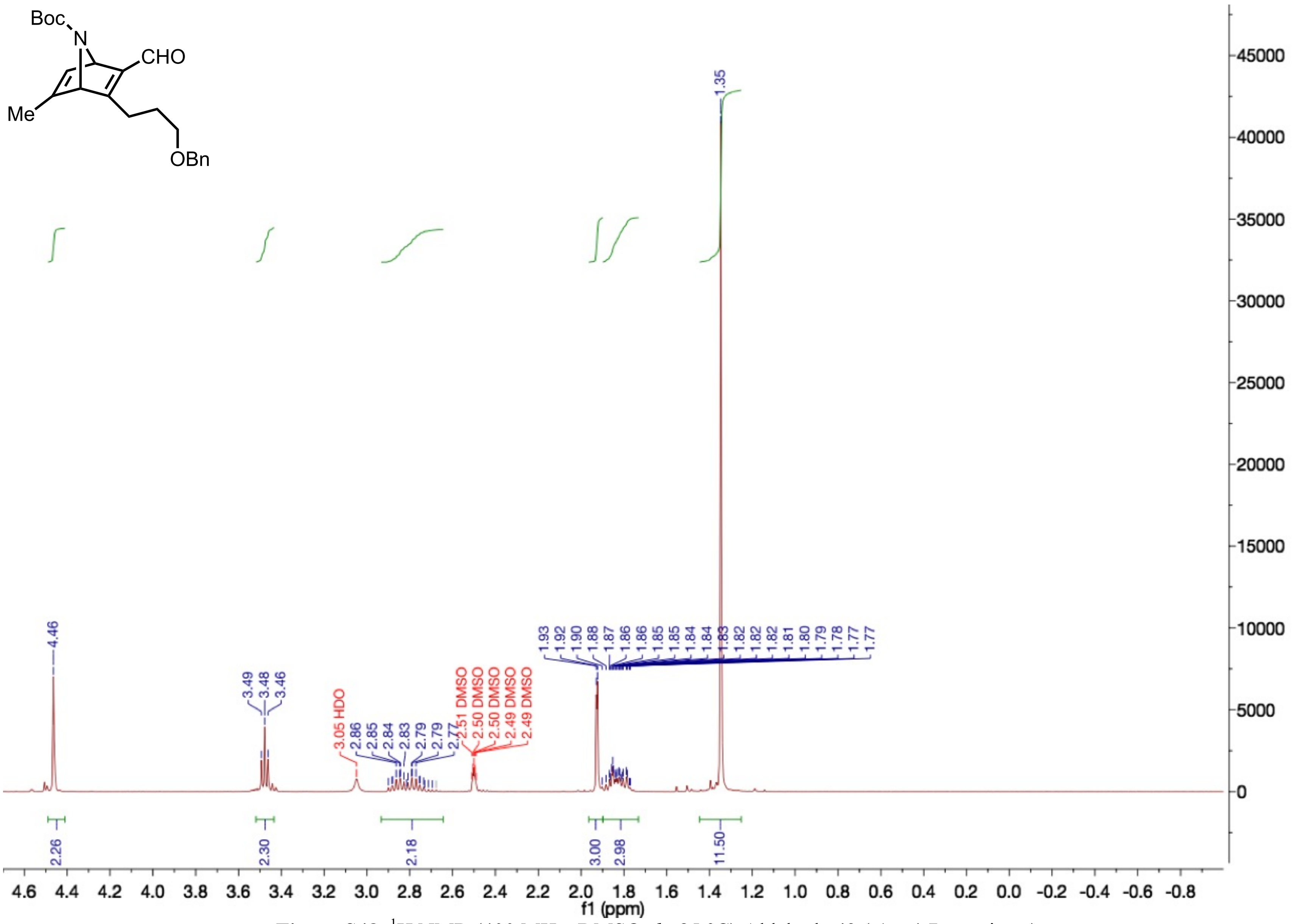

Figure S48. ^{1}H NMR (400 MHz, DMSO-d_6, 85 °C) Aldehyde **42** (-1 – 4.7 ppm inset)

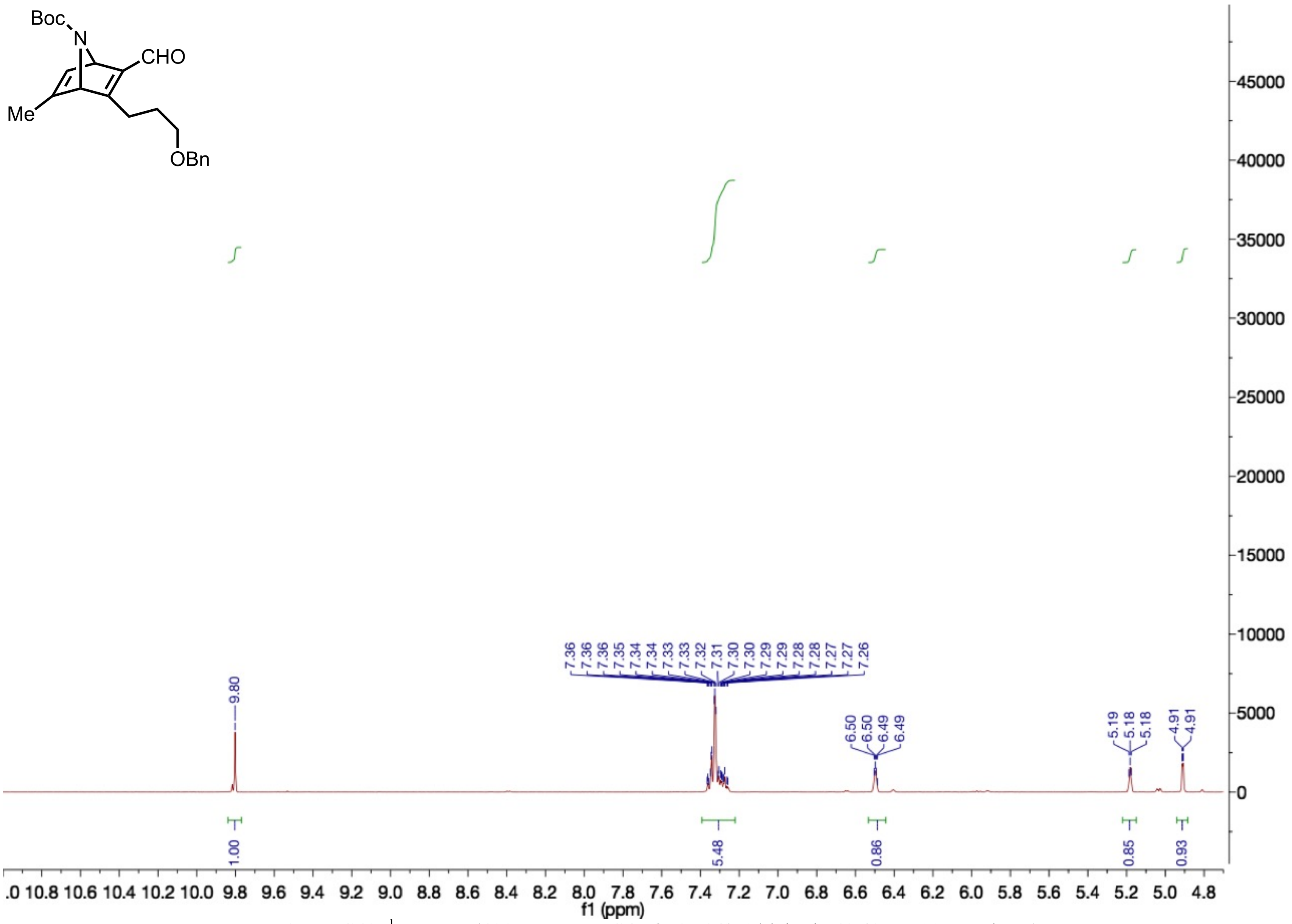

Figure S49. ^{1}H NMR (400 MHz, DMSO-d_6, 85 °C) Aldehyde **42** (4.7 – 11 ppm inset)

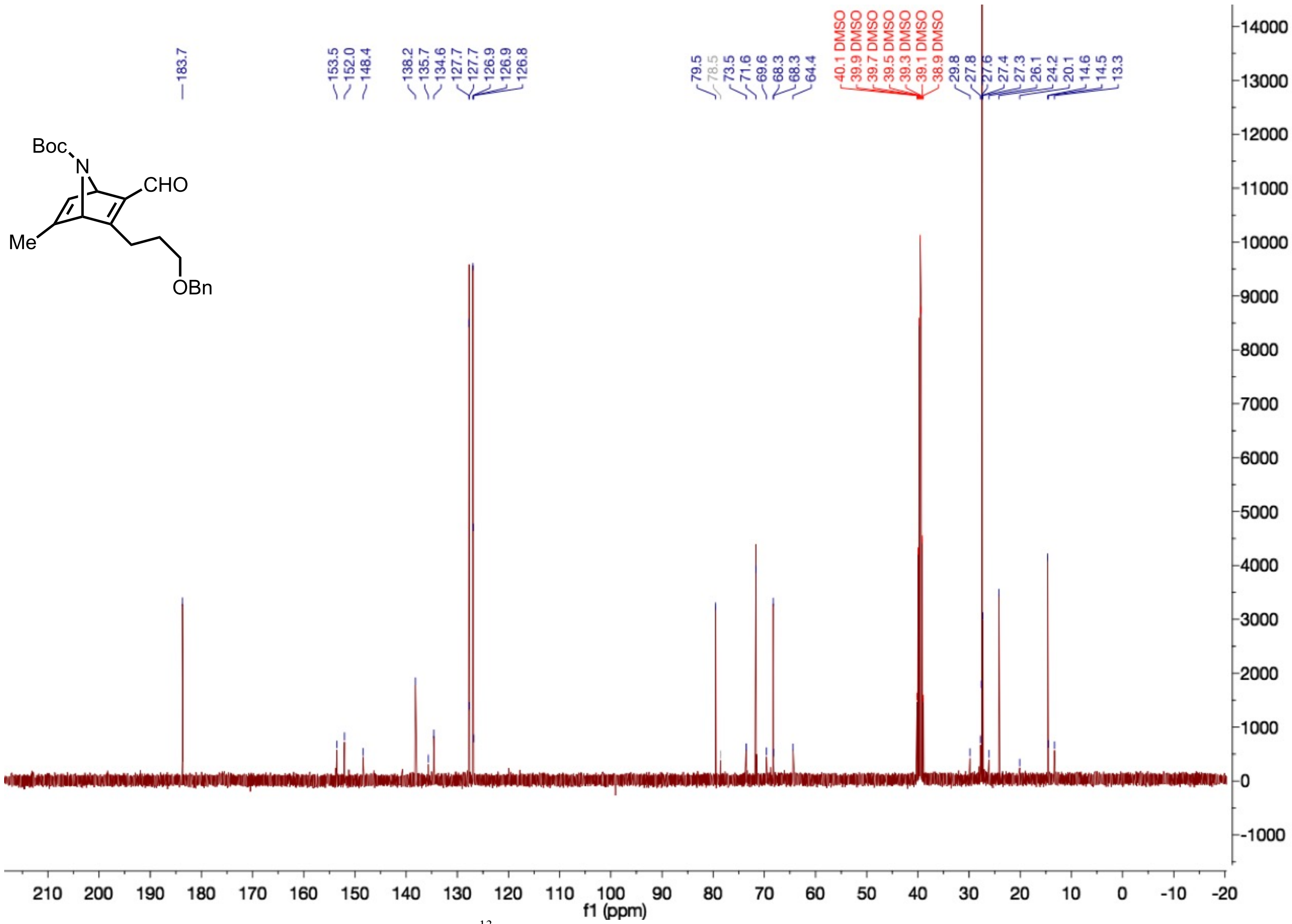

Figure S50. ^{13}C NMR (101 MHz, DMSO-d_6, 85 °C) Aldehyde **42**

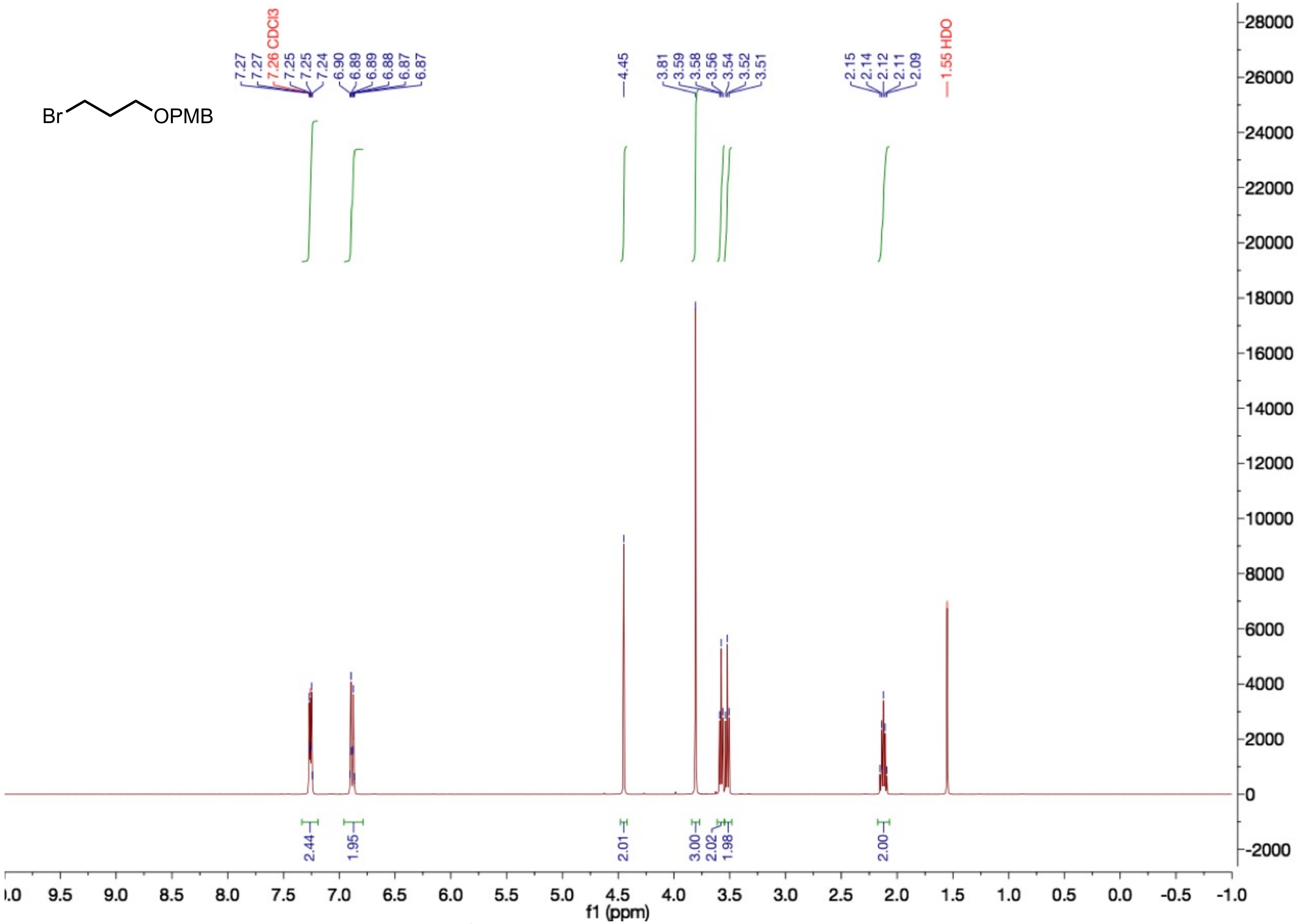

Figure S51. ^{1}H NMR (400 MHz, chloroform-*d*) alkyl bromide **SI-12**

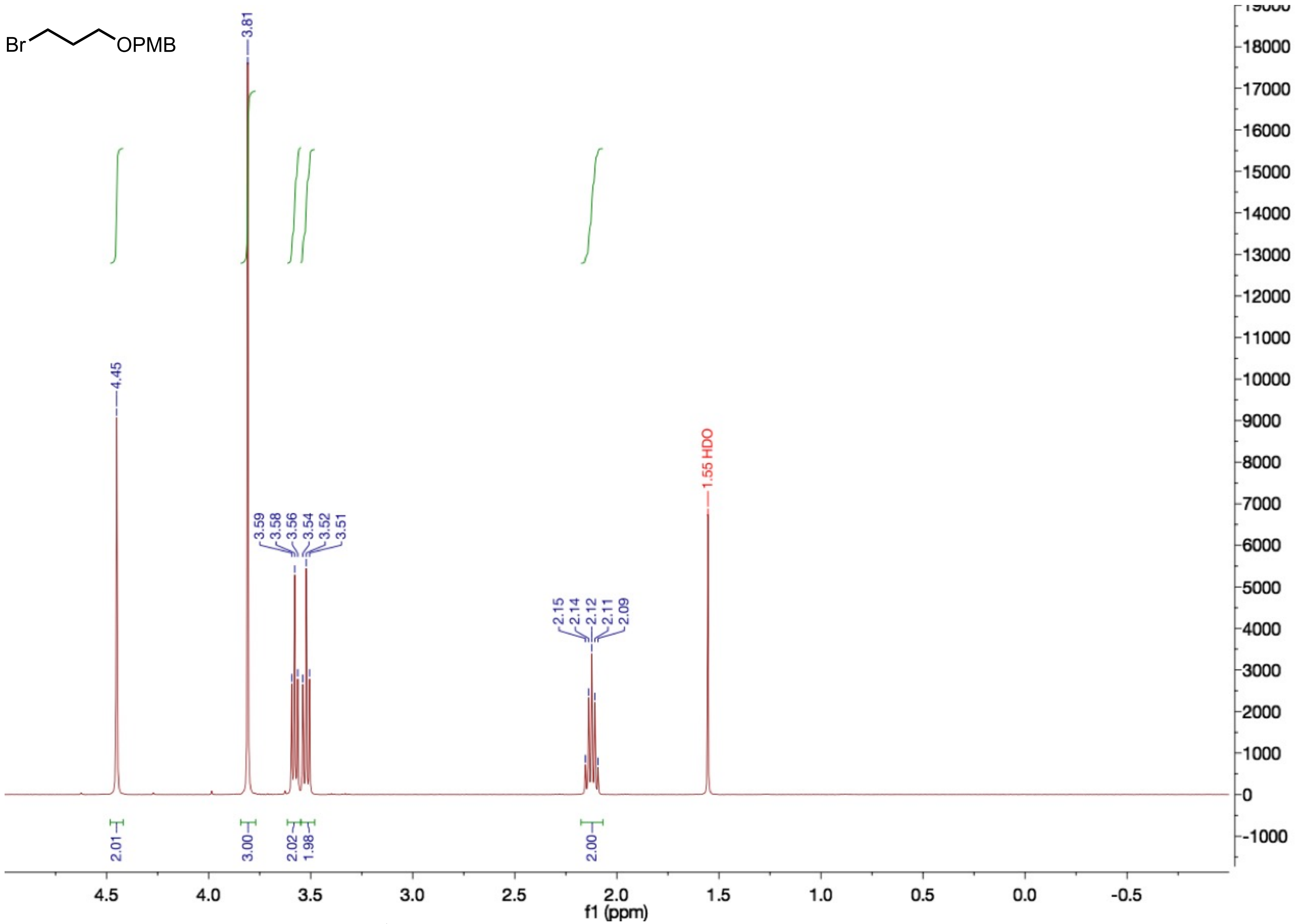

Figure S52. ^{1}H NMR (400 MHz, chloroform-*d*) alkyl bromide **SI-12** (-1 – 5 ppm inset)

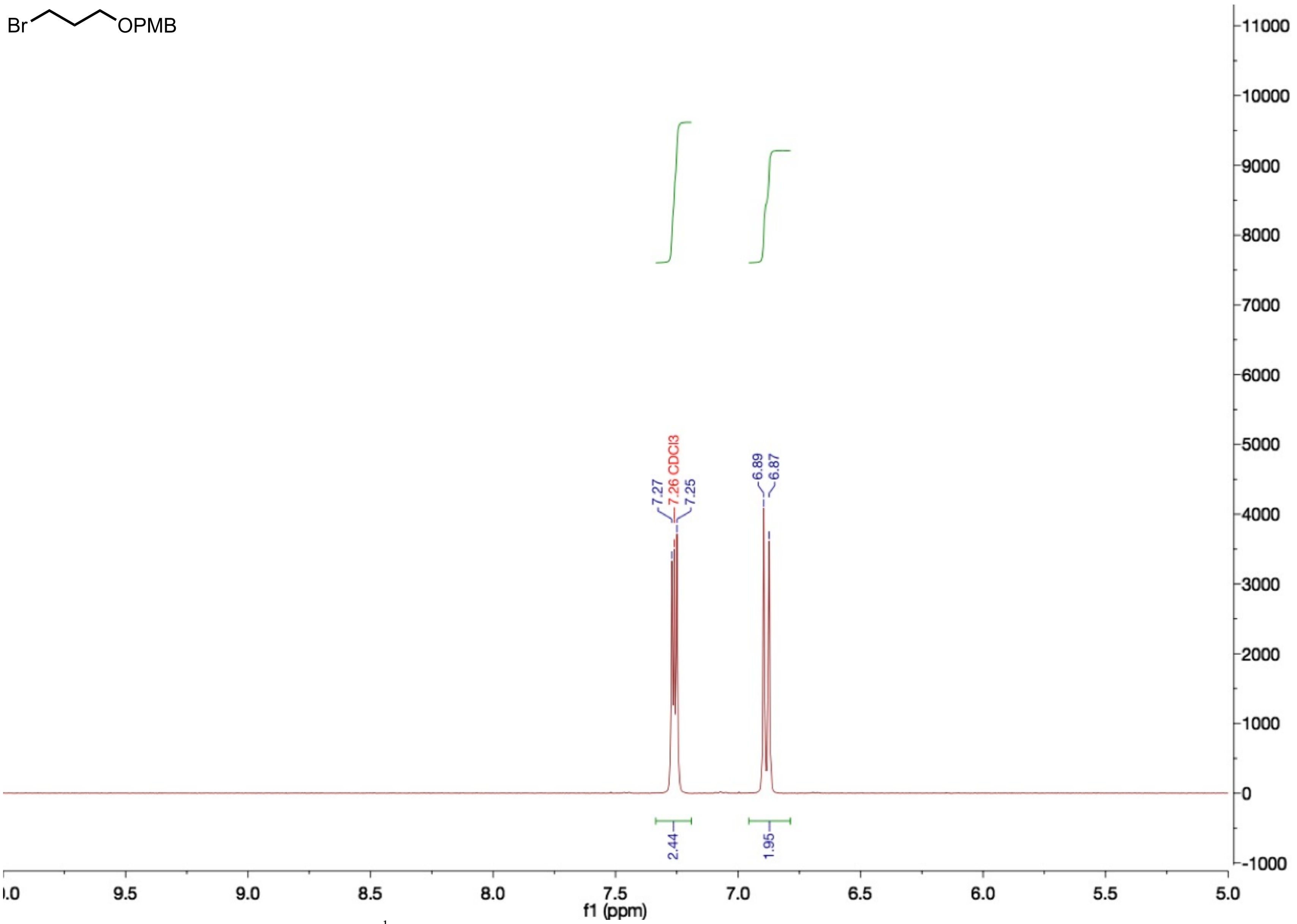

Figure S53. ^{1}H NMR (400 MHz, chloroform-*d*) alkyl bromide **SI-12** (5 – 10 ppm inset)

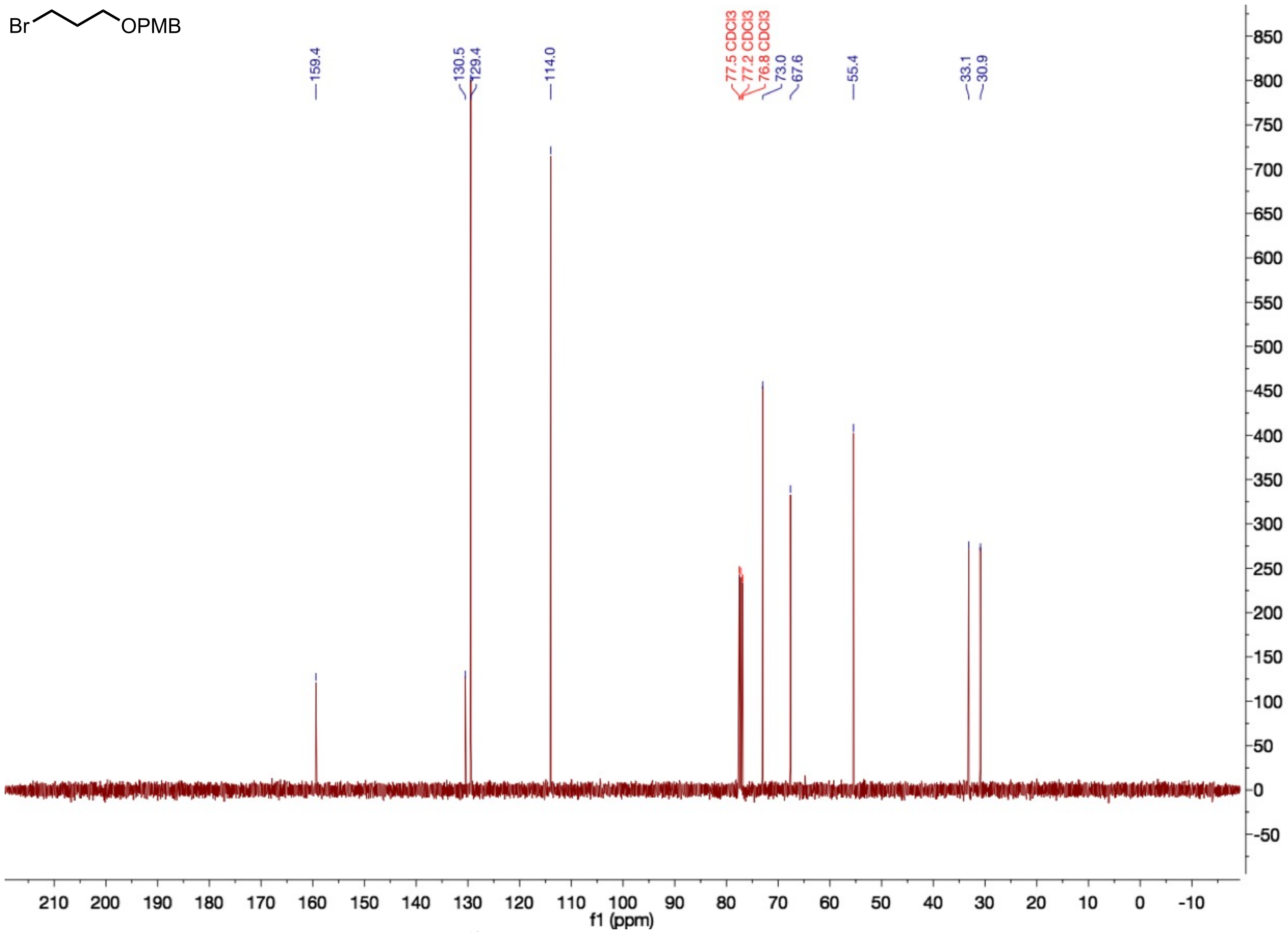

Figure S54. ^{13}C NMR (101 MHz, chloroform-*d*) alkyl bromide **SI-12**

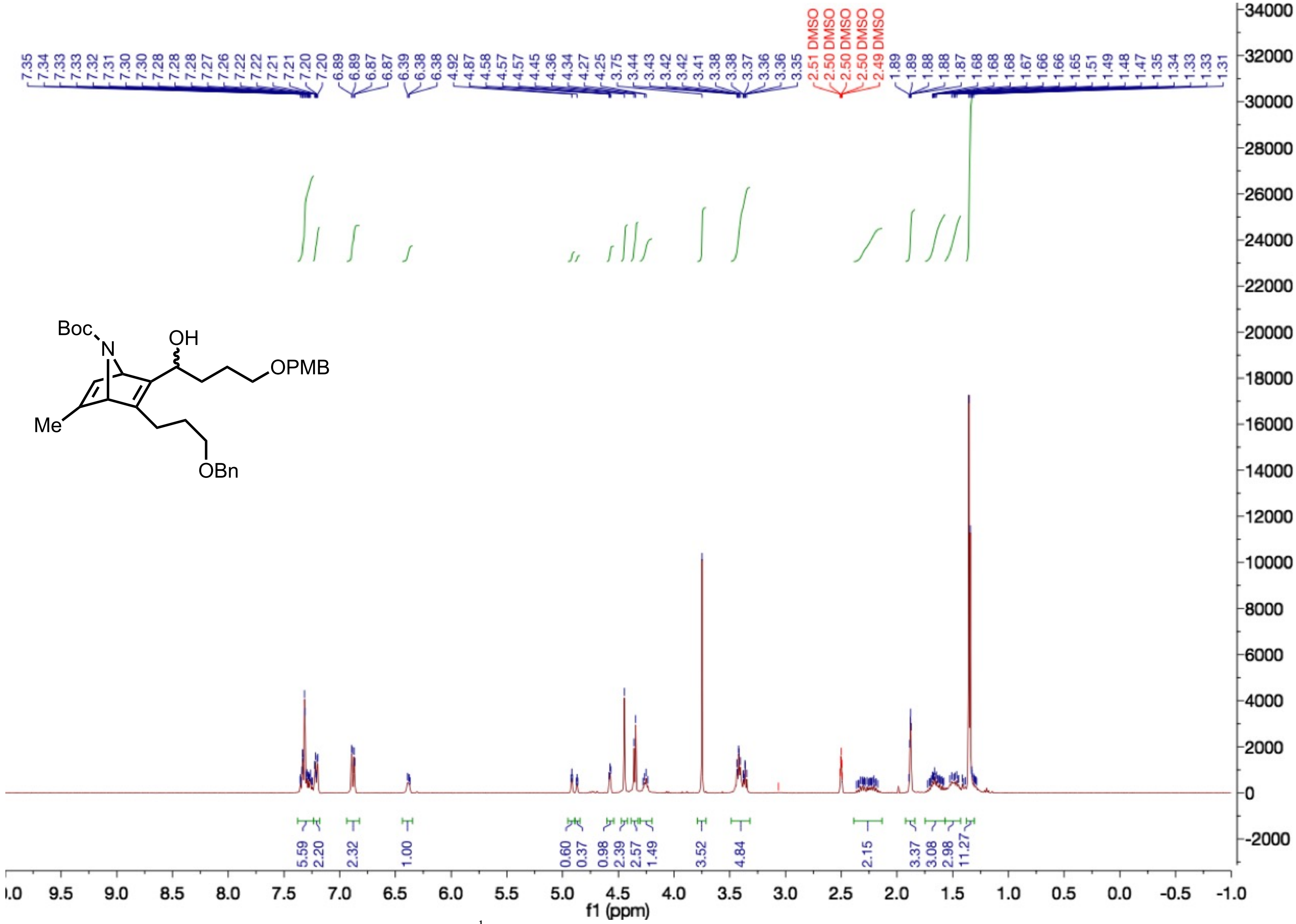

Figure S55. ^{1}H NMR (400 MHz, DMSO-d_6, 85 °C) PMB Ether **44**

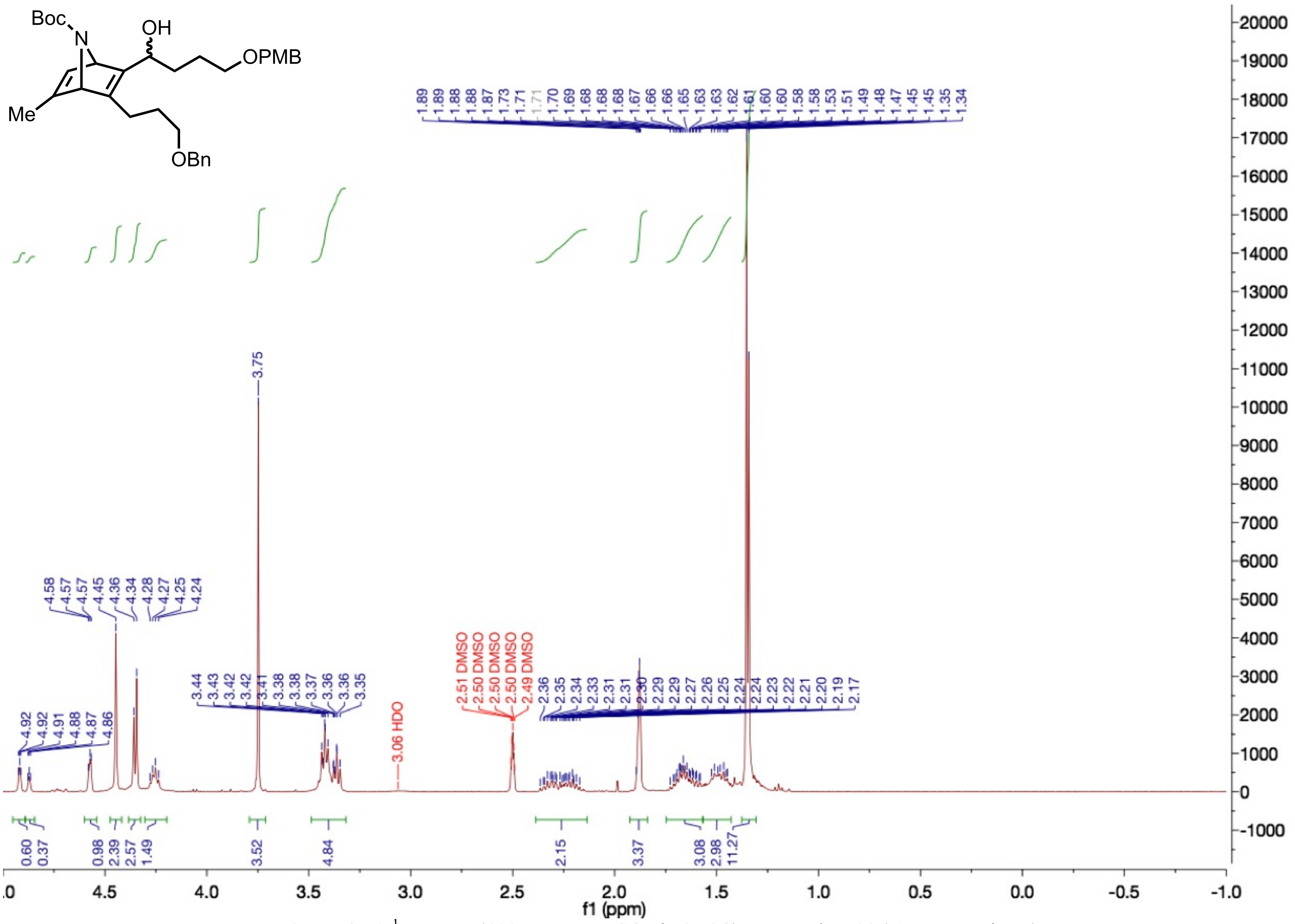

Figure S56. ^{1}H NMR (400 MHz, DMSO-d_6, 85 °C) PMB Ether **44** (-1 – 5 ppm inset)

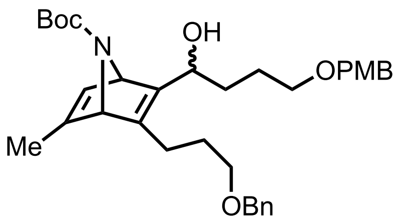

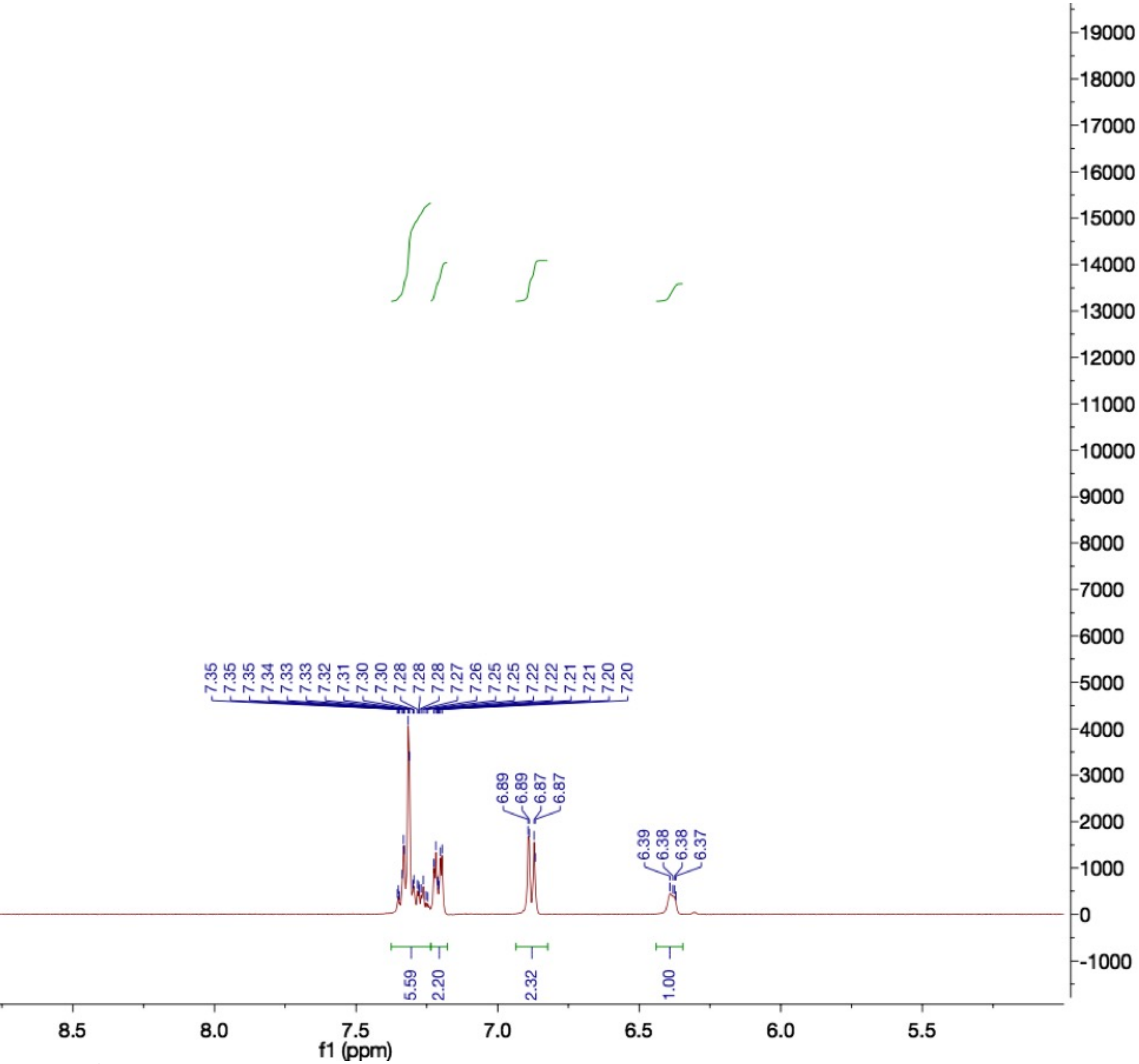

Figure S57. ^{1}H NMR (400 MHz, DMSO-d_6, 85 °C) PMB Ether **44** (5 – 10 ppm inset)

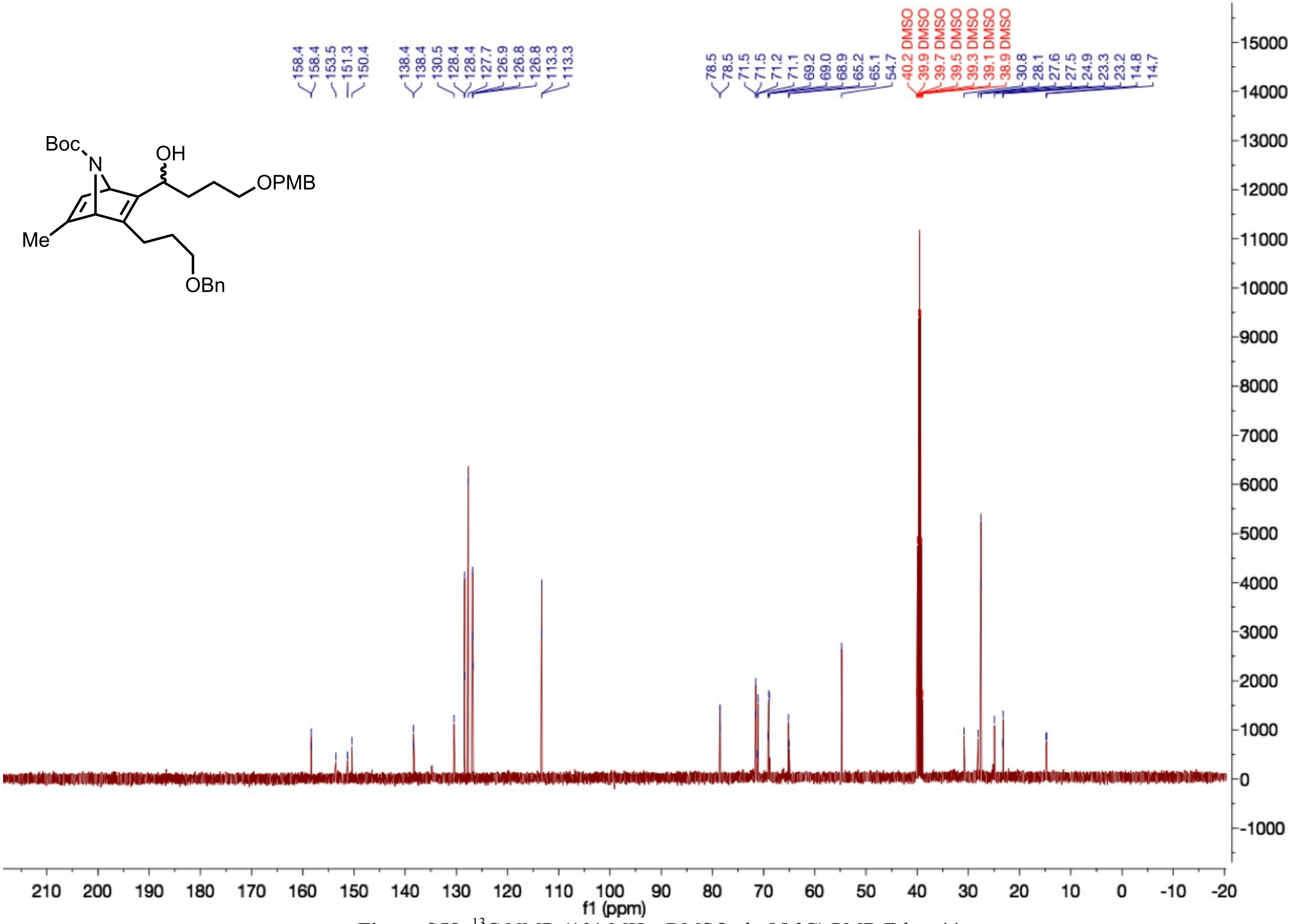

Figure S58. ^{13}C NMR (101 MHz, DMSO-d_6, 85 °C) PMB Ether **44**

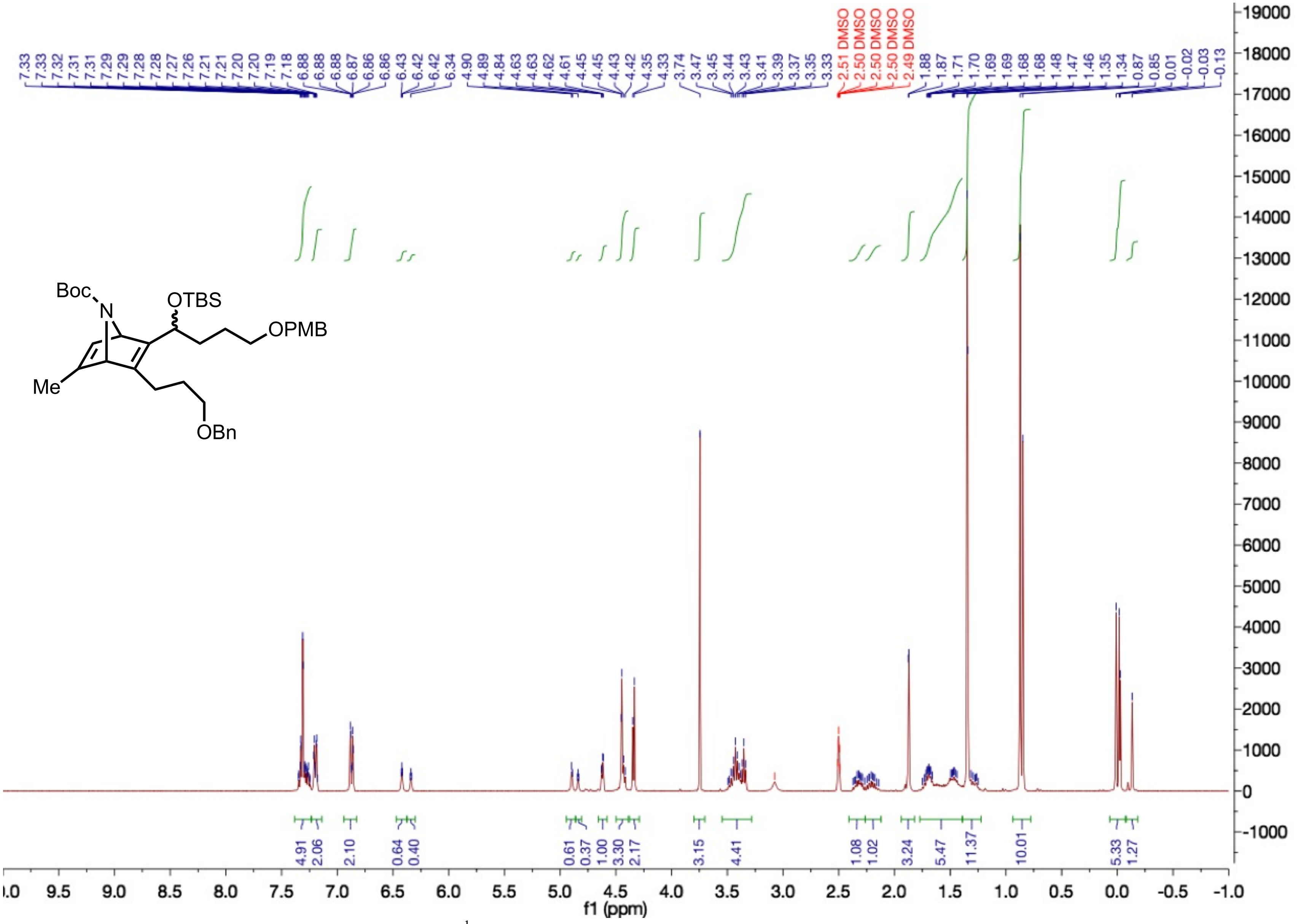

Figure S59. ^{1}H NMR (400 MHz, DMSO-d_6, 85 °C) TBS Ether **SI-13**

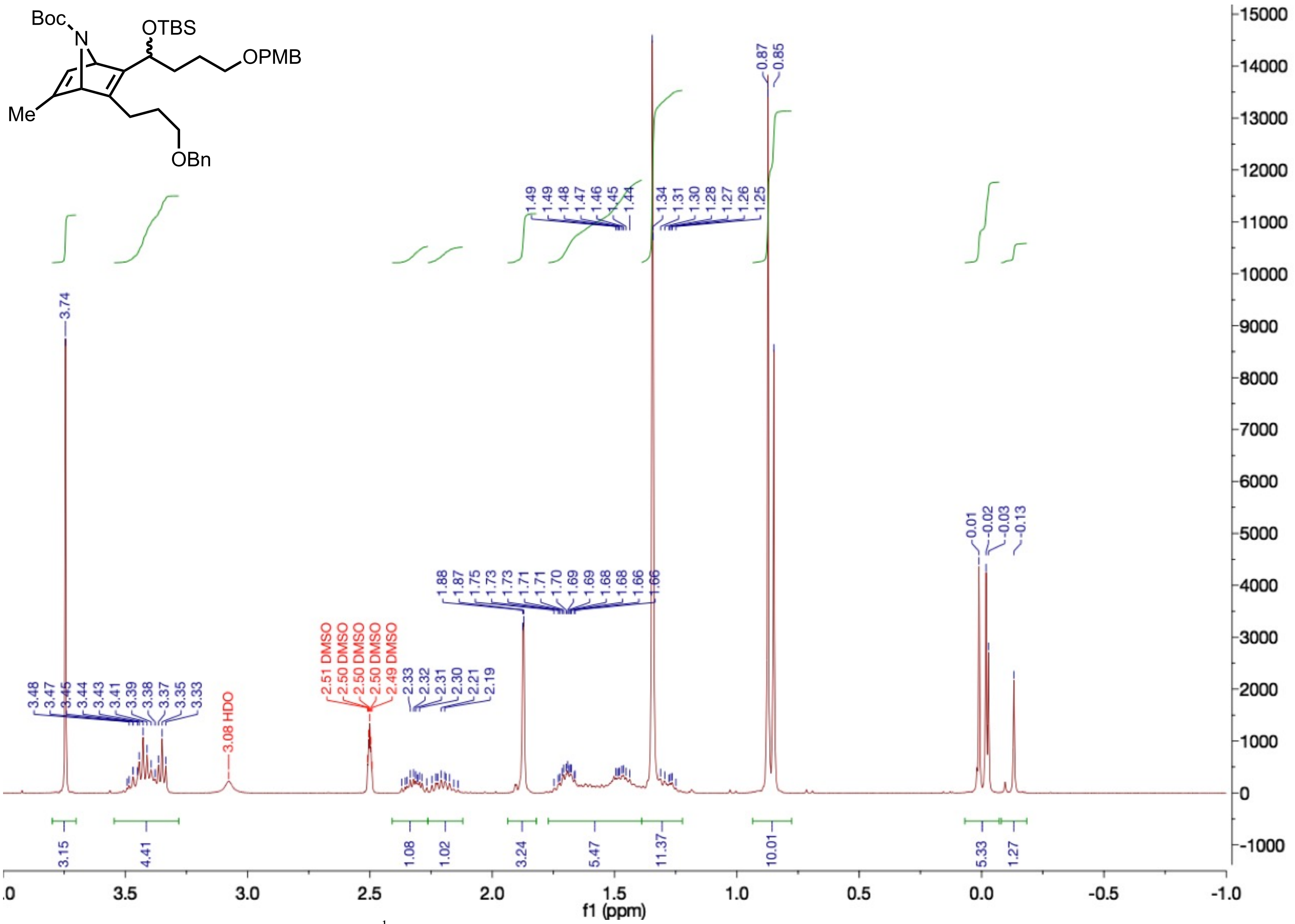

Figure S60. ^{1}H NMR (400 MHz, DMSO-d_6, 85 °C) TBS Ether **SI-13** (-1 – 4 ppm inset)

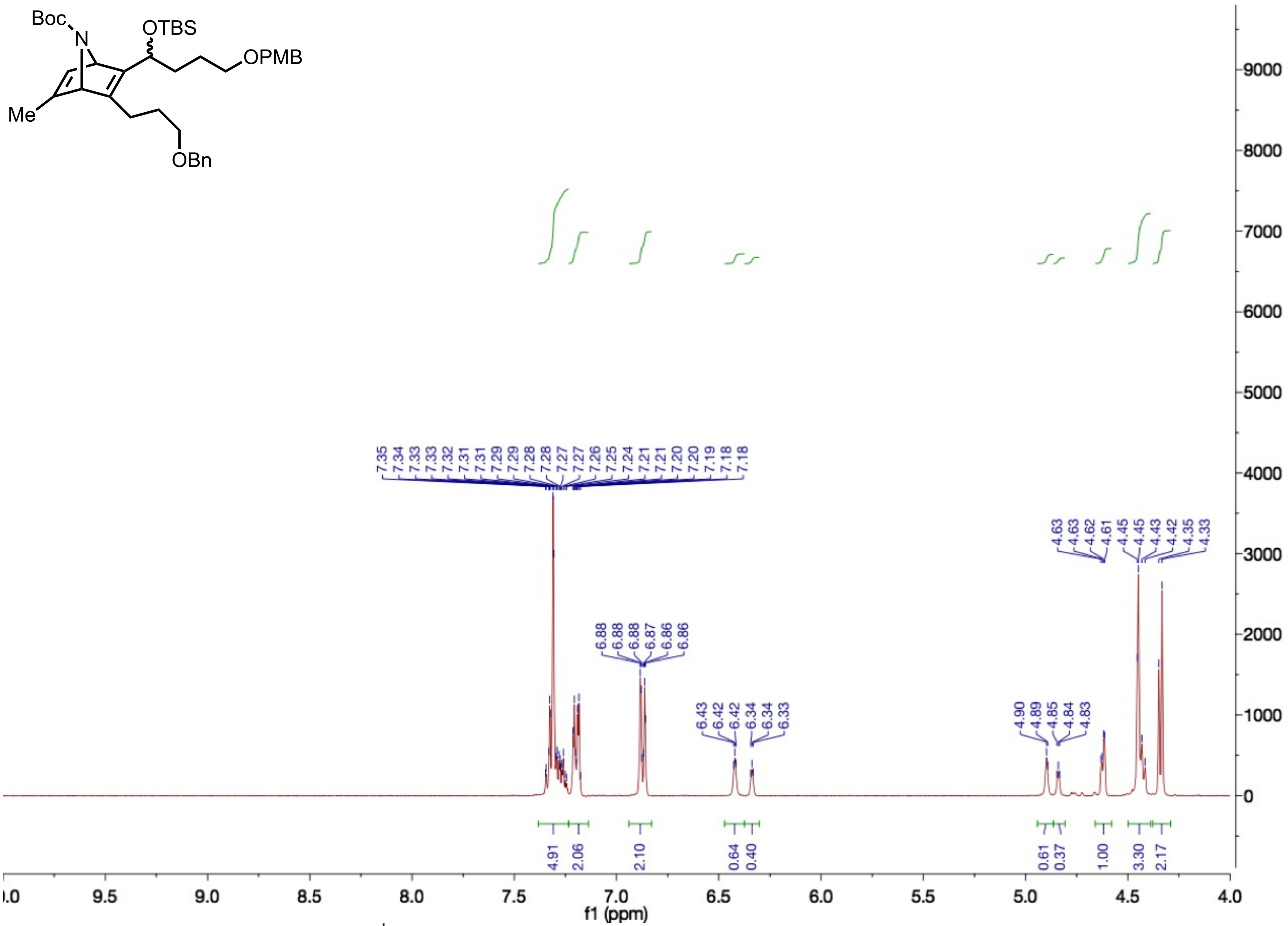

Figure S61. ^{1}H NMR (400 MHz, DMSO-d_6, 85 °C) TBS Ether **SI-13** (4 – 10 ppm inset)

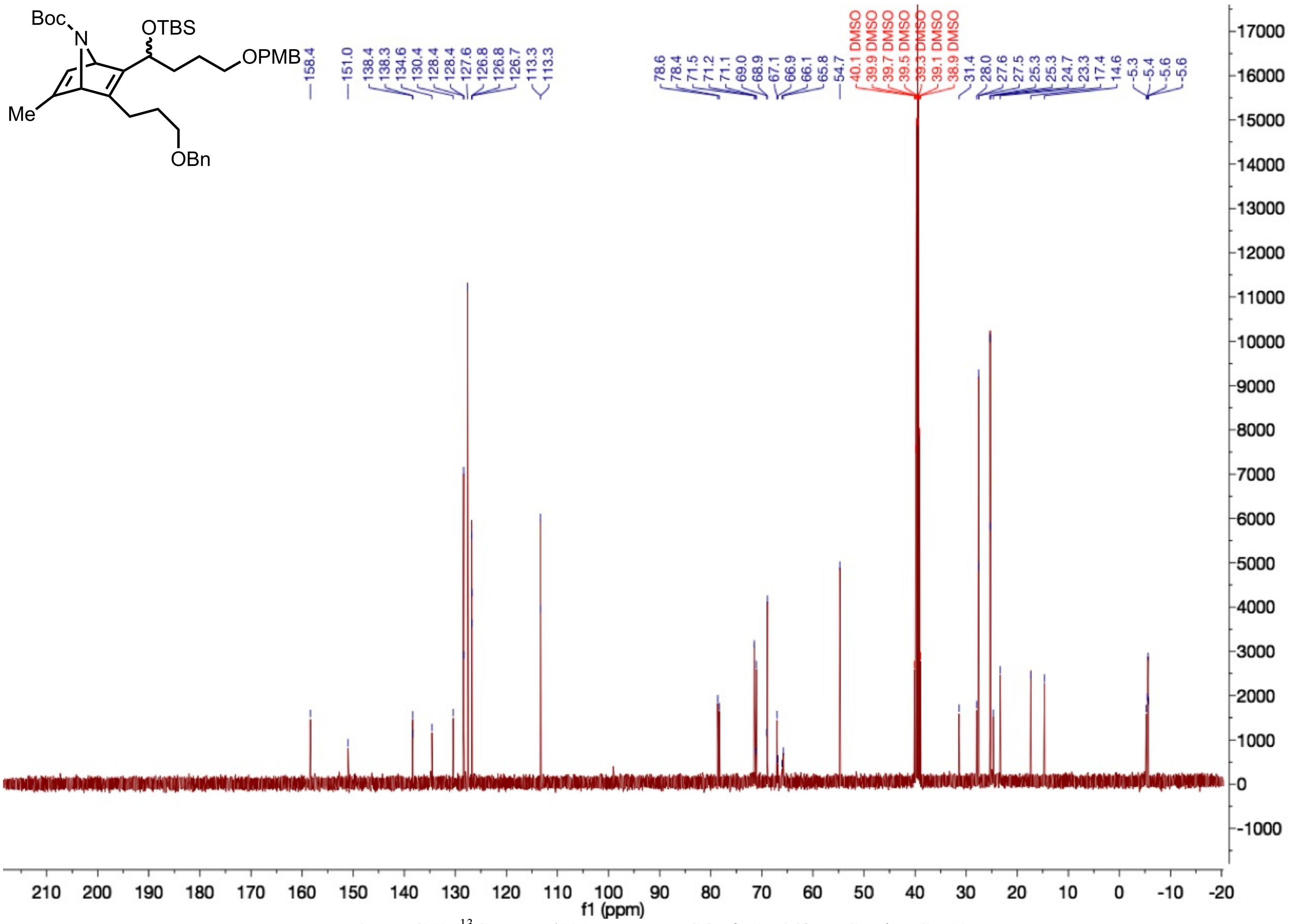

Figure S62. ^{13}C NMR (101 MHz, DMSO-d_6, 85 °C) TBS Ether **SI-13**

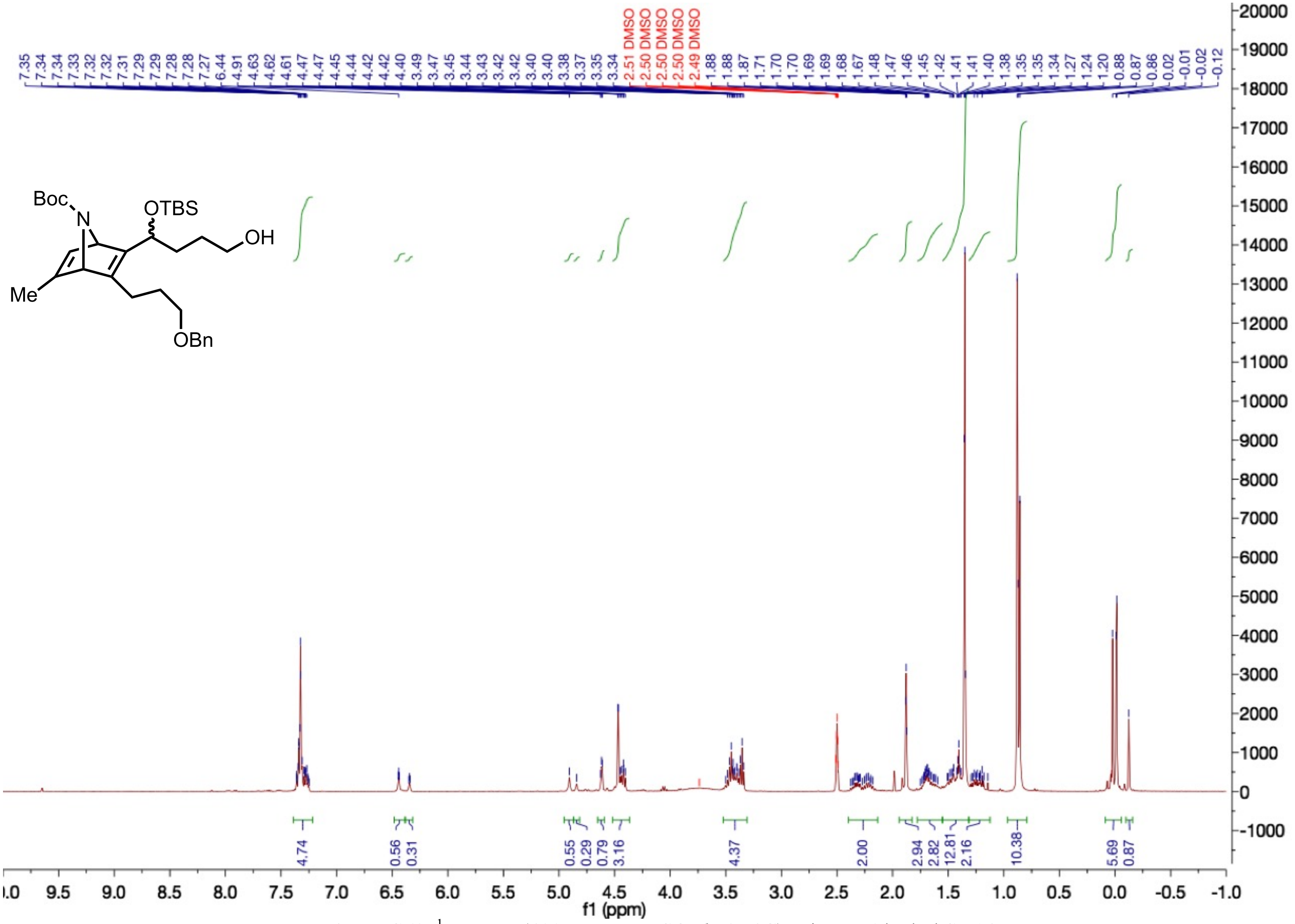

Figure S63. ^{1}H NMR (400 MHz, DMSO-d_6, 85 °C) Primary Alcohol **SI-14**

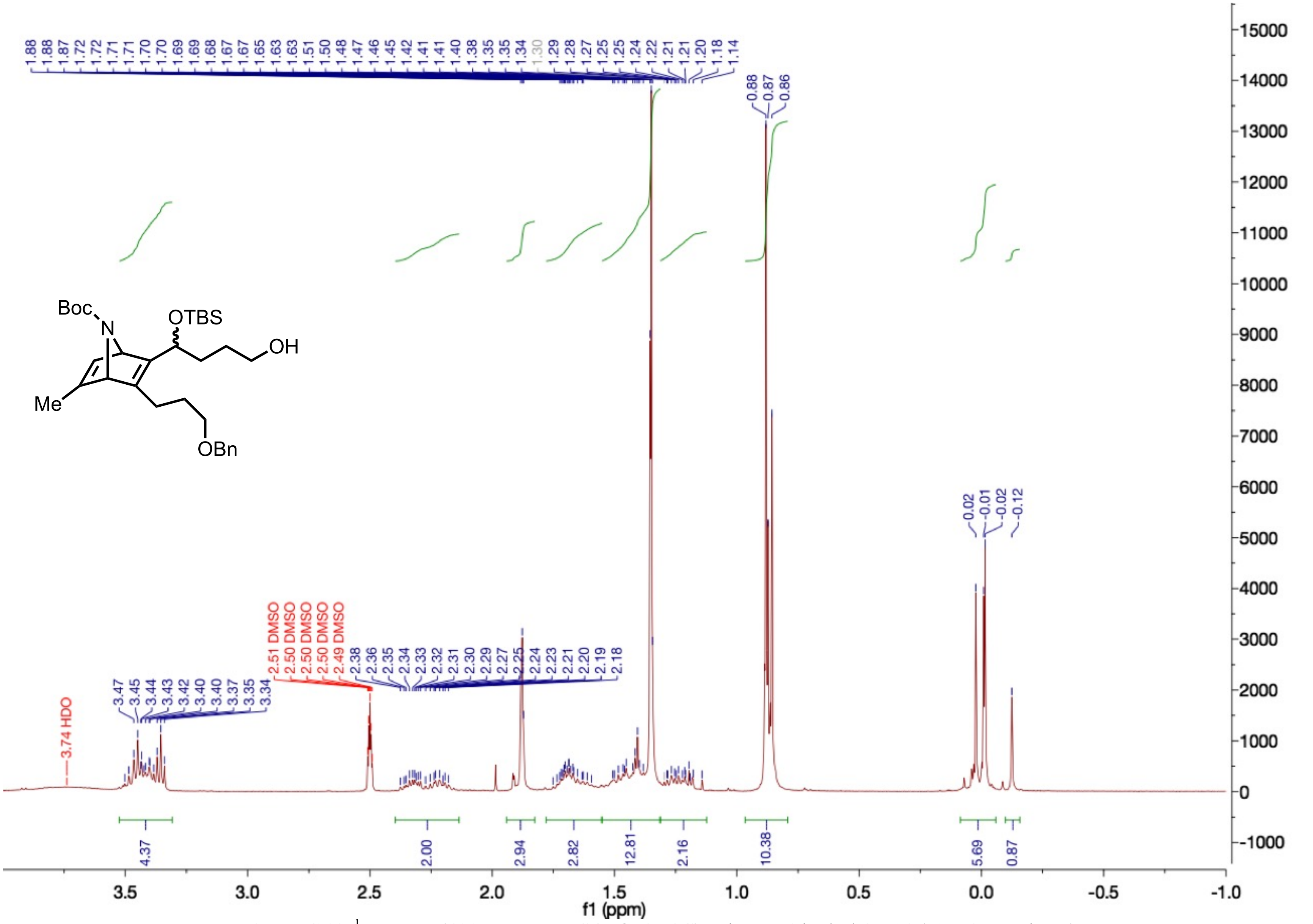

Figure S64. ^{1}H NMR (400 MHz, DMSO-d_6, 85 °C) Primary Alcohol **SI-14** (-1 – 4 ppm inset)

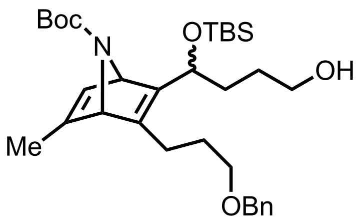

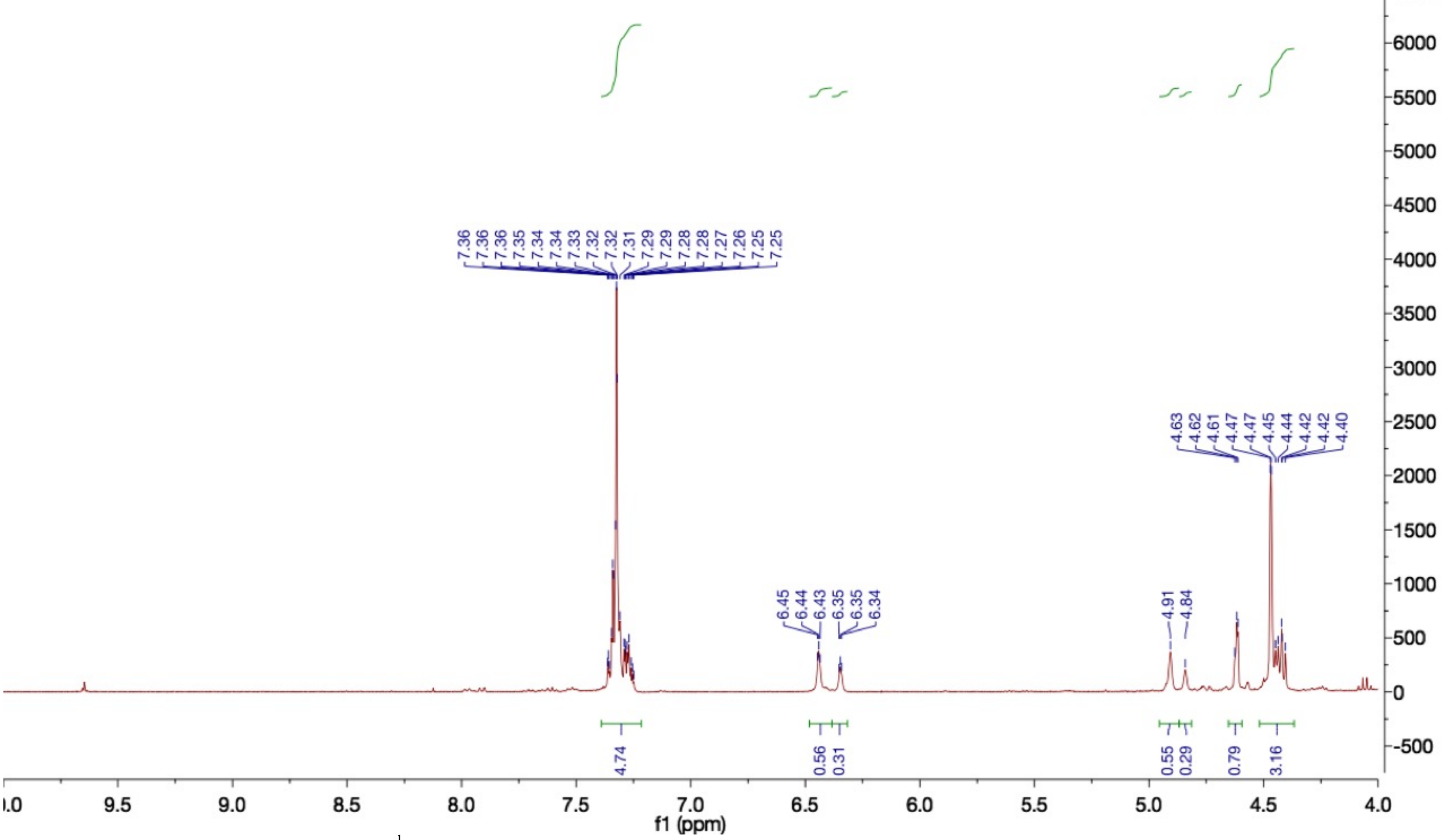

Figure S65. ^{1}H NMR (400 MHz, DMSO-d_6, 85 °C) Primary Alcohol **SI-14** (4 – 10 ppm inset)

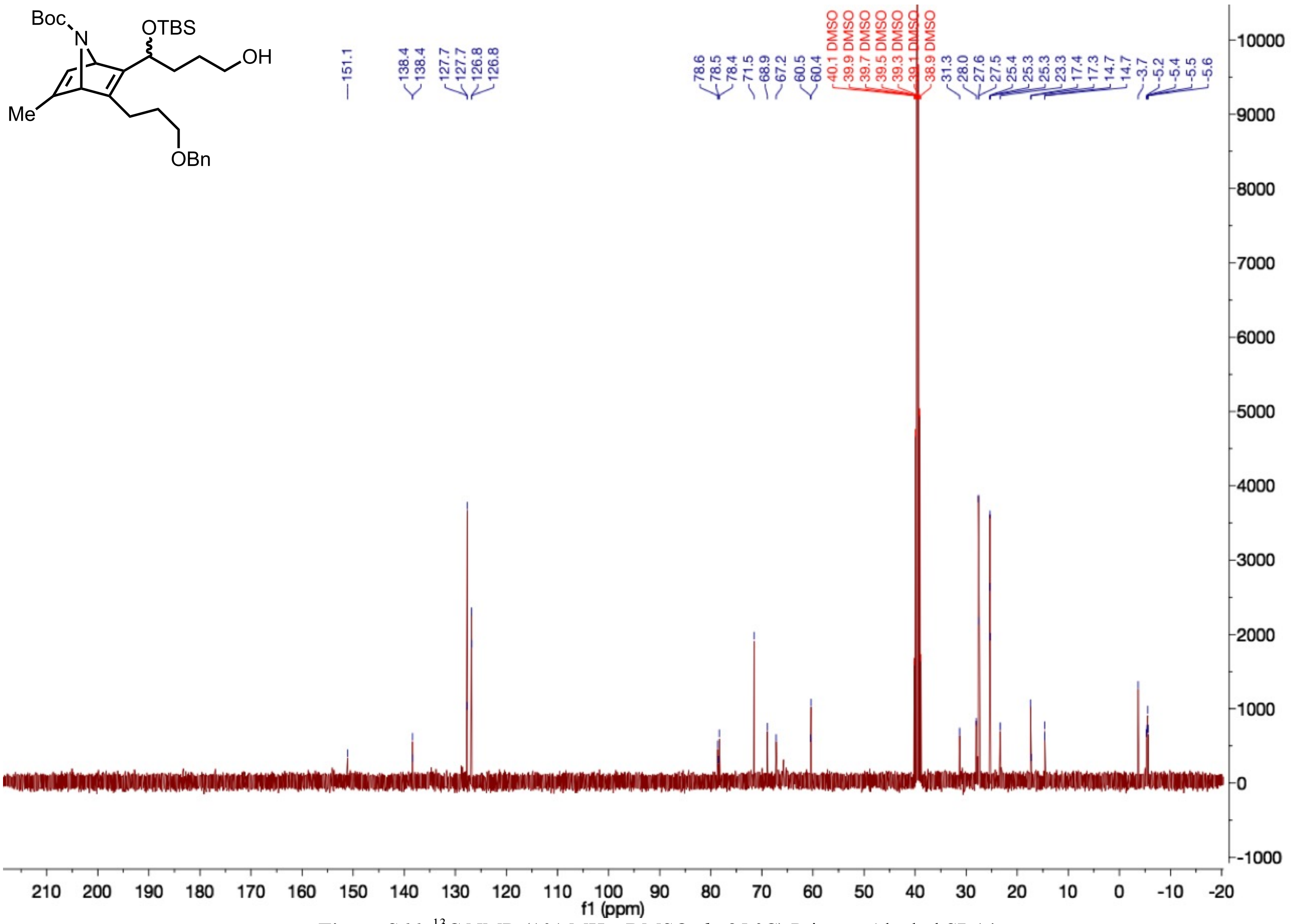

Figure S66. ^{13}C NMR (101 MHz, DMSO-d_6, 85 °C) Primary Alcohol **SI-14**

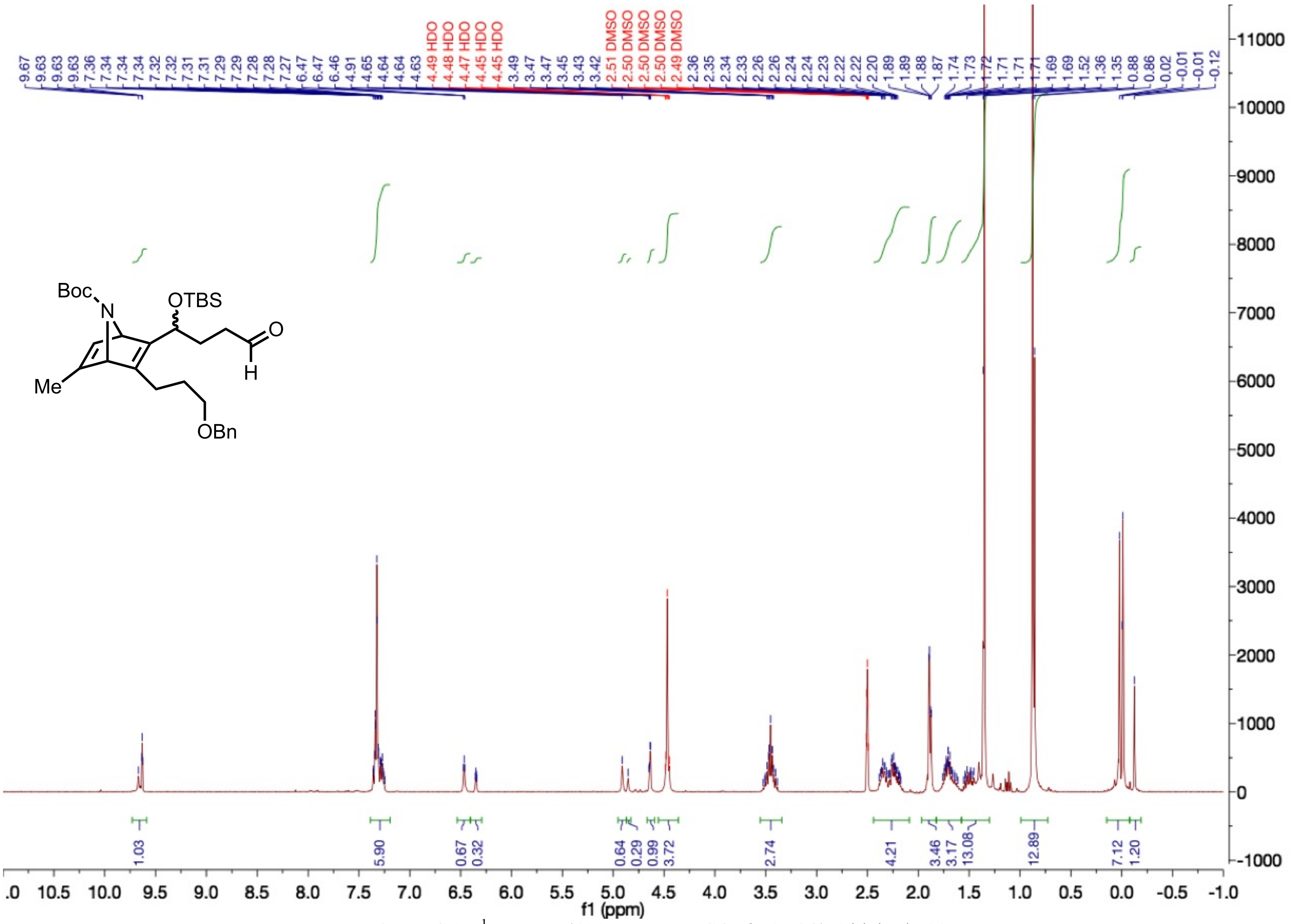

Figure S67. ^{1}H NMR (400 MHz, DMSO-d_6, 85 °C) Aldehyde **29**

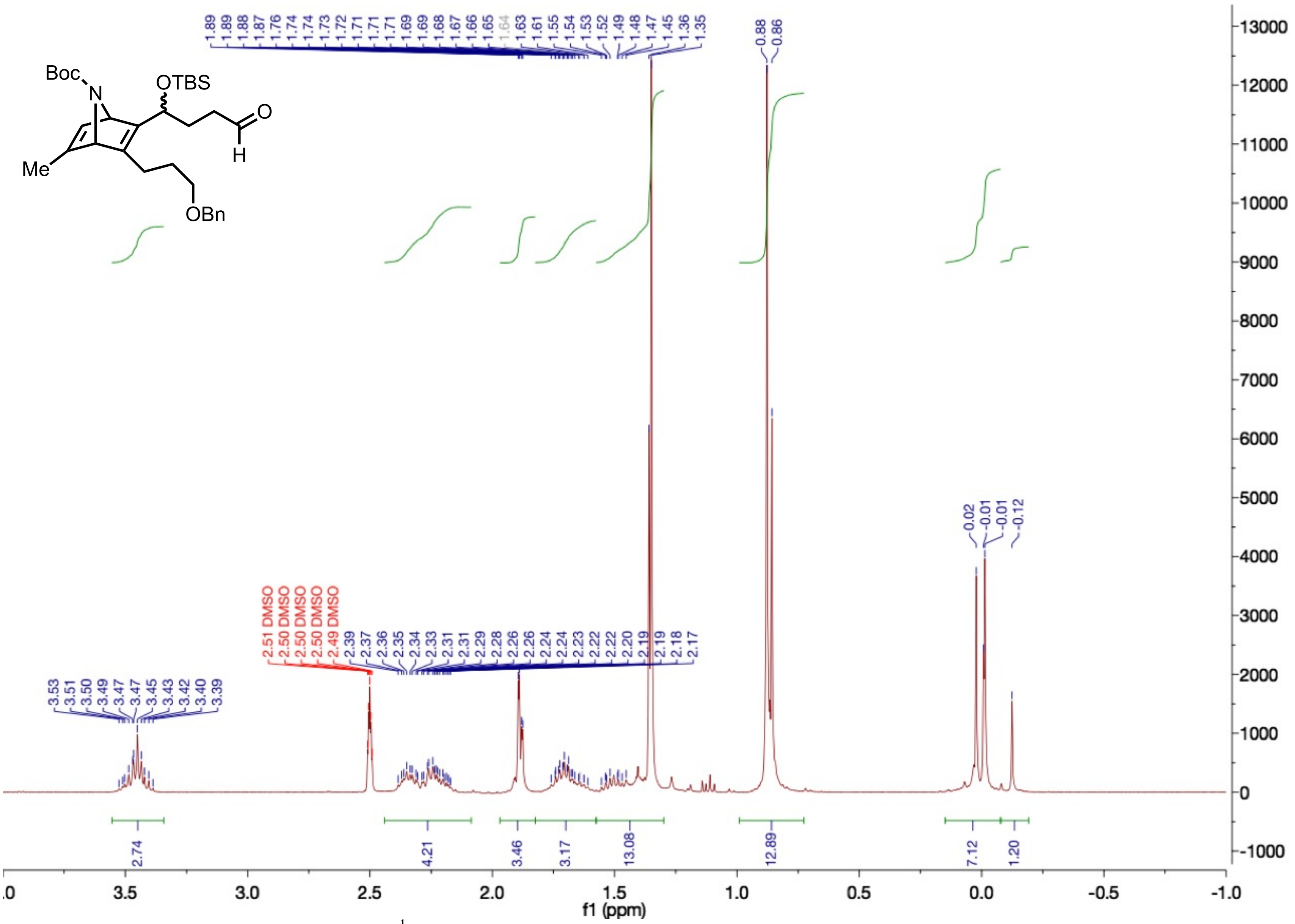

Figure S68. ^1H NMR (400 MHz, DMSO-d_6, 85 °C) Aldehyde **29** (-1 – 4 ppm inset)

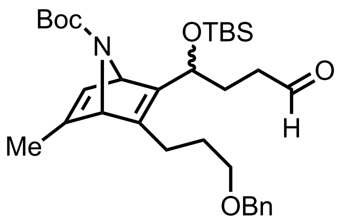

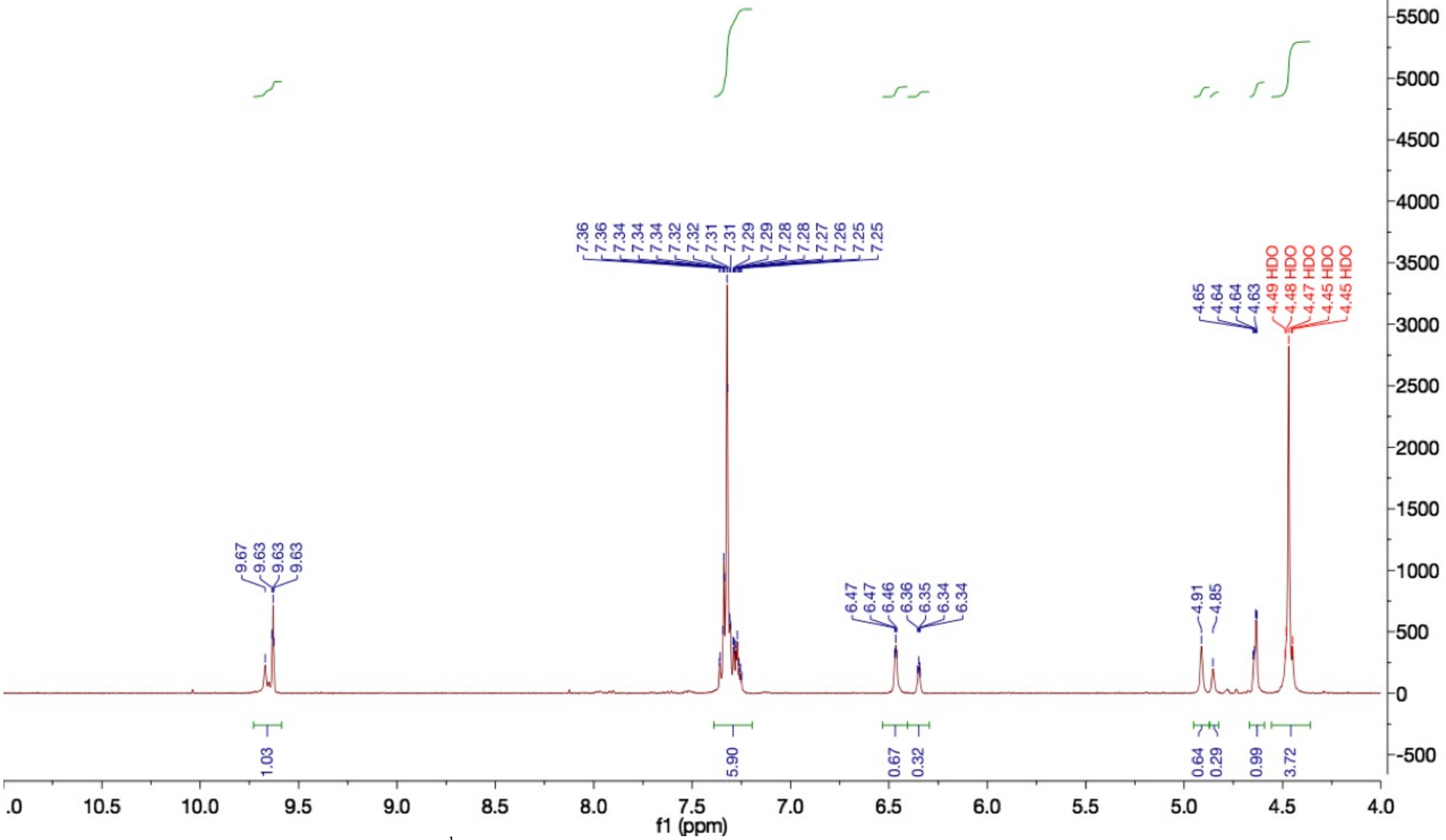

Figure S69. ^{1}H NMR (400 MHz, DMSO-d_6, 85 °C) Aldehyde **29** (4 – 11 ppm inset)

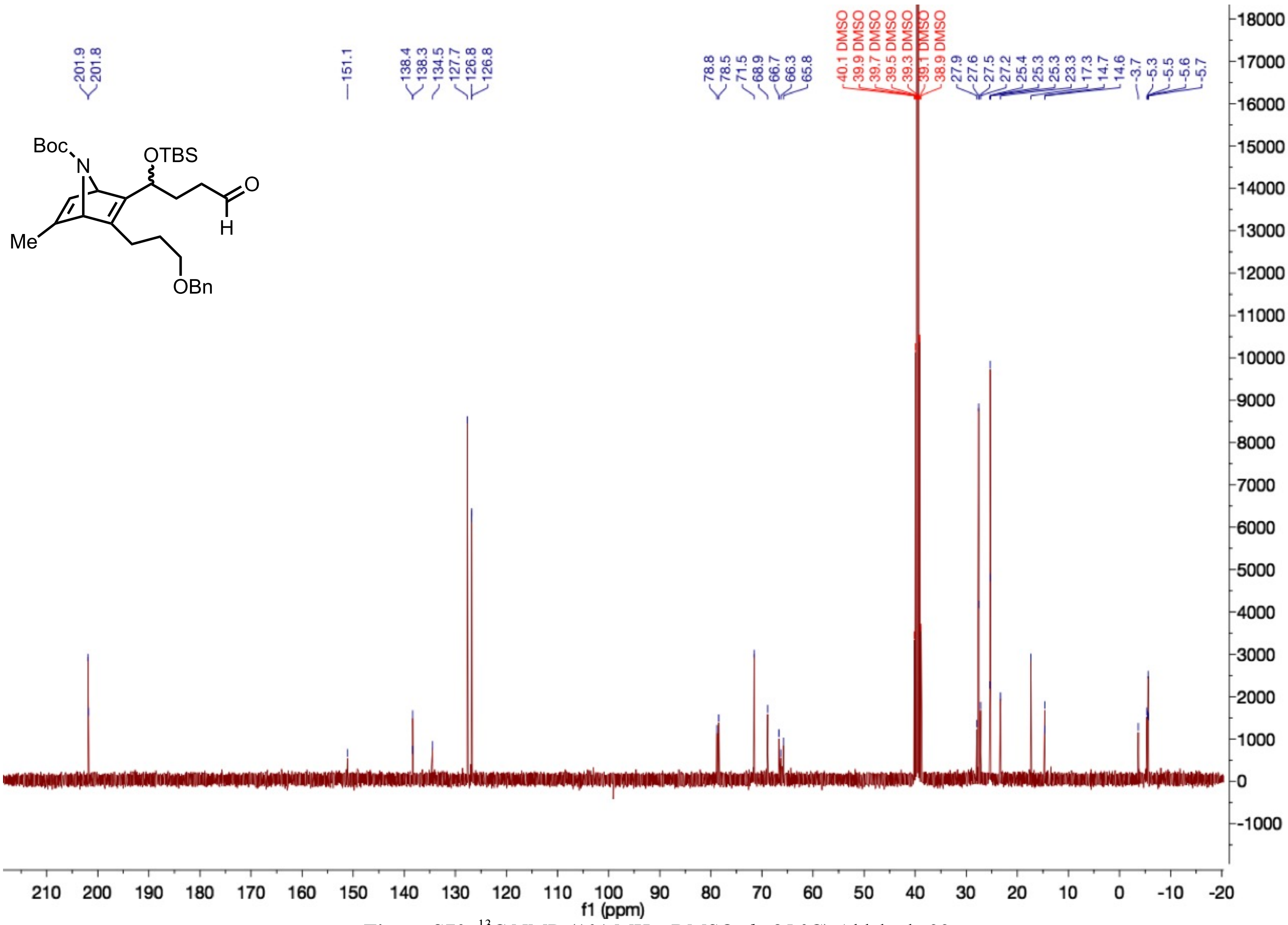

Figure S70. ^{13}C NMR (101 MHz, DMSO-d_6, 85 °C) Aldehyde **29**

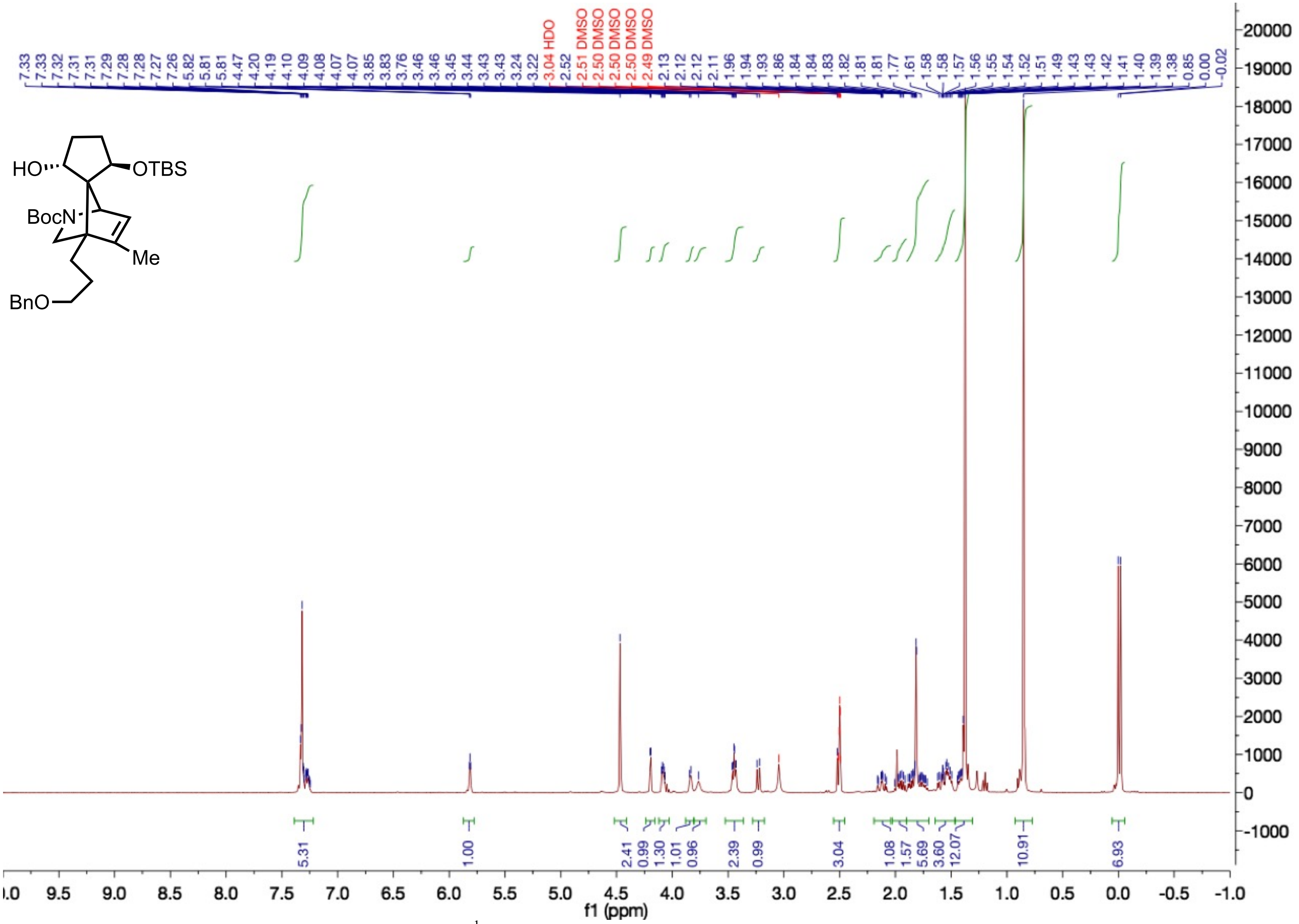

Figure S71. ^{1}H NMR (400 MHz, DMSO-d_6, 85 °C) Spirocycle **48a**

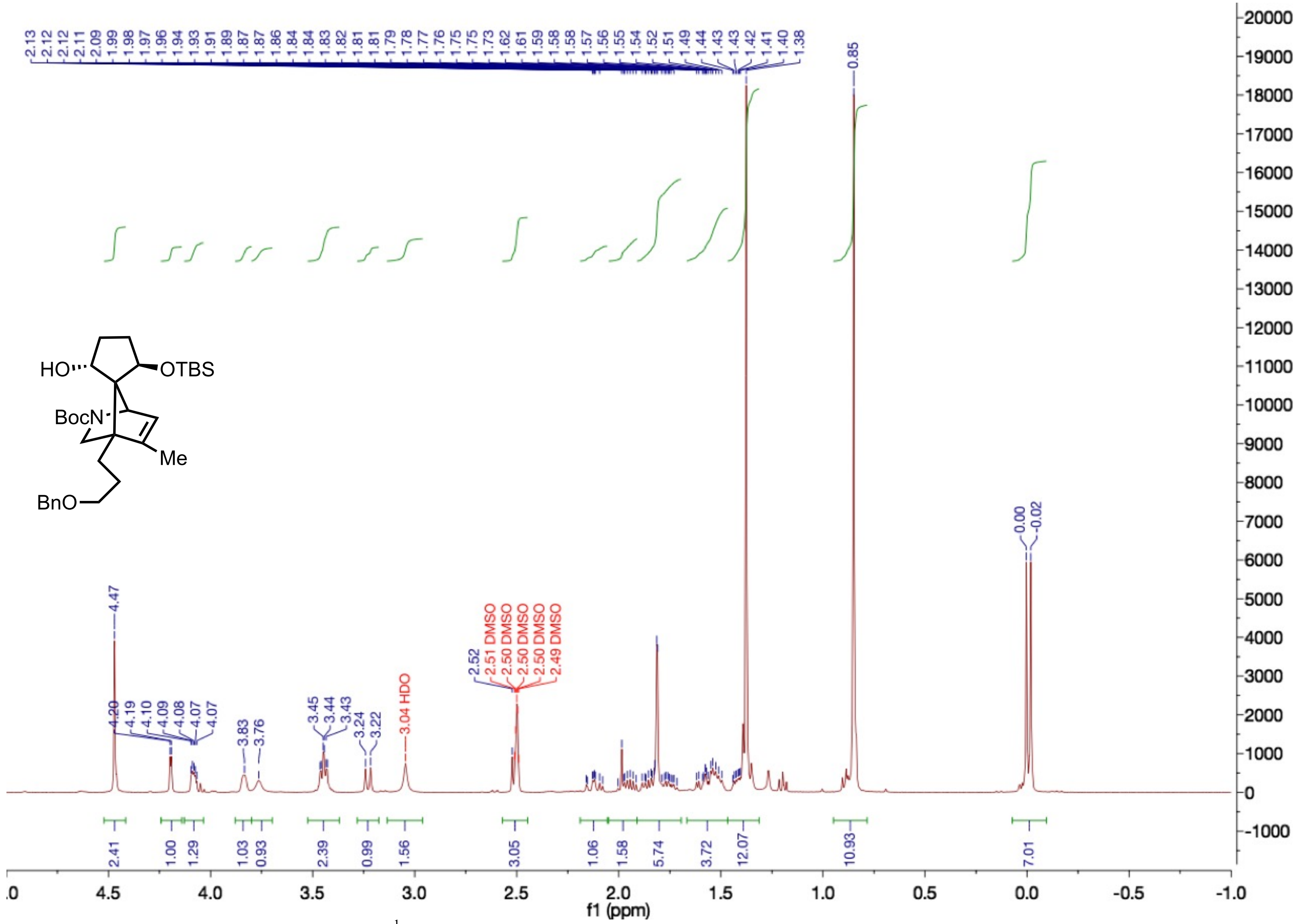

Figure S72. ^{1}H NMR (400 MHz, DMSO-d_6, 85 °C) Spirocycle **48a** (-1 – 5 ppm inset)

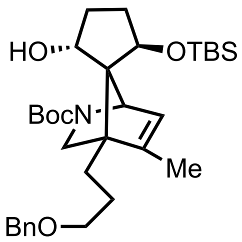

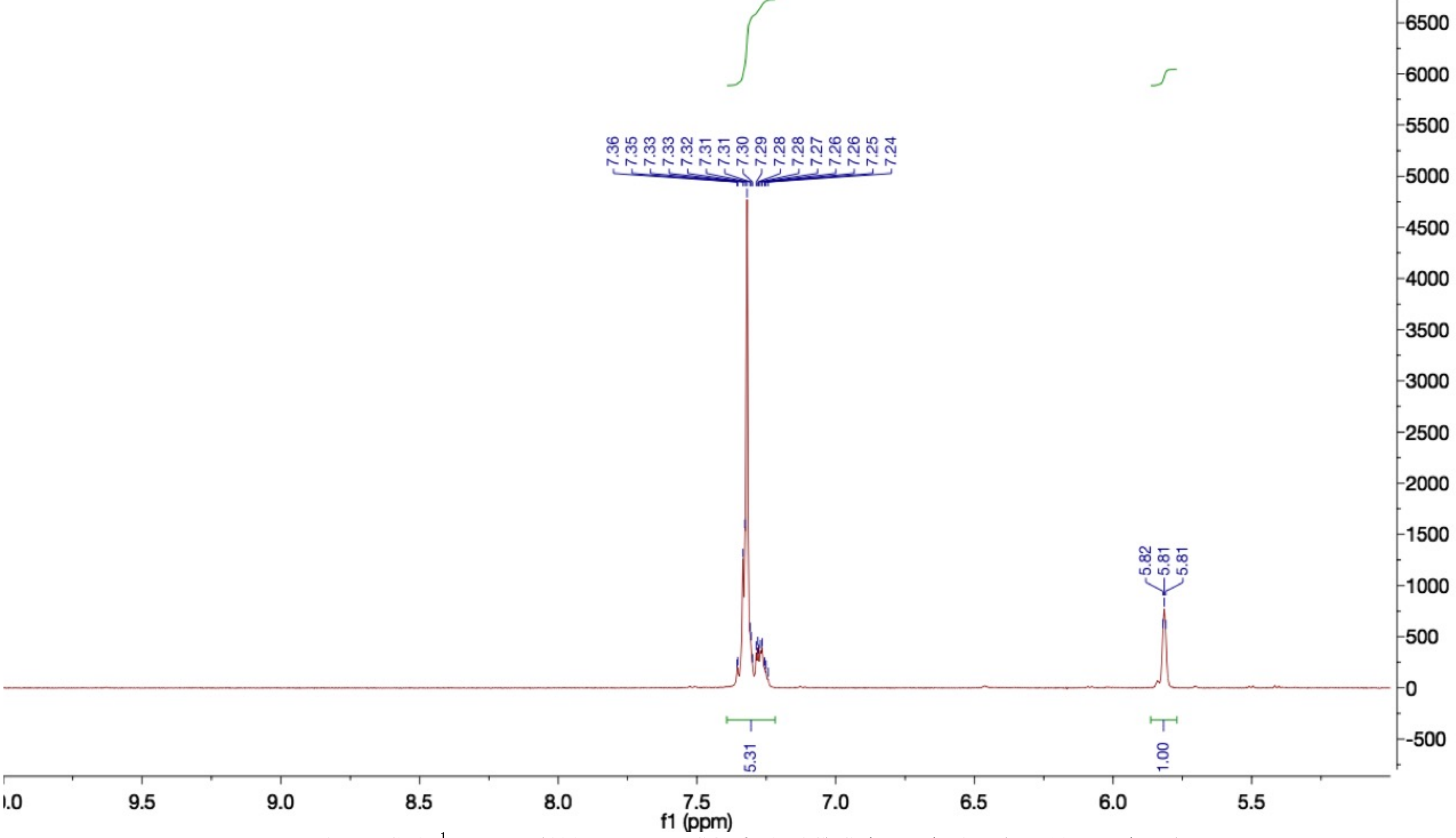

Figure S73. ^{1}H NMR (400 MHz, DMSO-d_6, 85 °C) Spirocycle **48a** (5 – 10 ppm inset)

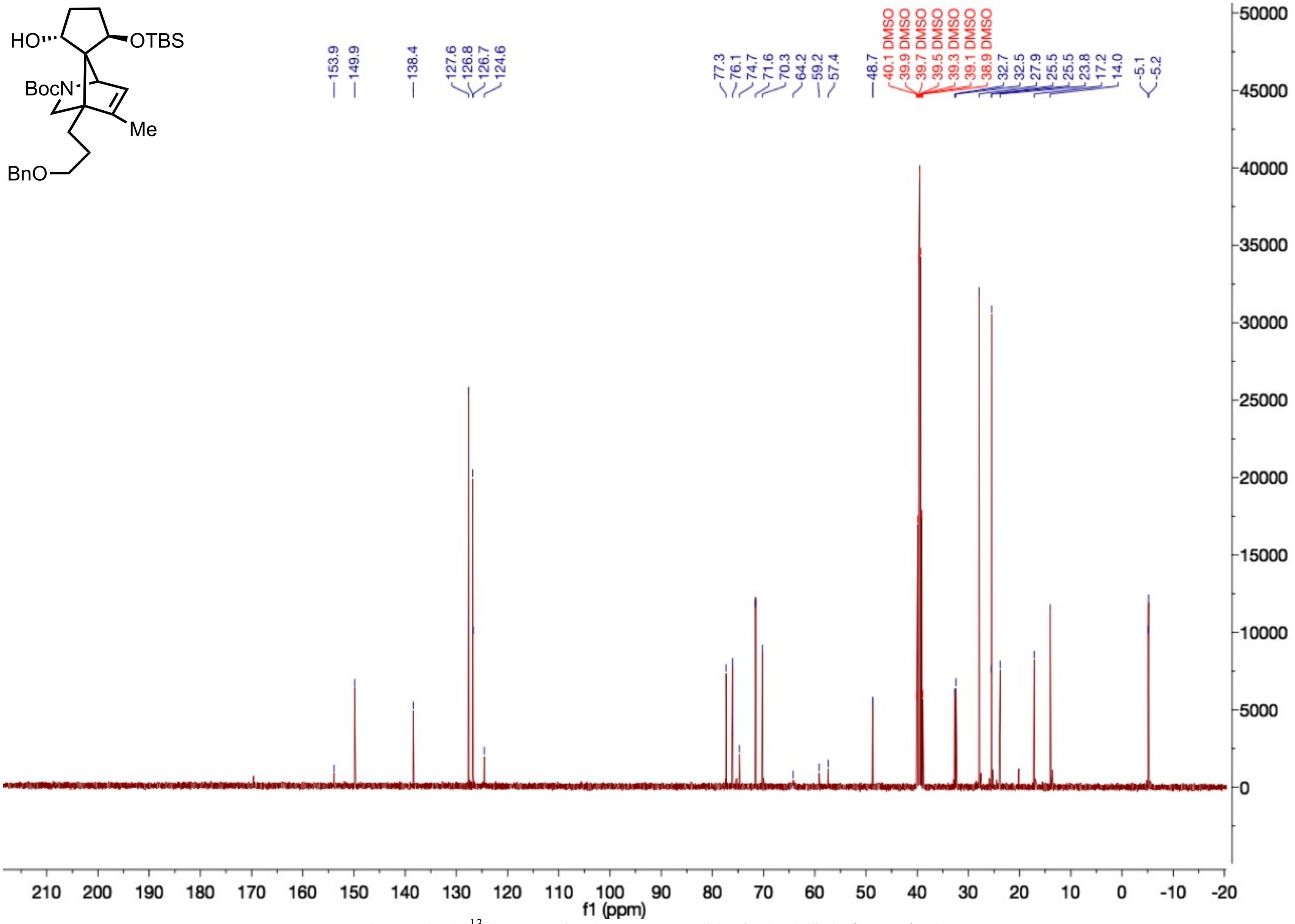

Figure S74. ^{13}C NMR (101 MHz, DMSO-d_6, 85 °C) Spirocycle **48a**

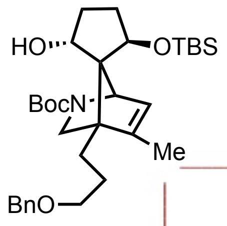

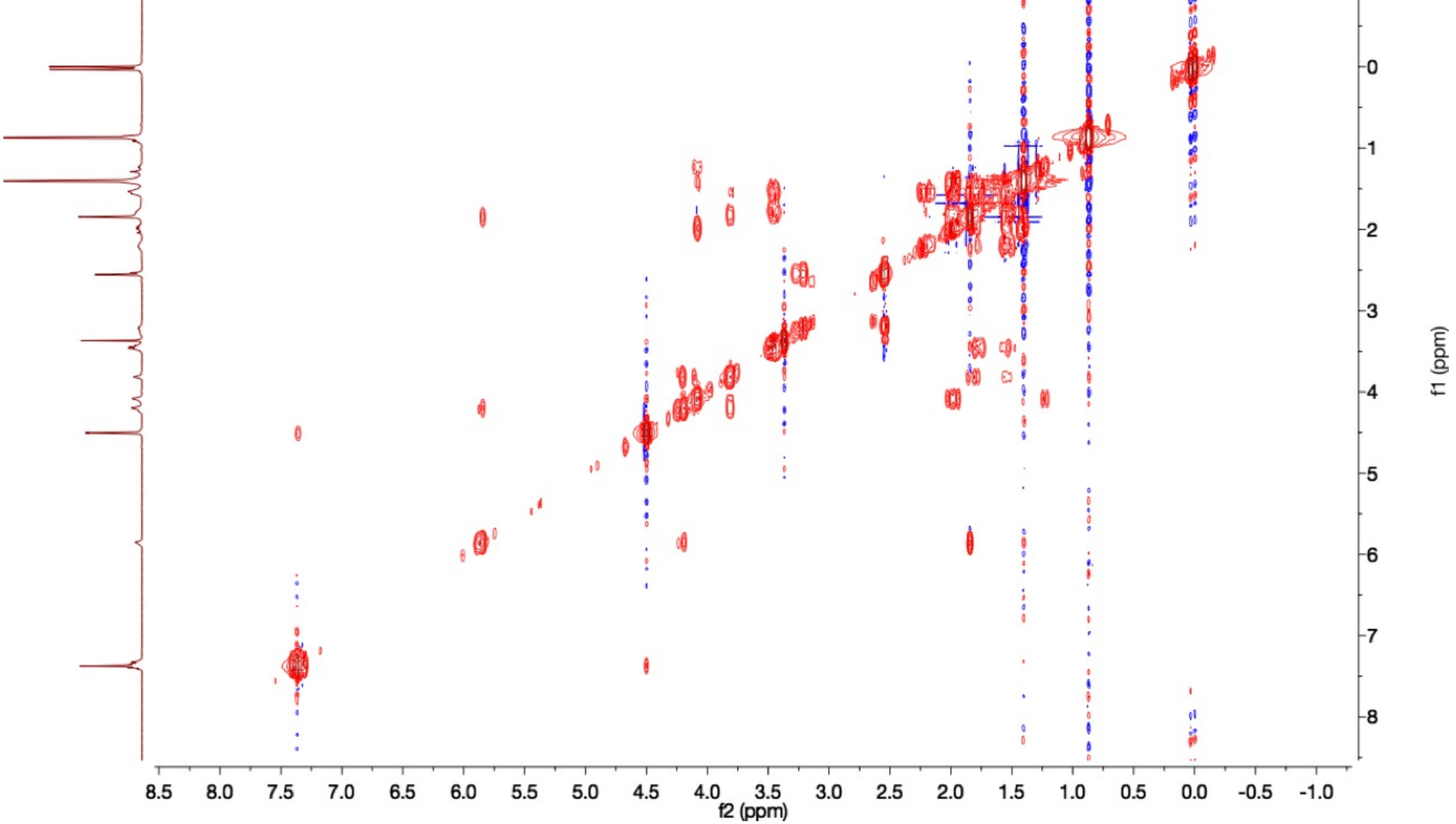

Figure S75. COSY (DMSO-d_6, 27 °C) Spirocycle **48a**

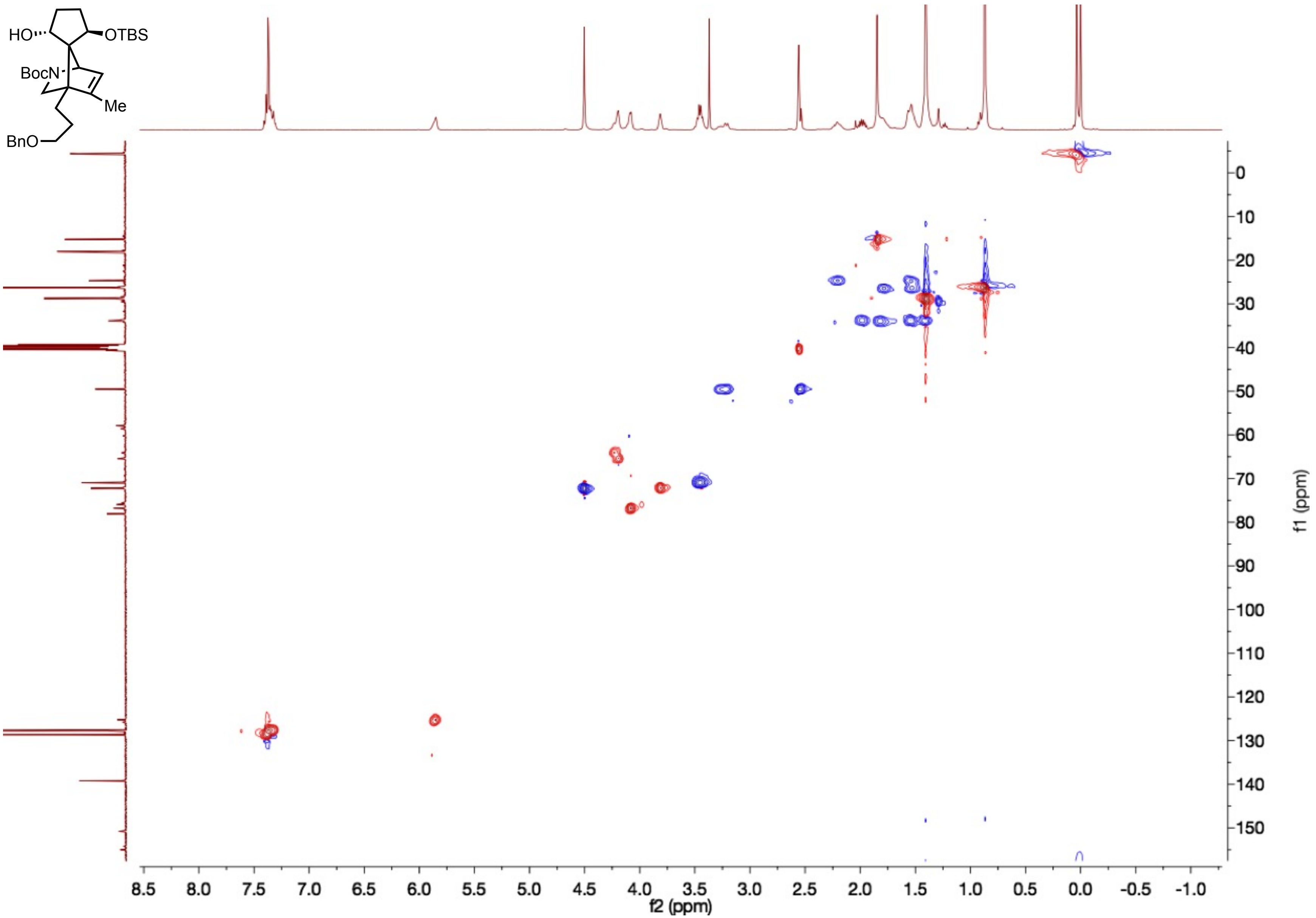

Figure S76. HSQC (DMSO-d_6, 27 °C) Spirocycle **48a**

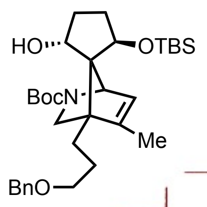

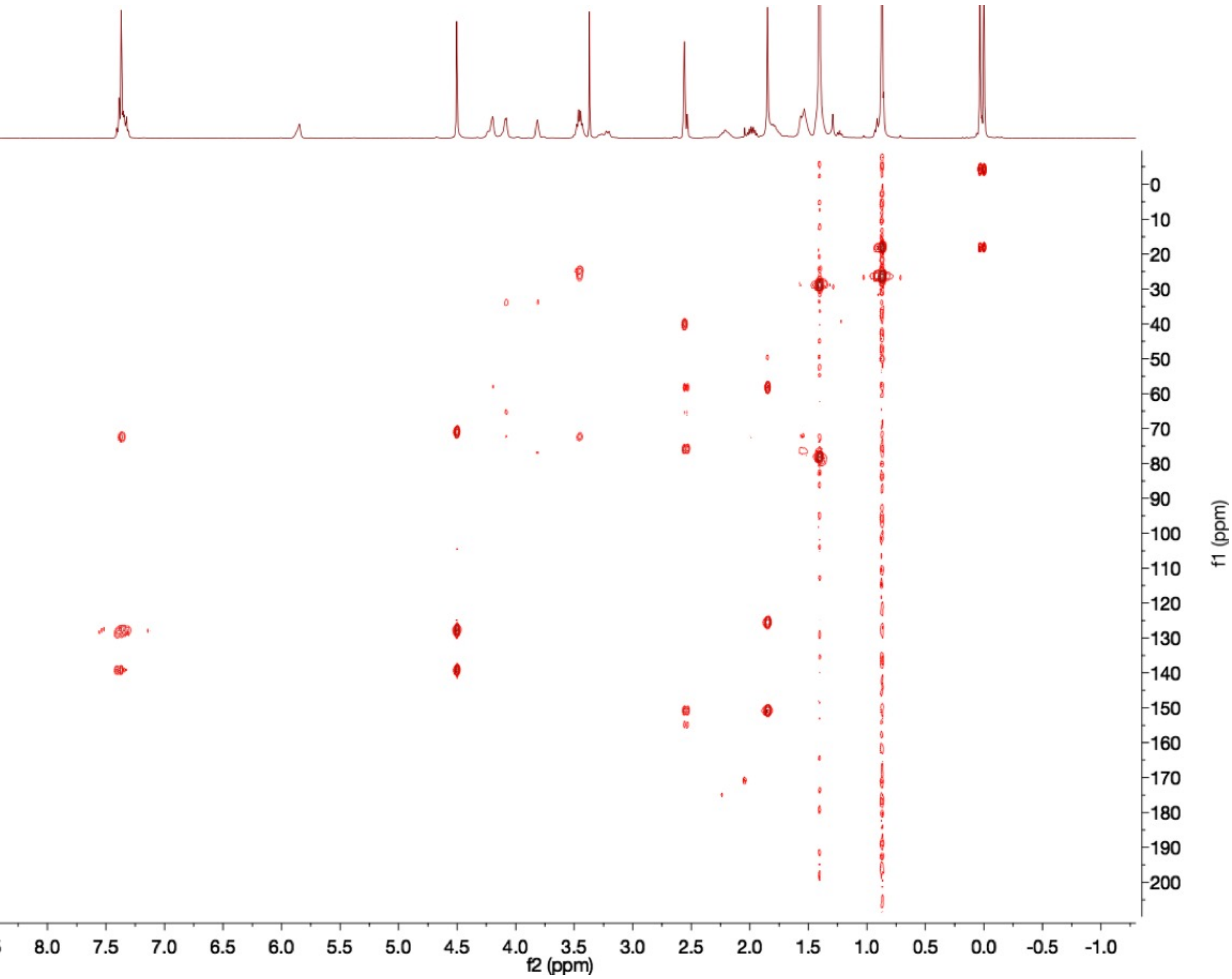

Figure S77. HMBC (DMSO-d_6, 27 °C) Spirocycle **48a**

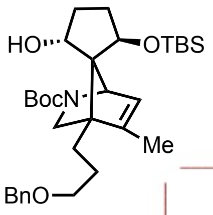

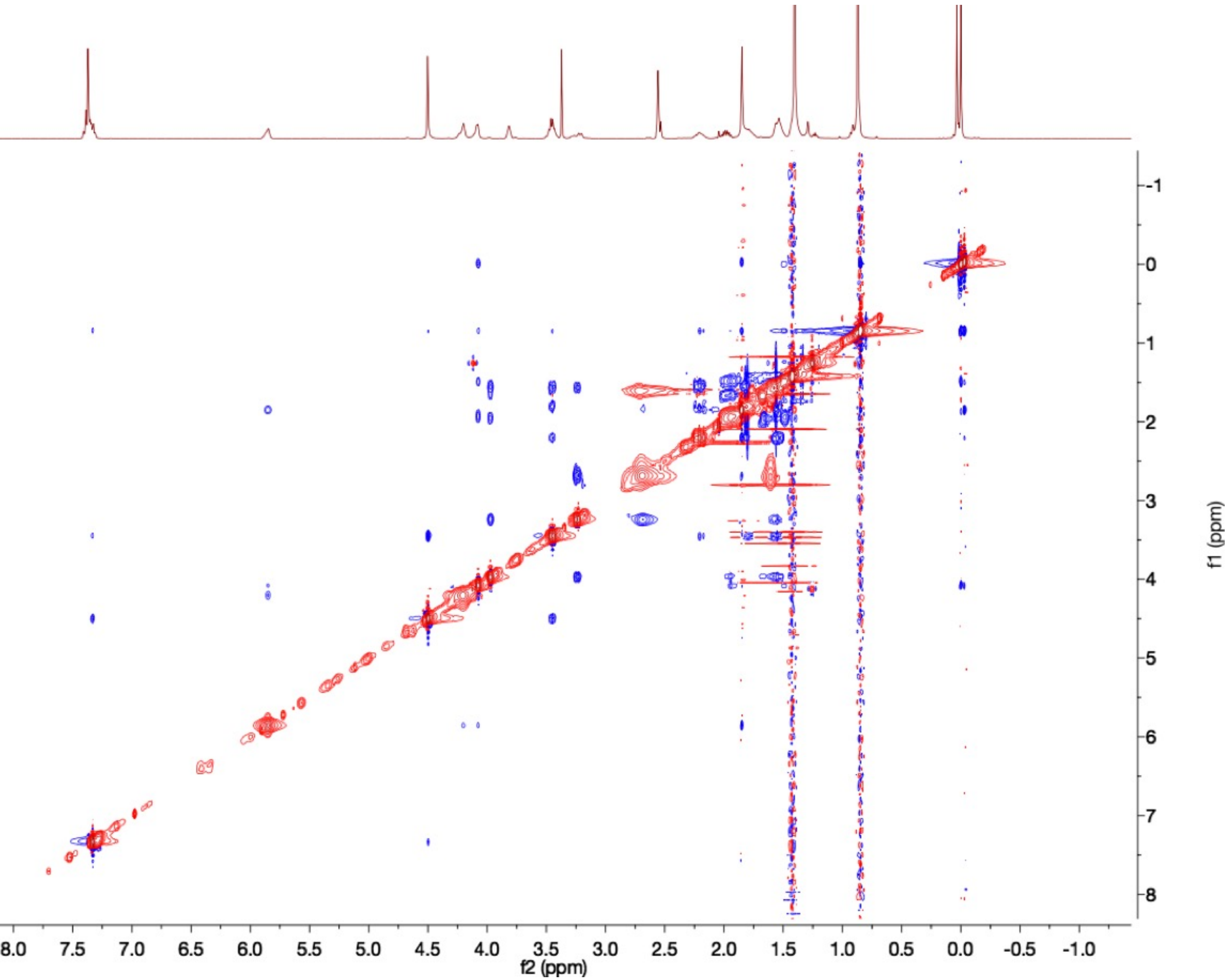

Figure S78. NOESY (DMSO-d_6, 27 °C) Spirocycle **48a**

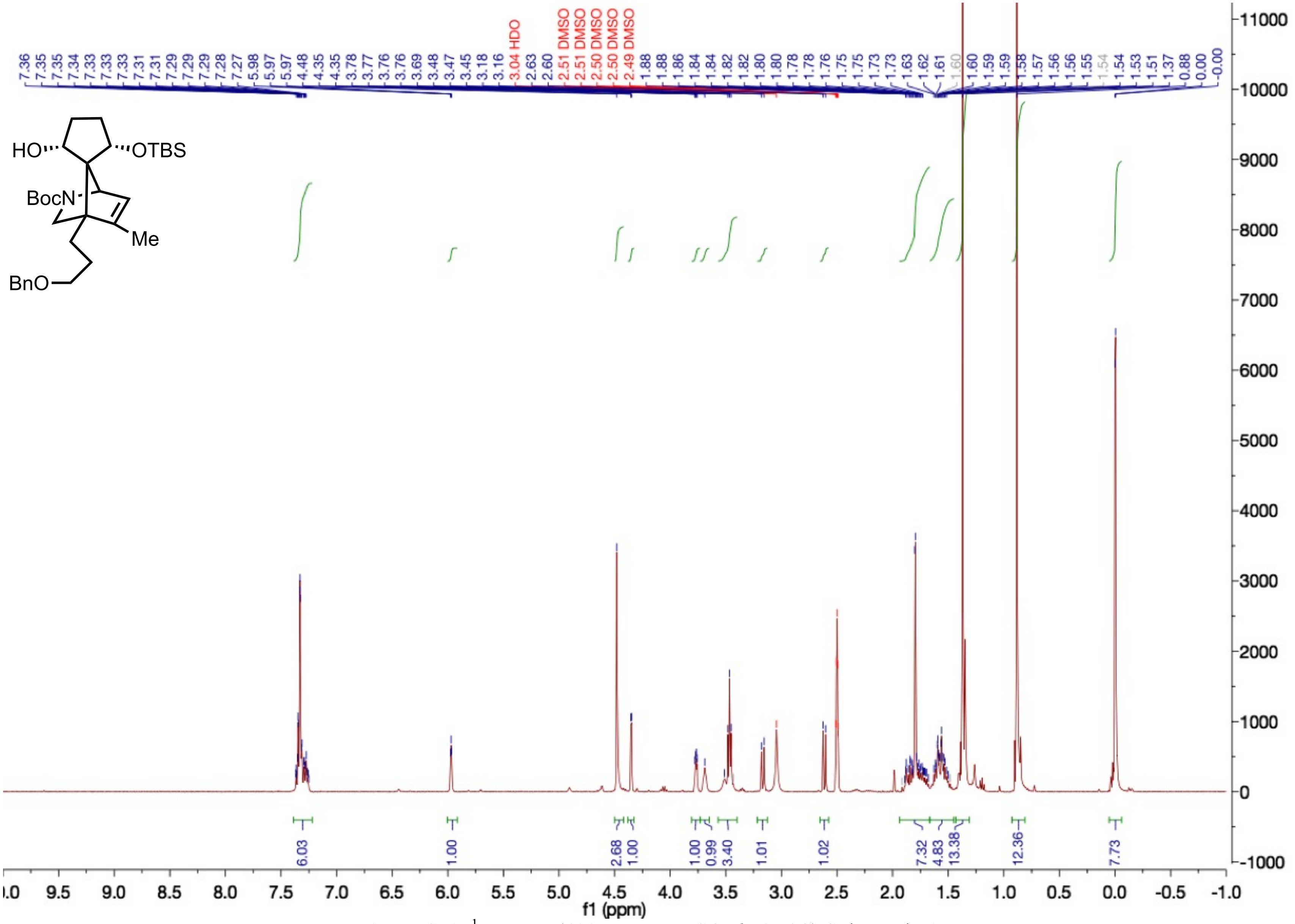

Figure S79. ^{1}H NMR (400 MHz, DMSO-d_6, 85 °C) Spirocycle **48b**

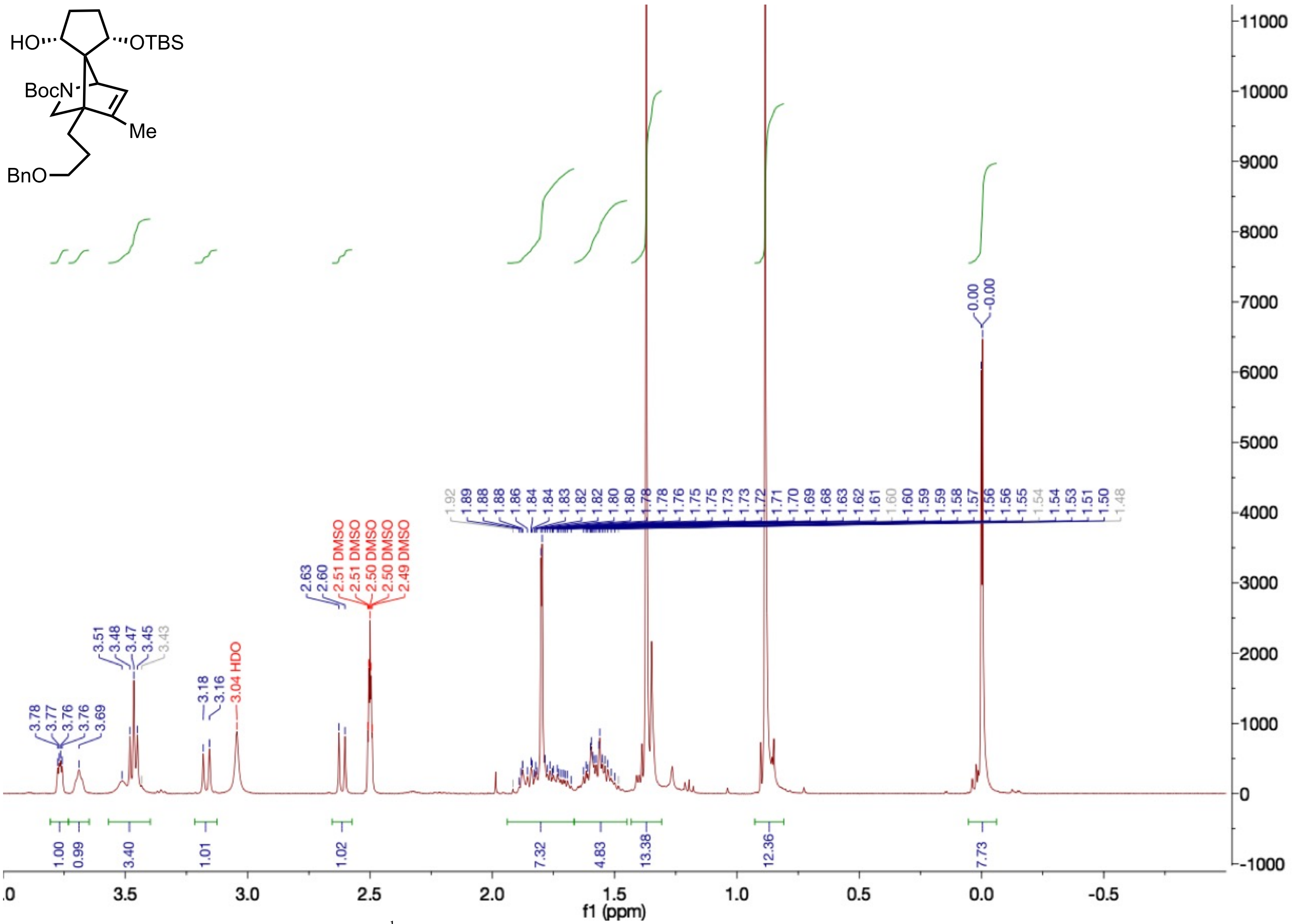

Figure S80. ^{1}H NMR (400 MHz, DMSO-d_6, 85 °C) Spirocycle **48b** (-1 – 4 ppm inset)

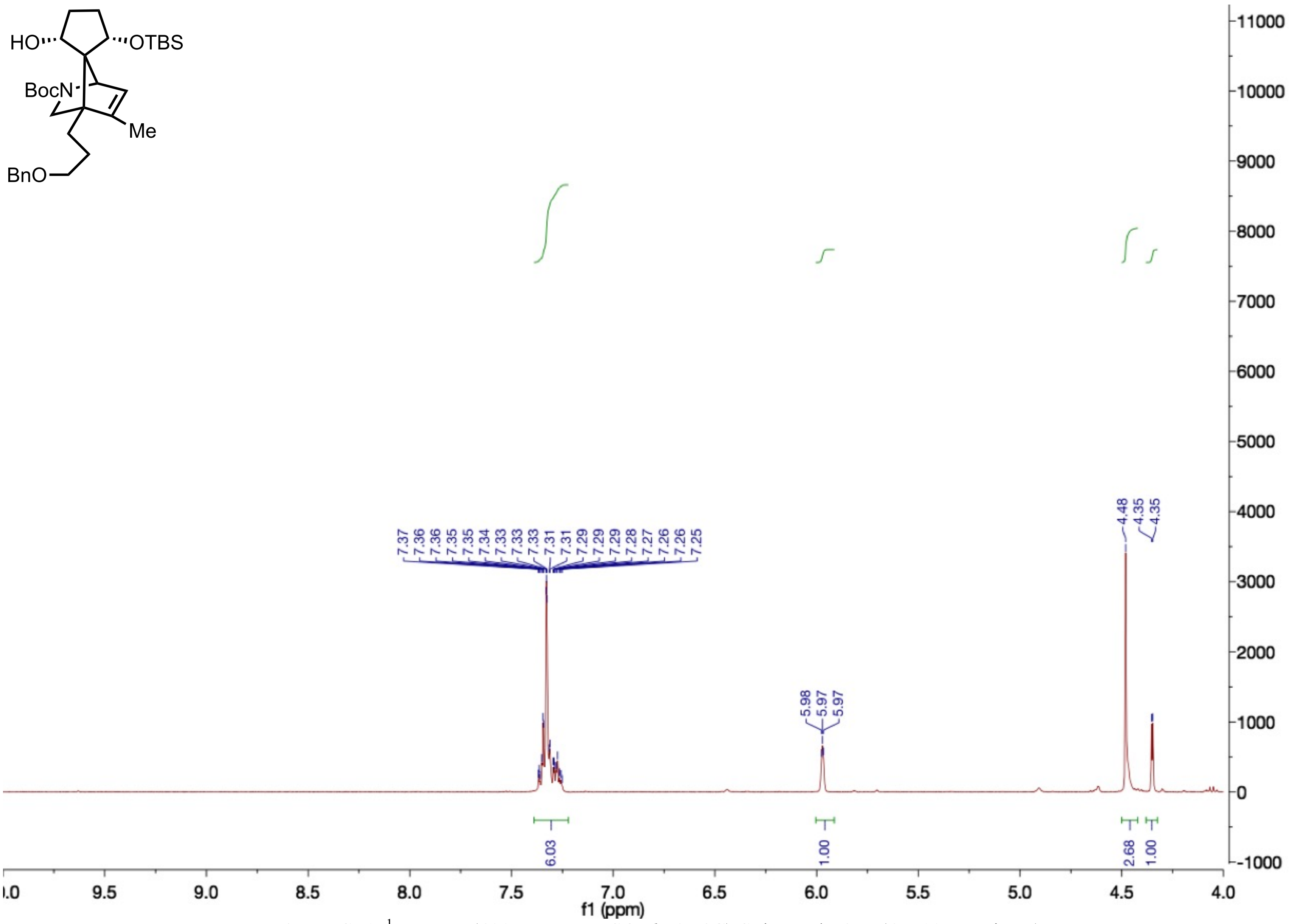

Figure S81. ^{1}H NMR (400 MHz, DMSO-d_6, 85 °C) Spirocycle **48b** (4 – 10 ppm inset)

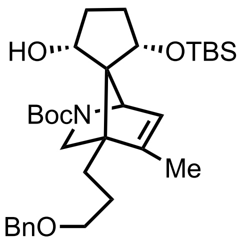

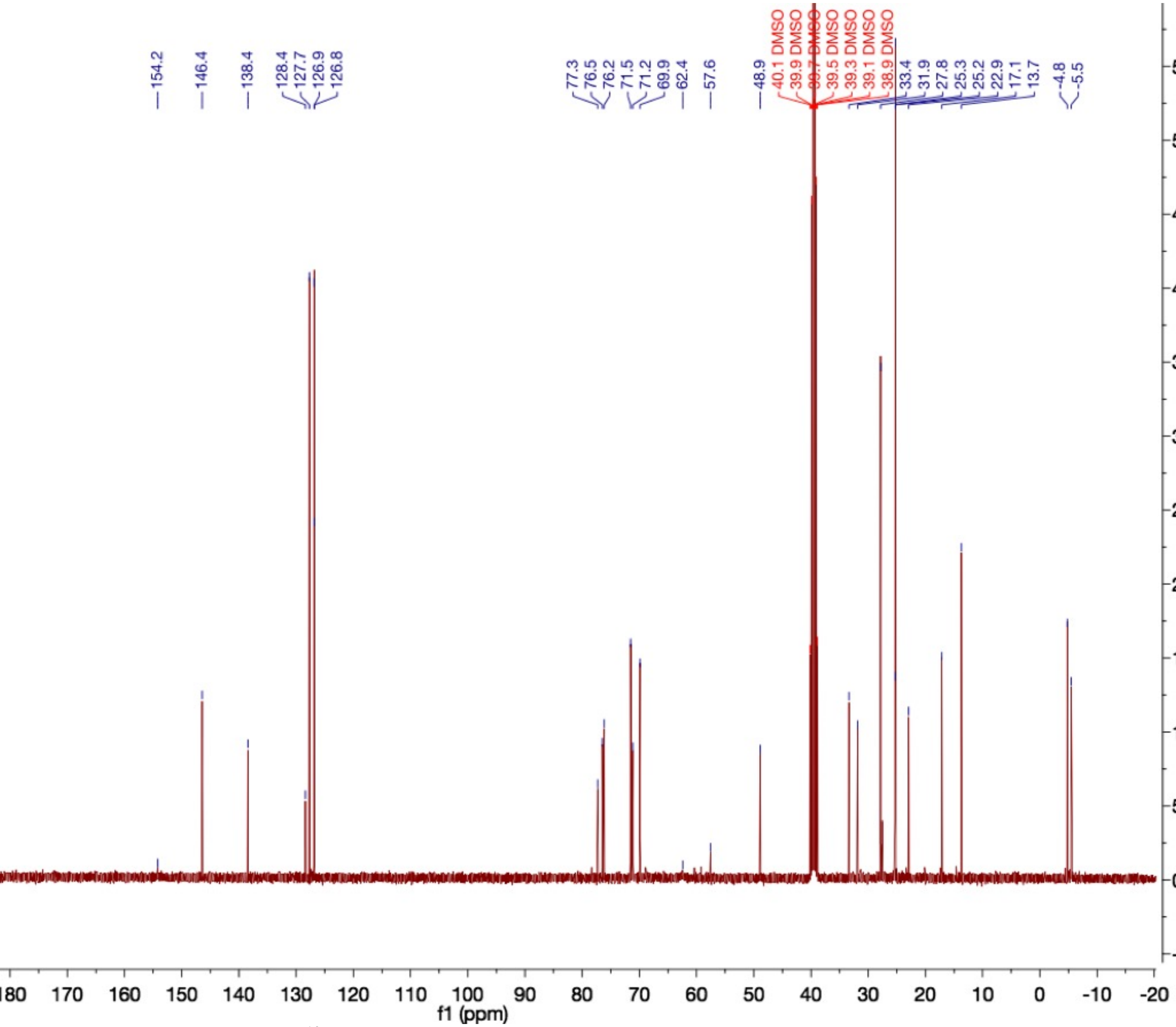

Figure S82. ^{13}C NMR (101 MHz, DMSO-d_6, 85 °C) Spirocycle **48b**

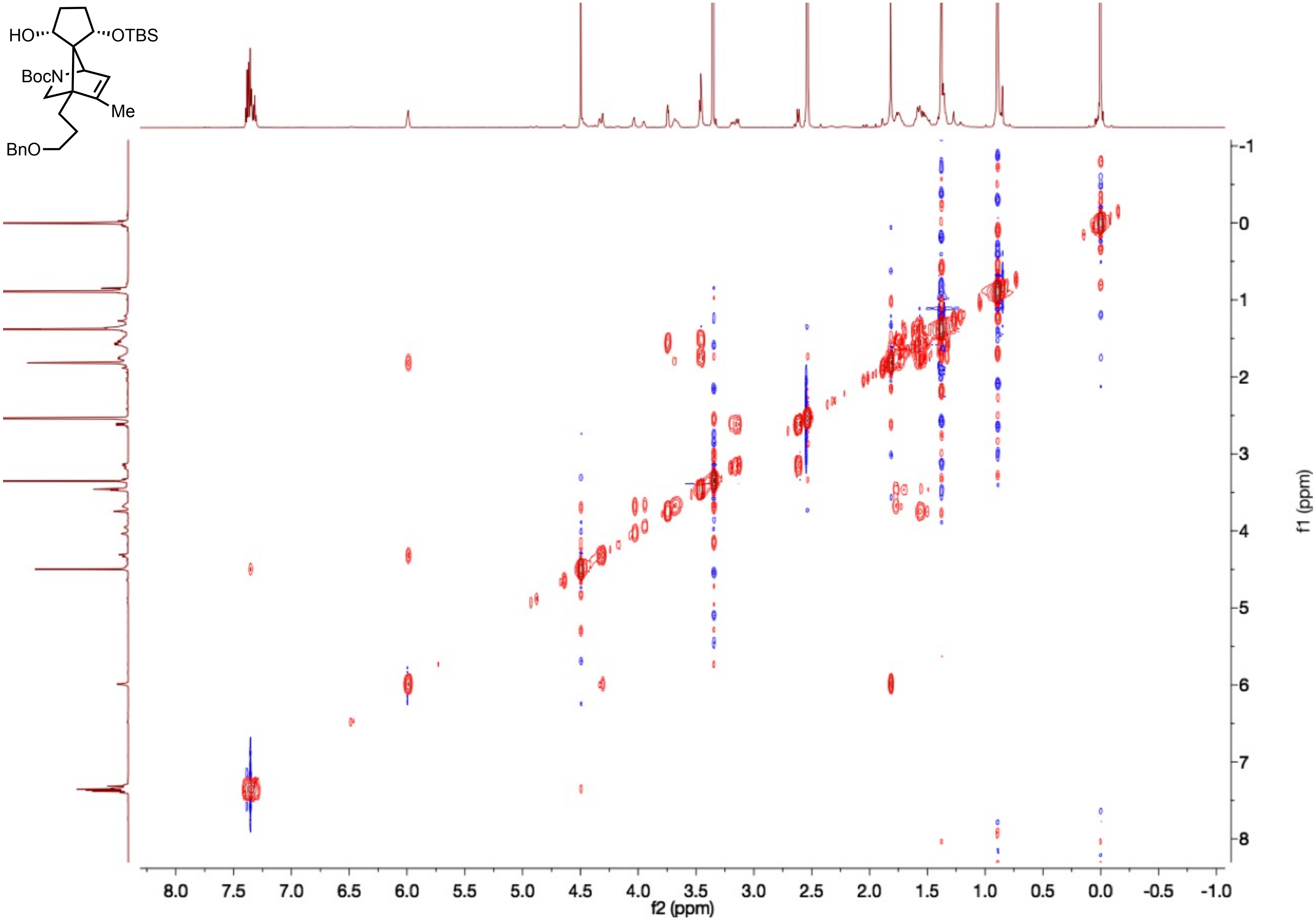

Figure S83. COSY (DMSO-d_6, 27 °C) Spirocycle **48b**

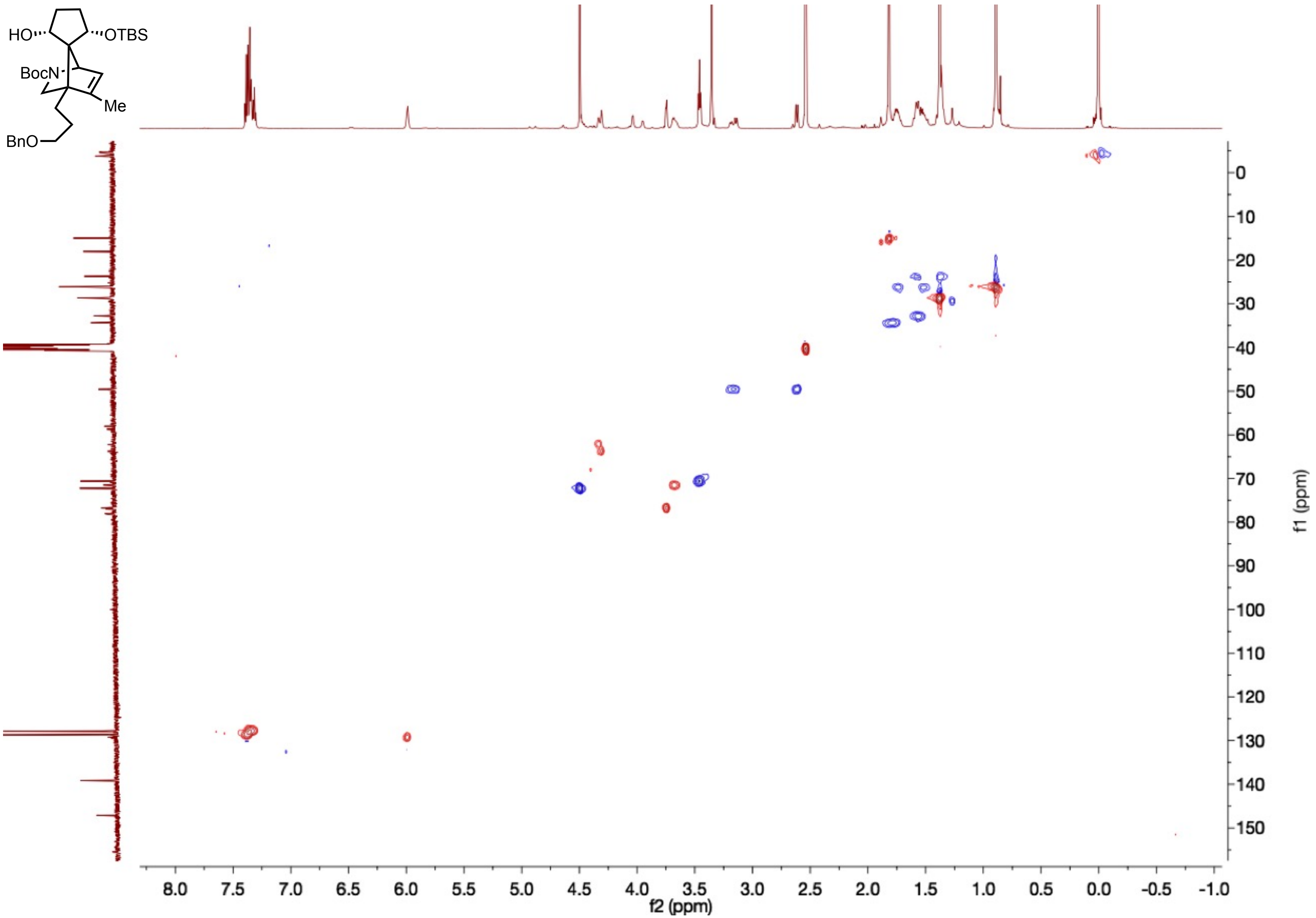

Figure S84. HSQC (DMSO-d_6, 27 °C) Spirocycle **48b**

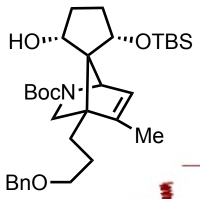

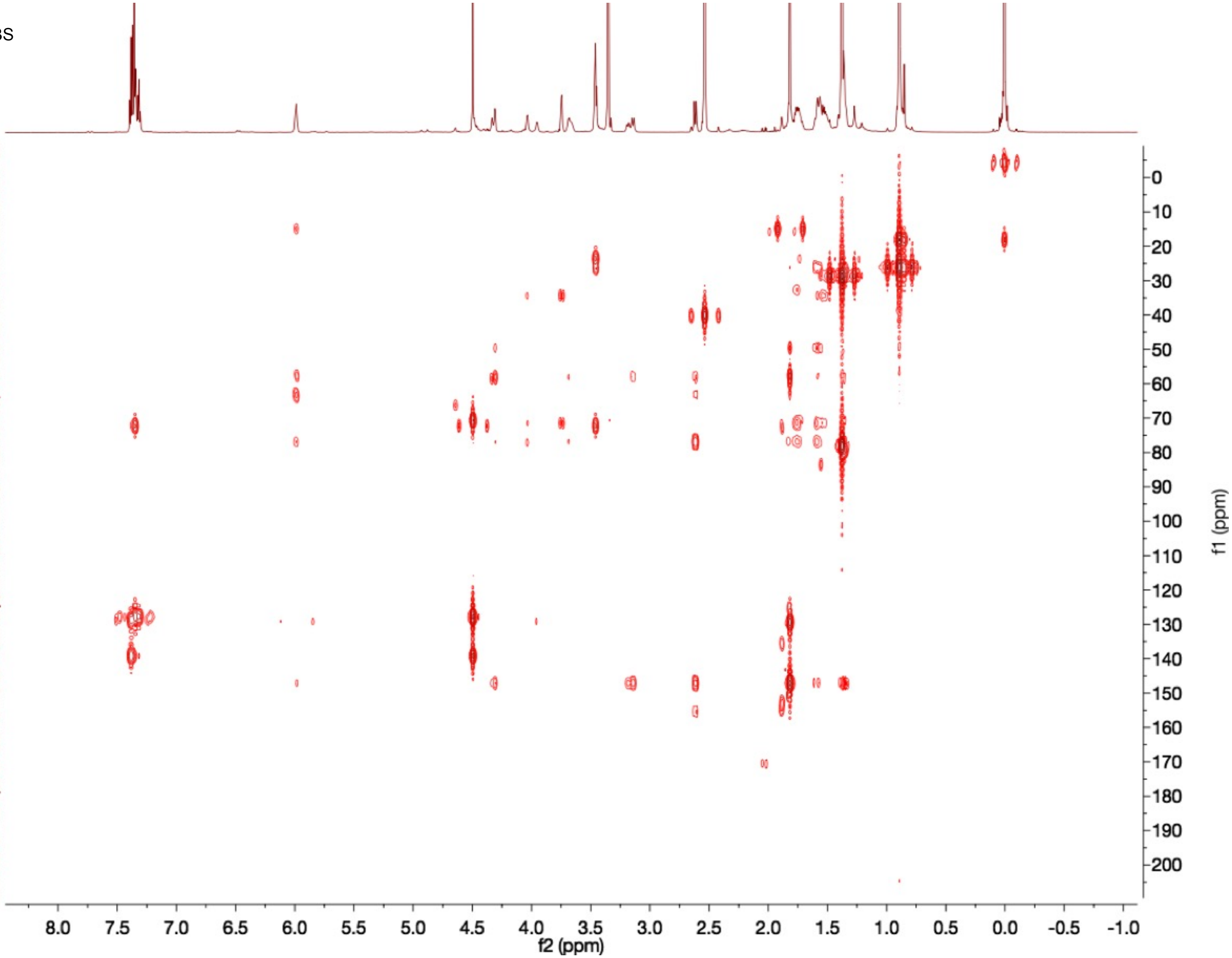

Figure S85. HMBC (DMSO-d_6, 27 °C) Spirocycle **48b**

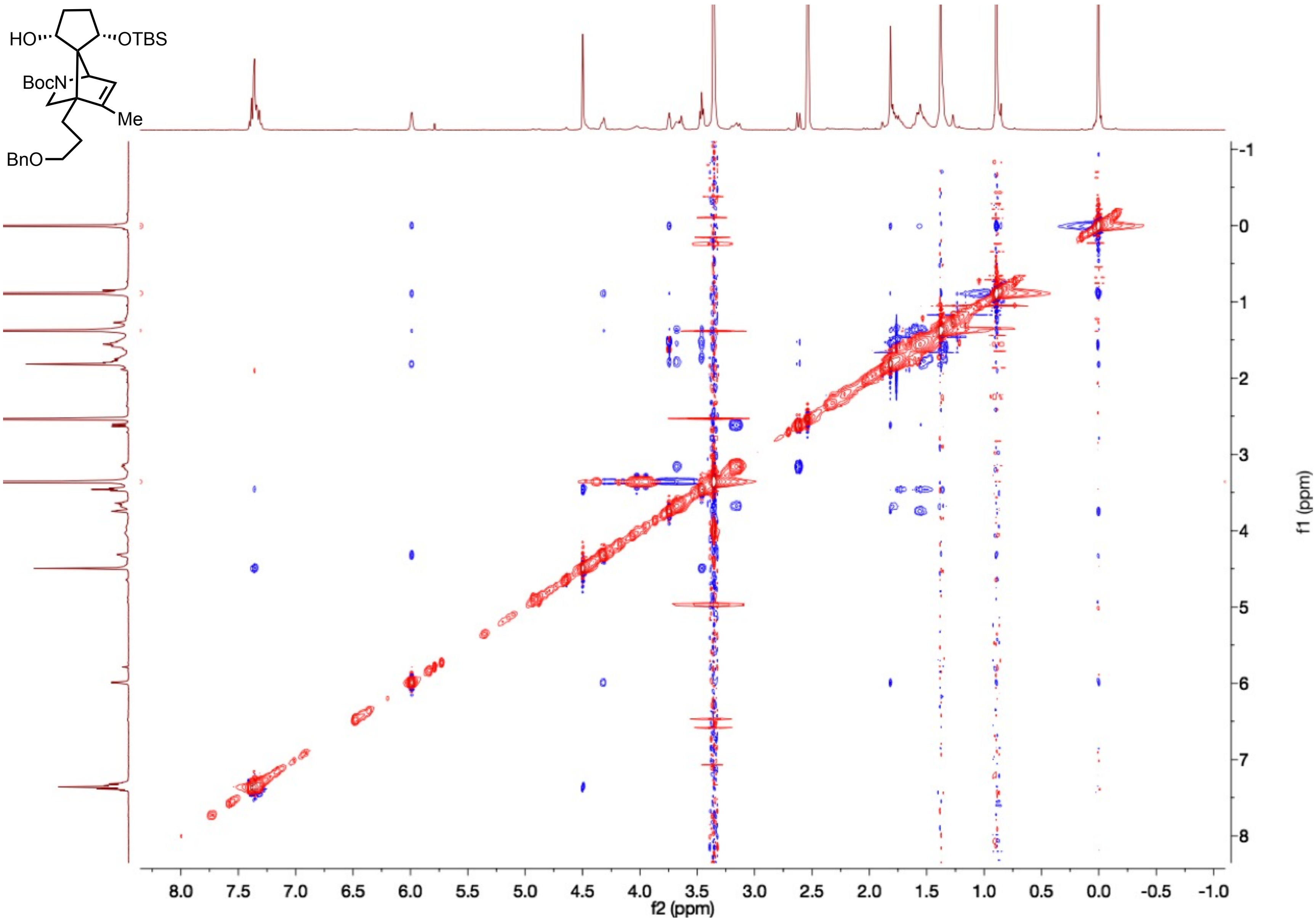

Figure S86. NOESY (DMSO-d_6, 27 °C) Spirocycle **48b**

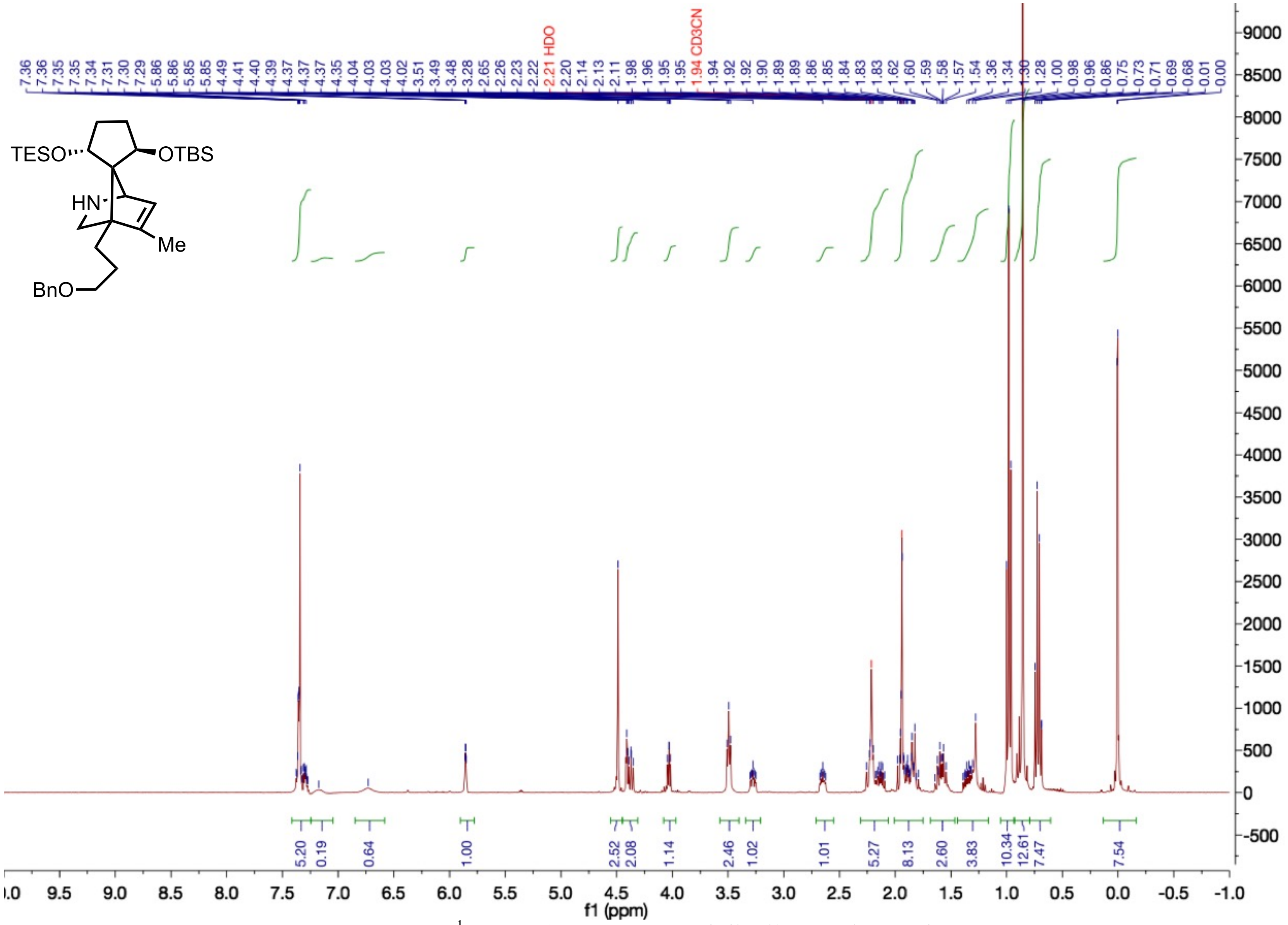

Figure S87. ^{1}H NMR (400 MHz, acetonitrile-d_3) Secondary Amine **49**

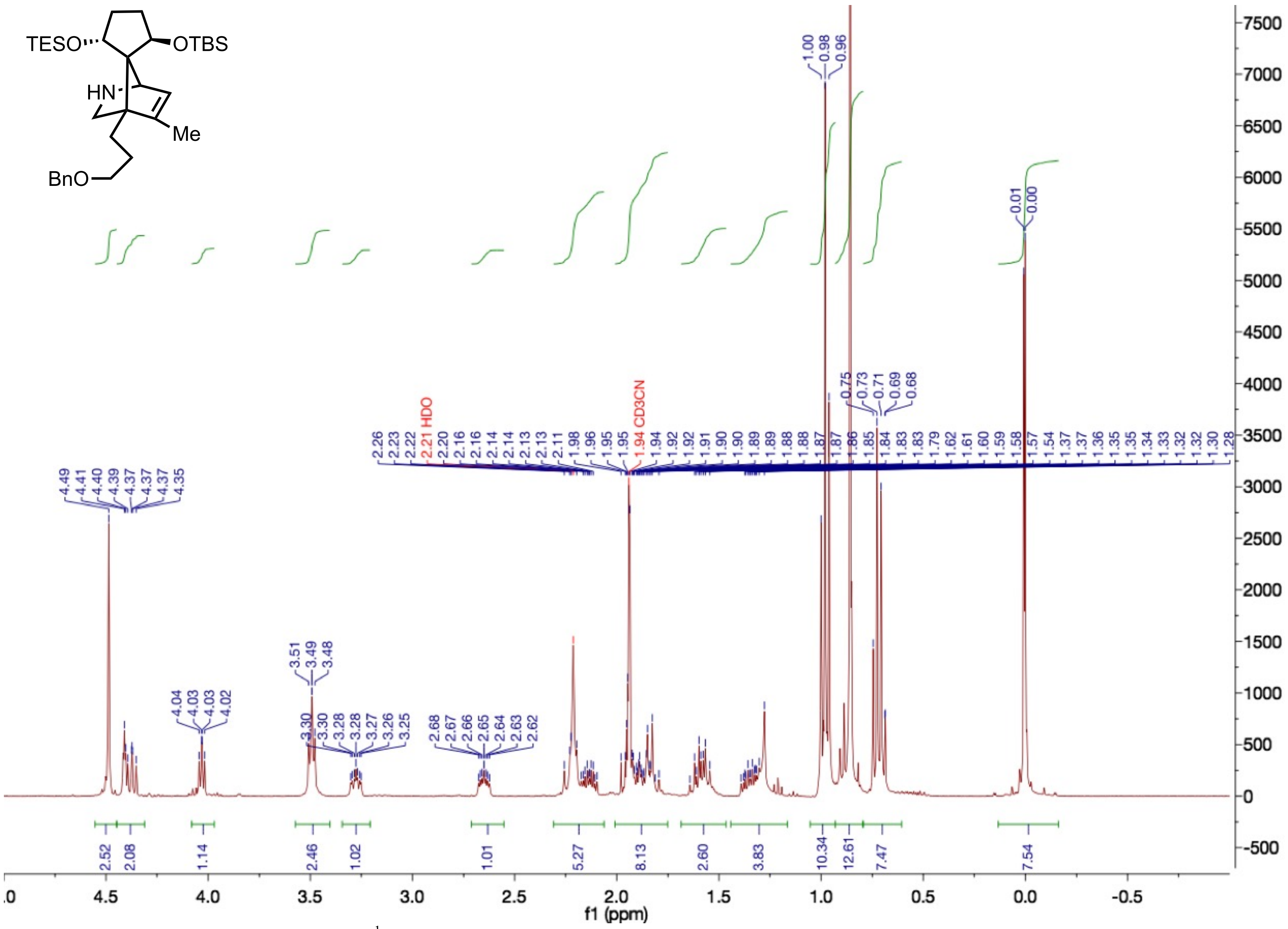

Figure S88. ^1H NMR (400 MHz, acetonitrile-d_3) Secondary Amine **49** (-1 – 5 ppm inset)

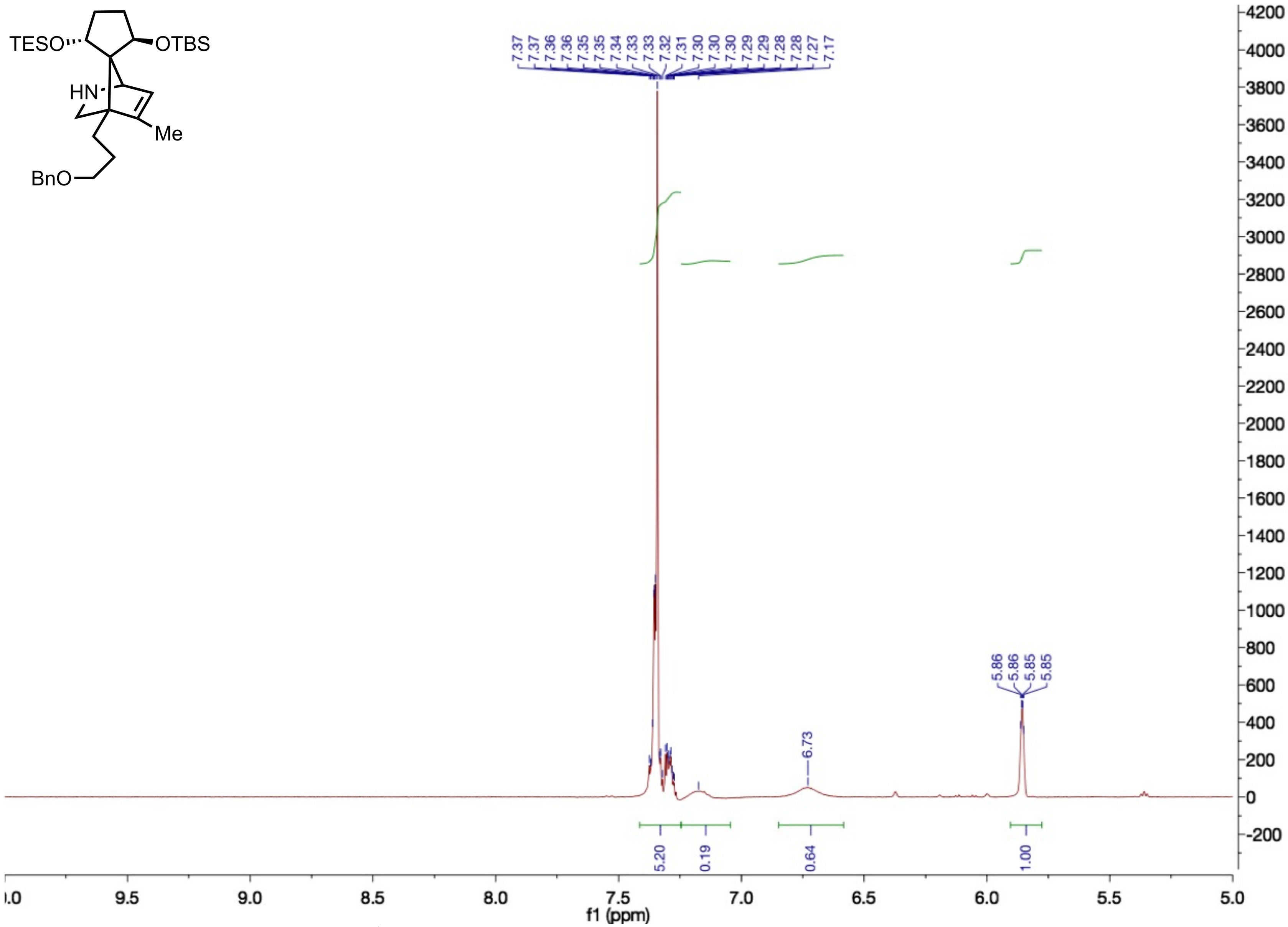

Figure S89. ^{1}H NMR (400 MHz, acetonitrile-d_3) Secondary Amine **49** (5 – 10 ppm inset)

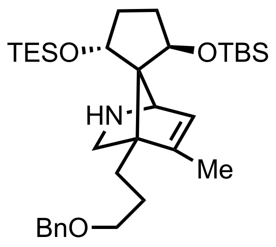

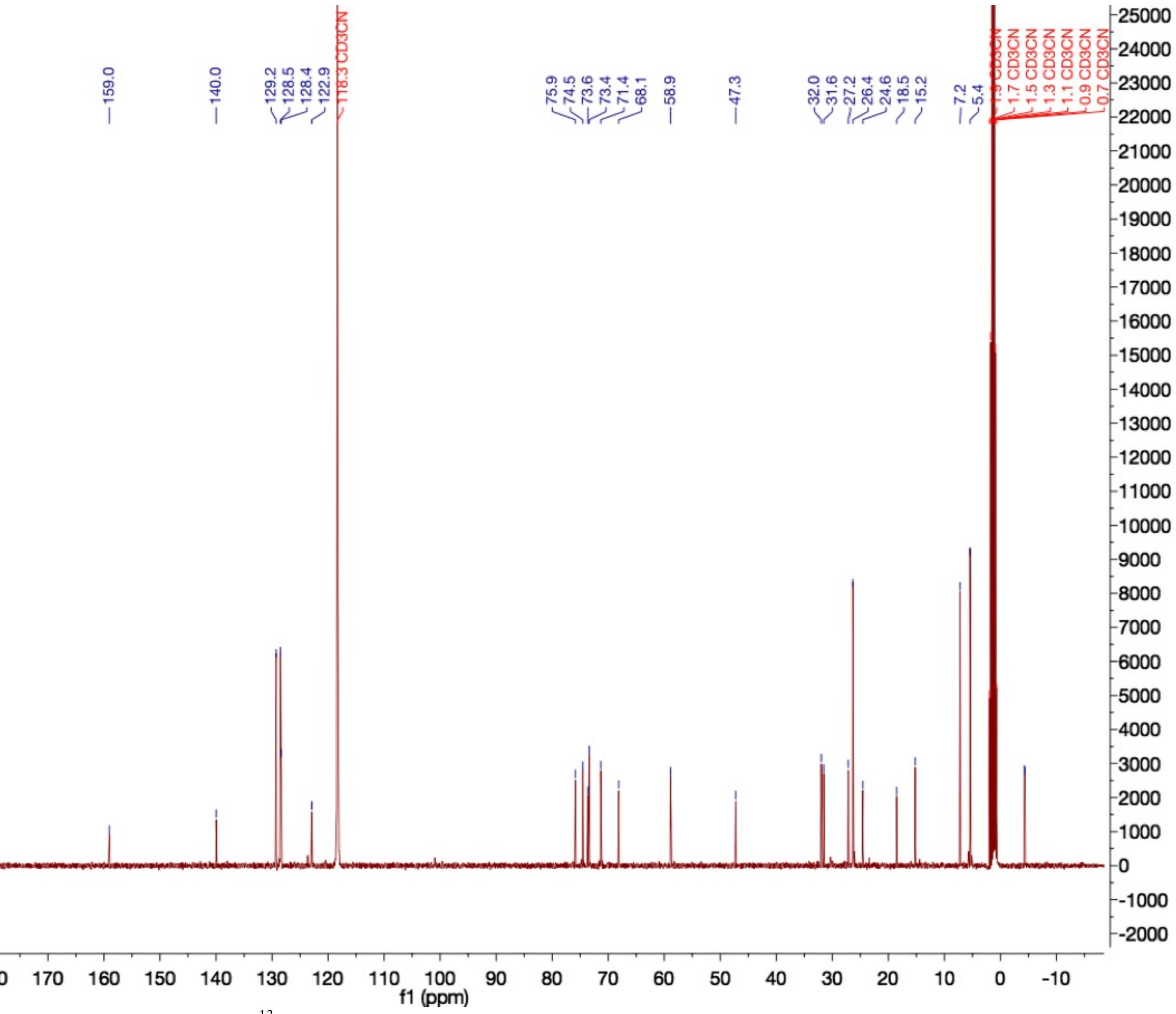

Figure S90. ^{13}C NMR (101 MHz, acetonitrile-d_3) Secondary Amine **49**

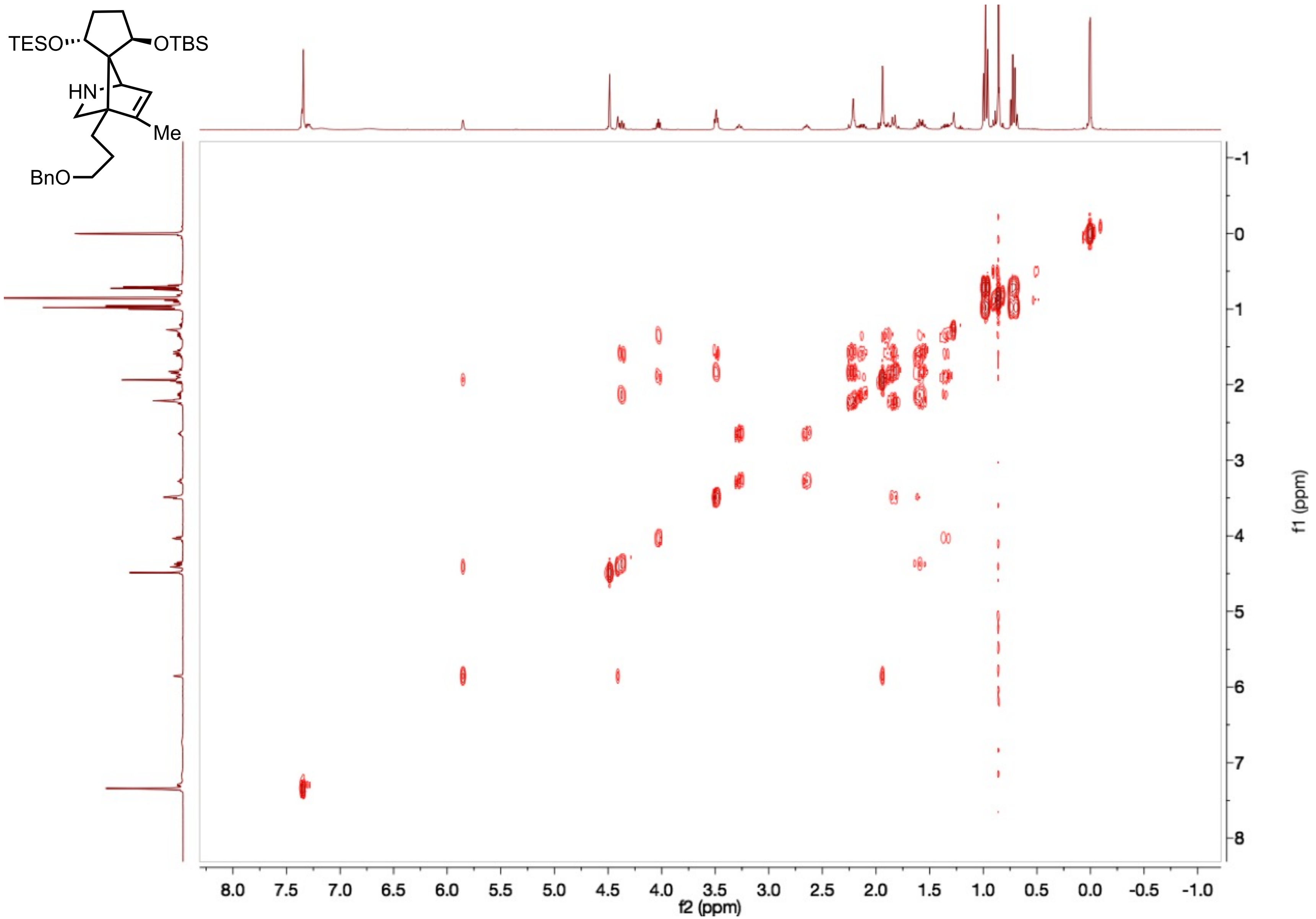

Figure S91. COSY (acetonitrile-d_3) Secondary Amine **49**

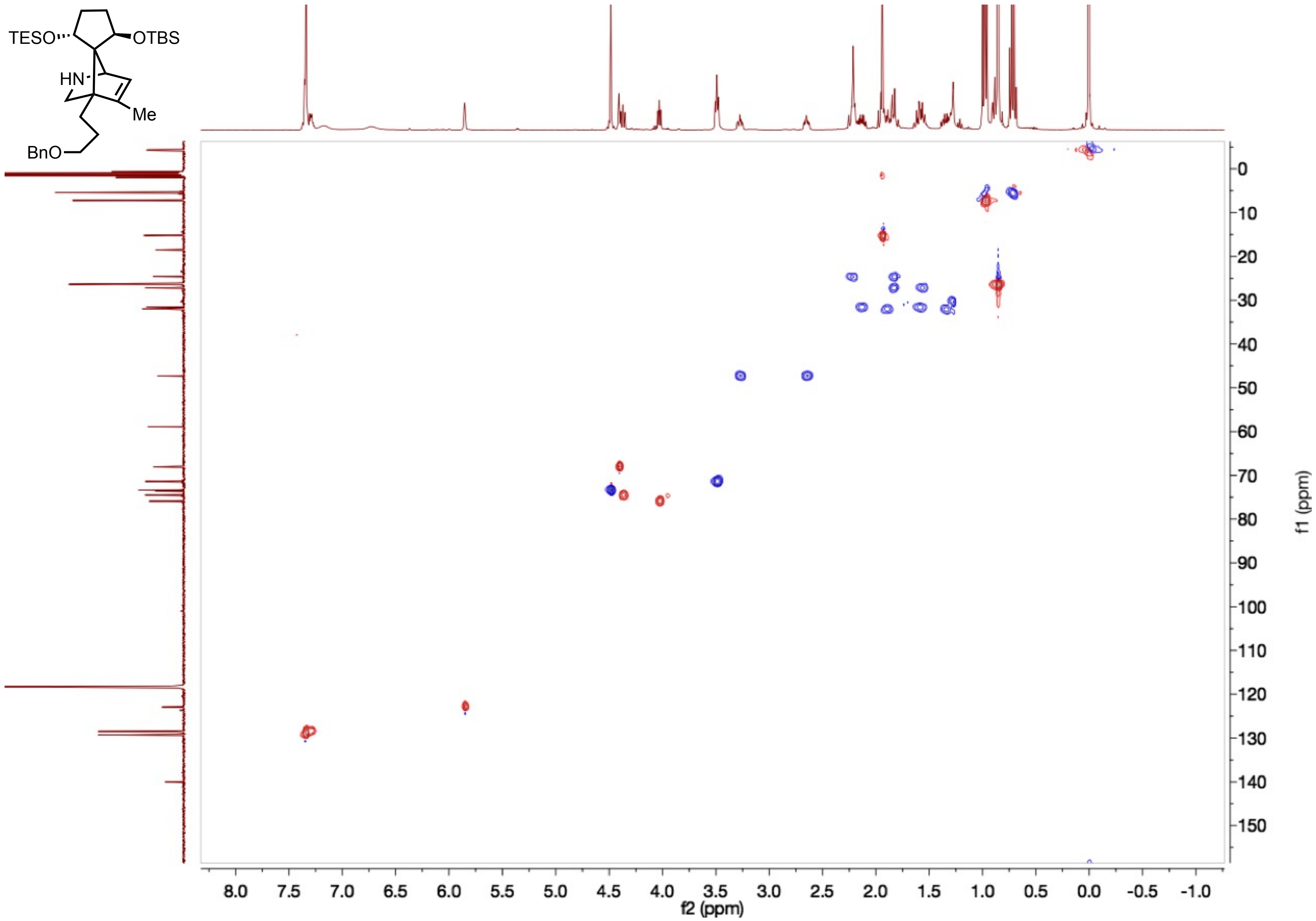

Figure S92. HSQC (acetonitrile-d_3) Secondary Amine **49**

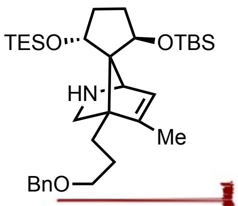

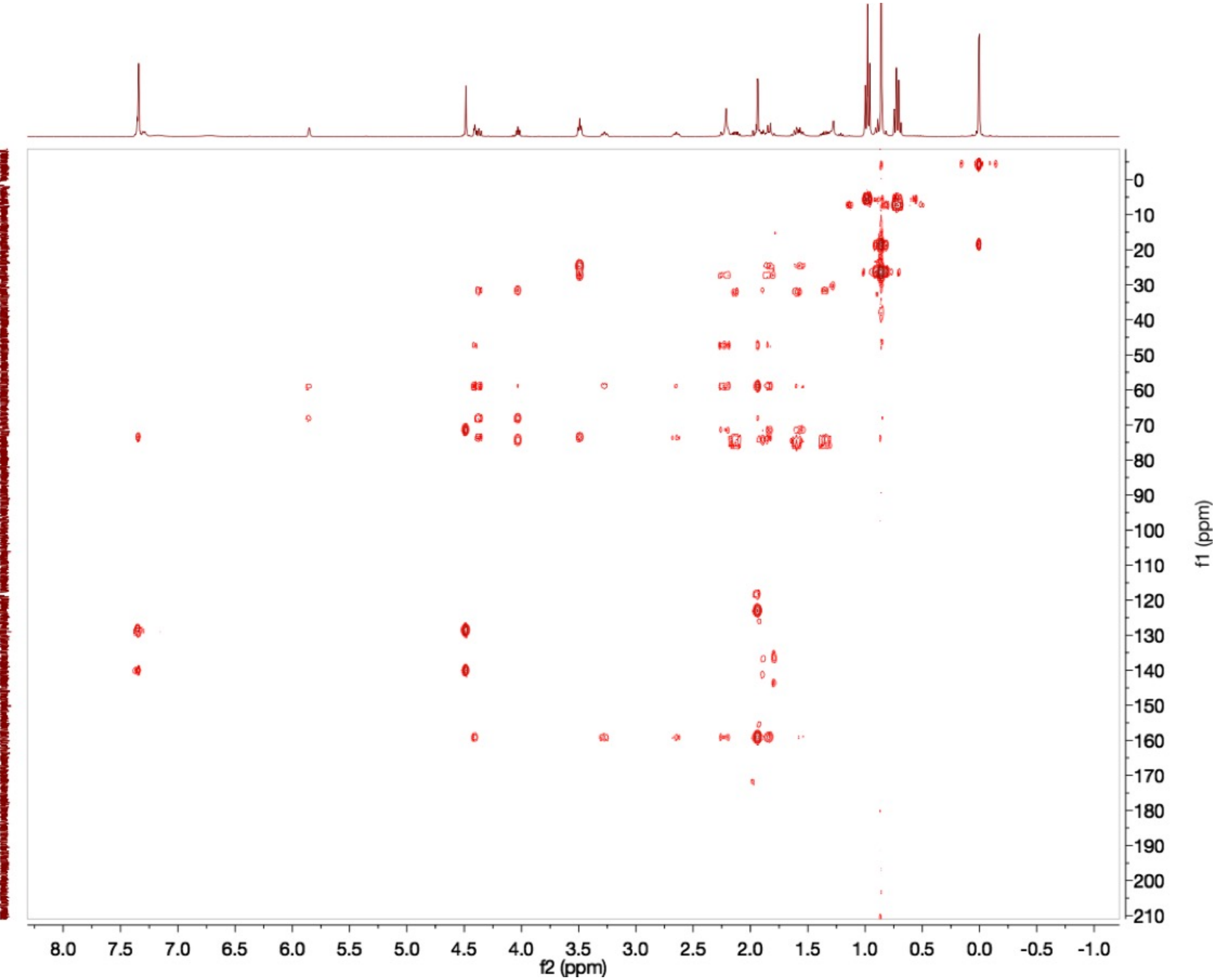

Figure S93. HMBC (acetonitrile-d_3) Secondary Amine **49**

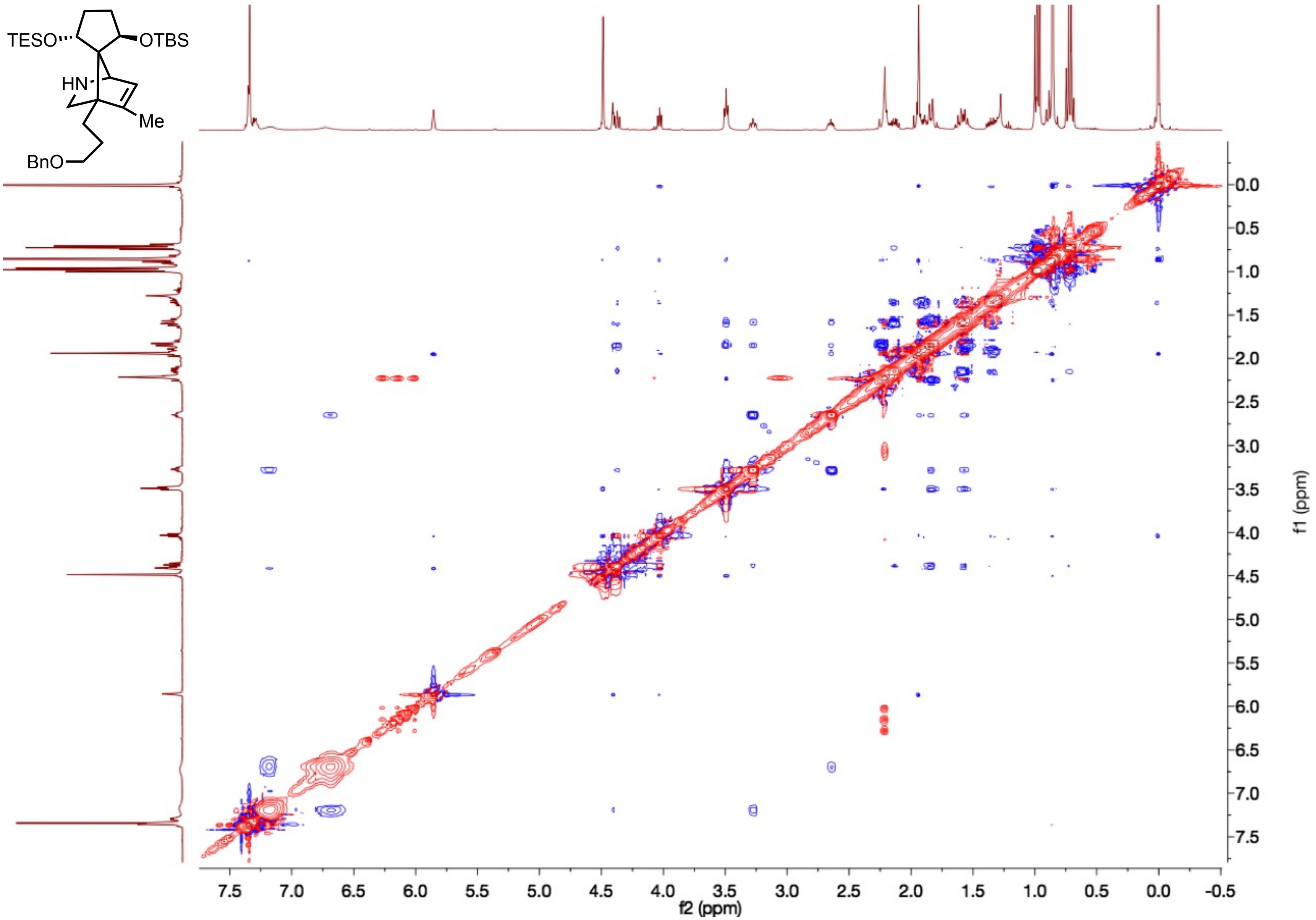

Figure S94. NOESY (acetonitrile-d_3) Secondary Amine **49**
